# Bioequivalence between innovator and generic tacrolimus in liver and kidney transplant recipients: A randomized, crossover clinical trial

**DOI:** 10.1371/journal.pmed.1002428

**Published:** 2017-11-14

**Authors:** Rita R. Alloway, Alexander A. Vinks, Tsuyoshi Fukuda, Tomoyuki Mizuno, Eileen C. King, Yuanshu Zou, Wenlei Jiang, E. Steve Woodle, Simon Tremblay, Jelena Klawitter, Jost Klawitter, Uwe Christians

**Affiliations:** 1 Division of Nephrology and Hypertension, Department of Internal Medicine, University of Cincinnati College of Medicine, Cincinnati, Ohio, United States of America; 2 Division of Clinical Pharmacology, Cincinnati Children’s Hospital Medical Center, Cincinnati, Ohio, United States of America; 3 Department of Pediatrics, University of Cincinnati College of Medicine, Cincinnati, Ohio, United States of America; 4 Division of Biostatistics, Cincinnati Children’s Hospital Medical Center, Cincinnati, Ohio, United States of America; 5 Office of Research and Standards, Office of Generic Drugs, Center for Drug Evaluation and Research, U.S. Food & Drug Administration, Silver Spring, Maryland, United States of America; 6 Division of Transplantation, Department of Surgery, University of Cincinnati College of Medicine, Cincinnati, Ohio, United States of America; 7 iC42 Clinical Research and Development, University of Colorado Anschutz Medical Campus, Aurora, Colorado, United States of America; Royal Derby Hospital, UNITED KINGDOM

## Abstract

**Background:**

Although the generic drug approval process has a long-term successful track record, concerns remain for approval of narrow therapeutic index generic immunosuppressants, such as tacrolimus, in transplant recipients. Several professional transplant societies and publications have generated skepticism of the generic approval process. Three major areas of concern are that the pharmacokinetic properties of generic products and the innovator (that is, “brand”) product in healthy volunteers may not reflect those in transplant recipients, bioequivalence between generic and innovator may not ensure bioequivalence between generics, and high-risk patients may have specific bioequivalence concerns. Such concerns have been fueled by anecdotal observations and retrospective and uncontrolled published studies, while well-designed, controlled prospective studies testing the validity of the regulatory bioequivalence testing approach for narrow therapeutic index immunosuppressants in transplant recipients have been lacking. Thus, the present study prospectively assesses bioequivalence between innovator tacrolimus and 2 generics in individuals with a kidney or liver transplant.

**Methods and findings:**

From December 2013 through October 2014, a prospective, replicate dosing, partially blinded, randomized, 3-treatment, 6-period crossover bioequivalence study was conducted at the University of Cincinnati in individuals with a kidney (*n* = 35) or liver transplant (*n* = 36). Abbreviated New Drug Applications (ANDA) data that included manufacturing and healthy individual pharmacokinetic data for all generics were evaluated to select the 2 most disparate generics from innovator, and these were named Generic Hi and Generic Lo. During the 8-week study period, pharmacokinetic studies assessed the bioequivalence of Generic Hi and Generic Lo with the Innovator tacrolimus and with each other. Bioequivalence of the major tacrolimus metabolite was also assessed. All products fell within the US Food and Drug Administration (FDA) average bioequivalence (ABE) acceptance criteria of a 90% confidence interval contained within the confidence limits of 80.00% and 125.00%. Within-subject variability was similar for the area under the curve (AUC) (range 12.11–15.81) and the concentration maximum (C_max_) (range 17.96–24.72) for all products. The within-subject variability was utilized to calculate the scaled average bioequivalence (SCABE) 90% confidence interval. The calculated SCABE 90% confidence interval was 84.65%–118.13% and 80.00%–125.00% for AUC and C_max_, respectively. The more stringent SCABE acceptance criteria were met for all product comparisons for AUC and C_max_ in both individuals with a kidney transplant and those with a liver transplant. European Medicines Agency (EMA) acceptance criteria for narrow therapeutic index drugs were also met, with the only exception being in the case of Brand versus Generic Lo, in which the upper limits of the 90% confidence intervals were 111.30% (kidney) and 112.12% (liver). These were only slightly above the upper EMA acceptance criteria limit for an AUC of 111.11%. SCABE criteria were also met for the major tacrolimus metabolite 13-O-desmethyl tacrolimus for AUC, but it failed the EMA criterion. No acute rejections, no differences in renal function in all individuals, and no differences in liver function were observed in individuals with a liver transplant using the Tukey honest significant difference (HSD) test for multiple comparisons. Fifty-two percent and 65% of all individuals with a kidney or liver transplant, respectively, reported an adverse event. The Exact McNemar test for paired categorical data with adjustments for multiple comparisons was used to compare adverse event rates among the products. No statistically significant differences among any pairs of products were found for any adverse event code or for adverse events overall. Limitations of this study include that the observations were made under strictly controlled conditions that did not allow for the impact of nonadherence or feeding on the possible pharmacokinetic differences. Generic Hi and Lo were selected based upon bioequivalence data in healthy volunteers because no pharmacokinetic data in recipients were available for all products. The safety data should be interpreted in light of the small number of participants and the short observation periods. Lastly, only the 1 mg tacrolimus strength was utilized in this study.

**Conclusions:**

Using an innovative, controlled bioequivalence study design, we observed equivalence between tacrolimus innovator and 2 generic products as well as between 2 generic products in individuals after kidney or liver transplantation following current FDA bioequivalence metrics. These results support the position that bioequivalence for the narrow therapeutic index drug tacrolimus translates from healthy volunteers to individuals receiving a kidney or liver transplant and provides evidence that generic products that are bioequivalent with the innovator product are also bioequivalent to each other.

**Trial registration:**

ClinicalTrials.gov NCT01889758.

## Abstract 2

In a randomized 3-treatment crossover clinical trial, Rita Alloway of the University of Cincinnati and colleagues establish bioequivalence between 3 versions of the drug tracolimus (a brand name and 2 disparate generics) in kidney and liver transplant recipients.

## Author summary

**Why was this study done?:**

- Consensus documents developed by professional transplantation societies worldwide have cautioned the use of generic immunosuppressants such as tacrolimus in individuals with a solid organ transplant. Reasons have included repeated switching between innovator (that is, “brand” products) and generics and among different generics, especially when not controlled by physicians.
- There was uncertainty in the transplant community as to whether tacrolimus generics that are bioequivalent to the innovator are also bioequivalent to each other.
- For market approval, generic drug products of the narrow therapeutic index drug tacrolimus had to be studied only in healthy individuals and not in the much more complex organ transplant population.

**What did the researchers do and find?:**

- We performed a randomized, prospective, 3-treatment, 6-period, crossover, replicate dose study in individuals with a kidney or liver transplant.
- Thirty-five individuals with a kidney transplant and 36 individuals with a liver transplant receiving tacrolimus were studied to compare the tacrolimus time concentration profiles of 3 different products in their blood: namely, Innovator (Prograf), Generic Hi (Sandoz), and Generic Lo (Dr. Reddy) 1.0 mg tacrolimus capsules.
- Generic products were selected based upon pharmacokinetic data from healthy volunteer studies since bioequivalence data were not available in individuals with an organ transplant.
- We observed bioequivalence based on average bioequivalence and scaled average bioequivalence criteria in individuals after kidney or liver transplant between tacrolimus innovator and the 2 generics on the US market as well as between the 2 generics.

**What do these findings mean?:**

- Similar tacrolimus exposure is expected in individuals with a kidney or liver transplant when receiving Prograf, Sandoz generic, or Dr. Reddy’s generic tacrolimus.

## Introduction

Most individuals receiving a solid organ transplant require lifelong immunosuppression. Switching to generic immunosuppressants may lead to significant savings and improved adherence [1,2], which is essential for long-term graft survival [3]. The current US Food and Drug Administration (FDA) generic drug approval process has performed well [4]. However, concerns persist regarding whether 2-way crossover studies in healthy individuals using conventional average bioequivalence (ABE) acceptance criteria of a 90% confidence interval contained within the confidence limits of 80.00% to 125.00% are a valid approach for generic immunosuppressant approval for use after transplantation [5,6]. This debate started when cyclosporine generics were developed over 15 years ago [7,8] and was reinvigorated when tacrolimus generics were approved. Consensus documents developed by professional societies from the US, Europe, and Canada [9–12] have cautioned against generic immunosuppressant use, citing (1) the lack of data in transplant recipients, especially “high risk” transplant recipients; (2) the need to implement stricter bioequivalence standards, as tacrolimus is a narrow therapeutic index (NTI) drug for which small changes in dose or exposure can result in therapeutic failure or toxicity; and (3) the lack of bioequivalence data between generics. Molnar et al. published a systematic review and meta-analysis to compare the clinical efficacy and bioequivalence of generic immunosuppressive drugs in individuals with a transplant and concluded that high-quality data were lacking. The authors went further to state that given the serious consequences of rejection and allograft failure, well-designed studies on the bioequivalence and safety of generic immunosuppression in individuals with a transplant are needed [13].

Differing worldwide bioequivalence regulatory standards for NTI drugs make it difficult to interpret bioequivalence study results [14–16]. For NTI drugs, the European Medicines Agency (EMA) requires a narrower 90.00%–111.11% acceptance criterion for the area under the curve (AUC, a measure of actual body exposure to a drug) but uses the usual 80.00%–125.00% acceptance criterion for the concentration maximum (C_max_) for NTI drugs [14]. Health Canada has adopted standards similar to those of EMA, with an AUC acceptance criterion of 90.00%–112.00% [15]. The FDA has classified tacrolimus as an NTI drug and recommended the scaled average bioequivalence (SCABE) approach to determine bioequivalence [16,17]. With this SCABE approach, both generic and innovator products are given twice with fully replicating measurements in each individual. An innovator pharmaceutical product is the one that was first authorized for marketing on the basis of quality, safety and efficacy. This allows for determination of within-subject variability, which is then used for scaling the bioequivalence acceptance limits based on the reference product for all products tested. This approach creates more stringent bioequivalence criteria: (1) the ABE limits for both AUC and C_max_ are narrowed based on the within-subject variability of the reference product and are never wider than 80.00%–125.00%, and (2) the within-subject variabilities of all products are compared to each other.

Tacrolimus has a complex pharmacokinetic profile, as it is metabolized mainly by hepatic and intestinal cytochrome (CYP) P4503A enzymes and over 90% is eliminated as metabolites. CYP3A5 expressers (CYP3A5 *1/*1 and CYP3A5 *1/*3) are considered patients who “poorly absorb” and may exhibit higher within-subject variability of tacrolimus pharmacokinetics than nonexpressers (CYP3A5 *3/*3). These genetic differences have been associated with poorer outcomes. Tacrolimus is also a substrate of the drug efflux protein p-glycoprotein, ABCB1, thus impacting tacrolimus exposure [17–24]. Because of these complex metabolic and transport processes, stringent ABE testing is used to ensure product excipients do not impact these processes.

Given the aforementioned public concerns [9–12], we hypothesized that 2 generic tacrolimus products currently on the US market meet both FDA ABE and SCABE limits in individuals with a kidney or liver transplant when compared to innovator tacrolimus and when compared to each other in a high-quality study. All products met these bioequivalence criteria. In addition, we applied EMA NTI ABE criteria, and all products met the criteria except for one that narrowly fell above the AUC limit.

## Methods

### Study conduct and oversight

The study design was developed in collaboration with the American Society of Transplantation (Mount Laurel, New Jersey, US), the American Society of Transplant Surgeons (Arlington, Virginia, US), and the FDA. Individuals were recruited from the University of Cincinnati Medical Center and The Christ Hospital in Cincinnati. This trial adhered to the Declaration of Helsinki and was approved by local institutions’ review boards (2012–4891) and the FDA Research Involving Human Subjects Committee (13-018D). All individuals provided written informed consent. The study was monitored locally and by the FDA and registered on clinicaltrials.gov (NCT-01889758). Methodologies for tacrolimus quantification in whole blood (Tables A–F in S1 Appendix and Fig A in S1 Appendix) and genetic polymorphism testing (Table G in S1 Appendix) are described. The study protocol and statistical analysis plan are also included in the supporting information as S1 Text and S2 Text. Changes from the prespecified analysis plan included the analysis of the minimum concentration (C_min_) in lieu of C_0_ and C_12_ tacrolimus concentrations as appropriate based upon guidance documents, and dose normalization was not performed because each individual received the same dose in all treatment periods. No interim analyses were conducted prior to these data analyses.

### Test product selection

At study initiation, 5 FDA-approved generic tacrolimus products were available in addition to the innovator product, Prograf (Astellas, Northbrook, Illinois) [25]. Abbreviated New Drug Applications (ANDAs) are submitted to the FDA for all generic products and represent the only data readily available for all products. ANDA data include, but are not limited to, pharmacokinetic data in healthy volunteers that demonstrate bioequivalence between the innovator and generic products by evaluating the pharmacokinetic parameters of AUC and C_max_. Product composition, manufacturing, and pharmacokinetic data for all approved generics were reviewed to identify the 2 most disparate generics. ANDA pharmacokinetic data from each product are provided in Table H-I in S1 Appendix. Additional manufacturing comparisons are summarized in Table J in S1 Appendix [26–31]. One tacrolimus product (Panacea; Baddi, India) was FDA-approved but not commercially available, and it was therefore excluded. Pharmacokinetic parameters of AUC and C_max_ were examined for the greatest difference between the generic and the innovator product as being the most disparate and named Generic Hi and Generic Lo. Sandoz tacrolimus (Sandoz, Princeton, New Jersey, US) was identified as Generic Hi based upon higher point estimates and higher upper 90% confidence interval compared to innovator. Dr. Reddy tacrolimus (Dr. Reddy, Bachupally, India) was identified as Generic Lo based upon lower point estimates and lowest lower 90% confidence interval compared to innovator. Single tacrolimus 1 mg capsule lots (the most frequently prescribed dosage strength) of Innovator (Prograf), Generic Hi (Sandoz, Princeton, New Jersey, US), and Generic Lo (Dr. Reddy, Bachupally, India) were purchased from a pharmacy wholesaler and controlled by the University of Cincinnati Medical Center Investigational Drug Services. The University of Iowa Pharmaceuticals (Iowa City, Iowa, US), iC42 Clinical Research and Development (Aurora, Colorado, US), and the FDA independently performed dissolution, purity, and content uniformity testing according to applicable US Pharmacopeia Convention guidelines [29]. Similar results were obtained by both groups. The FDA results are reported in Tables K–O in S1 Appendix.

### Study population, randomization and blinding

**Fig 1:**
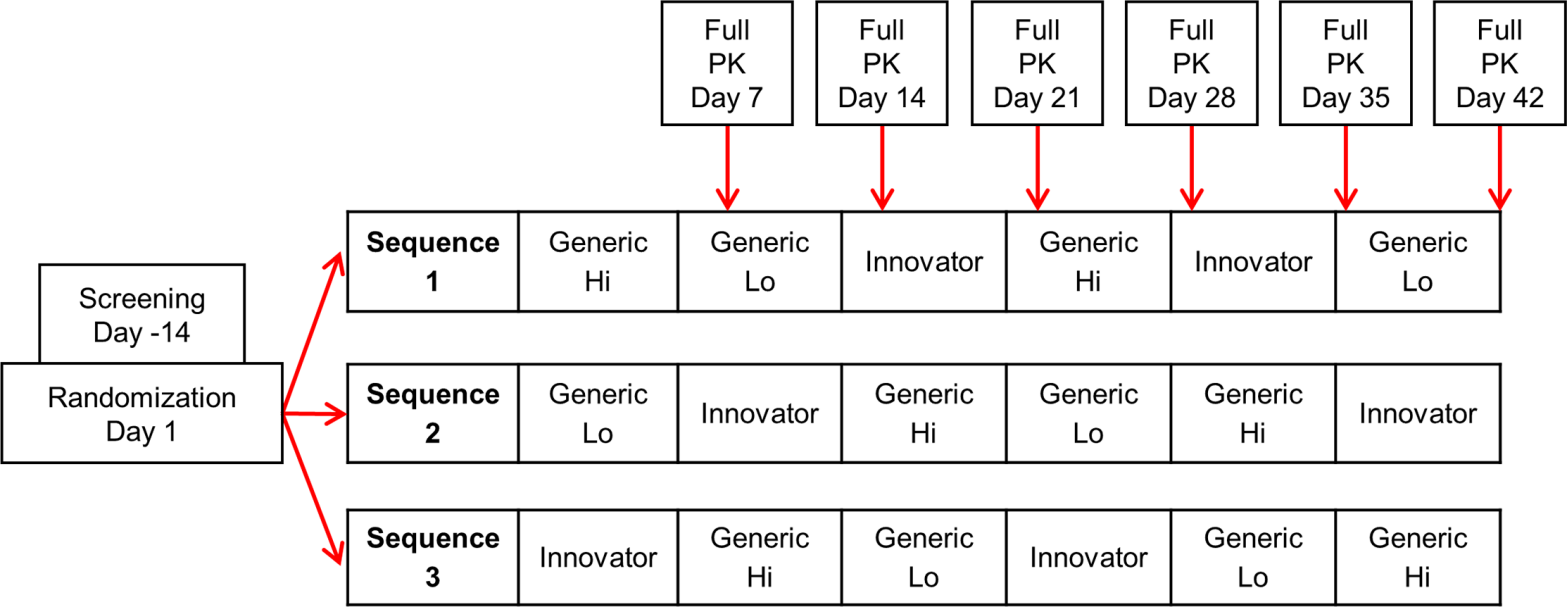
Randomization sequence and study design. PK, pharmacokinetics.

Individuals with a kidney or liver transplant included in the present study were at least 18 years old, with stable organ function and no evidence of rejection. Said individuals were at steady state and on stable doses of immunosuppressants including tacrolimus with no expected changes to their immunosuppressive drug regimens to eliminate confounders that occur early post-transplant or during times of rejection. Other eligibility criteria are listed in Section F of S1 Appendix. Study participants were stratified by organ type and randomized to 1 of 3 treatment sequences, each including 2 periods of Innovator (Prograf), Generic Hi (Sandoz), and Generic Lo (Dr. Reddy) (Fig 1). The replicate dosing design of administering each product twice allowed for analysis of within-subject variability by product. An independent statistician generated a randomization list using SAS (version 9.03, SAS Institute, Cary, North Carolina, US) and provided it to the investigational drug pharmacist. Eligible transplant recipients were recruited from 2 clinical sites, but all screening visits occurred at the University of Cincinnati Medical Center. The investigational drug pharmacist consecutively assigned individuals to a treatment sequence as received, independent of site. All parties were blinded to the randomization sequence allocation until after the pharmacokinetics (PK) analysis was completed. (Additional blinding information is located in Section G of S1 Appendix.)

### Treatment protocol

Eligible transplant recipients were screened via telephone. Potential study participants completed a baseline visit, including written informed consent and laboratory, physical, and genetic polymorphism testing (including CYP3A5*3, CYP3A4*1B, CYP3A4*22, POR*28, and 3 *ABCB1* SNPs) [18–24]. The study assessment schedule is shown in Table P in S1 Appendix. Study participants were seen 2 weeks later for baseline laboratory examination and randomization and were provided with study drug. Medications were dispensed with a Medication Event Monitoring System (MEMS, AARDEX, Palo Alto, California) bottle cap for electronic monitoring of study medication access. Pill counts were recorded at each pharmacokinetic visit. Individuals were excluded from the analysis if they were nonadherent within 48 hours of PK assessment (Section I in S1 Appendix).

Tacrolimus doses remained constant during the entire study period. After a 7-day treatment period, individuals underwent a 12-hour tacrolimus pharmacokinetic assessment with dosing and sampling times strictly controlled and monitored. The 7-day treatment period was adequate to reach steady state based upon observed half-life in individuals receiving a kidney or liver transplant. Fifteen tacrolimus samples were collected at C_0_ (before the morning dose) and at 20, 40, 60, 80, 100, 120, 140, and 160 minutes and 3, 4, 5, 6, 8, and 12 hours following dosing. Six 12-hour pharmacokinetic assessments were completed after 7 days of administration of each tacrolimus product. All samples were analyzed for tacrolimus and metabolites using a validated high-performance liquid chromatography-tandem mass spectrometry (LC-MS/MS) assay (Sections A and B in S1 Appendix). All individuals fasted until a standardized meal was allowed after the 4-hour and 12-hour sample collection. At each visit, study participants were assessed for safety lab tests, adverse events, medication adherence, and medication regimen changes (Table P in S1 Appendix).

### Statistical, pharmacokinetic, and bioequivalence analysis

Data were stored electronically using a REDCap database [32], including all laboratory, patient diary, and bioanalytical data. Data monitoring and analysis plans defined a priori were executed. After data were monitored and all queries resolved, the database was locked. Only individuals completing all 6 pharmacokinetic study periods were analyzed. Actual sample collection times were used for analysis. For each type of organ transplant, a sample size of 24 individuals was required to achieve 90% statistical power for concluding bioequivalence in crossover trials at an alpha of 0.05 using standard bioequivalence limits of 80%–125% and assuming a true difference of 0 [16,33,34].

The primary outcome was to determine whether Innovator (Prograf), Generic Hi (Sandoz), and Generic Lo (Dr. Reddy) tacrolimus products were bioequivalent with each other by comparing their AUC and C_max_ using conventional ABE limits (the 90% CIs of the ratio of geometric means of the 2 products for C_max_ and AUC were within the range of 80%–125%) [34] and reference SCABE limits [16]. The observed C_min_ represented the minimum concentration and was analyzed in lieu of C_0_ or C_12._ Observed T_max_ represented the actual time at which the maximum concentration was measured. Each patient served as his or her own control; therefore, dose normalization was not required. The investigators and the FDA independently analyzed the primary end-point data using WinNonlin software (version 6.4. Phoenix, Certara, Princeton, New Jersey, US) and SAS (version 9.3, SAS Institute, Cary, North Carolina), respectively. The investigators’ analysis results are reported.

Secondary outcomes included ABE and SCABE assessment in prespecified subgroups and assessments for safety and efficacy. Subgroups in which there were at least 6 recipients and the statistical model converged are reported. The prespecified subgroups included recipient sex; age; African-American race; diabetes; CYP3A4/5, POR 28, and ABCB1 polymorphisms; and donor CYP3A5, as these subgroups are reported to strongly influence tacrolimus concentrations [18]. The study was not powered to identify differences by subgroup. In addition, pharmacokinetic parameters of the primary tacrolimus metabolite, 13-O-desmethyl tacrolimus, were compared.

Safety was assessed at baseline and weekly throughout the study by complete metabolic panels and complete blood cell count with differential. Baseline and weekly assessments included markers of transplant function in individuals with a kidney transplant (creatinine) or liver transplant (aspartate aminotransferase [AST], alanine aminotransferase [ALT], and alkaline phosphatase). To compare kidney and liver function tests among products, a mixed effects model was run with a term designating the product received in each period and a random subject term and using the Tukey’s honest significant difference (HSD) test for multiple comparisons, where needed [35]. The study was not powered to assess differences in transplant organ function. Total daily tacrolimus dose data were summarized using means and standard deviation, and between-group differences were analyzed using the *t* test. Reports of adverse events were collected at each visit and coded utilizing the Common Terminology Criteria for Adverse Events (CTCAE v4.0) [36]. The Exact McNemar test for paired categorical data with adjustments for multiple comparisons was used to compare adverse event rates among the products [37]. No statistically significant differences among any pairs of products were found for any adverse event code or for adverse events overall.

ABE was assessed within each transplant organ group (i.e., kidney and liver) by using a mixed effects analysis of variance model for a 6-period crossover design with the log_e_-transformed pharmacokinetic parameter estimates (AUC, C_max_, and C_min_) as the response variable. Fixed effect terms in the model included sequence, period, and treatment. Random effects included subject nested within sequence. The error variance structure accounted for the repeated measures of treatments within each subject. Two-sided 90% confidence intervals using the differences in least square means and the appropriate error terms from the model were calculated for each pairwise assessment of bioequivalence. The estimates and end points of the confidence intervals were back-transformed to obtain the ratios of the parameters being assessed for bioequivalence and the corresponding 90% confidence interval for the ratios. If the entire confidence interval was contained within the range of 80% to 125%, then ABE was established.

To assess SCABE, the estimate of within-subject variability for each treatment was obtained by using a mixed effects model within each organ type and treatment group. Fixed effect terms included sequence, replication (i.e., first or second), and sequence-by-replication interaction. Random effect terms include subject nested within sequence. This model provided estimates of the within-subject variability for each treatment, and these were then used to adjust the bioequivalence end points to obtain the SCABE limits and calculate the criterion bound in accordance with FDA guidance for NTI drugs [16]. SCABE was concluded if each of the following criteria were met: (1) the 2-sided 90% confidence interval calculated for the ABE assessment must fall entirely within the SCABE limits, (2) the criterion bound must be less than 0, and (3) the upper 90% confidence limit for the ratio of the within-subject variabilities for the 2 treatments being assessed must be less than 2.5 [16].

## Results

### Study individuals

**Fig 2:**
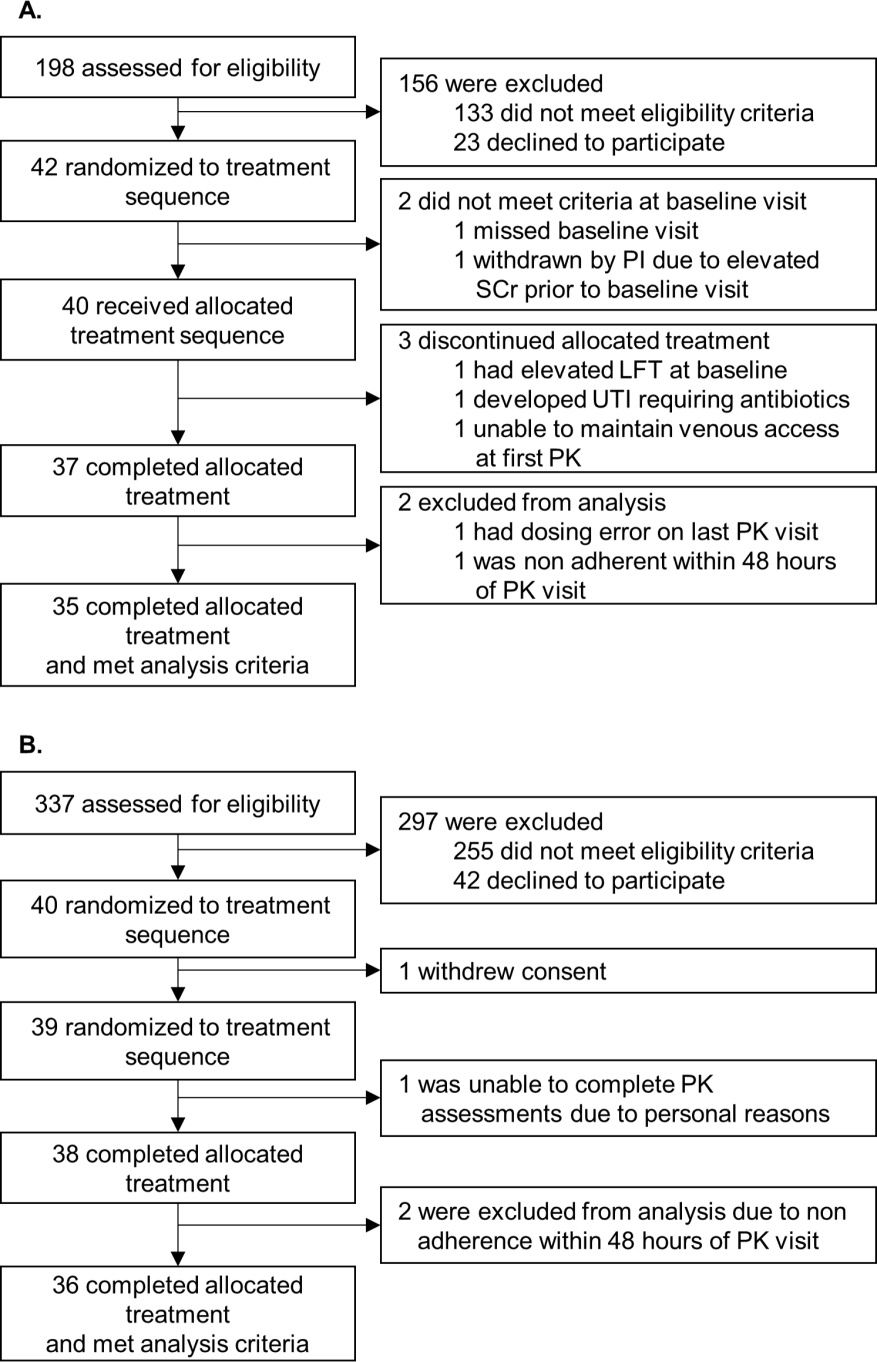
**Kidney (A) and liver (B) Consolidated Standards Of Reporting Trials (CONSORT) flow diagram.** (A) Individuals with a kidney transplant. (B) Individuals with a liver transplant. LFT, liver function tests; PI, principal investigator; PK, pharmacokinetics; SCr, serum creatinine; UTI, urinary tract infection.

From December 2013 through October 2014, 42 individuals with a kidney transplant and 40 individuals with a liver transplant were consented and followed as per the study protocol. Seventy-one individuals were analyzable (kidney, *n* = 35; liver, *n* = 36). The most frequent causes of noneligibility during screening were (1) a greater than 3-hour drive from the study center, (2) receiving the 0.5-mg tacrolimus dosage form, (3) renal function < 35 ml/min, (4) not receiving tacrolimus, (5) a history of multiorgan transplant (i.e., kidney and pancreas or liver and kidney), (6) documented nonadherence, or (7) a history of cancer. Moreover, individuals with a liver transplant and active hepatitis C were not eligible. A complete list of inclusion and exclusion criteria is in Section F in S1 Appendix. Consolidated Standards Of Reporting Trials (CONSORT) flow diagrams (Fig 2) and checklist (S1 CONSORT Checklist) are provided.

The demographic and baseline characteristics reported to impact the tacrolimus pharmacokinetics of the analyzed individuals are summarized in Table 1 and were similar to intent-to-treat individuals (Table Q in S2 Appendix). Most individuals received tacrolimus, mycophenolate, and corticosteroid-free immunosuppression. Immunosuppressive regimens, including tacrolimus doses, remained constant throughout the 6-week study. Individuals with a kidney or liver transplant received a median mg/day (IQR) tacrolimus dose of 5.0 (4.0–8.0) or 4.0 (3.0–6.0), respectively. During the study, no patient initiated, discontinued, or changed doses of known CYP3A inhibitors or inducers that could impact pharmacokinetic observations.

**Table 1:** Demographic and baseline characteristics of analyzed study individuals with a kidney or liver transplant.

Variable	Individuals with a kidney transplant (*n* = 35)	Individuals with a liver transplant (*n* = 36)
**Age (y) median (IQR)**	52 (39.0–59.0)	57 (48.5–61.0)
**Gender (male)**	65.7%	47.2%
**Race (African American)**	17.1%	2.8%
**Transplant donor type**		
**Deceased**	25.7%	100%
**Living related**	34.3%	0%
**Living unrelated**	40%	0%
**Time post-transplant (y) median (IQR)**	4.5 (3.3–7.9)	3.2 (1.8–6.9)
**Presence of diabetes (%)**	37.1	27.8
**Maintenance immunosuppression**		
**Steroids (%)**	14.3	8.3
**Mycophenolic acid (%)**	100	88.9
**Median mg/d tacrolimus dose (IQR)**	5.0 (4.0–8.0)	4.0 (3.0–6.0)

### Adherence monitoring

Adherence was evaluated using the MEMS system to insure the quality of the pharmacokinetic evaluations. Adherence was defined as the degree to which the number of medication doses taken each day matched the number of prescribed doses. Over 6 weeks, MEMS-based adherence was 99.75% (range: 97.67%–100%). Three individuals were excluded from the analysis due to nonadherence (kidney, *n* = 1; liver, *n* = 2) (Fig 2). MEMS-based adherence rates are reported in Tables R and S in S2 Appendix.

### ABE, SCABE, and within-subject variability comparison

**Fig 3:**
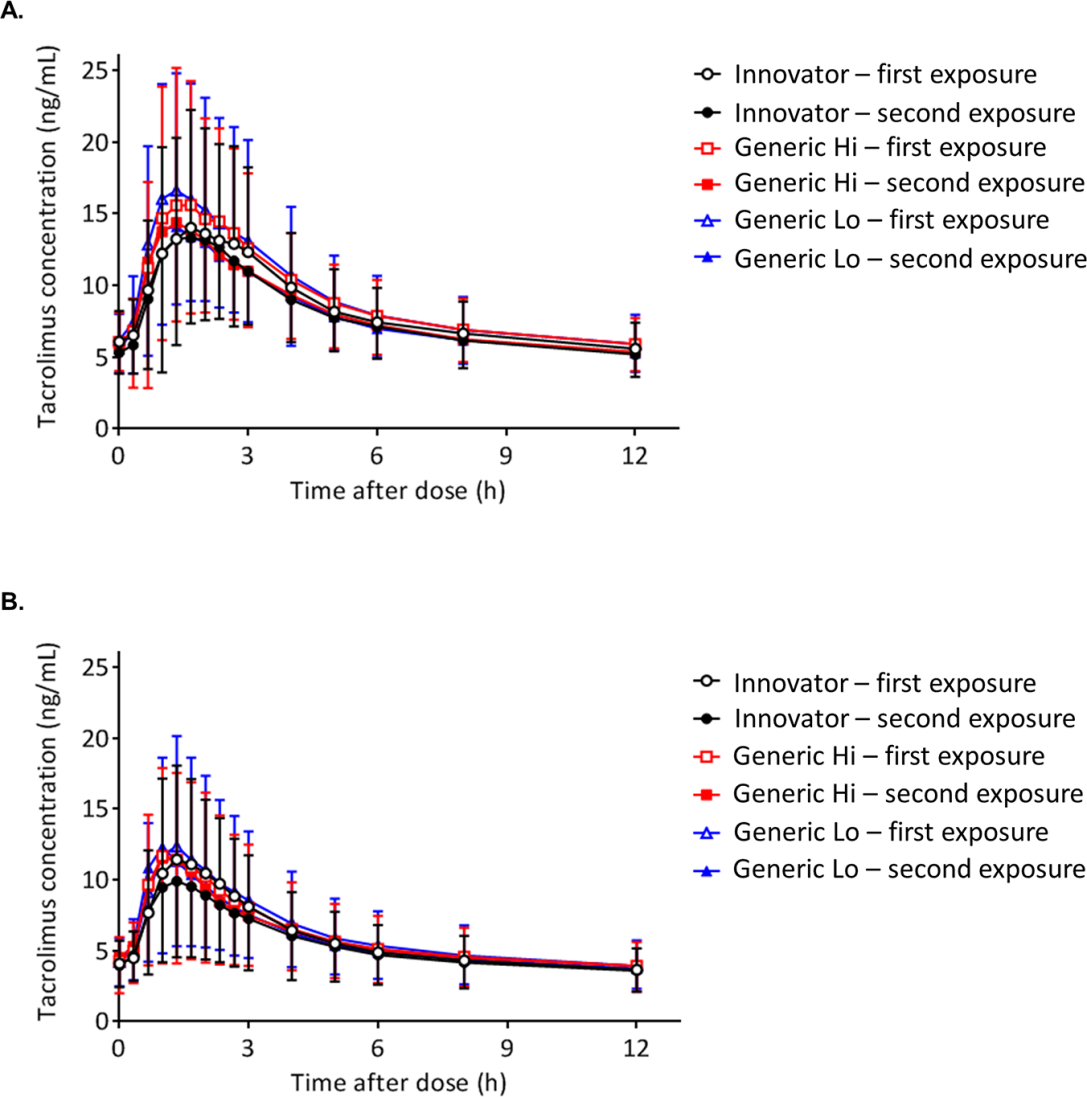
Comparison of tacrolimus time concentration curves by product. (A) Individuals with a kidney transplant. (B) Individuals with a liver transplant. Fifteen tacrolimus blood samples were collected at C_0_ (before the morning dose) and at 20, 40, 60 (1 hour), 80, 100, 120 (2 hours), 140, 160, and 180 (3 hours) minutes and 4, 5, 6, 8, and 12 hours to construct the tacrolimus concentration-time curves. Each tacrolimus product—Innovator, Generic Hi, and Generic Lo—was administered according to the randomly assigned treatment sequence (Fig 1) twice for 1 week before collection of steady-state pharmacokinetic profiles. Each point on the curve displays the tacrolimus mean whole blood concentrations; standard deviations are depicted by error bars. There were no statistically significant differences between any time points.

Tacrolimus 12-hour concentrations following chronic dosing are presented for individuals with a kidney (Fig 3A) or liver transplant (Fig 3B). Two tacrolimus concentration-time curves for each product are depicted, representing the first and second exposures to the product. Values for the mean and the standard deviation (SD) for each pharmacokinetic time point are reported in Table 2. There were no statistically significant differences observed by product between time points as assessed by Kruskal-Wallis [38].

**Table 2:** Tacrolimus level results at each time point for individuals with a kidney or liver transplant.

Individuals with a kidney transplant													
PK time point (min/h)	Innovator				Generic Hi				Generic Lo				*p*-Value
	First exposure		Second exposure		First exposure		Second exposure		First exposure		Second exposure		
	Mean ng/ml	SD	Mean ng/ml	SD	Mean ng/ml	SD	Mean ng/ml	SD	Mean ng/ml	SD	Mean ng/ml	SD	
0	6.1	2.2	5.3	1.5	6.1	2.0	5.7	1.7	6.2	1.8	5.6	1.7	0.36
20 min	6.5	2.5	5.8	2.0	6.8	2.3	6.9	4.0	7.4	3.3	6.8	3.0	0.25
40 min	9.7	4.9	9.0	4.9	11.2	6.0	11.6	8.8	12.9	6.8	11.5	6.4	0.06
1 h	12.2	7.4	12.3	8.4	14.7	9.2	13.7	7.6	16.0	8.0	13.9	6.7	0.11
80 min	13.2	7.1	13.3	7.4	15.6	9.6	14.4	6.9	16.6	8.2	14.1	5.4	0.33
100 min	14.0	8.2	13.3	5.9	15.6	8.7	13.9	5.8	16.0	8.1	13.4	4.5	0.72
2 h	13.6	7.4	13.2	5.6	14.6	7.0	13.1	5.0	15.2	7.9	13.0	4.1	0.86
140 min	13.1	6.7	12.7	5.0	14.5	6.5	12.1	4.5	14.3	7.3	12.0	3.6	0.76
160 min	12.9	6.8	11.7	4.6	13.6	5.9	11.4	3.8	13.7	7.4	11.6	3.5	0.72
3 h	12.3	5.9	11.0	3.7	12.6	5.2	11.0	3.9	13.1	7.0	11.0	3.6	0.79
4 h	9.9	3.8	9.0	3.0	10.4	3.3	9.3	3.1	10.7	4.8	9.3	3.5	0.37
5 h	8.2	3.0	7.7	2.3	8.8	2.7	8.0	2.4	8.9	3.2	7.8	2.3	0.44
6 h	7.4	2.4	7.1	2.3	7.9	2.5	7.2	2.1	7.9	2.8	7.0	2.0	0.54
8 h	6.6	2.2	6.2	1.9	6.9	2.2	6.2	1.6	6.9	2.3	6.2	1.7	0.50
12 h	5.6	1.8	5.2	1.6	5.9	1.8	5.3	1.3	5.9	2.1	5.3	1.3	0.51
**Individuals with a liver transplant**													
0	4.1	1.6	3.9	1.5	4.3	1.7	4.5	2.5	4.2	1.7	4.2	1.7	0.90
20 min	4.5	1.9	4.7	1.8	5.0	2.0	5.3	2.6	5.0	2.2	5.4	2.5	0.57
40 min	7.7	4.4	7.8	4.5	9.7	4.9	9.5	5.6	9.1	4.9	10.9	6.6	0.18
1 h	10.5	6.7	9.5	5.3	11.7	6.2	11.5	7.4	11.8	6.9	12.3	7.5	0.53
80 min	11.4	6.6	9.9	5.4	11.4	6.1	11.4	7.3	12.4	7.8	11.3	6.0	0.85
100 min	11.1	6.0	9.5	5.0	10.9	6.0	10.6	6.2	11.4	7.2	10.4	5.1	0.91
2 h	10.5	5.2	8.9	4.5	10.2	5.9	9.4	5.2	10.7	6.7	9.5	4.3	0.80
140 min	9.7	4.6	8.2	4.1	9.3	5.3	8.4	4.4	9.7	5.9	8.7	3.7	0.68
160 min	8.8	4.0	7.6	3.8	8.5	4.7	7.8	3.9	9.1	5.4	8.0	3.4	0.72
3 h	8.1	3.6	7.3	3.6	8.0	4.4	7.4	3.5	8.5	4.9	7.5	3.1	0.81
4 h	6.4	2.7	6.1	3.1	6.5	3.3	6.4	2.8	6.9	3.7	6.2	2.4	0.89
5 h	5.5	2.2	5.3	2.5	5.6	2.7	5.5	2.5	5.9	2.8	5.4	2.0	0.95
6 h	4.9	1.9	4.7	2.1	5.1	2.3	5.1	2.4	5.4	2.4	4.9	1.9	0.88
8 h	4.3	1.7	4.2	1.8	4.6	2.1	4.5	2.0	4.7	2.1	4.4	1.7	0.92
12 h	3.7	1.5	3.6	1.5	3.9	1.7	3.9	1.8	3.9	1.8	3.7	1.4	0.96

PK, pharmacokinetics; SD, standard deviation.

Pharmacokinetic parameters for individuals with a kidney or liver transplant by product are summarized in Table 3. Point estimates of the geometric means with the resulting 2-sided 95% confidence intervals are presented for AUC, C_max_, C_min_, and T_max_ by product. Product selection was based upon pharmacokinetic studies in healthy individuals as previously described. The Generic Hi (Sandoz) observed exposure was numerically higher than that of Innovator in both individuals with a kidney transplant and those with a liver transplant. In this study, the point estimate for Generic Lo exposure was numerically higher than that of both Innovator and Generic Hi in both individuals with a kidney transplant and those with a liver transplant. However, this can be expected to be caused by random variability since these products were shown to be bioequivalent. (Table 3)

**Table 3:** Comparison of key pharmacokinetic parameters after administration of the different tacrolimus products.

**Individuals with a kidney transplant**				
**PK metrics**	**Point estimate geometric mean (95% confidence interval)**			
	**Innovator**	**Generic Hi**		**Generic Lo**
**AUC**^**1**^ (ng*h/ml)	92.86 (83.41–103.38)	97.93 (88.33–108.58)		98.38 (88.57–109.28)
**C**_**MAX**_^**2**^ **(ng/ml)**	14.47 (12.57–16.65)	15.72 (13.67–18.08)		15.88 (13.79–18.30)
**C**_**MIN**_^**2**^ **(ng/ml)**	4.89 (4.42–5.41)	5.13 (4.67–5.63)		5.11 (4.65–5.61)
**T**_**MAX**_^**3**^ **(h)**	1.70 (1.50–1.94)	1.47 (1.27–1.70)		1.48 (1.28–1.72)
**Individuals with a liver transplant**				
**PK metrics**	**Point estimate geometric mean (95% confidence interval)**			
	**Innovator**	**Generic Hi**		**Generic Lo**
**AUC**^**1**^ (ng*h/ml)	62.26 (53.87–71.96)	65.26 (56.02–76.01)		66.87 (57.70–77.49)
**C**_**MAX**_^**2**^ **(ng/ml)**	10.49 (8.92–12.34)	11.16 (9.38–13.29)		11.73 (9.85–13.97)
**C**_**MIN**_^**2**^ **(ng/ml)**	3.26 (2.83–3.76)	3.49 (3.00–4.05)		3.42 (2.96–3.94)
**T**_**MAX**_^**3**^ **(h)**	1.35 (1.19–1.52)	1.16 (1.00–1.34)		1.19 (1.05–1.35)

AUC, area under the curve; PK, pharmacokinetics.

^1^AUC is quantified by the units of tacrolimus concentration (ng/ml) over time (h).

^2^The C_MAX_ and the C_MIN_ tacrolimus concentrations are reported in units of ng/ml and represent the highest and lowest observed concentration, respectively.

^3^T_max _is the time the maximum concentration is observed and is reported in hours (h).

The primary end point of bioequivalence of these pharmacokinetic parameters was tested using SCABE on log-transformed data. When comparing Innovator with Generic Hi and Generic Lo, and Generic Hi with Generic Lo, AUC, C_max_, and C_min_ fell within conventional ABE limits of 80%–125%, as well as within the tighter SCABE acceptance limits. Comparing interindividual variability of systemic tacrolimus exposure (AUC), within-subject variability ranged from 12.11% to 15.81%. Similarly, C_max_ within-subject variability ranged from 17.96% to 24.72% for all products. Per the FDA guidance for SCABE testing, all products exhibited similar pharmacokinetic variability since the upper limit of the 90% confidence interval for the ratio of within-subject variability was equal to or less than 2.5 (Tables 4 and 5). SCABE criteria were met for all product comparisons for AUC and C_max_ in both individuals with a kidney and those with a liver transplant. In reference to the EMA bioequivalence acceptance range for AUC of 90.00%–111.11%, these limits were met, with the only exception being in the case of Innovator versus Generic Lo. Here the upper limits of the 90% -confidence intervals—111.30% (kidney) and 112.12% (liver)—were slightly above the upper EMA AUC acceptance criterion [14], whereas only Innovator versus Generic Lo in liver transplant recipients was also above the Health Canada AUC bioequivalence acceptance limits for critical dose drugs of 90%–112%[15].

**Table 4:** Results of bioequivalence testing using average bioequivalence (ABE) and scaled average bioequivalence (SCABE) metrics for the area under the curve (AUC) (A), maximum concentration (C_max_) (B), and the minimum concentration (C_min_) (C) in individuals with a kidney transplant.

**(A) AUC**									
**Reference**	**Test**	**AUC geometric mean ratio (%)**	**Reference within-subject variability (S**_**WR,**_ **%)**	**Observed 90% confidence interval**	**Confidence bound**	**SCABE limit**	**Variability comparison (^1^statistics of σ_WT_/σ_WR_)**	**Product approval standards**	
								**FDA**^**2**^	**EMA**^**3**^
Innovator	Generic Hi	105.47	12.11	101.44–109.65	−0.0060	88.02–113.61	1.62	Pass	Pass
Innovator	Generic Lo	105.95	12.11	100.85–111.30	−0.0035	88.02–113.61	1.75	Pass	Fail
Generic Hi	Generic Lo	100.46	14.62	95.45–105.72	−0.0163	85.72–116.65	1.45	Pass	Pass
Generic Lo	Generic Hi	99.54	15.81	94.59–104.76	−0.0192	84.65–118.13	1.24	Pass	Pass
**(B) C**_**MAX**_									
**Reference**	**Test**	**C**_**MAX**_ **geometric mean ratio (%)**	**Reference within-subject variability (S**_**WR,**_ **%)**	**Observed 90% confidence interval**	**Confidence bound**	**SCABE limit**	**Variability comparison (^1^statistics of σ_WT_/σ_WR_)**	**Product approval standards**	
								**FDA**^**2**^	**EMA**^**3**^
Innovator	Generic Hi	108.68	22.74	102.77–114.94	−0.0293	80.00–125.00	1.46	Pass	Pass
Innovator	Generic Lo	109.80	22.74	101.06–119.29	−0.0207	80.00–125.00	1.12	Pass	Pass
Generic Hi	Generic Lo	101.02	24.72	93.69–108.93	−0.0469	80.00–125.00	1.03	Pass	Pass
Generic Lo	Generic Hi	98.99	18.99	91.81–106.73	−0.0266	81.86–122.16	1.75	Pass	Pass
**(C) C**_**MIN**_									
**Reference**	**Test**	**C**_**MIN**_ **geometric mean ratio (%)**	**Reference within-subject variability (S**_**WR,**_ **%)**	**Observed 90%confidence interval**	**Confidence bound**	**SCABE limit**	**Variability comparison (^1^statistics of σ_WT_/σ_WR_)**		
Innovator	Generic Hi	104.85	12.24	100.74–109.12	−0.0071	87.90–113.77	1.24		
Innovator	Generic Lo	104.48	12.24	99.68–109.51	−0.0067	87.90–113.77	1.66		
Generic Hi	Generic Lo	99.65	11.33	94.81–104.73	−0.0094	88.75–112.68	1.80		
Generic Lo	Generic Hi	100.36	15.14	95.49–105.48	−0.0177	85.25–117.30	1.01		

EMA, European Medicines Agency; FDA, US Food and Drug Administration; **σ**_WR_, within-subject standard deviation for the reference product; **σ**_WT_**,** within-subject standard deviation for the test product.

^1^Upper limit of the 90% confidence interval of the ratio of within-subject standard deviation of test product to reference product**,** **σ**_WT_**/σ**_WR_.

^2^FDA product approval standards for SCABE require the AUC and the C_MAX_ observed 90% confidence intervals to fall within the SCABE limit.

^3^EMA product approval standards for tacrolimus ABE require the observed 90% confidence interval to fall within 90.00%–111.10% for AUC and within 80.0%–125.0% for C_MAX_.

**Table 5:** Results of bioequivalence testing using average bioequivalence (ABE) and scaled average bioequivalence (SCABE) metrics for the area under the curve (AUC) (A), maximum concentration (C_max_) (B), and the minimum concentration (C_min_) (C) in individuals with a liver transplant.

**(A) AUC**									
**Reference**	**Test**	**AUC geometric mean ratio (%)**	**Reference within-subject variability (S**_**WR,**_ **%)**	**Observed 90% confidence interval**	**Confidence bound**	**SCABE limit**	**Variability comparison (^1^statistics of σ_WT_/σ_WR_)**	**Product approval standards**	
								FDA^**2**^	EMA^**3**^
Innovator	Generic Hi	104.81	14.26	101.28–108.47	−0.0124	86.05–116.22	1.34	Pass	Pass
Innovator	Generic Lo	107.40	14.26	102.88–112.12	−0.0071	86.05–116.22	1.19	Pass	Fail
Generic Hi	Generic Lo	102.47	14.26	97.93–107.23	−0.0142	86.05- 116.22	1.19	Pass	Pass
Generic Lo	Generic Hi	97.59	12.70	93.26–102.12	−0.0106	87.48–114.32	1.50	Pass	Pass
**(B) C**_**MAX**_									
**Reference**	**Test**	**C**_**MAX**_ **geometric mean ratio (%)**	**Reference within-subject variability (S**_**WR,**_ **%)**	**Observed 90% confidence interval**	**Confidence bound**	**SCABE limit**	**Variability comparison (^1^statistics of σ_WT_/σ_WR_)**	**Product approval standards**	
								**FDA**^**2**^	**EMA**^**3**^
Innovator	Generic Hi	106.39	17.96	101.96–111.02	−0.0192	82.76–120.83	1.83	Pass	Pass
Innovator	Generic Lo	111.84	17.96	105.98–118.03	−0.0050	82.76–120.83	1.69	Pass	Pass
Generic Hi	Generic Lo	105.12	24.61	98.50–112.19	−0.0424	80.00–125.00	1.23	Pass	Pass
Generic Lo	Generic Hi	95.13	22.67	89.14–101.52	−0.0348	80.00–125.00	1.45	Pass	Pass
**(C) C**_**MIN**_									
**Reference**	**Test**	**C**_**MIN**_ **geometric mean ratio (%)**	**Reference within-subject variability (S**_**WR,**_ **%)**	**Observed 90% confidence interval**	**Confidence bound**	**SCABE limit**	**Variability comparison (^1^statistics of σ_WT_/σ_WR_)**		
Innovator	Generic Hi	106.88	12.03	103.35–110.54	−0.0043	88.09–113.52	1.44		
Innovator	Generic Lo	104.64	12.03	100.89–108.54	−0.0074	88.09–113.52	1.09		
Generic Hi	Generic Lo	97.91	12.94	93.96–102.03	−0.0118	87.25–114.61	1.02		
Generic Lo	Generic Hi	102.14	9.84	98.02–106.43	−0.0057	90.15–110.93	1.76		

EMA, European Medicines Agency; FDA, US Food and Drug Administration; **σ**_WR_, within-subject standard deviation for the reference product; **σ**_WT_, within-subject standard deviation for the test product.

^1^Upper limit of the 90% confidence interval of the ratio of within-subject standard deviation of test product to reference product**,** **σ**_WT_**/σ**_WR_.

^2^FDA product approval standards for SCABE require the AUC and the C_MAX_ observed 90% confidence intervals to fall within the SCABE limit.

^3^EMA product approval standards for tacrolimus ABE require the observed 90% confidence interval to fall within 90.00%–111.10% for AUC and within 80.0%–125.0% for C_MAX._

Individual pharmacokinetic tacrolimus time-concentration curves for each pharmacokinetic period by product are presented (Figs D and E in S2 Appendix) [39]. Upon visual inspection of individual pharmacokinetic curves, differences can be observed upon comparison between products and between replicate administration of the same product.

### Subgroup analysis

The study was not powered to show a difference by any subgroup analyzed. A majority of individuals with a kidney transplant were genotyped as nonexpressers with CYP3A5 *3/*3 (*n* = 23), and 12 were expressers carrying *1/*3 (*n* = 10), and *1/*1(*n* = 2) variants (Figs F and G in S2 Appendix). Most individuals with a liver transplant were genotyped as nonexpressers (*n* = 30); however, 6 expressed the *1/*3 variant (Figs H and I in S2 Appendix). Donor samples were available for 17 individuals with a kidney transplant and for 24 with a liver transplant. Most kidney donors were genotyped as nonexpressers (*n* = 12); however, 5 were expressers with *1/*3 (*n* = 4) and *1/*1 (*n* = 1) variants (Figs J and K in S2 Appendix). Most liver individual donors were genotyped as CYP3A5 nonexpressers (*n* = 14); however, 10 were expressers with *1/*3 (*n* = 5) and *1/*1 (*n* = 5) variants (Figs L and M in S2 Appendix).

Individuals with a kidney transplant expressing CYP3A5 required significantly higher tacrolimus doses to achieve target tacrolimus trough blood levels (8.17 ± 2.5 versus 4.26 ± 1.9 mg/day [*p* = 0.0002]). In individuals with a liver transplant, recipient CYP3A5 genotype had no effect on the tacrolimus doses required to achieve target trough blood levels (4.67 ± 2.2 versus 4.5 ± 1.2 mg/day [*p* = 0.80]). When assessing the donor variant status, donor CYP3A5 expression had no impact on tacrolimus dose requirements for either individuals with a kidney transplant (6.25 ± 2.1 versus 5.40 ± 4.2 mg/day [*p* = 0.69]) or those with a liver transplant (4.71 ± 1.9 versus 5.70 ± 2.4 mg/day [*p* = 0.29]). No differences in within-subject variability were observed by CYP3A5 genotype in individuals with a kidney or liver transplant. No association between AUC and ABCB1 genotype as well as no impact on dosing requirements for individuals with a kidney (ANOVA *p* = 0.08) or liver transplant (ANOVA *p* = 0.35) was found (Figs N and O in S2 Appendix for AUCs).

ABE and SCABE limits for AUC were calculated for subgroups to assess for consistency of results (Figs PA–PC in S2 Appendix [kidney] and Figs QA–QC in S2 Appendix [liver]). In general, all FDA ABE limits were met, with most also meeting the stricter SCABE limits; however, most exceeded the EMA upper limit of 111.11%. For subgroups not meeting the ABE or SCABE criteria, the number of observations was 10 or fewer, resulting in low statistical power to conclude ABE or SCABE, except for POR*28. ABE was concluded for both POR*28 carriers and noncarriers for individuals with a kidney or liver transplant. For POR*28 carriers, SCABE was demonstrated except in the case of Generic Lo versus Innovator in individuals with a kidney transplant (*n* = 17) and Generic Hi versus Generic Lo in individuals with a liver transplant (*n* = 17). For POR*28 noncarriers, SCABE was demonstrated except for Generic Hi versus Innovator in individuals with a kidney transplant (*n* = 18) and individuals with a liver transplant (*n* = 19) and for Generic Lo versus Innovator in individuals with a liver transplant (*n* = 19). ABE criteria were met for most subgroups, except for individuals with a kidney transplant and a CYP3A4*1B genotype and individuals with a liver transplant and a CYP3A5 *1/*1 or *1/*3 genotype (Figs PA-C in S2 Appendix [kidney], Figs QA–C in S2 Appendix [liver]).

### Tacrolimus metabolite exposure

The blood concentrations of the major tacrolimus metabolite, 13-O-desmethyl tacrolimus, were also found to meet FDA ABE and SCABE AUC bioequivalence acceptance criteria in individuals with a kidney or liver transplant, but they failed EMA AUC acceptance limits (Tables T–U in S2 Appendix).

### Safety

One serious adverse event of pyelonephritis was reported in an individual with a kidney transplant, resulting in hospitalization prior to study drug administration. This event resolved with treatment, and the individual was withdrawn. Fifty-two percent and 65% of all individuals with a kidney or liver transplant, respectively, reported an adverse event. The adverse events are sorted by formulation and by individuals with a kidney or liver transplant and CTCAE disorder classification. The Exact McNemar test for paired categorical data with adjustments for multiple comparisons was used to compare adverse event rates among the products. No statistically significant differences among any pairs of products were found for any adverse event code or for adverse events overall (Table V in S2 Appendix).

No acute rejections occurred during the study period of 6 weeks. Baseline and weekly assessments included markers of transplant function in individuals with a kidney (creatinine) or liver (AST, ALT, and alkaline phosphatase) transplant. To compare kidney and liver function tests among products, a mixed effects model was run with a term designating the product received in each period and a random subject term and using the Tukey HSD test for multiple comparisons, where needed. No statistically significant differences were found among products (Fig R–W in S2 Appendix).

## Discussion

Public concerns remain regarding generic tacrolimus use in individuals with a kidney or liver transplant despite the significant market penetration of generic tacrolimus in the US. Historically, concerns were generated by a lack of definitive clinical evidence with properly controlled trials in target populations [13]. Limitations of previous studies [40–45] include retrospective evaluations, case reports, poor study design (underpowered or without appropriate controls), analysis of trough concentrations only, lack of analysis of confounders such as comedications and comorbidities, incorrect pharmacokinetic analysis, and use of nonspecific immunoassays in which metabolites may interfere with tacrolimus concentration measurements, thus leading to considerable bias and limited conclusions [13].

The present randomized, prospective, 3-treatment, 6-period, crossover, replicate-dosing study in stable individuals with a kidney or liver transplant systematically addresses the aforementioned public concerns regarding generic tacrolimus. The replicate dosing study design allowed the analysis of tacrolimus products using the tighter SCABE standards required by the FDA for NTI drugs. Clinically, the present study represents a scenario in which an individual with a kidney or liver transplant is randomly switched between 3 tacrolimus products every week for 6 weeks. The pharmacokinetic parameters (AUC and C_max_) demonstrated bioequivalence by SCABE criteria, implying similar tacrolimus exposure is achieved when individuals with a kidney or liver transplant are switched between these tacrolimus products. Although not required for bioequivalence testing, C_min_ also met the SCABE criteria.

These results support a previous prospective, multicenter, open-label, randomized, 2-period, crossover, pharmacokinetic study comparing twice-daily generic tacrolimus (Sandoz) versus reference tacrolimus (Prograf) in stable kidney transplant recipients [30]. In 68 kidney transplant recipients, there were no significant differences in AUC C_0_, C_max_, or T_max_ between the generic and reference products, resulting in ratios of the geometric mean and 90% CI for AUC and C_max_ that were reported as 102% (97%–108%) and 109% (101%–118%), respectively [46]. Post hoc analysis revealed the products also met SCABE acceptance criteria [47]. In contrast to the present study, the latter did not include comparisons to other generic tacrolimus products or the comparison of 2 generics, and liver transplant recipients and relevant genetic polymorphisms were not analyzed.

Pharmacogenomic profiling of individuals was performed, specifically, genotyping of CYP3A5 polymorphisms to identify the “poor absorber” [20,23]. The genetic polymorphisms most important for tacrolimus pharmacokinetics were assessed and, in general, had no effect on bioequivalence. The only exceptions were that bioequivalence of tacrolimus AUC was not found for individuals with a kidney transplant expressing CYP3A4*1B and individuals with a liver transplant expressing CYP3A5, but this analysis was underpowered for said subgroups. Moreover, an influence of POR*28 polymorphism on bioequivalence using SCABE metrics could not be excluded, although ABE criteria were met. Dosing differences by genotype were similar across products [48]. The major tacrolimus metabolite concentrations were bioequivalent for AUC. In contrast to tacrolimus, 13-O-desmethy tacrolimus is not directly administered but formed from tacrolimus, mostly by intestinal and liver cytochrome P4503A enzymes. Thus, its formation is greatly influenced by the aforementioned polymorphisms and its pharmacokinetics more variable than that of tacrolimus.

In this study, the 2 tacrolimus generic products met US FDA SCABE criteria when compared to the innovator product and with each other in individuals after a kidney or liver transplant. However, when applying the more rigid EMA criteria, the Generic Lo product failed AUC testing when compared to Innovator in individuals receiving a kidney or liver transplant. The EMA requires a narrow 90% confidence interval contained within the confidence limits of 90.00%–111.11% acceptance criterion for AUC, but not for C_max_, for which the usual 80.00%–125.00% acceptance limit applies [14]. As shown in Table 4, in general these acceptance criteria were met, with the only exception being that of Innovator versus Generic Lo, for which the upper limits of the 90% confidence intervals—111.30% (kidney) and 112.11% (liver)—were slightly above the upper EMA acceptance criterion for AUC. The conflicting approval guidelines lead to different interpretations of the bioequivalence data of the same study. In this context, it should be considered that the EMA and Health Canada bioequivalence limits for NTI drugs were set with single-dose healthy volunteer studies in mind, a population that is inherently less variable than individuals receiving a transplant after multiple doses [4]. While the FDA SCABE limits adjust based on the pharmacokinetic variability of the innovator in the study population, the EMA and Health Canada bioequivalence limits for NTI drugs are fixed, which explains the different conclusions when FDA, EMA, or Health Canada limits are employed. This study highlights the need for global harmonization of bioequivalence approval standards of NTI drugs to prevent different interpretations of bioequivalence study results.

Individual pharmacokinetic time concentration curves for all products are provided in Section C in S2 Appendix. These data visually depict the intraindividual variability that can be observed within the same product despite administering the same lot at the same dose in a controlled setting. Such observed intraindividual variability led the FDA to require repeat crossover study designs for NTI drugs, which requires that each individual receives each of the tested products twice to assess and to compare intraindividual variability between tacrolimus innovator and generic products. Upon visual inspection of individual pharmacokinetic curves, differences can be observed between products and between replicate administration of the same product.

Safety was similar across products over the observation period of 6 weeks. Although we did not evaluate the long-term impact of generic tacrolimus on acute rejection and graft survival, this study evaluates pharmacokinetic parameters as a surrogate for safety exposure.

Several design elements strengthen the findings, such as validated, specific, and sensitive high-performance liquid chromatography (HPLC)-tandem mass spectrometry analysis of tacrolimus and metabolites, quality control of the tacrolimus test batches, independent parallel analysis by the study team and the FDA, genotypic analysis of individuals donating or receiving a kidney or liver transplant, and close adherence monitoring using a combination of diaries, MEMS caps, and pill counts. Finally, this study was adequately powered to assess bioequivalence in both kidney and liver transplant recipients. However, our study also had certain limitations. A differential carryover or sequence effect cannot be fully excluded, even though we did not detect any statistically significant sequence effects in statistical modeling. When considering the half-life of immediate-release tacrolimus and the length of the treatment period, this effect is unlikely to occur. The pharmacokinetic profiling occurred in strictly controlled conditions with recipients who were highly adherent, which did not allow the evaluation about the impact of nonadherence or the impact of feeding on possible pharmacokinetic differences between products. The study design also did not allow for assessment of the potential impact of differing appearances of the 3 tacrolimus products on adherence. The safety data should be interpreted cautiously in the light of the small number of participants and short observation periods. Lastly, only the 1 mg tacrolimus dosage strength was utilized, limiting generalizability to the 0.5 mg and 5 mg dosage strengths; however, the 1 mg capsule is the most common clinically utilized dosage strength.

The present study was specifically designed to address lingering concerns in the transplant community [8–11]. While typical single-dose healthy volunteer bioequivalence studies mainly assess prescribability, our study in steady-state, stable individuals with a kidney or liver transplant mainly assessed the more important switchability between innovator and generics and between generics [5,6]. The present study suggests that tacrolimus and the tested generic products in healthy volunteers were also bioequivalent in individuals with a kidney or liver transplant. Moreover, the generics were bioequivalent to each other. Even the tighter FDA SCABE criteria were met, and there was no difference between the different tacrolimus products in terms of within-subject variability.

## Supporting information

S1 Consort Checklist Consolidated Standards Of Reporting Trials (CONSORT) checklist. (DOC)

S1 Text Trial protocol. (PDF)

S2 Text Statistical analysis plan. (DOCX)

S1 Appendix Supporting information methods. (DOCX)

S2 Appendix Supporting information results. (DOCX)

## Abbreviations

ABE: average bioequivalence
ALT: alanine aminotransferase
ANDA: Abbreviated New Drug Application
AST: aspartate aminotransferase
AUC: area under the curve
C_max_: maximum concentration
C_min_: minimum concentration
CONSORT: Consolidated Standards Of Reporting Trials
CTCAE: Common Terminology Criteria for Adverse Events
CYP: cytochrome
EMA: European Medicines Agency
FDA: United States Food and Drug Administration
HPLC: high-performance liquid chromatography
HSD: honest significant difference
MEMS: Medication Event Monitoring System
NTI: narrow therapeutic index
PK: pharmacokinetics
Reference: tacrolimus product that is used as reference product for analysis
SCABE: scaled average bioequivalence
T_max_: time-to-maximum concentration

## References

[1] Ensor CR, Trofe-Clark J, Gabardi S, McDevitt-Potter LM, Shullo MA. Generic maintenance immunosuppression in solid organ transplant recipients. Pharmacotherapy. 2011;31(11):1111–29. Epub 2011/10/27. doi: 10.1592/phco.31.11.1111 . 22026398 10.1592/phco.31.11.1111

[2] O’Neil J. How increased competition from generic drugs has affected prices and returns in the pharmaceutical industry. Congressional Budget Office 1988 [cited 2012 May 29]. Available from: http://www.fda.gov/ohrms/DOCKETS/dailys/04/June04/061404/03p-0029-bkg0001-Ref-15-vol3.pdf

[3] Sellares J, de Freitas DG, Mengel M, Reeve J, Einecke G, Sis B, et al Understanding the causes of kidney transplant failure: the dominant role of antibody-mediated rejection and nonadherence. Am J Transplant. 2012;12(2):388–99. Epub 2011/11/16. doi: 10.1111/j.1600-6143.2011.03840.x . 22081892 10.1111/j.1600-6143.2011.03840.x

[4] Davit BM, Nwakama PE, Buehler GJ, Conner DP, Haidar SH, Patel DT, et al Comparing generic and innovator drugs: a review of 12 years of bioequivalence data from the United States Food and Drug Administration. Ann Pharmacother. 2009;43(10):1583–97. Epub 2009/09/25. doi: 10.1345/aph.1M141 . 19776300 10.1345/aph.1M141

[5] Christians U, Klawitter J, Clavijo CF. Bioequivalence testing of immunosuppressants: concepts and misconceptions. Kidney Int Suppl. 2010;(115):S1–7. Epub 2010/02/13. doi: 10.1038/ki.2009.504 . 20150904 10.1038/ki.2009.504

[6] Hauck WW, Anderson S. Measuring switchability and prescribability: when is average bioequivalence sufficient? J Pharmacokinet Biopharm. 1994;22(6):551–64. Epub 1994/12/01. . 7473081 10.1007/BF02353794

[7] Pollard S, Nashan B, Johnston A, Hoyer P, Belitsky P, Keown P, et al A pharmacokinetic and clinical review of the potential clinical impact of using different formulations of cyclosporin A. Berlin, Germany, November 19, 2001. Clin Ther. 2003;25(6):1654–69. Epub 2003/07/16. . 12860490 10.1016/s0149-2918(03)80161-3

[8] Alloway RR, Isaacs R, Lake K, Hoyer P, First R, Helderman H, et al Report of the American Society of Transplantation conference on immunosuppressive drugs and the use of generic immunosuppressants. Am J Transplant. 2003;3(10):1211–5. Epub 2003/09/27. . 14510694 10.1046/j.1600-6143.2003.00212.x

[9] Harrison JJ, Schiff JR, Coursol CJ, Daley CJ, Dipchand AI, Heywood NM, et al Generic immunosuppression in solid organ transplantation: a Canadian perspective. Transplantation. 2012;93(7):657–65. Epub 2012/01/24. doi: 10.1097/TP.0b013e3182445e9d . 22267158 10.1097/TP.0b013e3182445e9d

[10] van Gelder T. European Society for Organ Transplantation Advisory Committee recommendations on generic substitution of immunosuppressive drugs. Transpl Int. 2011;24(12):1135–41. Epub 2011/10/29. 10.1111/j.1432-2277.2011.01378.x. 22032583. doi: 10.1111/j.1432-2277.2011.01378.x 22032583 10.1111/j.1432-2277.2011.01378.x

[11] Uber PA, Ross HJ, Zuckermann AO, Sweet SC, Corris PA, McNeil K, et al Generic drug immunosuppression in thoracic transplantation: an ISHLT educational advisory. J Heart Lung Transplant. 2009;28(7):655–60. Epub 2009/06/30. 10.1016/j.healun.2009.05.001. 19560691. doi: 10.1016/j.healun.2009.05.001 19560691 10.1016/j.healun.2009.05.001

[12] Klintmalm GB. Immunosuppression, generic drugs and the FDA. Am J Transplant. 2011;11(9):1765–6. Epub 2011/07/29. doi: 10.1111/j.1600-6143.2011.03616.x . 21794082 10.1111/j.1600-6143.2011.03616.x

[13] Molnar AO, Fergusson D, Tsampalieros AK, Bennett A, Fergusson N, Ramsay T, et al Generic immunosuppression in solid organ transplantation: systematic review and meta-analysis. BMJ. 2015;350:h3163 Epub 2015/06/24. doi: 10.1136/bmj.h3163 ; PubMed Central PMCID: PMCPMC4476317. 26101226 10.1136/bmj.h3163 PMC4476317

[14] European Medicines Agency. European Medicines Agency. Guideline on the Investigation of Bioequivalence London, UK2010 [cited 2016 October 26]. Available from: http://www.ema.europa.eu/docs/en_GB/document_library/Scientific_guideline/2010/01/WC500070039.pdf

[15] Health Canada. Guidance Document—Comparative Bioavailability Standards: Formulations Used for Systemic Effects Ottawa, Ontario: Health Canada; 2012 [cited 2016 October 26]. Available from: http://www.hc-sc.gc.ca/dhp-mps/alt_formats/pdf/prodpharma/applic-demande/guide-ld/bio/gd_standards_ld_normes-eng.pdf

[16] Food and Drug Administration. Draft Guidance on Tacrolimus 2014 [cited 2015 December 2]. Available from: http://www.fda.gov/downloads/Drugs/GuidanceComplianceRegulatoryInformation/Guidances/UCM406344.pdf

[17] Yu LX, Jiang W, Zhang X, Lionberger R, Makhlouf F, Schuirmann DJ, et al Novel bioequivalence approach for narrow therapeutic index drugs. Clin Pharmacol Ther. 2015;97(3):286–91. Epub 2015/02/12. doi: 10.1002/cpt.28 . 25669762 10.1002/cpt.28

[18] Birdwell KA, Decker B, Barbarino JM, Peterson JF, Stein CM, Sadee W, et al Clinical Pharmacogenetics Implementation Consortium (CPIC) Guidelines for CYP3A5 Genotype and Tacrolimus Dosing. Clin Pharmacol Ther. 2015;98(1):19–24. Epub 2015/03/25. doi: 10.1002/cpt.113 ; PubMed Central PMCID: PMCPMC4481158. 25801146 10.1002/cpt.113 PMC4481158

[19] Picard N, Bergan S, Marquet P, van Gelder T, Wallemacq P, Hesselink DA, et al Pharmacogenetic Biomarkers Predictive of the Pharmacokinetics and Pharmacodynamics of Immunosuppressive Drugs. Ther Drug Monit. 2016;38 Suppl 1:S57–69. Epub 2015/10/16. doi: 10.1097/ftd.0000000000000255 . 26469711 10.1097/FTD.0000000000000255

[20] Kuypers DR, de Jonge H, Naesens M, Lerut E, Verbeke K, Vanrenterghem Y. CYP3A5 and CYP3A4 but not MDR1 single-nucleotide polymorphisms determine long-term tacrolimus disposition and drug-related nephrotoxicity in renal recipients. Clin Pharmacol Ther. 2007;82(6):711–25. Epub 2007/05/15. doi: 10.1038/sj.clpt.6100216 . 17495880 10.1038/sj.clpt.6100216

[21] Pallet N, Jannot AS, El Bahri M, Etienne I, Buchler M, de Ligny BH, et al Kidney transplant recipients carrying the CYP3A4*22 allelic variant have reduced tacrolimus clearance and often reach supratherapeutic tacrolimus concentrations. Am J Transplant. 2015;15(3):800–5. Epub 2015/01/16. doi: 10.1111/ajt.13059 . 25588704 10.1111/ajt.13059

[22] Elens L, Hesselink DA, Bouamar R, Budde K, de Fijter JW, De Meyer M, et al Impact of POR*28 on the pharmacokinetics of tacrolimus and cyclosporine A in renal transplant patients. Ther Drug Monit. 2014;36(1):71–9. Epub 2013/09/26. doi: 10.1097/FTD.0b013e31829da6dd . 24061445 10.1097/FTD.0b013e31829da6dd

[23] Stefanovic NZ, Cvetkovic TP, Jevtovic-Stoimenov TM, Ignjatovic AM, Paunovic GJ, Velickovic RM. Investigation of CYP 3A5 and ABCB1 gene polymorphisms in the long-term following renal transplantation: Effects on tacrolimus exposure and kidney function. Exp Ther Med. 2015;10(3):1149–56. Epub 2015/12/02. doi: 10.3892/etm.2015.2598 ; PubMed Central PMCID: PMCPMC4533232. 26622455 10.3892/etm.2015.2598 PMC4533232

[24] Christians U, Jacobsen W, Benet LZ, Lampen A. Mechanisms of clinically relevant drug interactions associated with tacrolimus. Clin Pharmacokinet. 2002;41(11):813–51. Epub 2002/08/23. doi: 10.2165/00003088-200241110-00003 . 12190331 10.2165/00003088-200241110-00003

[25] Food and Drug Administration. Orange Book. Approved Drug Products with Therapeutic Equivalence Evaluations 2015 [cited 2015 December 1]. Available from: http://www.accessdata.fda.gov/scripts/cder/ob/docs/queryai.cfm

[26] Sandoz Tacrolimus Abbreviated New Drug Application Number A065461 [cited 2015 December 1]. Available from: https://www.accessdata.fda.gov/scripts/cder/daf/index.cfm?event=overview.process&ApplNo=065461

[27] Dr Reddy Tacrolimus Abbreviated New Drug Application Number A090509 [cited 2015 December 1]. Available from: https://www.accessdata.fda.gov/scripts/cder/daf/index.cfm?event=overview.process&ApplNo=090509

[28] Mylan Tacrolimus Abbreviated New Drug Application Number A090596 [cited 2015 December 1]. Available from: https://www.accessdata.fda.gov/scripts/cder/daf/index.cfm?event=overview.process&ApplNo=090596

[29] Accord Tacrolimus Abbreviated New Drug Application Number A091195 [cited 2015 December 1]. Available from: https://www.accessdata.fda.gov/scripts/cder/daf/index.cfm?event=overview.process&ApplNo=091195

[30] Panacea Tacrolimus Abbreviated New Drug Application Number A09082 [cited 2015 December 1]. Available from: https://www.accessdata.fda.gov/scripts/cder/daf/index.cfm?event=overview.process&ApplNo=090802

[31] Convention United SP. Tacrolimus Revision Bulletin, April 1, 2013 Rockville, MD2013 [cited 2015 December 1]. Available from: http://www.usp.org/sites/default/files/usp_pdf/EN/USPNF/revisions/tacrolimus_capsulesm.pdf

[32] Harris PA, Taylor R, Thielke R, Payne J, Gonzalez N, Conde JG. Research electronic data capture (REDCap)—a metadata-driven methodology and workflow process for providing translational research informatics support. Journal of biomedical informatics. 2009;42(2):377–81. Epub 2008/10/22. doi: 10.1016/j.jbi.2008.08.010 ; PubMed Central PMCID: PMCPMC2700030. 18929686 10.1016/j.jbi.2008.08.010 PMC2700030

[33] Diletti E, Hauschke D, Steinijans VW. Sample size determination for bioequivalence assessment by means of confidence intervals. Int J Clin Pharmacol Ther Toxicol. 1992;30 Suppl 1:S51–8. Epub 1992/01/01. . 1601532

[34] Administration FAD. Food And Drug Administration. Guidance for Industry—Statistical Approaches to Establishing Bioequivalence Rockville, MD2001 [cited 2012 June 7]. Available from: https://www.fda.gov/downloads/drugs/guidances/ucm070244.pdf

[35] Montgomery DC. Design and Analysis of Experiments 1st ed. Wiley 2013.

[36] National Cancer Instiute. Common Toxicty Criteria for Adverse Events, v 4.0 2010 [cited 2015 December 1]. Available from: http://ctep.cancer.gov/protocolDevelopment/electronic_applications/docs/ctcae_4_with_lay_terms.pdf

[37] Agresti A. Categorical Data Analysis (PDF). Hooken, New Jersey: John Wiley & Sons, Inc. 2002. ISBN 0-471-36093-7

[38] Daniel WW. Kruskal–Wallis one-way analysis of variance by ranks Applied Nonparametric Statistics. 2nd ed. Boston: PWS-Kent pp. 226–234. 1990. ISBN 0-534-91976-6

[39] Sauter R, Steinijans VW, Diletti E, Bohm A, Schulz HU. Presentation of results from bioequivalence studies. Int J Clin Pharmacol Ther Toxicol. 1992;30(7):233–56. Epub 1992/07/01. . 1506127

[40] Duong SQ, Lal AK, Joshi R, Feingold B, Venkataramanan R. Transition from brand to generic tacrolimus is associated with a decrease in trough blood concentration in pediatric heart transplant recipients. Pediatr Transplant. 2015;19(8):911–7. Epub 2015/10/27. doi: 10.1111/petr.12608 . 26497983 10.1111/petr.12608

[41] Robertsen I, Asberg A, Ingero AO, Vethe NT, Bremer S, Bergan S, et al Use of generic tacrolimus in elderly renal transplant recipients: precaution is needed. Transplantation. 2015;99(3):528–32. Epub 2014/08/26. doi: 10.1097/TP.0000000000000384 . 25148382 10.1097/TP.0000000000000384

[42] Min SI, Ha J, Kim YS, Ahn SH, Park T, Park DD, et al Therapeutic equivalence and pharmacokinetics of generic tacrolimus formulation in de novo kidney transplant patients. Nephrol Dial Transplant. 2013;28(12):3110–9. Epub 2013/10/03. doi: 10.1093/ndt/gft300 . 24084327 10.1093/ndt/gft300

[43] Spence MM, Nguyen LM, Hui RL, Chan J. Evaluation of clinical and safety outcomes associated with conversion from brand-name to generic tacrolimus in transplant recipients enrolled in an integrated health care system. Pharmacotherapy. 2012;32(11):981–7. Epub 2012/10/18. doi: 10.1002/phar.1130 . 23074134 10.1002/phar.1130

[44] Momper JD, Ridenour TA, Schonder KS, Shapiro R, Humar A, Venkataramanan R. The impact of conversion from prograf to generic tacrolimus in liver and kidney transplant recipients with stable graft function. Am J Transplant. 2011;11(9):1861–7. Epub 2011/07/01. doi: 10.1111/j.1600-6143.2011.03615.x . 21714845 10.1111/j.1600-6143.2011.03615.x

[45] McDevitt-Potter LM, Sadaka B, Tichy EM, Rogers CC, Gabardi S. A multicenter experience with generic tacrolimus conversion. Transplantation. 2011;92(6):653–7. Epub 2011/07/27. doi: 10.1097/TP.0b013e31822a79ad . 21788920 10.1097/TP.0b013e31822a79ad

[46] Alloway RR, Sadaka B, Trofe-Clark J, Wiland A, Bloom RD. A randomized pharmacokinetic study of generic tacrolimus versus reference tacrolimus in kidney transplant recipients. Am J Transplant. 2012;12(10):2825–31. Epub 2012/07/05. doi: 10.1111/j.1600-6143.2012.04174.x ; PubMed Central PMCID: PMCPMC3472020. 22759200 10.1111/j.1600-6143.2012.04174.x PMC3472020

[47] Bloom RD, Trofe-Clark J, Wiland A, Alloway RR. A randomized, crossover pharmacokinetic study comparing generic tacrolimus vs. the reference formulation in subpopulations of kidney transplant patients. Clin Transplant. 2013;27(6):E685–93. Epub 2013/10/15. doi: 10.1111/ctr.12256 . 24118450 10.1111/ctr.12256

[48] Macphee IA, Fredericks S, Tai T, Syrris P, Carter ND, Johnston A, et al Tacrolimus pharmacogenetics: polymorphisms associated with expression of cytochrome p4503A5 and P-glycoprotein correlate with dose requirement. Transplantation. 2002;74(11):1486–9. Epub 2002/12/20. doi: 10.1097/01.TP.0000045761.71385.9F . 12490779 10.1097/00007890-200212150-00002

