# Supplementary material for: Bioequivalence between innovator and generic tacrolimus in liver and kidney transplant recipients: A randomized, crossover clinical trial
Source: PLoS Med. 2017 Nov 14;14(11):e1002428. doi: 10.1371/journal.pmed.1002428 (PMC5685573; doi:10.1371/journal.pmed.1002428)
Supplement: S1 Text — (PDF) [file pmed.1002428.s002.pdf]

TITLE PAGE

Date: July 17, 2015

Title: Pharmacokinetic Studies of Tacrolimus in Transplant Patients

Principal Investigator: Rita Alloway, Pharm.D., FCCP
University of Cincinnati Medical Center
Department of Internal Medicine, Division of Nephrology
231 Albert Sabin Way ML 558
Cincinnati, Ohio 45267
Phone: 513-558-1568 / Fax: 513-558-4944 / Email Address: Rita.Alloway@uc.edu

Co-investigators: E. Steve Woodle, M.D., F.A.C.S
University of Cincinnati Medical Center
Department of Surgery/Division of Transplantation
231 Albert Sabin Way, ML 585
Cincinnati, OH 45219
Phone: 513-585-6001 / Fax: 513-558-8689/ Email Address: woodlees@uc.edu

Michael Cardi, M.D.
The Kidney and Hypertension Center
2123 Auburn Avenue, Suite 404
Cincinnati, OH 45219
Phone: 513-241-5630 / Fax: 513-241-7146/ Email Address: mike.cardi@gmail.com

Kenneth Sherman, M.D.
University of Cincinnati Medical Center
Division of Digestive Diseases
231 Albert Sabin Way, ML 0595
Cincinnati, OH 45267
Phone: 513-558-3918 / Fax: 513-558-1744/ Email Address: Kenneth.Sherman@uc.edu

Tiffany Kaiser, Pharm.D, BCPS
University of Cincinnati Medical Center
Department of Internal Medicine, Division of Digestive Disease
231 Albert Sabin Way, ML 0595
Cincinnati, OH 45267
Phone: 513-558-0821/ Email Address: kaisertn@uc.edu

Adele Rike-Shields, Pharm.D.
University of Cincinnati Medical Center
Department of Surgery/Division of Transplantation
231 Albert Sabin Way, ML 585
Cincinnati, OH 45219
Phone: 513-585-2145 / Fax: 513-558-3580/ Email Address: rikea@uc.edu

Uwe Christians, MD, PhD
University of Colorado
School of Medicine
Bioscience, East, Suite 100
1999 North Fitzsimons Parkway
Aurora, CO 80045-7503

Phone: 303-724-5670 / Fax: 303-724-5662/
Email Address: Uwe.Christians@ucdenver.edu

Alexander Vinks, PharmD, PhD, FCP
Cincinnati Children's Hospital Medical Center
Division of Clinical Pharmacology
3333 Burnet Avenue MLC 6018
Cincinnati, OH 45229-3039
Phone: 513-636-5833/ Fax: 513-636-4402/ Email Address: Sander.Vinks@cchmc.org

Consultants:

Randy Yeates
The University of Iowa Pharmaceuticals
115 South Grand Avenue
Iowa City, IA 52242
Phone: 319-335-8674/ Fax: 319-335-9418/ Email Address: randhall-yeates@uiowa.edu

Laszlo Endrenyi, PhD
University of Toronto
Department of Pharmacology and Toxicology
Phone: 416-978-8620/ Fax: 416-978-6395/ Email Address: l.endrenyi@utoronto.ca

Dennis Drotar, Ph.D.
Cincinnati Children's Hospital Medical Center
Division of Behavioral Medicine and Psychology
3333 Burnet Avenue ML 7039
Cincinnati, OH 45229-3039
Phone: 513-636-3936/ Fax: 513-803-0415/ Email Address: Dennis.Drotar@cchmc.org

Study Coordinators:

Elizabeth Cole
University of Cincinnati Medical Center
231 Goodman Street
Cincinnati, OH 45219

Stefanie Young
University of Cincinnati Medical Center
231 Goodman Street
Cincinnati, OH 45219

Rachel Craddock-Jones
The Christ Hospital
2123 Auburn Avenue
Cincinnati, OH 45219

Research Facilities:

University of Cincinnati Medical Center
234 Goodman Street
Cincinnati, OH 45219

The Christ Hospital
2123 Auburn Avenue
Cincinnati, OH 45219

IRB Oversight:

University of Cincinnati Institutional Review Board
University of Cincinnati Medical Center
University Hall, Suite 300
51 Goodman Drive
PO Box 210567
Cincinnati, OH 45221-0567
Phone: 513-558-5259

The Christ Hospital Institutional Review Board
The Christ Hospital
2139 Auburn Avenue, Room 3131, 3-North
Cincinnati, OH 45219
Phone: 513-585-2107

Research Involving Human Subjects Committee (RIHSC)

Table of Contents

1. Abstract 8
2. Background 8
3. Innovation 10
4. Objectives 11
5. Credentials of Investigators / Contractors 13
6. Background 14
6.1. Healthy volunteer studies with tacrolimus 16
6.2. Previous Studies, Experience and Preliminary Data Relevant to this Proposal 17
6.3. Once-Daily Modified Release Tacrolimus (FKMR) 18
6.4. LCP Tacrolimus (LCP –Tacro, once daily modified release tacrolimus) 19
6.5. Sandoz generic tacrolimus 21
6.6. Dr. Alloway's representation for the AST on Generic and Clinical Trial Organization. 21
6.7. Co-Investigators 21
7. Introduction 21
7.1. Justification and Feasibility 22
8. Product Information 24
8.1. Selection of the Final Products and Lot 27
9. Statistical Analysis 28
9.1. Expected Outcomes 28
10. Rationale: AIM 2 Blinding of the study results during analysis 30
11. Expected Outcomes, Potential Problems and Alternative Strategies 31
12. Rationale and Review of the Relevant Literature 32
13. Research Design 35
14. Study objectives 35
14.1. Primary objectives 35
14.2. Secondary objectives 36
14.3. Exploratory objectives 36
15. Investigational Plan 36
15.1. Logistic overview 36
15.2. Study design 37
15.3. Collection of pharmacokinetic profiles 37
15.4. Sample handling and storage 37

16. Population 37
16.1. Inclusion criteria 38
16.2. Exclusion criteria 38
17. Treatment 39
17.1. Investigational and control treatment 39
17.2. Randomization to treatment arms 39
17.3. Treatment blinding and unblinding 40
17.4. Subject numbering 40
17.5. Supply, storage, dispensing and tracking of study medication 40
17.6. Instructions for prescribing and taking study treatment 40
17.7. Permitted dose adjustments and interruptions of study treatment 41
17.8. Adherence monitoring 41
17.9. Rescue medication 41
17.10. Concomitant treatment 41
17.11. Prohibited treatment 41
17.12. Discontinuation of study treatment and premature subject withdrawal and replacement of drop-outs. 42
17.13. Study completion and post-study treatment 42
17.14. Early study termination 42
18. Visit schedule and assessments 43
18.1. Information to be collected on screening failures 46
18.1.1. Subject demographics/other baseline characteristics 46
18.1.2. Treatment exposure and compliance 46
18.1.3. Efficacy 46
18.1.4. Safety 46
18.1.5. Physical examination 46
18.1.6. Vital signs 46
18.1.7. Height and weight 46
18.2. Laboratory evaluations 47
18.2.1. Hematology 47
18.2.2. Clinical chemistry 47
18.2.3. Urinalysis 47
18.2.4. Tacrolimus Pharmacogenetic Testing 47
18.2.5. Pregnancy and assessments of fertility 47

18.2.6. Appropriateness of safety measurements 47
18.3. Other assessments 47
18.3.1. Blood samples for pharmacokinetic evaluation 47
18.4. Payment for Participation 48
19. Protection of Human Subjects 50
19.1. Potential Health Risks 51
19.2. Adverse Event Monitoring 51
19.2.1. Adverse events 51
19.2.2. Serious adverse event reporting 52
19.2.3. Pregnancies 52
19.2.4. Data and Safety Monitoring Committee 52
19.3. Adequacy of Protection against Risks 53
19.4. Potential Benefits of the Proposed Research to Subjects and Others 53
19.5. Importance of Knowledge to be Gained 53
19.6. Data Safety and Monitoring Plan 53
19.7. Inclusion of Women and Minorities 54
20. Tacrolimus pharmacogenetics 55
20.1. Study Sample Analysis Using a Validated Tacrolimus LC-MS/MS Assay for the Quantification of Tacrolimus. 56
21. Data review and database management 59
21.1. Site monitoring 59
21.2. Data collection 60
21.3. Database management, quality control and data base lock 60
22. Data analysis 60
22.1. Analysis sets 61
22.2. Subject demographics and other baseline characteristics 61
22.3. Treatments (study drug, rescue medication, other concomitant therapies, compliance) 61
22.4. Analysis of the primary variable(s) 61
22.4.1. Variable 61
22.4.2. Pharmacokinetics and Bioequivalence Metrics 62
22.4.3. Presentation of data 70
22.4.4. Handling of missing values/censoring/discontinuations 70
22.5. Analysis of secondary variables 70
22.5.1. Efficacy variables 71

22.5.2. Safety variables 71
23. Sample size calculation and statistical power 71
24. Reporting 72
25. Expected Outcomes, Problems and Alternative Strategies 72
25.1. Rationale 73
26. Population Pharmacokinetics/ Pharmacometrics 73
26.1. Covariate analysis 74
26.2. Stratification and Subpopulation Analysis 74
26.3. Evaluation of Tighter Bioequivalence Acceptance Limits 75
26.4. Individual Bioequivalence Metrics 76
26.5. Scaled Average Bioequivalence 76
27. Expected Outcomes, Problems and Alternatives Aim 4 77
REFERENCES 78

1. Abstract

After organ transplantation patients often require life-long treatment with immunosuppressive drugs to prevent allo-immune reactions against the transplant organ. Unfortunately, most immunosuppressants such as tacrolimus are considered critical dose/narrow therapeutic index drugs and require careful dose titration guided by therapeutic drug monitoring to ensure that blood concentrations within a narrow therapeutic target window are maintained. Too high exposure increases the risk of toxicity, over-immunosuppression and cancer; too low exposure may lead to rejection of the organ by the host immune system. It has also been shown that variable pharmacokinetics with frequent episodes of *too high* and *too low* exposure in subgroups of patients referred to as "poor absorbers" can be detrimental to transplant organ function and the patient. Thus, with advent of the first cyclosporine generics 15 years ago, transplant physicians and their patients have been concerned that the quality, pharmacokinetics, and therapeutic efficacy of generics may differ from the branded product. At a more practical level there is also concern that these generics are not freely interchangeable with the brand version and, even more importantly, amongst each other. Indeed, such concerns have been published repeatedly in numerous consensus documents, editorials and reviews. Unfortunately, these arguments and the resulting recommendations are often based on theoretical concerns, anecdotal data or statistically underpowered and/or poorly controlled, open label clinical trials. There is therefore a huge unmet clinical need for well-designed and adequately powered studies in this field. We propose, herein, to address the most important of such concerns in a prospective, appropriately powered, fully replicated, blinded, 6-way cross-over study in kidney and liver transplant patients that will test the following primary hypothesis:

Generic immunosuppressants for which bioequivalence was established in single-dose healthy volunteer studies are also bioequivalent to the brand in stable transplant patients under steady-state conditions and there will be no difference in intra-subject variability

We will also test the following secondary hypotheses: (a) generic immunosuppressants will be bioequivalent independent of the transplant type (kidney or liver) (b) both generic immunosuppressants will be bioequivalent even if the most disparate generic formulations currently approved in the United States as determined in *in vitro* dissolution studies are compared (c) alternative bioequivalence metrics such as narrower acceptance intervals, scaled average bioequivalence and a population pharmaco-kinetic approach will confirm bioequivalence between the brand and the two generics in the complete study population as well as in the above-mentioned subgroups.

We will address these hypotheses through the following specific aims: **Aim 1:** Identification of the most disparate tacrolimus generic drug formulations (Generic Hi and Generic Lo) among those currently approved in the United States. **Aim 2:** Blinding of study procedures. **Aim 3:** Comparison of the replicate relative bioavailability and steady-state pharmacokinetics of Brand, Generic Hi and Generic Lo in a prospective, appropriately powered, fully replicated, blinded, 6-way cross-over study including kidney (n=38) and liver (n=38) transplant patients. **Aim 4.** Subgroup analysis, individual bioequivalence, population pharmacokinetics, and scaled average bioequivalence.

2. Background

Most transplant patients require life-long immunosuppression. Hence, the switch to generic immunosuppressants potentially can lead to significant savings [1, 2]. However, most immunosuppressants including the CNI, tacrolimus, which is currently used in more than 80% of solid-organ transplant patients in the United States [3], are narrow therapeutic index drugs [4-10]. Dosing is adjusted based on therapeutic drug monitoring to ensure that blood concentrations are maintained in a relatively narrow therapeutic concentration window [11, 12]. Failure to achieve this goal increases the risk of potentially severe consequences. Too high exposure may lead to over-immunosuppression, toxicities such as cardiovascular, nephro-, and neurotoxicity as well as the development of diabetes, and cancer. Too low exposure may result in increased activity of chronic or acute allo-immune reactions leading to accelerated destruction of the transplant organ. High pharmacokinetic intra-individual variability results in frequent episodes of over- and under-immunosuppression

and is detrimental to both the transplant organ and patient survival and also significantly increases costs to the healthcare system [13, 14].

The current US FDA generic drug approval process has been in place for almost 30 years [15] and in general has performed very well [16-18]. Despite its longevity and track record, the bioequivalence approval process has been debated heavily by the transplant community ever since the CNI, cyclosporine, came off patent and the first cyclosporine generics were developed more than 15 years ago. The debate about whether the standard bioequivalence approval process based on two-way cross-over studies in healthy volunteers and comparison of the pharmacokinetics of the test/ reference products based on average bioequivalence metrics is a valid approach in transplant patients has been ongoing ever since. This debate was recently reinvigorated when generic formulations of tacrolimus were approved in the United States and in Europe. Major concerns as discussed in recent consensus documents, reviews and editorials include, but are not limited to, the following:

Transplant patients are much more complex than healthy volunteers with multiple morbidities and co-medications. Results from healthy volunteer studies, therefore, cannot be translated to transplant patients and bioequivalence in the target population should be mandatory [6,7,8]

The bioequivalence acceptance interval of 80-125% is too wide and a tighter window for narrow therapeutic index drugs should be required [6, 7, 8].

Bioequivalence is established based on single dose pharmacokinetics; however, what really counts in transplant patients is bioequivalence under steady-state conditions [7].

Current regulatory guidances require only the establishment of bioequivalence between a generic and the brand. However, patients will be switched from generic to generic. Bioequivalence and interchangeability (switchability) among generics is not sufficiently established [6, 7].

It cannot be excluded that generics will behave differently than the brand in case of drug-drug, food-drug and disease-drug interactions [6, 8].

Establishment of bioequivalence mainly relies on surrogate markers of exposure (AUC), extent and rate of absorption (C_{max} and t_{max}). However immunosuppressant dosing, such as in the case of tacrolimus, is adjusted based on trough blood concentrations (C_{min}). Therefore, bioequivalence of trough blood concentrations and elimination half-life also needs to be demonstrated. [7]

Generic and brand may be different in subpopulations such as patients who express cytochrome P4503A5 and are poor absorbers, in patients who have diseases that affect immunosuppressant pharmacokinetics such as diabetes and hepatitis C virus infections as well as in pediatric populations. These patients may also metabolize the drugs released from different formulations differently [6].

This discussion has led to considerable confusion among transplant physicians and patients, with far-reaching consequences such as the request that brand-generic and generic-generic switches need to be authorized by transplant physicians [7,8], increased frequency of therapeutic drug monitoring after the switch [7,8,20] potentially offsetting the financial benefits of generic drug use in transplant patients [6,19,20], all the way to carve-out legislature in several US states preventing or limiting the use of immunosuppressant generic drug substitution [21]. The aforementioned concerns have at least a theoretical scientific basis and in several cases are supported by peer-reviewed publications, albeit many of those are based on underpowered and/or poorly controlled, open label clinical trials or case reports [6, 22]. In addition, there have unfortunately been less qualified and blatantly incorrect concerns and discussions that have found their way even into reputable

nephrology and transplant journals, such as [20,23]. These issues have previously been described and addressed by us in detail [22, 24].

This proposal must be considered highly significant and of far-reaching direct clinical impact as we propose to address the most important of the aforementioned concerns in a prospective, appropriately powered, fully replicated, 3-way cross-over study in kidney and liver transplant patients.

It is reasonable to expect that the results of the proposed study will have a significant impact on the clinical acceptance of immunosuppressant generics, future requirements for immunosuppressant generic bioequivalence testing and approval as well as the requirement for post-approval risk evaluation and mitigation strategies (REMS). It is reasonable to expect that this will have a significant impact on health care costs after organ transplantation.

3. Innovation

(1) Study design. The study design of a prospective, appropriately powered, fully replicated, blinded, 3-sequence cross-over study to directly compare the steady-state pharmacokinetics of tacrolimus brand and two generics in stable kidney and liver transplant patients must already be considered innovative as such a study has not been carried out before.

(2) Steady-state pharmacokinetics. While bioequivalence studies are usually single dose studies, patients in the present study will be treated with the test drug formulation prior to pharmacokinetic evaluation for one week, which will be sufficient to reach steady-state.

(3) Comparison of Generic Hi and Generic Lo. We are not aware of any study that, although discussed in several review and consensus papers, has compared two immunosuppressant generic drug formulations with each other in transplant patients. To be able to extrapolate our results to all immunosuppressant generics approved in the United States, we propose herein to identify the most disparate immunosuppressant generics using *in vitro* dissolution studies that will test all immunosuppressant generics in the United States (1mg strength, 3 different production lots each) under exactly the same conditions. By comparing the two generics with the highest (Generic Hi) and lowest (Generic Lo) dissolution rates in the clinical trial, it seems reasonable to expect that all other generics will fall in between the pharmacokinetics of the two generics tested. To avoid potential patient and investigator bias, while eliminating the impact of blinding methods on the dissolution of the test product, we propose to blind each of the study sites. The PK study site will be blinded to sequence of study drug administration. The analytical site will be blinded to study sequence and study product. The pharmacokinetic analysis site will receive tacrolimus levels and analyze each PK period and finally link each PK period to the product administered by obtaining this information from the IDS pharmacy. Adherence monitoring using MEMS drug bottle caps. The goal of the proposed study is to compare the steady state pharmacokinetics of the Brand, Generic Hi and Generic Lo with each other. To achieve steady-state, it will be critical that the patients reliably take their medications prior to pharmacokinetic evaluation. MEMS drug bottle caps allow for the electronic monitoring of access to the drug bottles and is an innovative and effective technology for adherence monitoring.

(4) Pharmacogenomics/ pharmacogenetics evaluation of individuals enrolled into the study. As aforementioned, it has been suspected that so called “poor absorbers” may show differences between the pharmacokinetics after oral administration of brand and generics. “Poor absorbers” are also considered high-risk patients as they typically also have higher intra-individual variability of tacrolimus pharmacokinetics than other patients. On the other hand, it has been shown that the expression of cytochrome P4503A5 and certain p-glycoprotein (MDR-1/AbCB1) haplotypes are associated with the poor tacrolimus absorber phenotype. We will stratify patients based on genotype and assess bioequivalence between the Brand, Generic Hi and Generic Lo with each other.

(5) Bioequivalence of tacrolimus metabolite pharmacokinetics. There has been speculation that tacrolimus released from generic formulations may result in different blood metabolite patterns and pharmacokinetics. A potential reason that has been discussed is that excipients may interact with intestinal efflux transporters and drug metabolizing enzymes. Tacrolimus undergoes significant first pass metabolism in the intestine. It is well established that efflux transporters and drug metabolizing

enzymes acting in concert are involved. We have a validated LC-MS/MS assay to quantify all clinically relevant tacrolimus metabolites. To the best of our knowledge, our group is currently the only laboratory site which can analyze the complete set of tacrolimus metabolites and thus we are in the unique position of having all the necessary reference materials available.

(6) Population pharmacokinetic modeling. Recent consensus papers have requested that the terminal half-life of immunosuppressants has to be considered critical to establish bioequivalence in transplant patients. However, the authors did not address the fact that transplant patients are dosed every 12 hours, which is less than one half-life of tacrolimus. Thus, the terminal half-life cannot reliably be estimated. We will conduct population pharmacokinetic analyses that will allow us to compare the apparent clearance of the different tacrolimus formulation as well as other pharmacokinetic parameters and will allow for assessing the potential influence of co-variates. In addition we will also evaluate if comparison of pharmacokinetic parameters using scaled average bioequivalence will add value.

The proposed study and research strategy will systematically assess and challenge concerns that have been discussed in recent consensus papers, reviews and editorials. As none of the strategies described above have yet been used in bioequivalence testing of immunosuppressant drugs, each of these must be considered innovative. However, the integration of these aspects is even more unique, will provide a comprehensive picture of the field, will reveal important new information and will outline a roadmap on how to address similar questions in the future.

4. Objectives

It is the goal to test the following hypotheses:

Primary hypothesis:

Generic immunosuppressants for which bioequivalence were established in single-dose healthy volunteer studies are also bioequivalent to the brand in stable transplant patients under steady-state conditions and there will be no difference in intra-subject variability.

Secondary hypotheses:

Generic immunosuppressants will be bioequivalent independent of the transplant type (kidney or liver)

Both generic immunosuppressants will be bioequivalent to each other even if the most disparate generic formulations currently approved in the United States as determined in *in vitro* dissolution studies are compared.

The three formulations tested will be bioequivalent with each other even in patients who are known expressors of cytochrome P4503A5 and/or high activity of the efflux transporter p-glycoprotein (based on MDR-1 haplotype analysis), which are known to be "poor absorbers" of tacrolimus.

There will be no difference in the metabolism of tacrolimus among the three formulations in the study population as well as in the subgroups.

Alternative bioequivalence metrics such as using narrower acceptance intervals, scaled average bioequivalence and a population pharmacokinetic approach will confirm bioequivalence between the brand and the two generics among each other in the complete study population as well as in the above-mentioned subgroups.

To test these hypotheses, we propose the following four Aims (please see also the flowchart in Figure 1 below):

Aim 1: *Identification of the most disparate tacrolimus generic drug formulations among those currently approved in the United States.* We will conduct systematic dissolution testing of the brand and all currently

approved tacrolimus drug formulations using the FDA-recommended dissolution method [25, 26]. We propose to test and compare the 1mg capsule strength (3 production lots/ manufacturer). In addition, we will compare the different formulations in terms of potency, purity and other quality attributes. This work will be carried out in the GMP-compliant facilities of The University of Iowa Pharmaceuticals (uip.pharmacy.uiowa.edu) and the University of Colorado (iC42 Clinical Research and Development, Laboratory Director: U. Christians). Based on these studies the two most disparate generic tacrolimus formulations (Generic Hi and Generic Lo) will be selected and compared with the Brand (Prograf[R], Astellas, Deerfield, IL) in the clinical trial described in Aim 3.

<u>Aim 2:</u> *Blinding of the study procedures.* Analysis of the study will be blinded at the 3 major points, ie PK study site blinded to study product sequence, analytical site blinded to study product, PK analysis site blinded to study product until each PK period results are analyzed.

<u>Aim 3:</u> *Comparison of the replicate relative bioavailability and steady-state pharmacokinetics of Brand, Generic Hi and Generic Lo in a prospective, appropriately powered, fully replicated, blinded, 3-way cross-over study including kidney (n=38) and liver (n=38) transplant patients.* As we proposed to test bioequivalence in the steady-state, patients will receive the test formulations for one week prior to pharmacokinetic evaluation. Thus, it will be critical that the patients are adherent to their test medication to ensure that they have reached steady state. This will be monitored using test diaries, pill counts and MEMS caps (Medication Event Monitoring System (MEMS), AARDEX Corp, Palo Alto, CA). Bioequivalence will be tested using average bioequivalence metrics and analysis of variance as appropriate and intra-individual variability of the formulations will be compared. This will also include the analysis of potential period and sequence effects.

<u>Aim 4.</u> *Subgroup analysis, individual bioequivalence, population pharmacokinetics, and scaled average bioequivalence.* Aim 4 is an exploratory aim in which we will (A) address the concern that bioequivalence in the "general" patient population will not translate to special subgroups ("poor absorbers" and patients with diseases known to affect drug metabolizing enzymes and transporters such as diabetes) and (B) test alternative bioequivalence metrics that have been proposed for the analysis for immunosuppressant generics such as narrower acceptance intervals, individual and scaled average bioequivalence was well as population pharmacokinetics.

Figure 1. *Flowchart of the proposed research strategy and aims.*

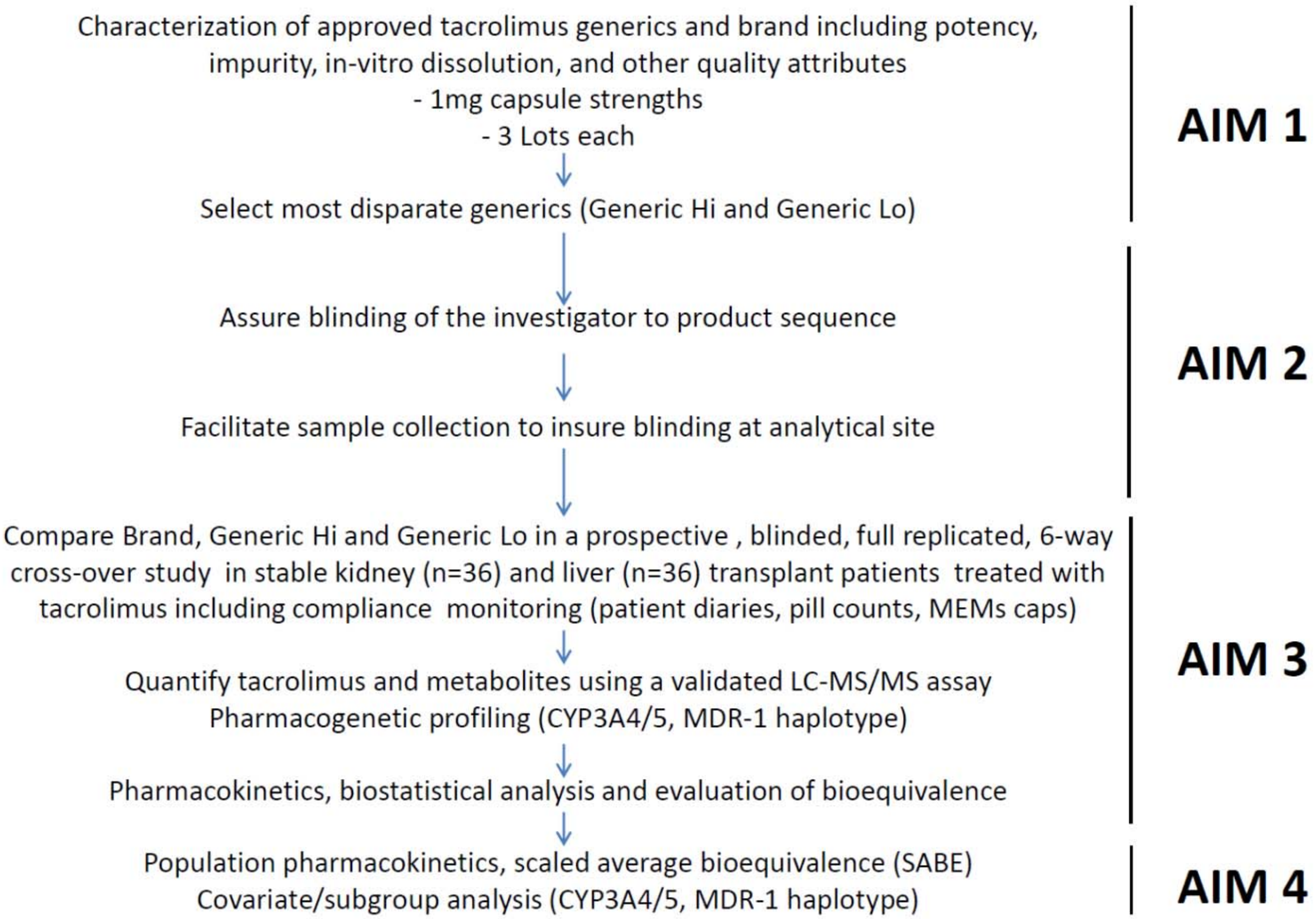

5. Credentials of Investigators / Contractors

The team assembled for this project includes Dr. Alloway from the University of Cincinnati Medical Center, Dr. Christians from the University of Colorado, and Dr. Vinks from Cincinnati Children's Medical Center in addition to expert consultants Mr. Yeates and Dr. Endrenyi from the University of Iowa and University of Toronto, respectively. This team combines expertise in generic clinical trial design (Drs. Alloway and Christians) and conduct with world class analytical and pharmacokinetic expertise (Drs. Vinks and Christians). We have identified an expert in in-vitro dissolution testing to facilitate product selection (Mr. Yeates, University of Iowa). The addition Dr. Drotar and his team who are leading experts in the assessment of treatment adherence by a variety of techniques will provide us with the ability to monitor patient adherence to assure quality study conduct. We consider it a special strength that we could bring together a team of leading experts in biostatistics, population pharmacokinetics/ pharmacometrics and bioequivalence testing who will devote considerable time and effort to the analysis of the data (Drs. Vinks, Endrenyi and Tran) The overall expertise of this team along with three transplant programs with a proven track record for subject enrollment and quality study conduct optimizes success of the proposal. The sample size, study timeline, and overall organization of the team that we have incorporated into the proposal are fully consistent with the RFA, and the University of Cincinnati transplant study team has the experience, expertise, and patient population required to accomplish the goals stated in the announcement. The clinical study team leaders include Drs. E. Steve Woodle, Michael Cardi, Kenneth Sherman, Adele Shields, and Tiffany Kaiser.

6. Background

Factors that fuel the debate over bioequivalence testing may be divided into 3 categories: 1) Scientific, 2) Business/Financial, and 3) Logistic. The present proposal will focus upon the scientific factors and attempt to answer if the current FDA approval process is appropriate for immunosuppressive agents by comparing various pharmacokinetic data analysis methods in the target patient population of kidney and liver transplant recipients and measure short term outcomes associated with formulation conversion. The replicate studies will be used to determine the intra-individual variabilities of the pharmacokinetic responses of the two generic and brand products, as well as subject-by-formulation interaction variances. The data will also be used to calculate simultaneously all parameters that are needed to compare the 3 products in average and individual bioequivalence and outlier analyses. The differences between the disparate generics will be used to establish if the current standards translate to equivalence within the limits of the brand intra-subject variation. The factors we will use to determine the most disparate generics include the results from the *in vivo* data from the ABE studies submitted to the FDA in the ANDA and *in vitro* chemical assay (potency) and dissolution data performed on several currently available lots. The intent is to study the specific lot of the generic product predicted to result in the lowest levels and compare it to the specific lot from another generic product predicted to result in the highest levels. All formulations will then be blinded to assure subject and clinician freedom from bias. Subjects will maintain a daily diary documenting the dose and time tacrolimus was taken, and drug accountability will be performed at each study visit. In addition, objective MEMS caps data will be reviewed for adherence.

Upon rigorous data collection and analysis of these scientific factors, it is expected concerns related to the business/financial impact upon the manufacturer of the reference or generic formulation, medical field specialty reliant on future drug development, and most importantly, the patient may be clearly, objectively, and scientifically addressed. In addition, any additional logistic factors such as increased therapeutic drug monitoring requirements may be addressed.

Calcineurin inhibitors (CNIs) have been the cornerstone of immunosuppression for kidney and liver transplantation since the introduction of cyclosporine [27]. Successful CNI treatment has long been associated with stable blood concentrations within a target therapeutic level range [28]. The intra and inter patient pharmacokinetic variability has been well established. Many sources of variability may be minimized (i.e. timing of daily doses and levels, dietary effects on drug absorption, drug interactions, type of CNI assay methodology, etc.), while other sources of pharmacokinetic variability are inherent and have no or limited possibility of intervention (i.e. gastric motility disorders, liver function, genetic polymorphisms, etc.). Clinicians have attempted to recognize and minimize factors impacting pharmacokinetic variability to improve transplant outcomes. Although the use of generic equivalents for prednisone and azathioprine are commonplace in transplantation, their effective use is not associated with therapeutic drug monitoring thus these agents are not historically categorized as narrow therapeutic index drugs. CNIs such as cyclosporine and tacrolimus, in addition to being the cornerstone of immunosuppression, are categorized as narrow therapeutic index drugs due to their steep dose response between efficacy and toxicity and requirement for therapeutic drug monitoring [5-8]. Until recently, international regulatory approval for generic products utilized similar bioequivalence testing criteria. Regulatory approval of generic products required only evidence of equivalent relative oral bioavailability versus the originator drug in healthy volunteers. Such studies are generally performed in small populations (12-36 subjects) using a single-dose, two-way cross-over design. With agents known to exhibit food effects upon pharmacokinetic parameters, fasted and fed studies in healthy volunteers are required. Pharmacokinetic parameters such as area under the curve (AUC) concentration measurements act as surrogate markers for the extent of absorption while peak concentration (C_{max}) and time to C_{max} (t_{max}) characterize the rate of absorption. The FDA requires that the 90% confidence interval (CI) of the ratio of the geometric means for the generic compared with the originator falls between 80% and 125% for both AUC and

C_{max} [25, 29]. The European Medicines Agency (EMA) stipulates a slightly narrower window for AUC (90-111%) [30]. Under the EMA guidelines, only AUC_{0-t} is always required while C_{max} assessment is only necessary when relevant (i.e. if it is of particular importance for safety, efficacy or drug level monitoring). Health Canada has adopted stricter acceptance margin standards for “critical dose drugs” of 90-112 for AUC. Tacrolimus has been identified as a “critical dose drug [6]. In 2011 and 2012, the FDA convened a series of Advisory Panels that suggested narrow therapeutic index drugs were a distinct group of products and current bioequivalence standards were not sufficient. Definitions for narrow therapeutic index drugs were proposed along with study design and analysis changes for bioequivalence testing of narrow therapeutic drugs in healthy volunteers. To date, these suggestions have not translated to changes in the generic drug approval process. In addition it is not obvious how new regulatory standards, if adopted, would impact narrow therapeutic index drugs with generic formulations already FDA approved and marketed.

With the introduction of the first CNI generic equivalent (cyclosporine) to transplantation in 1998, clinicians have debated whether generic alternatives introduce a significant source of variability that is avoidable, and therefore results in an unnecessary risk to transplant recipients overall graft survival [22]. Despite this over 50% of modified cyclosporine was dispensed as a generic formulation (2009) and approximately 60% of tacrolimus was dispensed as a generic formulation (2011). The percentage of branded formulation market share tends to remain relatively stable, while market shares of each generic formulation varies. Since expiry of the tacrolimus patent in 2008, generic preparations have become available and have been widely adopted. Figure 2 shows tacrolimus market share from August 2010 to January 2011. As of October 2011, the market shares of Prograf[R] were 40%, Sandoz 25%, Dr. Reddy 19%, and Mylan 15%. (IMS Dataview, 2011) This suggests extensive interchanging between formulations hypothetically due to formulary changes or financial influences.

Figure 2. *Market share of tacrolimus formulations in the United States.*

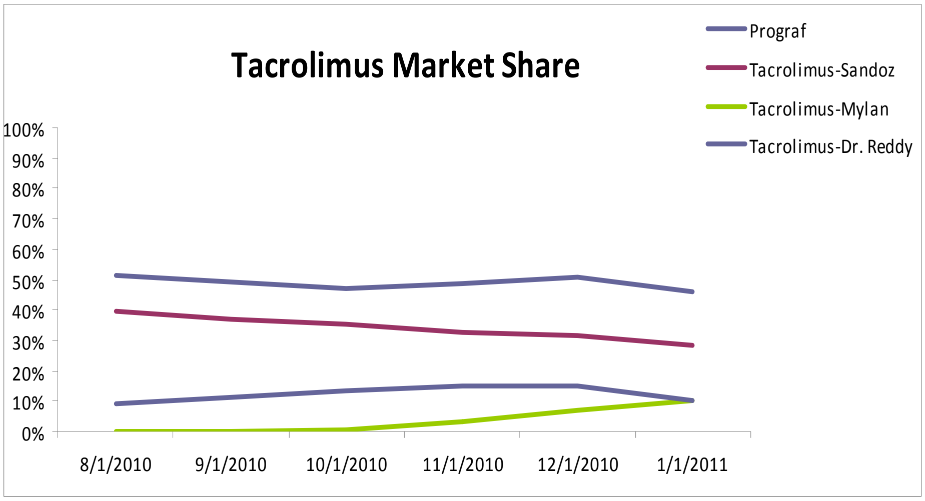

Tacrolimus pharmacokinetics are relatively complex with a high degree of inter- patient variability such that therapeutic drug monitoring is mandatory [11, 12, 31-34]. Differences between patients can be affected by a multitude of factors, including patient demographics, liver function, diurnal variation, concomitant immunosuppressants, gastrointestinal disturbances, co-existing diabetes mellitus and genetic differences in CYP3A and P-glycoprotein expression [35]. In transplant patients, the key contributors to intra-patient variability in immunosuppressant dosing are usually drug–drug, drug–disease, and food–drug interactions [34, 36]. Against this background, careful examination of generic tacrolimus preparations compared to the reference preparation (Prograf[R]) is essential to ensure that exposure is similar on substitution.

Although more stringent regulatory criteria for generic approval of narrow therapeutic index drugs has been adopted or considered by several regulatory agencies worldwide [8, 30, 37], it seems reasonable to assume that the more stringent criteria do not address the clinicians concerns regarding the applicability of healthy volunteer data to the target transplant population and the interchangeability between various generic formulations, which is estimated to occur in over 50% of all transplant recipients in the US. It remains important to quantitate by replicate designed trials the variability between not only disparate generic formulations, but also the branded formulation [7].

6.1. Healthy volunteer studies with tacrolimus

Regulatory approval of generic products requires evidence of equivalent relative oral bioavailability versus the originator drug in healthy volunteers, studies that are generally performed in small populations using a single-dose, two-way crossover design [38]. Kidney transplant patients, however, exhibit a higher rate of tacrolimus clearance than healthy volunteers [39], possibly due to low hematocrit and albumin levels, concomitant administration of corticosteroids [40], and high rates of disturbed gastrointestinal motility and diabetes [6]. Thus, robust pharmacokinetic data in the kidney transplant population would be highly relevant to physicians considering adoption of a generic formulation [1, 20] Specific guidance from the FDA for bioequivalence testing of tacrolimus preparations, however, does not require any special requirements other than for testing with and without food [25]. While the FDA still requires for tacrolimus that the 90% confidence interval (CI) of the ratio of the geometric means for the generic compared with the originator falls between 80% and 125% for both AUC and C_{max} [25], the European Medicines Agency (EMA) stipulates a slightly narrower window for AUC (90-111%) [30]. Under the EMA guidelines, only AUC_{0-t} is always required while C_{max} assessment is only necessary when relevant (i.e. if it is of particular importance for safety, efficacy or drug level monitoring).

Table 1 summarizes the 6 generic tacrolimus ANDA ABE data in healthy volunteers (Sandoz tacrolimus capsules ANDA application # A065461, Watson Labs tacrolimus capsules ANDA application # A090402, Dr. Reddy's Laboratory tacrolimus capsules ANDA application # A090509, Mylan tacrolimus capsules ANDA application # A090596, Accord Healthcare tacrolimus capsules ANDA application # A091195, Panacea Biotech Limited ANDA application #A090802). On September 28, 2012, ANDA application #A090802 from Panacea Biotech Limited was approved.

Table 1. *Comparison of the AUC and C_{max} in fasted and fed states after administration of different tacrolimus generic formulations in comparison to the brand (*Prograf[R]*, Astellas) in two-way cross-over healthy volunteer studies.*

Tacrolimus AUC_{inf} (fasted)

Product	Point Estimate	90% Confidence Interval	CV%
Astellas	1.22		23.55
Sandoz	1.10	1.00 - 1.21	25.04
Panacea	0.92	0.8446 - 0.996	NA
Dr. Reddy	0.96	0.90 - 1.02	16.77
Mylan	1.02	0.95 - 1.09	26.61
Accord	0.98	0.93 – 1.03	NA

Tacrolimus AUC_{inf} (fed)

Product	Point Estimate	90% Confidence Interval	CV%
Astellas	1.02		10.49
Sandoz	0.96	0.92 – 0.99	10.35
Panacea	0.90	0.838 – 0.974	NA
Dr. Reddy	1.05	0.96 - 1.15	24.87
Mylan	0.99	0.95 - 1.04	18.86
Accord	1.03	0.97 – 1.10	NA

Tacrolimus C_{max} (fasted)

Product	Point Estimate	90% Confidence Interval	CV%
Astellas	1.09		20.88
Sandoz	1.10	1.02 - 1.19	19.56
Panacea	1.00	0.937 - 1.07	NA
Dr. Reddy	0.91	0.84 - 1.00	24.40
Mylan	1.04	0.99 - 1.09	17.93
Accord	1.18	1.12 – 1.247	NA

Tacrolimus C_{max} (fed)

Product	Point Estimate	90% Confidence Interval	CV%
Astellas	1.14		25.96
Sandoz	0.96	0.90 - 1.02	16.65
Pancea	1.06	0.979 - 1.149	NA
Dr. Reddy	1.09	0.99 - 1.19	25.43
Mylan	0.95	0.89 - 1.02	23.33
Accord	1.03	0.95 – 1.13	NA

6.2. Previous Studies, Experience and Preliminary Data Relevant to this Proposal

Upon the introduction of a generic cyclosporine modified liquid in 1998, the concerns over the use of generic immunosuppressants began. In 1999, Dr. Alloway published a summary of generic immunosuppressant use in solid organ transplantation which attempted to identify the financial and scientific issues driving this field [41]. In this publication Dr. Alloway recognized the need for thorough study of these agents in order to address the factors influencing the debate. She recognized that although economics may be the driving force for generic development, these forces must be tempered by consumer safety and efficacy. With the development of more generic immunosuppressants imminent, the transplant community must continue to enforce their high standards of a research driven discipline. Higher academic demands will continue to be expected for any generic developed for use in transplantation by the practitioners [41].

In 2001, the American Society of Transplantation invited experts to review the data and issues associated with the approval and use of generic immunosuppressants. A summary of this meeting was first-authored by Dr. Alloway [9]

Dr. Alloway has served as principal investigator on several generic and tacrolimus formulation studies: 5 cyclosporine modified liquid kidney transplant recipient studies(over 50 patients enrolled); 4 tacrolimus modified release once daily formulation studies in kidney, liver and heart transplant recipients (63 patients enrolled); 5 LCP tacrolimus modified release once daily studies in kidney and liver transplant recipients(61patients enrolled); and the first tacrolimus bioequivalence study between reference and generic tacrolimus formulations in kidney transplant patients (54 patients enrolled).

SangCyA (generic modified release cyclosporine): SangCya was the first CNI generic immunosuppressant that was FDA approved. It is an oral cyclosporine liquid formulation that produced equivalent blood levels and is bioequivalent when compared with the Neoral cyclosporine formulation. Demonstration of bioequivalence is based on Food and Drug Administration (FDA) required healthy volunteer study design. However, subsequent bioequivalence studies conducted with SangCya with apple juice failed bioequivalence testing standards, therefore SangCya was removed from the market. (Department of Health and Human Services, FDA to SangStat Medical Corp; withdrawal of approval on an abbreviated new drug application; cyclosporine. Federal Register 2000; 65: 75717.)

However, prior to its market withdrawal, several transplant recipient studies were performed with this product primarily administered with water or milk with the following results. The following paragraph summarizes these results.

Studies were performed in kidney transplant patients to assess SangCya pharmacokinetic behavior compared with Sandimmune and Neoral. In addition, patients were given long-term, chronic SangCya therapy to further assess pharmacokinetic and safety parameters. Dr. Alloway was involved in all of these studies. In brief:

*Study A (SangCya vs Neoral).*Thirty-two patients were enrolled in an open label, single-center 3-period (1 week each; 2 Neoral and 1 SangCya) crossover study with a 1:1 dose.

Study B (SangCya vs Sandimmune). Twelve patients were enrolled in an open label, single-center 3-period (1 week each; 2 Sandimmune and 1 SangCya) crossover study with a 1:1 dose.

Study C (Sandimmune conversion to SangCya). Forty-two patients were enrolled in an area-under-the-concentration-time curve (AUC) based, dose-adjusted protocol with multiple blood levels drawn over the 12 hours between cyclosporine doses at weekly intervals. SangCya dose adjustments were made to match SangCya AUC to the Sandimmune AUC and the dose could not be changed more than 20%.

Study D (SangCya vs Neoral). Preliminary analysis of this ongoing study is based on 30 patients in a randomized, crossover, double-blinded design over 2 weeks. The study patient demographics included: 9 females/21 males, 10 African Americans/20 Caucasians, with a mean age of 48 years.

The results were summarized in reference [42].

6.3. Once-Daily Modified Release Tacrolimus (FKMR)

Once-daily (QD) tablets (FKMR, Advagraf, Astellas, Deerfield, IL) is an extended release formulation of tacrolimus, that is marketed in many countries, but not in the United States. This product has been studied by Dr. Alloway in stable kidney, liver, and heart transplant recipients for conversion, and in *de novo* kidney transplant recipients.

Stable kidney conversion studies and 2 year follow up [43,44]: The purpose of this pharmacokinetic (PK) study was to evaluate tacrolimus exposure in stable kidney transplant recipients converted from Prograf^R twice a day to FKMR tacrolimus QD. This was an open-label, multicenter study with a crossover design. Patients received Prograf^R twice a day through day 7; 24-hour PK profiles were obtained on days 1 and 7. Patients were converted to the same milligram-for-milligram daily dose of FKMR tacrolimus qD in the morning on day 8; 24-hour PK profiles were obtained for FKMR tacrolimus on days 8, 14, and 21. The 90% confidence intervals (CI) for the FKMR tacrolimus vs Prograf^R comparison at steady state (days 14 and 21 vs days 1 and 7) were 90.7 and 99.4 for $AUC_{0–24}$ and 82.7 and 91.9 for C_{min}. FKMR tacrolimus was well tolerated with a safety profile comparable to that of Prograf^R. $AUC_{0–24}$ was highly correlated to C_{min} for Prograf^R (day 1, r = 0.80; day 7, r = 0.84) and FKMR tacrolimus (day 14, r = 0.92; day 21, r = 0.86). Kidney function remained stable after

conversion to FKMR tacrolimus. The 2-year post conversion patient (100%) and graft (98.5%) survival, incidence of biopsy-confirmed acute rejection (6.0%), incidence of multiple rejections (1.5%), and safety profile (posttransplant diabetes, hyperlipidemia, hypertension, infections, kidney dysfunction, hepatic dysfunction, and malignancies) were consistent with or more favorable than those previously reported for TAC twice-a-day.

Stable liver conversion studies and 2 year follow up. [45] The purpose of this pharmacokinetic study was to evaluate tacrolimus exposure in stable liver transplant recipients converted from Prograf^R twice a day to FKMR tacrolimus once daily. This was an open-label, multicenter study with a single sequence, four-period crossover design. Patients received Prograf^R twice a day on days 1 to 14 and 29 to 42. Patients were converted to the same milligram-for-milligram daily dose of FKMR once daily on days 15 to 28 and 43 to 56. Twenty-four-hour PK profiles were obtained on days 14, 28, 42, and 56. The $AUC_{0–24}$ of tacrolimus was comparable for Prograf^R twice a day (days 14 and 42) and FKMR tacrolimus once daily (days 28 and 56). The 90% confidence intervals for FKMR tacrolimus versus Prograf^R at steady state (days 28 and 56 vs days 14 and 42) was 0.85 to 0.92 for $AUC_{0–24}$. FKMR tacrolimus was well tolerated with a safety profile comparable to that of Prograf^R. $AUC_{0–24}$ was highly correlated to C_{min} for Prograf^R (day 14, r = .93; Day 42, r = .89) and for FKMR tacrolimus (day 28, r = .93; day 56, r = .92). Kidney and liver function remained stable. One patient experienced acute rejection.

In an open-label, multicenter study, stable liver transplant recipients (n=69) were converted from twice-a-day tacrolimus to FKMR once-daily in the morning, and were maintained for at least 2 years post conversion using the same therapeutic monitoring and patient care techniques employed with tacrolimus. Two years after conversion, the incidence of biopsy-confirmed acute rejection was 5.8% (4 of 69); patient and graft survival was 98.6% (68 of 69). The safety profile of FKMR was consistent with that previously reported for TAC. It was concluded that liver transplant recipients can be converted from twice-a-day tacrolimus to once-daily FKMR and maintained for at least 2 years post-conversion with neither unique efficacy nor safety concerns.

Stable heart transplant patient conversion studies. [46] This phase II, open-label, multicenter, prospective single-arm study compared the pharmacokinetics of tacrolimus in stable heart transplant patients before and after conversion from twice-daily tacrolimus (Tacrolimus BID) to Tacrolimus QD. Heart transplant recipients previously maintained on Tacrolimus BID-based therapy received Tacrolimus BID from Day 1–7 and were converted on a 1:1 (mg:mg) basis to Tacrolimus QD. Five 24-hour PK profiles were collected (Days 1, 7, 8, 14, 21). Steady-state tacrolimus $AUC_{0–24}$ and C_{min} were comparable for both formulations, with treatment ratio means (90% confidence intervals) of 90.5% (86.4–94.6%) and 87.4% (82.9–92.0%), respectively (acceptance interval: 80–125%). There was good correlation between $AUC_{0–24}$ and C_{min} for Tacrolimus QD and BID (r=0.94 and 0.91, respectively).

Four year follow up in all patients switched to FKMR. [47] This was a long-term, open-label, prospective, single-arm, phase III, follow-up study of Tacrolimus QD in transplant recipients who had participated in one of four phase II multicenter studies, 1) *de novo* study in kidney transplant recipients, 2) conversion study in kidney transplant recipients, 3) *de novo* liver transplant recipients and 4) conversion study in liver transplant recipient.

The results suggested that Tacrolimus QD offers a convenient alternative to standard Tacrolimus BID for both *de novo* liver and kidney recipients and stable kidney and heart transplant recipients. The data provided reassuring evidence that kidney, liver and heart transplant patients can be maintained for up to four years post-transplant on once-daily tacrolimus dosing, providing similar efficacy and safety to the well-established twice-daily formulation.

6.4. **LCP Tacrolimus** (LCP –Tacro, once daily modified release tacrolimus)

Once-daily (qd) tablets (LCP-Tacro; Veloxis Pharmaceuticals, Hørsholm, Denmark) is an extended release MeltDose formulation of tacrolimus currently undergoing Phase 3 clinical trials. This product has been studied by Dr. Alloway in stable kidney and liver recipients for conversion, and in *de novo* kidney transplant recipients.

LCP-Tacro in stable kidney recipients [abstracts] This study evaluated safety and tolerability in stable kidney transplant recipients converted from Prograf^R twice daily to once daily LCP-Tacro with 24hr PK assessments on Day 1, 7, and 14. The tacrolimus time concentration curves are below. In this study of LCP-Tacro in stable kidney transplant recipients revealed 1) LCP-Tacro had approximately 40% higher bioavailability than Prograf^R 2) the conversion dosage ratios result in bioequivalent exposure to Prograf^R as measured by AUC, C_{min}, and C_{avg}, 3) LCP-Tacro exhibited a lower C_{max} and C_{max}/C_{min} ratio and a robust correlation between AUC and C_{min}. There were no serious adverse events related to LCP-Tacro and the adverse event profile was consistent with tacrolimus. (**Alloway RR**, Germain M, Gaber AO, Bodziak KA, Mulgaonkar SP, Gohh RY, Kaplan E, Beckery M, Gordon RD. A Phase II, open-label, multi-center prospective, conversion study in stable kidney transplant patients to compare the pharmacokinetics of LCP-Tacro tablets once-a-day to Prograf^R capsules twice-a-day. (Poster) American Transplant Congress, Toronto, Ontario-Canada, May 31-June 4, 2008)

LCP Tacro in stable liver recipients [abstracts] This study evaluated safety and tolerability in stable liver transplant recipients converted from Prograf^R twice daily to once daily LCP-Tacro with 24hr PK assessments on Day 1, 7, and 14. This study of LCP tacrolimus in stable liver transplant recipients revealed 1) LCP-Tacro had approximately 31% higher bioavailability than Prograf^R 2) the conversion dosage ratios result in bioequivalent exposure to Prograf^R as measured by AUC, C_{min}, and C_{avg}, 3) LCP-Tacro exhibited a lower C_{max} and C_{max}/C_{min} ratio and a robust correlation between AUC and C_{min}. There were no rejection episodes, graft loss or death. One serious adverse event occurred, but it was unrelated to LCP-Tacro. Otherwise, the adverse event profile was consistent with tacrolimus. (**Alloway RR**, Eckhoff DE, Teperman LW, Washburn WK, Tzakis AG, Gaber AO, Wiesner RH, Freeman RB, del Rio Martin JV, Chudzinski RE, Lake JR, Katz E, Griffin HE, Gordon RD, Jexner Hamburg K, Chodoff L. A Phase 2, open-label, multi-center, prospective conversion study to compare the pharmacokinetics and safety of LCP-Tacro tablets once-a-day to Prograf^R capsules twice-a-day in stable liver transplant patients: Results at 12 months. *(Oral)* European Society for Organ Transplantation, Paris, France, August 30-September 2, 2009.)

This study was conducted to collect long term pharmacokinetic and safety data on the liver patients converted in the previous study. The systemic exposure, peak systemic exposure and trough tacrolimus levels were similar after six months of therapy (Week 26) compared to Day 14 of therapy. The degree of fluctuation, the degree of swing and the median Tmax values did not statistically differ between Day 14 and Week 26 of therapy. On both Day 14 and Week 26 , LCP-Tacro demonstrated a statistically significant correlation between AUC_t and C_{min}. Liver and kidney functions were stable throughout the study. (Washburn WK, **Alloway RR**, Eckhoff DE, Teperman L. A Phase II Open label multi-center extension study In stable liver transplant patients To assess the pharmacokinetic profile, safety and tolerability of once-daily extended release MeltDose®Tacrolimus tablets (LCP-Tacro™) In patients after six months of therapy *(Poster)* American Transplant Congress, Boston MA, June 2012)

LCP Tacro in *de novo* kidney recipients. [abstracts] This study evaluated long-term safety and tolerability in *de novo* kidney transplant recipients with 24hr PK assessments on Day 1, 7, and 14 in recipients randomized to receive Prograf^R or LCP-Tacro at the time of transplant. In this study, the proportion of patients within specified concentration range on Day 7 were 66.7% and 75% for LCP-Tacro and Prograf^R, respectively. On Day 14 the proportions were 78.8% and 57.1%. On Day 14, there was a robust correlation between C0 and AUC for both LCP tacro and Prograf^R. Graft and patient survival were 100% in both groups at one year. Treatment failure (death, graft loss, rejection, loss to followup) was 6.3% and 9.7% for LCP tacro and Prograf^R, respectively (p=0.67). **(Alloway RR**, Mulgaonkar S, Bowers VD, Ueda Stevenson KR, Cohen DJ, Katz E, Kosar H, Gordon RD, Jexner Hamberg K, Chodoff L. A Phase 2b, open-label, multi-center, prospective, randomized study to compare the pharmacokinetics and safety of LCP-Tacro tablets once-a-day to Prograf^R capsules twice-a-day in

de novo kidney transplant patients. *(Poster)* American Transplant Congress, Boston MA, May 29-June 4, 2009.)

6.5. Sandoz generic tacrolimus

To our knowledge, this is the first prospective study undertaken specifically to compare the pharmacokinetic characteristics of a generic tacrolimus preparation versus the reference drug in kidney transplant patients. Dr. Alloway worked extensively with the sponsor, Sandoz, on the design, conduct, and analyses of these study results. Dr. Alloway's site enrolled 54 patients from The University of Cincinnati Medical Center and The Christ Hospital kidney transplant programs. These results are described in more detail below. The manuscript was accepted for publication in the American Journal of Transplantation in May 2012 and a copy is attached in Appendix 1.

The above paragraphs clearly document the depth and breadth of Dr. Alloway's expertise in this field, her active participation and input since the advent of the first generic CNI, as also the very substantial contributions that she has made to this area of investigation.

6.6. Dr. Alloway's representation for the American Society of Transplantation (AST) on Generic and Clinical Trial Organization.

Dr. Alloway's expertise in generic immunosuppressive agents is well recognized within the leadership of the American Society of Transplantation (AST). She was invited by the AST Board to attend and report on the summary of the two FDA workshops by FDA CDER Advisory Committee for Pharmaceutical Sciences and Clinical Pharmacology in 2010 and 2011. Dr. Alloway also represented the AST as an organizing committee member and speaker for the FDA Workshop on Issues on Clinical Trial Endpoints in Kidney Transplantation. She has also been a faculty member and speaker at several AST-sponsored meetings discussing clinical trial organization.

6.7. Co-Investigators

Dr. Alloway will be supported by Dr. Vinks (Site PI, Cincinnati Children's Medical Center) and Dr. Christians (Site PI, University of Colorado). Dr. Vinks is a worldwide recognized expert in immunosuppression, pharmacogenetics, pharmacokinetics and pharmacometrics and has extensively published in these areas. Dr. Christians is laboratory director of a College of American Pathologists (CAP)-accredited and CLIA-certified therapeutic drug monitoring mass spectrometry laboratory. Dr. Christians has more than 20 years of experience with measuring tacrolimus, its metabolites and its clinical pharmacokinetics. In fact, in 1991, Dr. Christians' group was the first to publish a tacrolimus LC-MS assay and the first to describe the tacrolimus metabolites. Since 1996, he also has made continuous active contributions to the ongoing discussions of generic immunosuppressant drugs. Dr. Vinks' and Dr. Christians' publications directly relevant to this application are too numerous to discuss here. For more details, please see Dr. Vinks' and Dr. Christians' CVs.

<u>AIM 1:</u> *Identification of the most disparate tacrolimus generic drug formulations among those currently approved in the United States.*

7. Introduction

Generic tacrolimus products are available in one dosage form, capsules. There are a total of 6 Abbreviated New Drug Applications (ANDAs) approved for tacrolimus tablets up to September 2012. The manufacturers include Sandoz, Watson, Dr. Reddy's Lab, Mylan Pharmaceuticals Inc, Accord Healthcare Inc., and Panacea Biotech Ltd. (Sandoz tacrolimus capsules ANDA application # A065461, Watson Labs tacrolimus capsules ANDA application # A090402, Dr. Reddy's Laboratory tacrolimus capsules ANDA application # A090509, Mylan tacrolimus capsules ANDA application # A090596, Accord Healthcare tacrolimus capsules ANDA application # A091195, Panacea Biotech Limited ANDA application #A090802). On September 28, 2011, ANDA application # A090402 from Watson was withdrawn from the market. The ANDAs were approved based upon healthy volunteer data with the 5mg capsule in fasted and fed states. Approval of other strengths (0.5mg and 1mg) were based upon dissolution studies. Of note, the Sandoz generic is now available as both tacrolimus, Sandoz and Hecoria™, a branded generic. Therefore, currently there are 5 generic manufacturers of tacrolimus generic for 1mg strengths. **To most efficiently address the aforementioned public**

concerns with not only prescribability, but switchability of these generic tacrolimus formulations, we propose to (A) identify the two most disparate bioequivalent tacrolimus generics among those currently approved in the United States (ie, Generic Hi and Generic Lo) and (B) to compare their relative bioequivalence and pharmacokinetics with each other as well as with the brand formulation, Prograf^{R}, in the proposed clinical trial as described in Aim 3.

7.1. Justification and Feasibility

Pre-existing data. Establishing the most disparate products is not trivial. As a first step we propose to use differences in bioequivalence data under fasting and fed conditions to prescreen ANDAs for further selections

The ANDA ABE data for AUC and C_{max} in the fasting and fed conditions were compared. The following figures of these data allows for ease of comparison. Figure 3 compares the generic tacrolimus formulations for the pharmacokinetic parameter AUC_t.

The upper and lower confidence interval (CI) of AUC_t under fasting conditions for these five ANDAs varied between 90 and 121 with a range of point estimates of 0.98 to 1.22. The upper and lower confidence interval (CI) of AUC_t under fed conditions for these five ANDAs varied between 90 and 115 with a range of point estimates of 0.94 to 1.05.

The ANDAs were than ranked by AUC_t under fasting and fed conditions by the following (Table 3):

1) geometric mean ratio,

2) greatest distance of CI, and

3) greatest distance of CI from point estimate of 100.

Figure 3. *Comparison of 90% CIs for AUCt and point estimates of tacrolimus formulations under fasting and fed conditions.*

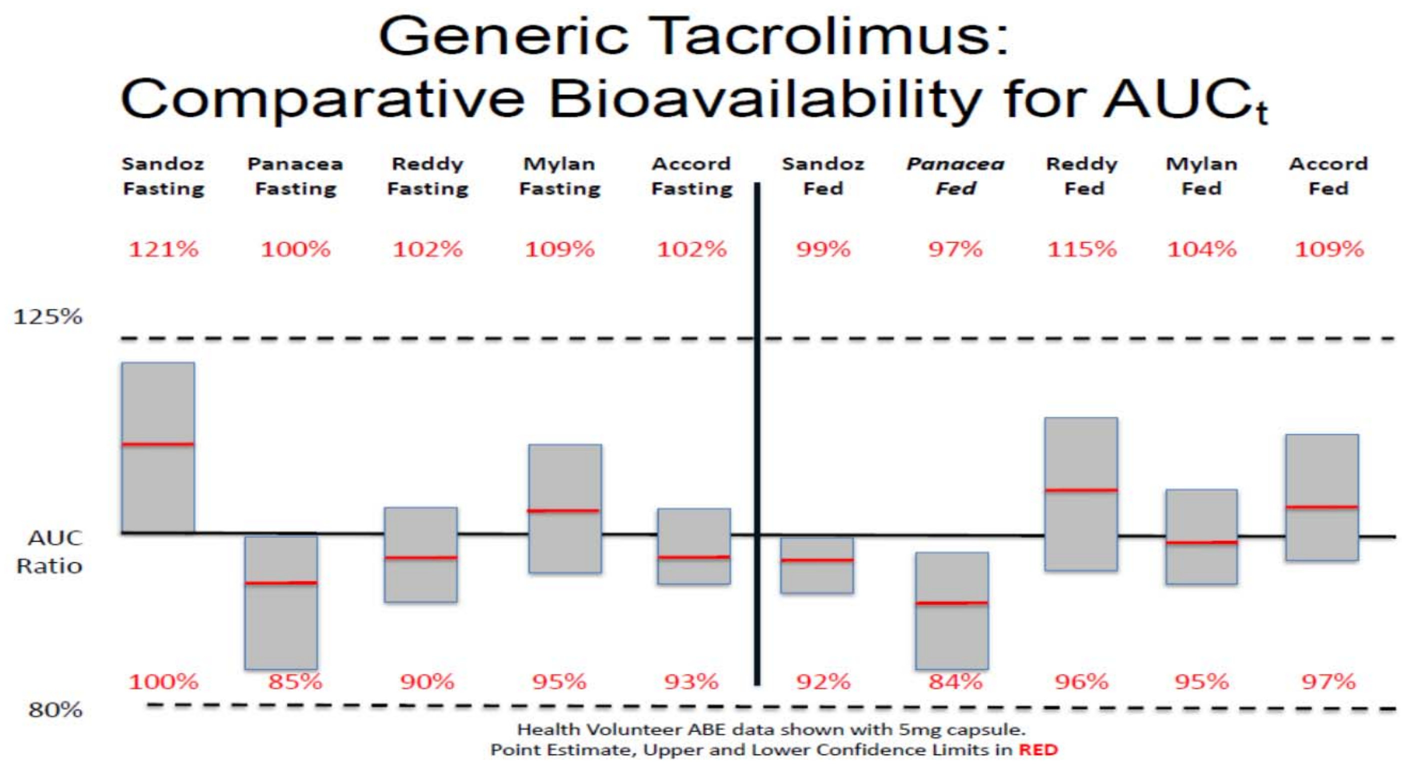

Table 3. *Ranking of tacrolimus formulations based on the bioequivalence data presented in Figure 3.*

**Formulation**	**AUC$_t$ fasting**			**AUC$_t$ fed**		
	Geometric mean Ratio (1-5, lo to hi)	Greatest distance of CI (1-5, lo to hi)	Greatest distance of CI from 100 (1-5, lo to hi)	Geometric mean Ratio (1-5, lo to hi)	Greatest distance of CI (1-5, lo to hi)	Greatest distance of CI from 100 (1-5, lo to hi)
Sandoz	5	5	5	2	1	2
Panacea	1	4	4	1	4	5
Dr. Reddy	2	2	3	5	5	4
Mylan	4	3	2	3	2	1
Accord	3	1	1	4	3	3

Of note, the food effect on the Sandoz formulation results in a decrease in point estimate while the food effect on the Dr. Reddy formulation results in an increase point estimate. In attempts to utilize ANDA ABE data, Pangraf (Pancea Biotech Ltd) could Generic Lo based upon AUC$_t$ data, however there is no obvious Generic Hi.

Figure 4 compares the generic tacrolimus formulations for the pharmacokinetic parameter C_{max}.

Figure 4. Comparison of the bioequivalence of C_{max} values of tacrolimus formulations under fasted and fed conditions.

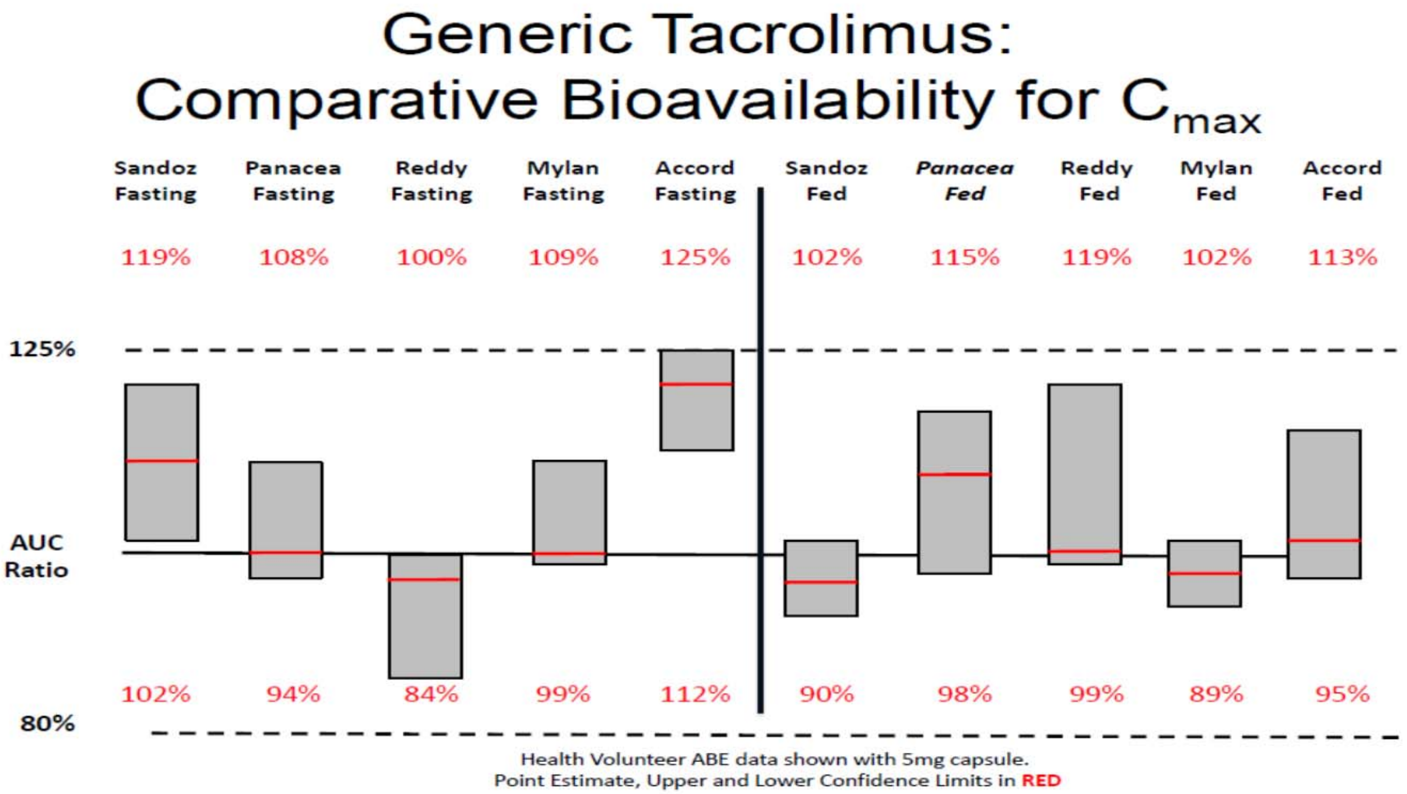

The upper and lower confidence interval (CI) of C_{max} under fasting conditions for these five ANDAs varied between 83 and 125 with a range of point estimates of 0.91 to 1.18 (Figure 4). The upper and lower confidence interval (CI) of C_{max} under fed conditions for these five ANDAs varied between 89 and 119 with a range of point estimates of 0.95 to 1.09. The ANDAs are ranked by C_{max} under fasting and fed conditions by the following:

1) geometric mean ratio,

2) greatest distance of CI, and

3) greatest distance of CI from point estimate of 100.

Table 4. *Ranking of tacrolimus formulations based on the bioequivalence data presented in Figure 4.*

Formulation	C_{max} fasting			C_{max} fed		
	Geometric mean Ratio (1-5, lo to hi)	Greatest distance of CI (1-5, lo to hi)	Greatest distance of CI from 100 (1-5, lo to hi)	Geometric mean Ratio (1-5, lo to hi)	Greatest distance of CI (1-5, lo to hi)	Greatest distance of CI from 100 (1-5, lo to hi)
Sandoz	4	5	4	2	1	1
Panacea	2	2 (same)	1	4	3	4
Dr. Reddy	1	4	3	5	5	5
Mylan	3	1	2	1	2	2
Accord	5	2 (same)	5	3	4	3

The food effect on formulation by point estimates formulations is not uniform. The Dr. Reddy formulation exhibited greatest food effect based upon confidence interval. In this comparison of Cmax point estimates, there is no obvious product that would represent Generic Hi or Generic Lo.

When attempting to combine the AUC_t and C_{max} point estimate and confidence interval data to identify a Generic Hi and a Generic Lo, there are no obvious candidates.

8. Product Information

Due to the lack of clear ANDA ABE data to identify Generic Hi and Generic Lo, further product selections are based on the following

Potency profile differences

Dissolution profile differences

Formulation differences

It also needs to be noted that the studies compared in Figures 3 and 4 as well as Tables 3 and 4, do not take potential variability in production lots and among different capsule strengths into account. That different strength tacrolimus capsules are not necessarily identical in terms of relative bioavailability was shown in reference [48, 49]. In a first study five 1 mg capsules (test) failed to meet bioequivalence acceptance criteria when tested against one 5 mg capsule (reference, all Prograf[R]).

Thus, we propose to identify the most disparate generic lots and formulations using *in vitro* chemical assay (potency) and dissolution data after testing 3 lots of tacrolimus capsule strength (1mg) from each available manufacturer.

All *in vitro* testing will be conducted at The University of Iowa Pharmaceuticals (UIP) (please also see letter of support). UIP is the largest and most experienced university-affiliated FDA-registered pharmaceutical manufacturing facility in the United States. UIP has been developing formulations, manufacturing products,

and conducting analytical testing in compliance with current Good Manufacturing Practices (cGMPs) since 1974. UIP is fully compliant with GMP regulations (21 CFR parts 11, 210, and 211).

Initial dissolution screening as well as comparison of chemical potency and purity will be conducted on three lots of tacrolimus purchased from the following manufacturers, 1) Astellas (brand) Prograf^{R}, 2)Sandoz, 3) Dr. Reddy's Lab, 4) Mylan Pharmaceuticals Inc, 5) Accord Healthcare Inc., 6) Panacea Biotech Ltd. As aforementioned, the 1mg strength will be tested. Assurance of adequate product and shelf-life of each lot to complete the entire study (dissolution and subject administration) will be attempted. From these results, the manufacturer to represent Generic Hi and Generic Lo will be identified as study product.

Potency and Purity. Potency and purity will be tested at the University of Colorado (iC42 Clinical Research and Development, Laboratory Director: U. Christians) using an established HPLC-UV-ion trap mass spectrometry assay. This assay is validated following all applicable and current FDA and Clinical and Laboratory Standards Institute guidances [50, 51] and is compliant with USP requirements. iC42 Clinical Research and Development is compliant with all applicable regulatory requirements. In brief, tacrolimus will be quantified based on UV detection at 205 nm wavelength. Ascomycin will be used as internal standard. All processes and data will be reviewed by iC42's quality assurance unit (QAU).

The ion trap mass spectrometer will be run in the full scan mode (m/z= 50-1000) will serve a dual purpose: as qualifier to ensure the purity of the detected tacrolimus peaks (tacrolimus elutes in a dual peak pattern due to limited rotation around the piperidine nitrogen) during quantification and, in addition, the ion trap mass spectrometer will be able to give first structural information regarding impurities. Most important will be to determine if these are related to tacrolimus (e.g. known adducts and degradation products). As deemed necessary, the structures of impurities will further be identified using MS^n, data base searches and NMR spectroscopy of the isolated material underlying the impurity peaks.

Dissolution: Dissolution testing will be performed according to the current USP monograph for tacrolimus capsules as well as mentioned in applicable FDA guidance [25] and standard test method <711> using the time testing profile specified on the FDA dissolution testing database [26] using VanKel and /or Distek dissolution testing stations. The pH will be adjusted to 4.5 using phosphoric acid in compliance with [25,26].

Prior to performing any dissolution testing the current USP monograph dissolution method for Tacrolimus Capsules will be qualified for use in the UIP laboratory. This qualification will be performed according to an approved protocol and comply with UIP standard operating procedures. This is important as tacrolimus is not water-soluble and it has to be ensured that the drug will not precipitate during the procedure, sample handling and storage. A total of 3 different lots of 6 manufacturer's products will be tested (1mg strength) to determine the fastest and slowest dissolving products. Samples will be collected at 30, 60, 90 and 120 min. We will put one capsule per cell of the dissolution apparatus and run 6 cells (= n=6/ lot of each capsule strength/ formulation, total 1080 samples). Samples will be frozen and shipped on dry ice to iC42 Clinical Research and Development where they will be quantified using the above-mentioned HPLC-UV-ion trap mass spectrometry assay. Even if the manufacturers of the brand and/or generics may have used difference dissolution testing methods during the development of their formulation and to establish bioequivalence, we will only use the above-mentioned FDA-recommended testing method as it is important that all products be tested with the same dissolution test method under identical, well-controlled, validated and documented conditions.

The results will be reported as profiles of percent of amount dissolved versus time.

Formulation Differences: Specific formulation data will be obtained from the FDA on all products for final product assessment of differences. Excipient information will be assessed for potential sources of product

variability. As it will be one of the goals to assess the concern that the formulation may affect intestinal first pass drug metabolism and transport of tacrolimus, we will assess available information regarding all excipients in regards to known p-glycoprotein and cytochrome P4503A and 3A5 interactions. For example, the generic Hexal cyclosporine formulation contains large amounts of liquid vitamin E. Liquid vitamin E is known to affect intestinal p-glycoprotein efflux and subsequently intestinal drug metabolism and drug-drug interactions [52,53]. If necessary, the iC42 Clinical Research and Development laboratory is set up to assess the potential effects of excipients on cyctochrome P4503A5 and 3A5-mediated tacrolimus metabolism and p-glycoprotein-mediated efflux in additional *in vitro* studies [54,55] as deemed necessary.

Selection of Final Products for Lot Testing

Based upon the previously described criteria, six approved tacrolimus capsule ANDAs were considered for the selection of the most disparate products using the following scheme: 1) initial selections were based on differences in BE data under fasting and fed conditions. The ANDAs were first ranked based on the point estimate (PE) of Cmax and AUC. For those with similar PE, the products with greater distance of the lower or upper bounds of the confidence interval (CI) from reference 100% received higher ranks. 2) Further selections were based on differences in impurity and dissolution profiles, formulation compositions, manufacturing processes, bioequivalence study sites and strengths. The tacrolimus predicted high and low should differ most in these aspects.

Based on all factors considered above, the following ANDAs are recommended as the most disparate ANDAs for the BE studies:

Tacrolimus predicted low: ANDA 090802 (ANDA 90509 will be the backup if ANDA 90802 is not available)

Tacrolimus predicted high: ANDA 065461

	**090802 (A)**	**065461 (B)**
Bioequivalence data	Under fasting, the point estimate of AUC and Cmax for Generic A and B differed by 19% and 9%, respectively.	
Bioequivalence study sites	Asia and North American respectively	
Bioequivalence strength	1 mg and 5 mg	5 mg
Formulation composition	Similar	
Fill weight	1 mg capsule, 85 mg in size “5” capsule;<br><br>5 mg capsule, 140 mg in size “4” capsule	1 mg capsule, 50 mg in size “4” capsule;<br><br>5 mg capsules, 250 mg in size “3” capsule
Manufacturing process	Similar	
Dissolution limits	Different	
Impurity limits	Different in specified impurity limit	

	C (Low backup, ANDA 090509)	B (ANDA 065461)
Bioequivalence data	Under fasting, the point estimate of AUC and Cmax for Generic C and B differed by 15% and 18%, respectively.	
Bioequivalence study sites	Asia and North American respectively	
Bioequivalence strength	5 mg	5 mg
Formulation composition	Similar	
Fill weight	1mg capsule, 140 mg in size “4” capsule<br><br>5 mg capsule, 140 mg in size “4” capsule	1 mg capsule, 50 mg in size “4” capsule<br><br>5 mg capsules, 250 mg in size “3” capsule
Manufacturing process	Similar, processing solvent slightly different	
Dissolution limits	Different	
Impurity limits	Slightly different	

This data was summarized by the FDA and presented to the U01 investigative team and representatives of AST and ASTS. It was agreed by the FDA, transplant community representatives and the U01 investigative team to proceed with lot testing of the above products upon final protocol approval. Concerns exist regarding the market availability of ANDA 090802. To our knowledge as of January 2013, this product has not been sold in the United States. The plan includes making all attempts to test ANDA 090802, but not to delay study conduct.

8.1. Selection of the Final Products and Lot

During the development of a medicinal product a dissolution test is used as a tool to identify formulation factors that are influencing and may have a crucial effect on the bioavailability of the drug [30]. Dissolution testing is used to ensure both batch-to-batch consistency and that the dissolution profiles remain similar to those of pivotal clinical comparators. Furthermore, a dissolution test can be used to demonstrate bioequivalence [30]. Hence, we consider the following the primary selection criteria to identify Generic Hi and Lo: Chemical potency and Dissolution rate.

Chemical potency will determine systemic exposure (pharmacokinetic surrogate marker: AUC_{τ}) and dissolution rate the rate of absorption (surrogate markers: t_{max} and C_{max}), all of which are critical acceptance criteria of the clinical bioequivalence study.

As it is the goal to identify the most disparate individual generic formulations/ lots, the calculation of distribution statistics and statistical comparison would be counterproductive. Therefore, in terms of chemical potency the different individual lots independent of generic formulation will simply be ranked. We will use the same strategy for the dissolution rates. We propose to use the percent amount released at 90 min. Since each experiment will be carried out with n=4 for each lot and formulation, we will calculate the geometric means instead of means to

minimize the influence of outliers. As in the case of the different individual lots of the generic formulations, they will simply be ranked.

The selection of the lots for the study will be based upon the following: 1) The lot with the highest potency and most rapid dissolution will be selected for the Generic Hi (= lowest sum of both rank numbers), 2) The lot with the lowest potency and slowest dissolution will be selected for the Generic Lo (=highest sum of both rank numbers). Where the potency and dissolution data conflict, the investigators will examine the data to select the most disparate lots for the *in vivo* study and formulation differences. For example, if the dissolution data is not conclusive, we will compare other time points such as the 60 min, 30 min and 120 min data in this order. If there still should be a tie, we will select Generic Hi and Lo based on formulation differences. This means we will select the formulations that, based on the known effects of its excipients is most likely to affect intestinal drug metabolism and transport versus the formulation that is the least likely to do so.

Recently Dr. Jiang et al presented a poster at AAPS entitled, "Selection of Disparate Generic Lamotrigine Tablets for Bioequivalence Studies in Epilepsy patients." While this is the first public report, the goal of this study is to select the most disparate generic tacrolimus formations by the same procedures.

The final product selection will be made by a group consisting of representatives from the study investigative team, the FDA, the AST (American Society of Transplantation) and the ASTS (American Society of Transplant Surgeons).

Ultimately the intent is to study the specific lot of the generic product predicted to result in the highest levels and compare it to the specific lot from another generic product predicted to result in the lowest levels.

9. Statistical Analysis

Independent of the primary goal of Aim 1 to determine Generic Hi and Generic Lo, the data generated here will be of considerable value as this will be the first study to systematically compare the tacrolimus brand and all generic formulations approved in the United States under identical conditions, including a comparison of their different formulation strengths in three different production lots. Data will be tested for distribution and log-transformed as appropriate. Data will be compared using analysis of variance using Dunnett's t-test or Scheffé's methods as post-hoc test. Co-variate analysis may be considered as deemed appropriate. In addition, the similarity of dissolution testing results may be compared by model- independent or model-dependent methods e.g. by statistical multivariate comparison of the parameters of the Weibull function or the percentage dissolved at different time points as suggested in reference [30]. In addition we will consider calculating a similarity factor such as the f2 similarity factor defined in Appendix 1 of reference [30]. As far as possible, all statistical calculations will be carried out using the SAS statistics package (version 9.03, SAS Institute, Cary, NC). All SAS programming will be carried out by iC42 Clinical Research and Development's SAS programmer, Mr. J. Consoer (included in the per-sample price calculations iC42's/ University of Colorado's site budget), under Dr. Zung's supervision.

9.1. Expected Outcomes

A significant quantity of each lot will be obtained to conduct the initial dissolution screening. Upon identification of the product lots to over encapsulate as the finished product, it is expected that a significant quantity of each lot will be obtained to provide sufficient product to conduct the patient testing.

<u>Potential Problems and Alternative Strategies:</u>

An anticipated problem may arise from the fact that we are comparing FDA-approved bioequivalent generic tacrolimus generics. Although we will make every attempt to identify the Generic Hi and Lo lots/ formulations based on a pre-defined clear scientific rationale, we may fail to identify Generic Hi and Lo using the

comprehensive strategy described above. If this is the case, this will already address a series of concerns published in numerous consensus documents and published reviews and editorials in terms of potential differences in quality and purity of tacrolimus generics and their potential inferiority compared to the brand formulation.

In this event, all data will be reviewed by the principal investigators and the scientific staff at UI Pharmaceuticals and the final product will be selected. Evaluation of ABE data from fasting study are weighted more than the fed study if necessary to identify Generic Hi and Generic Lo. In the worst case, we propose to simply test the generic with the highest current market share (Sandoz, in its branded Hecoria version) with the generic with the second highest current market share in the United States (Dr. Reddy, see Figure 2) in the clinical trial in Aim 3 as the chances that transplant patients are switched between these two generic tacrolimus formulations is highest and thus clinically most relevant.

Identification of product is of concern from two perspectives, 1) Quantity of product from a single lot for entire study conduct, and 2) Product expiration date of at least 2 years after the start of dissolution testing to assure product from the same lot to complete pharmacokinetic study. All attempts will be made to assure product lots utilized in the initial dissolution testing will ultimately be provided to the subjects for pharmacokinetic study.

To address the issue of quantity, an analysis of drug requirement for dissolution testing and subject administration was estimated. It is estimated that over 36,000 tacrolimus 1mg capsules will be needed to conduct this study (Table 5).

Table 5. *Estimation of tacrolimus capsules required for the entire project.*

**Product**	**Strength**	**Initial Dissolution Screening***	**Subject Study Supplies****	**Product Totals**
Product 1		#	#	#
(Brand)	1.0 mg	300	11400	13300
Product 2				
(High)	1.0 mg	300	11400	13300
Product 3				
(Low)	1.0 mg	300	11400	13300
Product 4				
	1.0 mg	300		300
Product 5				
	1.0 mg	300		300
Product 6				

	1.0 mg	300		300

***One bottle of 100 capsules of 3 lots of each product 1mgstrength.**

****Average doses based upon similar previous kidney transplant bioequivalence studies.**

To address the issue of shelf-life, a sampling of various local pharmacies was conducted to estimate current product labeling as it relates to shelf-life (Table 6).

Table 6. *Survey of expiration dates of tacrolimus formulations currently available in pharmacies as of May 2012.*

**Sampling of Product Expiration Dates by Pharmacy (May 2012)**				
**Pharmacy**	**Prograf^R 0.5mg**	**Prograf^R 1mg**	**Generic Tacrolimus 0.5mg**	**Generic Tacrolimus 1mg**
1	August 2013	June 2014		Mylan<br>May 2013
2		June 2014	Mylan<br>November 2013	
3		June 2014		Mylan<br>September 2013
4	July 2014	October 2012	Dr Reddy<br>September 2013	Dr Reddy<br>November 2013
5		January 2014	Dr Reddy<br>October 2013	Dr Reddy<br>October 2013

Upon contact with the manufacturers to request product dating, the investigator was told this information was proprietary. (Alloway, personal communication)

All attempts will be made to assure that product lots from the initial dissolution testing will ultimately be provided to the subjects for pharmacokinetic study. However, if product is not available, additional lots will be purchased and previously described potency, dissolution, and impurity testing will be repeated on the new lots. This will have a significant budgetary impact, therefore, all efforts will be made to compress the time from the initiation of product identification and study enrollment.

10. Rationale: AIM 2 Blinding of the study results during analysis

The next step will be to blind the formulations of Brand, Generic Hi and Generic Lo to minimize potential investigator and patient bias. As illustrated in Table 7 below, the capsules are of significantly different color, shape and size. This limits the potential blinding strategies to “dump and refill” into a standard-size capsule and over-encapsulation. Both of these strategies have their advantages and disadvantages. The major problem with over-encapsulation is that the additional capsule may potentially affect the relative bioavailability and pharmacokinetics, that a motivated patient may remove the over-encapsulation and identify the capsule inside and thus unblind the formulation, and that in several case the filler material may need to be added to avoid rattling in case of smaller capsules. Such fillers may also potentially affect absolute oral bioavailability and pharmacokinetics. Although “dump and refill”, opening of the original capsule and filling its contents into a neutral capsule, seems associated with less risk of affecting relative bioequivalence and pharmacokinetics, this is a complex multi-step procedure that will be beyond the budgetary limits of this RFA. Upon consideration of these limitations, a consensus was reached between the investigators, representatives from the transplant community and the FDA to avoid either method of dosage form blinding. In an effort to preserve study integrity, the following procedures will be followed.

Table 7. *Comparison of the capsule sizes and appearance of the different tacrolimus formulations currently on the United States market.*

	0.5 mg capsule	1 mg capsule	5 mg capsule
**Brand (Prograf®)**			
Astellas	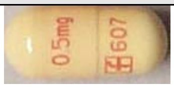	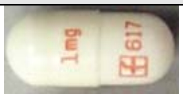	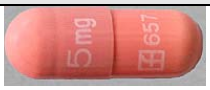
**Generic Products**			
Sandoz (generic)	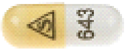	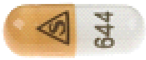	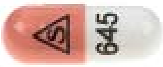
Hecoria™ (Novartis branded generic)	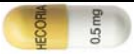	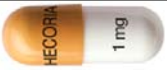	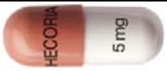
Dr Reddy's Laboratories (generic)	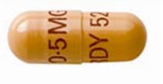	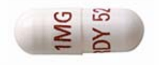	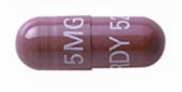
Mylan (generic)	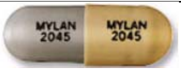	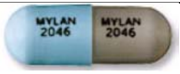	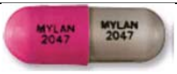
Accord healthcare (generic)	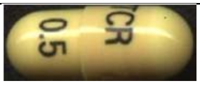	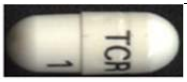	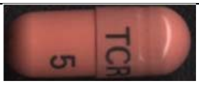

Table 8. *Blinding Method Summary*

Study Activity Site	Blinding Method
PK Study Site	Subjects randomly assigned to study drug sequence by the IDS Pharmacy based upon consecutive enrollment. Sequence of study drug administration will be unknown to study staff.
Analytical Site	Samples will be shipped to the analytical site identified only by subject number and date. Levels will be quantitated and results provided to the PK analysis site.
PK Analysis Site	PK analysis site will be provided the patient number and study drug sequence by the IDS pharmacy and will analyze the results accordingly.

11. Expected Outcomes, Potential Problems and Alternative Strategies

Blinding of this study is essential to address public concern with generic formulations and to prevent any subject or investigator bias. "Dump and refill" blinding techniques may be ideal due to the fact it is impossible to identify the formulation encapsulated. However this method of blinding was cost prohibitive as estimated at over US$800,000. Fillers utilized in the overfilling process may impact the pharmacokinetics of the formulations differently. Since over-encapsulation may significantly affect the relative bioavailability and pharmacokinetics of the study tacrolimus formulations (Brand, Generic Hi and Generic Lo), we will blind the study results at other points of analysis to insure overall study integrity.

AIM 3: *Comparison of the replicate relative bioavailability and steady-state pharmacokinetics of Brand, Generic Hi and Generic Lo in a prospective, appropriately powered, fully replicated, blinded, 3-way cross-over study including kidney (n=38) and liver (n=38) transplant patients*

12. Rationale and Review of the Relevant Literature

Several consensus documents related to generic immunosuppressant use in solid organ transplantation have been published by the American Transplant Society, National Kidney Foundation, European Society of Organ Transplantation, International Society of Heart and Lung Transplantation, and a Canadian Perspective [1,6-10,60].

In general, these consensus documents acknowledge the considerable debate regarding the safety and efficacy of generic drug substitution in solid organ transplant recipients. As already discussed above, the safety and efficacy concerns primarily stem from the extrapolation of healthy volunteer bioequivalence to transplant recipients and doubts pertaining to confidence in interval testing for bioequivalence. All consensus reports call for more research to confirm clinical and therapeutic equivalence and pharmacoeconomic benefit.

In a prospective, observational study of conversion from reference to generic tacrolimus (Sandoz) in 70 patients from four centers, McDevitt-Potter *et al.* [61] reported only minor changes in mean tacrolimus dose (4.4mg to 4.5mg, p=0.89) and mean tacrolimus trough concentrations (5.8 ng/mL to 5.9 ng/mL, p=0.81) after conversion, although dose adjustments were more frequent than in the same patients at a point six months previously (21% of patients versus 7% of patients). This latter finding is not unexpected since more intensive monitoring would be likely following conversion to a generic preparation or other changes in the immunosuppression regimen.

In two retrospective studies in which data were collected from kidney transplant patients converted from reference to generic tacrolimus (Sandoz), involving 45 patients [62] and 75 patients [63] the tacrolimus dose required to maintain therapeutic trough levels was similar with both preparations and trough levels were maintained [62,63]. In these trials no acute rejection occurred following conversion other than in one patient (1/75, 1.3%) with a history of rejection. Limited data comparing *de novo* use of generic tacrolimus (Sandoz) or reference tacrolimus in kidney transplant patients showed no difference in dose requirements and trough concentrations levels with both preparations [64]. One case of biopsy-proven acute rejection has, however, been reported after an inadvertent conversion from reference to generic tacrolimus (Sandoz) [65]. The rejection was not associated with a change in tacrolimus trough concentration and the authors suggested that the subsequent impairment in kidney function was more likely to be due to underlying chronic allograft nephropathy [65], but that the risk of inadvertent switch at the pharmacy level is of concern.

<u>PIs Preliminary Studies:</u> A prospective, multicenter, open-label, randomized, two-period (14 days per period), two-sequence, crossover, steady-state pharmacokinetic study was undertaken to compare twice-daily generic tacrolimus (Sandoz) versus reference tacrolimus (Prograf^R) in stable kidney transplant patients. Of 71 patients enrolled, 68 provided evaluable pharmacokinetic data. During Period 1 (days 1-14), patients in Sequence 1 received reference tacrolimus (Prograf^R, Astellas Pharma, Deerfield, IL) and patients in Sequence 2 received generic tacrolimus. During Period 2 (days 15-28), the two groups crossed over to receive the alternative preparation. The generic formulation was Sandoz tacrolimus (Novartis Pharmaceuticals, East Hanover, NJ). The mean (SD) tacrolimus dose at baseline was 5.7 (4.2) mg/day (median 4.0 mg/day, range 0.5-20.0 mg/day). All patients received an unchanged dose throughout the study. Blood samples were collected pre-dose (C_0) and at 0.5, 1, 1.5, 1.75, 2, 3, 4, 8 and 12 hours after dosing. The resulting tacrolimus time concentration curves are provided in Figure 5.

Figure 5. *Comparison of the steady-state time-concentration profiles of tacrolimus after administration of Prograf*[R] *(brand, reference) and Sandoz (generic test) formulations to 68 stable kidney transplant patients in a two-sequence randomized, open-label cross-over study.* Please see also Table 9.

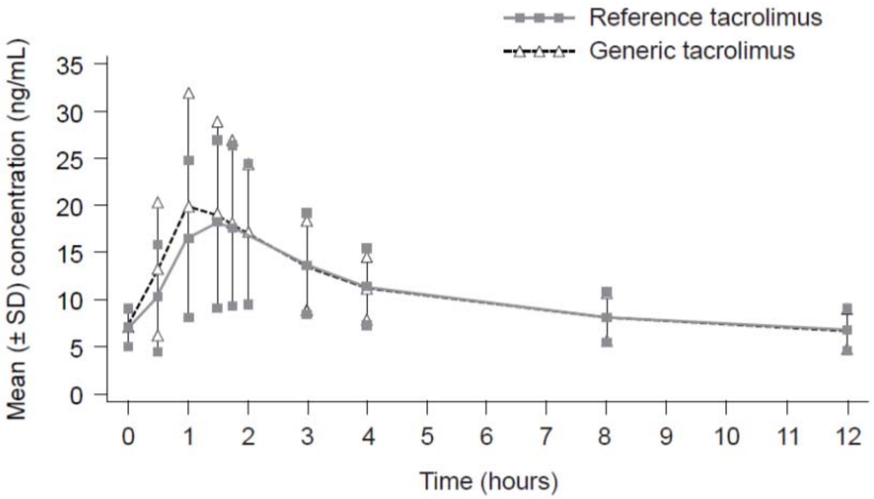

There were no significant differences in $AUC_{0\text{-}12h}$, C_0, C_{max} or t_{max} between the generic and reference preparations based on mean values of data obtained on days 14 and 28. Resulting pharmacokinetic parameters were as follows (Table 9).

Table 9. *Comparison of steady-state pharmacokinetic parameters after administration of Prograf*[R] *and Sandoz generic tacrolimus formulations to 68 kidney transplant patients in a two-sequence cross-over study.*

	**Generic tacrolimus**	**Reference tacrolimus**	**P value**
Dose-normalized $AUC_{0\text{-}12h}$, ng.h/mL	61.8±40.6	60.0±37.8	0.409
Dose-normalized C_{max}, ng/mL	9.6±5.5	9.1±5.5	0.199
C_0, ng/mL	7.3±1.8	7.0±2.1	0.354
T_{max}, hours	1.5±1.1	1.9±1.3	0.073

The ratios of geometric means of Brand versus Generic were 1.02 (90% CI of 97-108%, p=0.486) for $AUC_{0\text{-}12h}$, 1.09 (90% CI 101-118%, p=0.057) for C_{max} and 1.02 (90% CI of 0.95-1.09, p=0.651) for C_{12} (Table 10).

Table 10. *Geometric mean ratios and 90% confidence intervals (90%CI) as well as statistical comparison (2-sided t-test) after oral administration of tacrolimus brand (Prograf) and generic (Sandoz) in 68 stable kidney transplant patients.*

	**Ratio of geometric means**	**90% CI**	**P value**
(a) Generic tacrolimus			
$AUC_{0\text{-}12h}$			
Day 7 versus day 14	0.96	0.90, 1.03	0.327
Day 21 versus day 28	1.04	0.96, 1.12	0.450
C_{max}			
Day 7 versus day 14	0.98	0.90, 1.07	0.735
Day 21 versus day 28	1.06	0.94, 1.19	0.423
(b) Reference tacrolimus			
$AUC_{0\text{-}12h}$			
Day 7 versus day 14	0.96	0.91, 1.02	0.282

Day 21 versus day 28	0.98	0.91, 1.05	0.570
C_{max}			
Day 7 versus day 14	0.96	0.88, 1.05	0.459
Day 21 versus day 28	1.00	0.89, 1.12	0.970

For generic tacrolimus and reference tacrolimus, the mean (SEM) CV values were 13.4 (10.4) % versus 11.0 (9.8) % for $AUC_{0\text{-}12h}$, 16.9 (15.5)% versus 17.9 (14.9)% for C_{max}, and 13.2 (9.8)% and 11.1 (10.3) % for C_0, respectively. Correlations (r values) between C_{12} and $AUC_{0\text{-}12h}$ were 0.837 and 0.917 for generic tacrolimus at days 14 and 28, respectively, compared to 0.773 and 0.887 for reference tacrolimus.

The findings of the current study indicate that the Sandoz generic tacrolimus is bioequivalent to reference tacrolimus when assessed pharmacokinetically in a population of stable kidney transplant recipients. This manuscript was recently accepted for publication in American Journal of Transplantation [66]. Since it is not yet publically available, a copy has been included as Appendix to this grant application.

Tacrolimus Metabolism. It has been criticized that bioequivalence testing of immunosuppressants is limited to establishing bioequivalence of the parent drug [67]. Tacrolimus is extensively metabolized to more than 8 metabolites by cytochrome P4503A4 and 3A5 enzymes already during the first pass by the small intestine and the liver [12,36,54]. In trough blood samples, the concentrations of the metabolites add up to approximately the same concentration as the parent [68-70]. Although based on current knowledge the tacrolimus metabolites do not significantly contribute to overall immunosuppression (the active 31-O-desmethyl is only a minor metabolite) and/or toxicity [12], the metabolite patterns and potential changes hereof are clinically highly relevant, as in most transplant centers tacrolimus therapeutic drug monitoring is carried out using immunoassays. The antibodies on which such immunoassays are based upon are known to cross-react with several of the metabolites independent of their biological activity [12]. For example, several major metabolites in blood such as 15-O-desmethyl and 13,15-O-didesmethyl tacrolimus cross react with the antibody of the most commonly used immunoassay 90.5 and 92.2%, respectively [12,71]. This means that immunoassays overestimate the tacrolimus concentrations due to cross-reactivity with inactive metabolites. Thus, therapeutic concentration windows for patients monitored with immunoassays are usually higher than for those monitored with specific LC-MS/MS assays. This also means that the results of tacrolimus therapeutic drug monitoring assays depend on the metabolite/ parent ratios and that a change of these ratios may affect tacrolimus dosing decisions, potentially leading to over- or under-dosing a patient. There are two ways, the formulation of tacrolimus can potentially affect tacrolimus metabolite patterns:

(1) If excipients are used that are known to affect cytochrome P4503A enzymes and/or p-glycoprotein activity in the intestinal mucosa [72].
(2) If the formulation moves the absorption of the drug to more distal parts of the intestine. Drug metabolizing enzymes and drug transporters have different expression levels in different parts of the intestine [36,73,74].

Since there is evidence that tacrolimus drug-drug interactions may already occur on the intestinal level [54,75], this may also affect the extent of drug-drug interactions. To address these concerns, we will measure tacrolimus metabolite concentrations using LC-MS as already described by our group for the assessment of potential ethnic differences in tacrolimus pharmacokinetics [76].

As discussed above, one of the major concerns is that current bioequivalence guidelines require testing in healthy human individuals, but not in the target patient population [6,9,67]. It is noted that not only different patient populations can differ significantly from healthy individuals, but also among each other. To address this concern, we proposed to enroll kidney (n=38) and liver transplant patients (n=38). These patient groups differ in many aspects. One of the most important is that tacrolimus is metabolized by the liver and therefore a liver transplant will directly affect tacrolimus metabolism and pharmacokinetics. The difference between tacrolimus metabolite patterns in kidney and liver transplant patients have been studied by our group [76]. Although it seems highly unlikely that the metabolism of tacrolimus and the pharmacokinetics of the metabolites after absorption from a brand or generic formulation will be different in the same patient for the reasons discussed in

detail by us in [22], we will compare tacrolimus metabolite patterns and establish their equivalence in kidney and liver transplant patients to directly address any further concerns and discussions in this direction.

Tacrolimus pharmacogenetics. In consensus documents discussing immunosuppressant bioequivalence testing, high risk subpopulations have been discussed [6,60]. In addition to pediatric patients, these are so-called "poor absorbers". These patients typically have more variable tacrolimus pharmacokinetics than "normal absorbers" [78]. It has been shown that these differences in relative bioavailability are due to cytochrome P4503A5 polymorphisms and p-glycoprotein/ ABCB1 haplotype, which also explain the observed ethnic differences in tacrolimus bioavailability and pharmacokinetics [76]. A link between the polymorphisms of CYP3A4 and 3A5 and ABCB1 genes and the daily dose to achieve adequate tacrolimus blood concentrations has been established [79-85]. Therefore, we propose to genotype patients in terms of their cytochrome P4503A5 genotype and ABCB1 and to assess bioequivalence after stratification based on cyctochrome P4503A5 genotype and ABCB1 haplotype. Seven functional single nucleotide polymorphisms (SNPs) are selected in the genes of drug metabolizing enzymes CYP3A4/5, P450 oxidoreductase and drug transporter ABCB1/MDR1 [86-95]. These SNPs have been reported as being associated with the pharmacokinetics of tacrolimus (priority level 1). Eleven SNPs in the genes of nuclear factors, PXR, CAR and HNF4a [97-105], are also selected for screening purpose for potential influencing factors on CYP3A activity since they regulate CYP3A expressions (priority level 2). Additionally, eight minor SNPs (priority level 3) are selected for exploring influencing SNPs. These SNPs will be assessed by direct sequencing for patients who show extreme discordant tacrolimus concentrations. For a complete list, please see Table 13 in section 7 of the clinical protocol below. All recipients will have genotyping sample as per the assessment schedule (table 11). Donor samples for kidney and liver transplant recipients are collected and stored as per standard of care. When available, deceased donor samples will be obtained for genotyping. Living donor samples will be de-identified and linked via their donor identification number to the recipient for genotyping as outlined in the protocol. Living donors will be contacted via telephone and asked for consent to use their stored sample for genotyping. A note to file will be placed in the recipient's subject chart and the living donor will be mailed two copies of the consent form (one to keep, one to return). Dr. Alloway will call the living donor and use a telephone script to consent the subject over the phone.

Adherence monitoring. As we will compare the pharmacokinetics of Brand, Generic Hi, and Generic Lo under steady-state conditions, pharmacokinetic profiles will be collected after the patients have taken the study medication for one week. Adherence is critical to ensure that steady-state is reached at the time the pharmacokinetic profile for bioequivalence testing is collected. In addition to the usually monitoring methods such as diaries and pill counts, we propose to use an innovative electronic monitoring method, the Medication Event Monitoring System (MEMS®, AARDEX, Palo Alto, CA) [104]. This method has been shown by our collaborators at the Cincinnati Childrens' Medical Center to be a reliable and valid method and has the significant advantage of tracking the frequency, timing, and pattern of medication taking [105]. The MEMS system is similar to a prescription bottle, but contains a micro-electronic chip in the cap that registers dates/times when the bottle was opened and closed. Time-stamped medication events are stored in the MEMS and transferred to software (PowerView) that records the daily history of medication taking.

13. Research Design

The study is designed to compare the replicate steady-state pharmacokinetics of Prograf^{R} (Brand) and the two most disparate generic formulations (Generic Hi and Generic Lo) in a fully replicated, blinded, 3-way cross-over study in stable kidney (n=38) and liver transplant (n=38) subjects.

14. Study objectives

14.1. Primary objectives

- To estimate the ratio of C_0, C_{12}, $AUC_{0\text{-}12h}$ and C_{max} and apply CI testing at steady state of Prograf^{R} to generic Hi in stable kidney and liver transplant subjects.
- To estimate the ratio of C_0, C_{12}, $AUC_{0\text{-}12h}$ and C_{max} and apply CI testing at steady state of Prograf^{R} to Generic Lo in stable kidney and liver transplant subjects.

- To estimate the ratio of C_0, C_{12}, $AUC_{0\text{-}12h}$ and C_{max} and apply CI testing at steady state of Generic Hi to Generic Lo in stable kidney and liver transplant subjects.

14.2. Secondary objectives

- To compare bioavailability of each tacrolimus formulations in stable kidney and liver transplant subjects using the dose-normalized C_0, C_{12}, $AUC_{0\text{-}12h}$ and C_{max} data.
- To evaluate intra-patient variability of tacrolimus pharmacokinetics of each formulation by comparing C_0, C_{12}, $AUC_{0\text{-}12h}$, and C_{max}.
- To evaluate and compare the pharmacokinetics of tacrolimus metabolites in terms of C_0, C_{12}, $AUC_{0\text{-}12h}$, C_{max} and intra-individual variability
- To compare the safety and efficacy of Prograf^R, Generic Hi and Generic Lo in stable kidney and liver transplant subjects.

14.3. Exploratory objectives

These are described in more detail in **Aim 4**. In brief these include:

- Stratified bioequivalence analysis based on cytochrome P4503A5 genotype and ABCB1 haplotype as well as based on diseases potentially interfering with tacrolimus bioavailability and pharmacokinetics (diabetes versus non-diabetic)
- Population pharmacokinetic analysis
- Bioequivalence analysis using narrower acceptance intervals such as suggested in [30,37]
- Testing of individual bioequivalence
- Testing of scaled average bioequivalence

15. Investigational Plan

15.1. Logistic overview

There are 3 components of the study. The clinical component managed by Dr. Alloway, the level analysis component managed by Dr. Christians, and the data analysis managed by Dr. Sander.

For the clinical component, Dr. Alloway's research team will obtain a HIPAA waiver to prescreen patients for study participation via monitoring routine lab results and clinic visits. Upon identification of potential subjects, the physician will be contacted for clearance prior to obtaining informed consent. Pending physician approval, the initial study introduction may occur via phone or routine clinic visit. Persons agreeing to proceed will be consented and additional screening procedures completed in their respective outpatient transplant clinic. The outpatient kidney transplant clinics are located at The Christ Hospital and University of Cincinnati Medical Center. The liver outpatient clinic is located at University of Cincinnati Medical Center. Study enrollment and proceeding visits will occur at The Garfield Suites, 2 Garfield Place, Cincinnati, OH 45202. The Garfield Suites is a hotel our research team has adapted for study conduct to promote comfort and full completion of all PK visits. Subjects will be provided a hotel room the night before and the night of their pharmacokinetic study visits. The day of enrollment, they will travel to The Garfield Suites to familiarize themselves with the surroundings to ensure on time arrival. For the next PK study visits, we will gather all subjects in the hotel lab draw area and begin the study visits as described below in detail. During these visits, a doctor of pharmacy (Rita Alloway, Adele Shields, Tiffany Kaiser) who is co-investigator will supervise the study drug dosing, adherence and timing of blood sample collections. Research coordinators will conduct the blood draws and will be available throughout the study period. There are no emergency services located at The Garfield Suites. In the unlikely event of an emergency, 911 will be called. If necessary, all research staff are certified in basic life support. Twenty-four/seven access is available to study physicians.

Our extensive successful experience with prolonged pharmacokinetic studies such as this includes facilitating study completion by providing an environment (i.e. hotel) that allows for the subject to relax as much as possible by bringing a companion, etc.

15.2. Study design

As aforementioned this is an open label, prospective, multicenter, randomized, replicate, six-period, three-sequence cross-over study to compare the steady state pharmacokinetics of Prograf[R] to Generic Hi to Generic Lo in stable kidney and liver transplant subjects. The PK assessor will be blinded to the assigned treatment sequence and formulation. The person analyzing the levels and analyzing the results will be blinded to formulation sequence. Each subject will be randomized to one of the three sequences where Generic Hi and Generic Lo represent the two generics and B the brand, Prograf represented in Figure 6 below.

Figure 6. Randomization Sequence

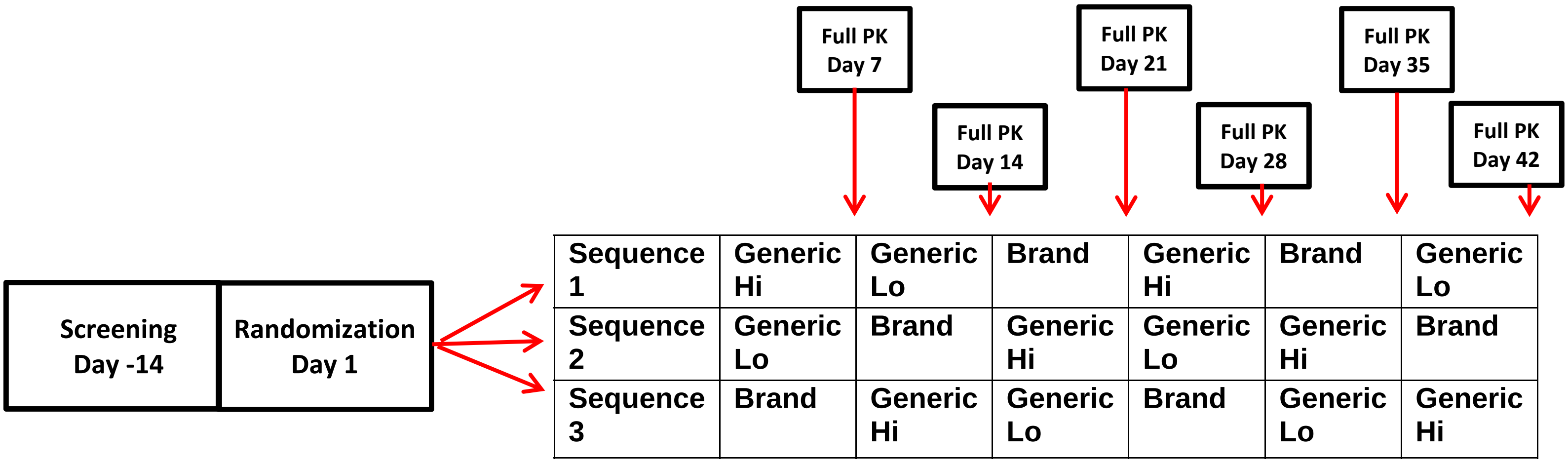

Sequence 1	Generic Hi	Generic Lo	Brand	Generic Hi	Brand	Generic Lo
Sequence 2	Generic Lo	Brand	Generic Hi	Generic Lo	Generic Hi	Brand
Sequence 3	Brand	Generic Hi	Generic Lo	Brand	Generic Lo	Generic Hi

15.3. Collection of pharmacokinetic profiles

The 6 period trial design is chosen to perform replicate, full pharmacokinetic assessments on each tacrolimus formulation. Full pharmacokinetic assessments will involve blood sampling over 12 hours starting with trough (C0 - before morning dosing) and continuing over a 12 hour period. The blood samples will be collected at C0 (before morning dose) and then 20, 40, 60(1hr), 80, 100, 120(2hr), 140, 160, 180(3hr) minutes, 4, 5, 6, 8 and 12 hour after dosing with each formulation.

15.4. Sample handling and storage

Whole blood is the matrix of choice and specimens will be collected into tubes containing ethylene diamine tetraacetic acid (EDTA) anticoagulant. Heparin anticoagulation is not recommended because of the tendency to form clots on storage. All samples will be collected and aliquot into 2 tubes and frozen at -80°C. All samples for each patient must be frozen, batched and shipped together for blinded analysis at iC42 Clinical Research and Development (University of Colorado) on dry ice using an overnight courier. One aliquot on each patient must be kept on site frozen until all samples for each patient have been completely analyzed at the bioanalytical laboratory. Samples will be stored at -80°C in an access-controlled and freezer at iC42 Clinical Research and Development until the final study report/ the last manuscript has been accepted. iC42 Clinical Research and Development freezers are constantly monitored, alert personnel automatically if the temperature increases above an alert threshold and samples will be transferred into a back-up freezer within one hour or less. In case electricity in the building is down, liquid nitrogen backup for cooling the freezers is available. All samples will be handled, stored and archived following applicable iC42 Clinical Research and Development standard operation procedures.

16. Population

The study population will consist of male and female subjects (≥ 18 years old) who are stable kidney or liver transplant recipients (at least 6 months post-transplant) and meet the inclusion/exclusion criteria. Subjects who enter the proposed study will be on a stable twice-daily dose of Prograf[R] or generic tacrolimus, as deemed

appropriate by their physicians. At least thirty-eight kidney subjects will be randomized in 2 clinical centers. At least thirty-eight liver subjects will be randomized in one clinical center. The transplant centers/programs participating in this multicenter trial are:

- The Christ Hospital, Cincinnati, co-investigator: Drs. M. Cardi and Adele Shields
- The University of Cincinnati Medical Center, Kidney Transplant Program: co-investigator: Drs. S. Woodle and Rita Alloway
- The University of Cincinnati Medical Center, Liver Transplant Program: co-investigator: Drs. K. Sherman and Tiffany Kaiser.

For more details, please see the clinical co-investigators' CVs.

16.1. Inclusion criteria

Subjects will be screened and enrolled into the trial if they meet all the inclusion criteria on day of study entry (Visit 1) and on the day of randomization (Visit 2).

Subjects eligible for inclusion in this study have to fulfill **all** of the following criteria:

1. ≥18 years old, male or female
2. Able to participate and willing to give written informed consent and to comply with the study visits and restrictions.
3. Subject who has received a primary or secondary kidney or liver transplant.
4. Subject who is at least 6 months post-transplant and on a stable dose of tacrolimus as defined by physician, one tacrolimus trough level within the physician defined target range within past 6 months and one additional trough level during the screening period within 30% of the physician defined target range.
5. BMI greater than or equal to 19 but less than or equal to 40.
6. Ability to perform daily finger sticks to provide blood sample.

16.2. Exclusion criteria

Subjects will be screened and enrolled into the trial if they meet none of the exclusion criteria on day of study entry (Visit 1) and on the day of randomization (Visit 2).

Subjects fulfilling **any** of the following criteria are not eligible for inclusion in this study:

1. Evidence of any acute rejection
2. Subjects who require dialysis within 6 months prior to study entry
3. Recipients of multiple organ transplants
4. Subjects who have tested positive for HBsAG or HIV, or who are recipients of organ from donors who are known to be HBsAG or HIV positive. Virology screening at the time of transplant.
5. HepC positive subjects with liver biopsy proven recurrent disease considered relevant by physician oversight.
6. Subjects with any severe medical condition requiring acute or chronic treatment that in the investigator's opinion would interfere with study participation
7. History of malignancy, treated or untreated, with the past 2 years with the exception of carcinoma in situ or excised basal cell carcinoma, or hepatocellular carcinoma prior to transplant.
8. GFR ≤ 35 ml/min measured as estimated using the MDRD4 formula
9. Subjects with AST, ALT, total bilirubin ≥ 3 X ULN or other evidence of severe liver disease
10. Subjects with white blood cell (WBC) count ≤2,000/ mm3 or with thrombocytopenia (platelet count ≤ 75,000/ mm3), with an absolute neutrophil count of ≤ 1,500/ mm3 or hemoglobin <8g/dL)
11. Subjects with clinically significant infections, requiring therapy, which, in the investigator's opinion, would interfere with the objectives of the study
12. Other mental or physical conditions which in the investigator's opinion, are considered clinically significant
13. Presence of intractable immunosuppressant complications or side effects resulting in dose adjustment of tacrolimus
14. Subjects who have been exposed to an investigational therapy within 30 days prior to enrollment or 5 half-lives of the investigational product, whichever is greater.
15. An anticipated change in the immunosuppressive regimen during subject participation other than that required by the protocol

16. Subject with severe GI disturbance or diarrhea which could interfere with tacrolimus absorption
17. Severe diabetic gastroparesis
18. Initiation of any medications that could interfere with tacrolimus blood levels, including OTC medications, herbal supplements, grapefruit or grapefruit juice.
19. Pregnant or nursing (lactating) women, where pregnancy is defined as the state of a female after conception and until the termination of gestation, confirmed by a positive BhCG laboratory test (> 5 mIU/mL)
20. Women of child-bearing potential, defined as all women physiologically capable of becoming pregnant, unless they are
    a. women whose career, lifestyle, or sexual orientation precludes intercourse with a male partner; women whose partners have been sterilized by vasectomy or
    b. using a highly effective method of birth control (i.e. one that results in a less than 1% per year failure rate when used consistently and correctly, such as implants, injectables, combined oral contraceptives, and some intrauterine devices (IUDs); periodic abstinence (e.g. calendar, ovulation, symptothermal, post-ovulation methods) is not acceptable.

17. Treatment

17.1. Investigational and control treatment

Brand: Prograf[R] Capsules, 1.0mg
Tacrolimus capsules containing white to off white powder equivalent to 1.0 mg of anhydrous tacrolimus are hard gelatin capsules with white opaque body and ivory capsules.
Generic Hi: Generic tacrolimus Capsules, 1.0mg. Manufacturer to be determined (Aim 1).
Generic Lo: Generic tacrolimus Capsules, 1.0mg. Manufacturer to be determined (Aim 1).

As discussed in detail above, Brand and two generic formulations will be purchased from the same drug lot number based on the results of Aim 1. The product will be provided to the Investigational Drug Services.
The Investigational Drug Services at the participating sites will dispense the prescribed study drug to the subjects according to their stable tacrolimus dosage in a blinded fashion in MEMs controlled bottles (*vide infra*)

Justification for Time-to-Steady State. As aforementioned, bioequivalence will be tested under steady state conditions. Based on the information provide (106), the average terminal half-life of tacrolimus in healthy individuals is 34.8 hours, that in transplant patients 18.8 hours (kidney) and 11.7 (liver). Assuming that 5 terminal half-lives are required to reach steady state, 5 x 34.8/ 24= 7.25 days will be required for tacrolimus in healthy individuals. This means that, as proposed in the present study, dosing for 7 days is adequate to reach steady state, especially when considering that the study is based on kidney and liver transplant patients that, as aforementioned, have substantially shorter average terminal half-lives than the 34.8 hours reported for healthy individuals.

To ensure that individual subjects had tacrolimus concentrations in the expected target concentration range and have most likely reached steady state, subjects will collect trough capillary dried blood samples (DBS) after finger stick on a daily basis until the day of PK sample collection. DBS can be stored at room temperature in a Ziploc bag that will be provided. The subjects will give the DBS filter cards to the clinical investigator at the beginning of the study visits. The tacrolimus concentrations will be quantified using the LC-MS/MS assay described in Section 8. This data will be used to identify patients who most likely did not reach steady state before the PK collection.

17.2. Randomization to treatment arms

In the study subjects who are on stable dose of tacrolimus will be assigned a subject number in sequence at the transplant center according to the study entry. The statistician (Dr. Zung) will have generated a randomization list using the SAS Proc PLAN (version 9.03). This list will only be made available to the study pharmacist who will assign the appropriate tacrolimus formulations. Under no circumstances will this list be shared with any other study personnel including the Principal Investigator or the patients before the study is completed, with exception of an emergency as described in 4.3. below. Subjects will be randomized into one of

the three sequences shown in Figure 6 (*vide supra*). At Visit 2 (Day 1) an eligible subject will be given the lowest available randomization number from the randomization list. This number assigns the subject to one of the treatment sequences. The investigator will enter the randomization number into the electronic CRF.

17.3. Treatment blinding and unblinding

The study will be blinded in terms of the tacrolimus formulation sequence to the PK study site, study product to the analytical site, and the pharmacokinetic analysis site after PK period results assessed. Only the statistician and the investigational pharmacist will know the treatment assignment sequences. Under no circumstances will an individual patient sequence be unblinded. The only exception is if a physician determines that this is an emergency situation with potentially severe medical consequences. In this case, the Principal Investigator can agree to unblinding of a specific patient. However, it is not anticipated that this will occur as all patients receive the same drug (tacrolimus) at known doses in FDA approved bioequivalent formulations. The study sequence will be unblinded only after all queries have been resolved and the data based is officially locked.

17.4. Subject numbering

Each subject is uniquely identified in the study by a unique number as assigned by the randomization list. Once assigned to a subject, a subject number will not be reused. If the subject fails to be randomized for any reason, the reason for not being randomized will be entered on the Screening Log.

17.5. Supply, storage, dispensing and tracking of study medication

Study treatment must be received by the Investigational Pharmacy at the study site, handled and stored safely and properly, and kept in a secured location to which only the Investigational Pharmacist can access. Upon receipt, all study drugs should be stored according to the instructions specified on the drug labels. Clinical supplies are to be dispensed only in accordance with the protocol.

Medication labels will be in the English language and comply with the US legal requirements.
They will include storage conditions for the drug, but no information about the subject.

The investigational pharmacist will supply the appropriate study drug according to the randomization list in packaging of identical appearance. Subjects will be dispensed study drug based upon sequence assignment for 9 days (7 days and 2 day for window or contaminated doses).

The investigator must maintain an accurate record of the shipment and dispensing of study drug in a drug accountability ledger. Monitoring of drug accountability will be performed by the field monitor during site visits and at the completion of the trial. Subjects will be asked to return all unused study drug and packaging at the end of the study or at the time of study drug discontinuation.

At the conclusion of the study, and as appropriate during the course of the study, the investigator will return all used and unused study drug, packaging, drug labels, and a copy of the completed drug accountability ledger to the lead investigator.

17.6. Instructions for prescribing and taking study treatment

Subjects who consent to the trial and meet all the inclusion criteria and none of the exclusion criteria will be entered into the trial. Thus, only subjects who are on a stable dose of tacrolimus will be entered into the trial and the dose of tacrolimus will be maintained during the study period.

After randomization subjects, will enter into Sequence 1-6 (Figure 6). During the trial subjects will be instructed to take tacrolimus without food.

Full PK assessment will be performed on Day 7 (+2), Day 14 (±2), Day 21 (±2), Day 28 (±2), Day 35 (±2), and Day 42 (±2). Subjects will be instructed to take study drug after an overnight fast, and no food is allowed until 2 hours post-dose. Study will end on Day 42(±2). A two day window will be allowed for PK assessments in the case of inclimate weather, patient scheduling conflicts, or other unforeseen circumstances.

All dosages prescribed and dispensed to the subject and all dose changes during the study must be recorded on the Dosage Administration Record CRF(s).

The investigator should promote compliance by instructing the subject to take the study drug exactly as prescribed and by stating that compliance is necessary for the subject's safety and the validity of the study. The subject should be instructed to contact the investigator if he/she is unable for any reason to take the study drug as prescribed.

17.7. Permitted dose adjustments and interruptions of study treatment

Study drug dose adjustments and/or interruptions are not permitted, unless indicated by the principal investigator for any clinical reason. Any change must be recorded on the Dosage Administration Record CRF(s). Subjects requiring dose changes will be withdrawn from the trial.

17.8. Adherence monitoring

Adherence will be monitored using a combination of:
- patient diary review
- pill counts
- the MEMS system
- daily finger stick for tacrolimus level assessment

As mentioned above, MEMS is similar to a prescription bottle, but contains a micro-electronic chip in the cap that registers dates/times when the bottle was opened and closed. Time-stamped medication events are stored in the MEMS and transferred to into the PowerView software and a database (in combination with SQL2012, both Microsoft) that records the daily history of medication taking. This information will be exported to SAS for statistical analysis. Patients will be instructed to take their medication only from the MEMS bottle for the duration of the study, not to open the bottle unless they are taking a dose of medication at that time, and to close the bottle immediately after removing the prescribed dose. A standardized form will be used during each download to capture information regarding extra openings, refills, and periods of nonuse. Adherence will be defined as the number of times that doses of oral medication were taken as prescribed. Electronic monitoring of oral medication usage has been used by this investigatory team to study medication adherence in a range of pediatric chronic illnesses [105].

Lack of adherence can have a negative effect on the patient reaching steady state of tacrolimus exposure by the time of PK sample collection. As aforementioned, individual patients will be monitored by daily dried blood spot collection for tacrolimus trough blood concentration monitoring.

17.9. Rescue medication

Use of rescue medication must be recorded on the Concomitant medications/Significant non-drug therapies and/or Immunosuppressive medication CRF.

Any drug that interferes with the pharmacokinetics of the tacrolimus should not be initiated or changed during the study period. If it is necessary for the subject's safety to add any medication that interferes or changes the PK of tacrolimus, the subject should be withdrawn from the trial as per the principal investigator's discretion.

17.10. Concomitant treatment

Prior treatments, defined as those taken within 30 days prior to screening, should be recorded on the Concomitant medications/Significant non-drug therapies and/or immunosuppressive medications CRFs. The investigator should instruct the subject to notify the study site about any new medications he/she takes after the start of the study drug. All medications and significant non-drug therapies (including physical therapy and blood transfusions) administered after the subject starts treatment with study drug must be listed on the Concomitant medications/Significant non-drug therapies CRF.

17.11. Prohibited treatment

Since tacrolimus is metabolized mainly by the CYP3A enzyme systems, substances known to inhibit these enzymes may decrease the metabolism or increase bioavailability of tacrolimus [36] as indicated by increased whole blood or plasma concentrations. Drugs known to induce these enzyme systems may result in an increased metabolism of tacrolimus or decreased bioavailability as indicated by decreased whole blood or plasma concentrations [36]. Monitoring of blood concentrations and appropriate dosage adjustments are essential when such drugs are used concomitantly. Please refer to tacrolimus prescribing information for further details on drug-drug interactions. Patients who will require dose changes or require a new treatment with such medications during the study period will be withdrawn.

17.12. Discontinuation of study treatment and premature subject withdrawal and replacement of drop-outs.

Subjects may voluntarily withdraw from the study for any reason at any time. They may be considered withdrawn if they state an intention to withdraw, fail to return for visits, or become lost to follow-up for any other reason. If premature withdrawal occurs for any reason, the investigator must make every effort to determine the primary reason for a subject's premature withdrawal from the study and record this information on the Study Completion CRF.

The investigator should discontinue study treatment for a given subject or withdraw the subject from study if, on balance, he/she believes that continuation would be detrimental to the subject's well-being.
Study treatment *must* be discontinued and the subject withdrawn from the trial under the following circumstances:

- Withdrawal of informed consent
- Emergence of the following adverse events: acute rejection or graft loss
- Non adherence (4.4.5.).
- Tacrolimus dose changes
- Initiation of any new drug that interferes with tacrolimus metabolism and pharmacokinetics [36]
- Pregnancy
- Any other protocol deviation that results in a significant risk to the subject's safety

The Study Completion CRF form should be completed, giving the date and primary reason for stopping study drug. Please see Table 11 for the required assessments of these subjects after discontinuation of study drug.
For subjects who are lost to follow-up (i.e. those subjects whose status is unclear because they fail to appear for study visits without stating an intention to withdraw), the investigator should show "due diligence" by documenting in the source documents steps taken to contact the subject, e.g. dates of telephone calls, registered letters, etc.

Subjects who are prematurely withdrawn from the study will be replaced by an equal number of newly enrolled subjects.

17.13. Study completion and post-study treatment

A subject will be considered to have completed the study when he or she completes the end of study visit. The duration of the study is approximately 56 days. If a subject is discontinued at any time after entering the study, the investigator will make every effort to see the subject as soon as possible and completed end of study assessments. The investigator must provide follow-up medical care for all subjects who are prematurely withdrawn from the study, or must refer them for appropriate ongoing care.

Subjects will be paid $3000 if they complete the study or prorated $500 for each of the six Pharmacokinetic visits. Subjects requiring immediately available funds to complete study participation can be advanced $100 for each study visit upon request. The total amount paid will not exceed $3000. The purpose of this payment strategy is to provide immediate funds necessary for a subject to participate in the study, but clearly communicate the importance of full completion of all pharmacokinetic visits for viable data analysis. This method of payment has historically been successful and acceptable by participating subjects.

17.14. Early study termination

The study can be terminated at any time. Should this be necessary, the subject should be seen as soon as possible and undergo an end of study visit. The investigator may be informed of additional procedures to be followed in order to ensure that adequate consideration is given to the protection of the subject's interests. The investigator will be responsible for informing IRBs and/or ECs of the early termination of the trial. Terminated subjects will be instructed NOT to stop study medication until they have verified an alternative source of tacrolimus. Subjects will be instructed to return all study medication at the time and an end of study visit will be conducted to evaluate safety and efficacy. The subject may terminate at any time during the study by simply informing any study or medical personnel.

18. Visit schedule and assessments

Visit 1 (Screen -14): Subjects will be screened and enrolled into the trial if they meet all the inclusion and none of the exclusion criteria on day of study entry (Visit 1). Subjects will be followed for 14 days and must be on the stable dose of tacrolimus during this time period as defined by the protocol. If any tacrolimus dose adjustment or addition of any medication that can affect tacrolimus levels is expected during this time period, the subject should not be randomized into the trial. Complete medical history, demography, prior concomitant medications/significant non-drug therapies and immunosuppressive medications, physical examination, vital signs, transplant background information, and clinical laboratory testing (including pregnancy test) will be performed at this visit. Samples will be collected for MDR1/ABCB1 haplotype and cytochrome P4503A5 genotype 3A5 expression. Subjects will be maintained during the screening on their usual stable dose of twice daily of tacrolimus.

Visit 2 (Randomization): Subjects will be reevaluated for inclusion/exclusion criteria on the day of randomization for eligibility. Subjects will be randomized to the tacrolimus formulation sequence (Figure 6), on the day of randomization on milligram for milligram basis and will start with the period 1 study medication. Medical history and prior concomitant medications/significant non-drug therapies and immunosuppressive medications will be reviewed. Vital signs and clinical laboratory testing will be performed. After start of study drug, all adverse events/serious adverse events, infections, concomitant medications/significant non-drug therapies, immunosuppressive medications, kidney allograft rejection, kidney allograft biopsy, graft loss and dosage administration will be recorded on the CRF. The patient will be instructed to provide blood via a lancet finger stick onto protein saver cards daily during the entire study period. The patient will be instructed to collect this sample after an overnight fast and prior to their daily morning dose. Collection time will be documented in the patient diary.

Visit 3 (Day 7 after randomization): Absorption of tacrolimus from the gastrointestinal tract after oral administration is incomplete and variable. The absolute bioavailability of tacrolimus was 17± 10% in adult kidney transplant subjects (N=26), 22 ± 6% in adult liver transplant subjects (N=17) and 18 ± 5% in healthy volunteers (N=16) [39]. The rate and extent of tacrolimus absorption were greatest under fasted conditions. The presence and composition of food decreased both the rate and extent of tacrolimus absorption when administered to 15 healthy volunteers. The effect was most pronounced with a high mean AUC and C_{max} were decreased 37% and 77%, respectively; T_{max} was lengthened 5-fold [39].

A high-carbohydrate meal (668 kcal, 85% carbohydrate) decreased mean AUC and mean C_{max} by 28% and 65%, respectively. In healthy volunteers (N=16), the time of the meal also affected tacrolimus bioavailability. When given immediately following the meal, mean C_{max} was reduced 71%, and mean AUC was reduced 39%, relative to the fasted condition. When administered 1.5 hours following the meal, mean C_{max} was reduced 63%, and mean AUC was reduced 39%, relative to the fasted conditions. In 11 liver transplant subjects, tacrolimus capsules administered 15 minutes after a high-fat (400 kcal, 34% fat) breakfast, resulted in decreased AUC (27 ± 18%) and C_{max} (50 ± 19%), as compared to a fasted state [39].

The elimination half-life of tacrolimus is 18.8±16.7 hr in kidney transplant recipients [39]. It is therefore estimated that steady-state pharmacokinetics will be reached after 7 days of treatment. This was also confirmed in our most recent study [66] (a copy is included in the Appendix). Therefore, it is critical to tightly standardize the composition and timing of meals.

Subjects will be admitted for full 12 hour blood sampling. Subjects must be instructed to take the evening dose approximately 12 hours before the 12-hour PK profile. Upon arrival, the subject's diary and MEMS cap data will be reviewed. Protein saver cards will be collected and diary reviewed. Subject adherence will be assessed to determine eligibility criteria for study continuation have been met. Subjects should be fasting after midnight. Day 7 meals will be provided by the site and recorded on the CRF. Standardized meals will be provided after 3hr, 6hr and 12hr sampling times. Blood samples (3 mL) will be collected in EDTA-containing tubes at: C0 (before morning dose) and then 20, 40, 60(1hr), 80, 100, 120(2hr), 140, 160, 180(3hr) minutes, and 4, 5, 6, 8 and 12 hours after dosing.

Vital signs and clinical laboratory testing will be performed. All adverse events/serious adverse events, infections, concomitant medications/significant non-drug therapies, immunosuppressive medications, kidney allograft rejection, kidney allograft biopsy, graft loss and any changes and/or interruptions of study medication will be recorded on the CRF.

Upon completion of the 12 hour blood sampling, the subject will be dispensed the next study drug formulation in sequence and begin taking this formulation with the night time dose.

Visit 4, 5, 6, and 7 (Day 14, 21, 28, 35 after randomization): Subjects will be admitted for full 12-hour blood sampling. Subjects must be instructed to take the evening dose 12 hours before the 12-hour PK profile. Upon arrival, the subject's diary and MEMS cap data will be reviewed. Protein saver cards will be collected and diary reviewed. Subject adherence will be assessed to determine eligibility criteria for study continuation have been met. Subjects should be fasting after midnight. Day 7 meals will be provided by the site and recorded on the CRF. Standardized meals will be provided after 3hr, 6hr and 12hr sampling times. Blood samples (3 mL) will be collected in EDTA-containing tubes at: C0 (before morning dose) and then 20, 40, 60(1hr), 80, 100, 120(2hr), 140, 160, 180(3hr) minutes, 4, 5, 6, 8 and 12 hour after dosing.

Vital signs and clinical laboratory testing will be performed. All adverse events/serious adverse events, infections, concomitant medications/significant non-drug therapies, immunosuppressive medications, kidney allograft rejection, kidney allograft biopsy, graft loss and any changes and/or interruptions of study medication will be recorded on the CRF.

Upon completion of the 12 hour blood sampling, the subject will be dispensed the next study drug formulation in sequence and begin taking this formulation with the night time dose.

Visit 8 (End of Study visit, Day 42): Subjects will be admitted for full 12-hour blood sampling. Subjects will be instructed to take the evening dose 12 hours before the 12-hour PK profile. Upon arrival, the subject's diary and MEMS cap data will be reviewed. Protein saver cards will be collected and diary reviewed. Subject adherence will be assessed to determine eligibility criteria for study continuation have been met. Subjects should be fasting after midnight. Day 7 meals will be provided by the site and recorded on the CRF. Standardized meals will be provided after 3hr, 6hr and 12hr sampling times. Blood samples (3 mL) will be collected in EDTA-containing tubes at: : C0 (before morning dose) and then 20, 40, 60(1hr), 80, 100, 120(2hr), 140, 160, 180(3hr) minutes, 4, 5, 6, 8 and 12 hour after dosing.

Vital signs and clinical laboratory testing will be performed. All adverse events/serious adverse events, infections, concomitant medications/significant non-drug therapies, immunosuppressive medications, kidney allograft rejection, kidney allograft biopsy, graft loss and any changes and/or interruptions of study medication will be recorded on the CRF. Upon completion of the 12-hour blood sampling, the subject will be treated according to the physician's discretion with tacrolimus.

Subjects are instructed not to make any changes in the dose strength or frequency of any of the tacrolimus formulations during the trial, unless required for clinical reasons. Twice-daily dosing is required throughout each period of the study. Study subjects will be instructed to take their study medications at the same times each day, as closely as possible. This will be monitored *via* the MEMS caps and will statistically be analyzed.

Meals on study visits (Day 7, 14, 21, 28, 35, and 42) will be similar in content and amount. Any dose adjustments will be reported on the case report form.

As aforementioned, if for any reason, dose change, interruption or addition of any medication that affects tacrolimus drug levels is necessary during the study period, the subject will be withdrawn. The discontinued subject will be replaced by enrolling and randomizing a new subject. Full PK assessments will be performed on Day 7 (+2), Day 14 (±2), Day 21 (±2), Day 28 (±2), Day 35 (±2), and Day 42 (±2). Subjects will be instructed to take study medication under observation at the study visits after an overnight fast, and no food is allowed until 2 hours post-dose. Subjects will be switched at each visit to study medication based upon sequence assignment on a milligram for milligram basis. Table 11 lists all of the assessments.

Table 11. *Assessment schedule.*

**VISITS**	**Visit 1**	**Visit 2**	**Visit 3**	**Visit 4**	**Visit 5**	**Visit 6**	**Visit 7**	**Visit 8**
**Study Day**	Day -14	Day 1	Day 7+2	Day 14±2	Day 21±2	Day 28±2	Day 35±2	Day 42±2
**Activity**	Screening	Randomization	PK 1	PK 2	PK 3	PK 4	PK 5	PK 6/ EOS
Transplant background information	X							
Vital Signs[1]	X	X	X	X	X	X	X	X
Physical Exam[2]	X							X
Randomization[3]		X						
Hematology/ Chemistry/ Local tacrolimus level[4]	X	X	X	X	X	X	X	X
Genotype and MDR haplotype[5]	X							
Pregnancy Test[6]	X							
Tacrolimus 12hr PK[7]			X	X	X	X	X	X
Meal Records[8]			X	X	X	X	X	X
AEs/SAEs[9]		X	X	X	X	X	X	X
Infections		X	X	X	X	X	X	X
Concomitant Medications[10]	X	X	X	X	X	X	X	X
Immunosuppressive Medications	X	X	X	X	X	X	X	X
Kidney allograft rejection/biopsy[11]								
Graft loss[11]								
Dose Administration Records[12]			X	X	X	X	X	X
Drug Accountability and collection of protein saver cards[13]			X	X	X	X	X	X
MEMS cap assessement[14]			X	X	X	X	X	X
Study Completion/ Termination								X

[1]Vital signs include sitting blood pressure, weight, height (only at the screening visit) and temperature.
[2]Physical Exam: rectal, genital, and breast exam may be deferred unless clinically indicated.
[3]Randomization into one of six sequences will be consecutively assigned
[4]Hematology labs will include Hemoglobin, hematocrit, red blood cell count, white blood cell count with differential, and platelet count. Chemistry labs will include blood urea, creatinine, glucose, carbon dioxide, total bilirubin, AST, ALT, alkaline phosphatase, sodium, potassium, chloride, calcium, total protein, and albumin. Tacrolimus level will be performed locally only for safety purposes.
[5] Samples will be collected for MDR haplotype and genotyped for 3A5 expression.
[6]Urine or serum pregnancy test if positive urine test confirmed by serum BhCG.
[7]PK samples to be collected at the following time points: C0 (before morning dose) and then 20, 40, 60(1hr), 80, 100, 120(2hr), 140, 160, 180(3hr) minutes, 4, 5, 6, 8 and 12 hour after dosing. Actual samples collection times will be captured. ±3 minute window is allowed for samples prior to 4hr and ±5 minute window for samples after 4 hrs.

[8] Meals will be provided in a controlled fashion with a light breakfast after the 3hr blood sample, lunch after the 6hr blood sample, and dinner after the 12hr blood sample.
[9] All abnormal findings will be collected as adverse events. Any abnormal finding meeting the criteria for SAE will be reported via proper channels. Unblinding will be available at the discretions of the PI and Physician.
[10] All concomitant medications will be collected during the study. A list of prohibited medications is provided.
[11] Any kidney dysfunction episodes will result in a kidney biopsy at the discretion of the PI and Physician. Any liver dysfunction episodes will result in a liver biopsy at the discretion of the PI and Physician. Any biopsy data will be captured. No protocol biopsies will be performed.
[12] Study drug dose and administration time will be captured at each visit.
[13] Drug accountability will be performed by pill counts and patient diary review. Collection of daily trough blood samples via protein saver cards and documentation of time of collection will be reviewed in the patient diary. Eligibility of subsequent PK period will be assessed.
[14] MEMS cap data will be downloaded. Eligibility of subsequent PK period will be assessed.

18.1. Information to be collected on screening failures

If the subject fails to be randomized for any reason, the reason for not being randomized will be entered on the Screening Log.

18.1.1. Subject demographics/other baseline characteristics

Subject demographic and baseline characteristic data to be collected on all subjects include: date of birth, age, sex, race and ethnicity. Recipient and donor kidney and liver transplantation background information will be collected. Relevant medical history/current medical condition will also be collected at the time of study entry. Whenever possible, diagnoses and not symptoms will be recorded.

18.1.2. Treatment exposure and compliance

Medication containers must be returned at each visit, as compliance will be assessed by tablet counts, study diary, and MEMS data. All dose changes or interruptions will be recorded on the Dosage Administration Record CRF(s).

18.1.3. Efficacy

This is a pharmacokinetic comparison study, but efficacy will be measured by reported kidney or liver function or biopsy-proven acute rejection or graft loss as these are the standard efficacy parameters in transplant studies.

18.1.4. Safety

This is a pharmacokinetic comparison study, but safety data will be collected *via* adverse and serious adverse event reporting. Please see also Protection of Human Subjects for further detail.

18.1.5. Physical examination

A complete physical examination will include the examination of general appearance, skin, neck (including thyroid), eyes, ears, nose, throat, lungs, heart, abdomen, back, lymph nodes, and extremities, vascular and neurological. If indicated based on medical history and/or symptoms, rectal, external genitalia, breast, and pelvic exams will be performed at screening visit.

Information for all physical examinations must be included in the source documentation at the study site. Significant findings that are present prior to the start of study drug must be included in the Relevant Medical History/Current Medical Conditions CRF. Significant findings made after the start of study drug which meet the definition of an Adverse Event (see Protection of Human Subject section) must be recorded on the Adverse Event/Infection CRF.

18.1.6. Vital signs

Vital signs include blood pressure and pulse measurements. After the subject has been sitting for five minutes, with back supported and both feet placed on the floor, systolic and diastolic blood pressure will be measured using an automated validated device with an appropriately sized cuff. Vital signs will be measured at the beginning and end of each visit.

18.1.7. Height and weight

Height in centimeters (cm) and body weight (to the nearest 0.1 kilogram [kg] in indoor clothing, but without shoes) will be measured. Body weight will be measured at the beginning of each visit, and height will only be measured at Visit 1.

18.2. Laboratory evaluations

Tacrolimus blood concentrations (daily protein saver and multiple PK sample venipuncture) will be analyzed using a validated LC-MS/MS method at a central laboratory (iC42 Clinical Research and Development, University of Colorado, *vide infra*). Trough tacrolimus blood concentrations for safety at baseline and each PK visit will be analyzed locally by immunoassay, but not be used in the pharmacokinetic analyses.
Analysis of other safety laboratory will be performed at the same local laboratory.

18.2.1. Hematology

Hemoglobin, hematocrit, red blood cell count, white blood cell count with differential, and platelet count will be measured.

18.2.2. Clinical chemistry

Blood urea, creatinine, glucose, carbon dioxide, total bilirubin, AST, ALT, alkaline phosphatase, sodium, potassium, chloride, calcium, total protein, and albumin will be measured.

18.2.3. Urinalysis

Not required.

18.2.4. Tacrolimus Pharmacogenetic Testing

Samples will be collected at baseline for MDR1/ABCB1 haplotyping and cytochrome P4503A5 genotyping as well as genotyping of other relevant SNPs (Table 13, *vide infra*).

18.2.5. Pregnancy and assessments of fertility

All pre-menopausal women who are not surgically sterile will have a urine or serum pregnancy test. A positive urine pregnancy test requires immediate interruption of study drug until serum BhCG is performed and found to be negative. If positive, the subject must not be enrolled in the trial or may be withdrawn.

18.2.6. Appropriateness of safety measurements

All safety assessments performed during this trial are standard and widely used and generally recognized as reliable, accurate and relevant.

18.3. Other assessments

18.3.1. Blood samples for pharmacokinetic evaluation

Tacrolimus blood concentration produced by the administered formulations will be processed in order to establish their pharmacokinetic profiles. All blood samples will be taken by either direct venipuncture or an indwelling cannula inserted in a forearm vein at the time points specified in Table 12. At each time point, a 3 mL blood sample will be collected into pre-cooled vacutainers. Blood samples will be collected in one 3ml EDTA-tube for each timed PK sample. The exact sample collection time will be recorded in the case report form. All deviations from the scheduled sampling time of 3 minutes or more for the first 2 hours (predose–3 hr), and 5 minutes or more for all remaining samples (4 hr–12 hr) will be reported as a protocol deviation.

Immediately after each tube of blood is drawn, it will be inverted gently several times to ensure the mixing of tube contents (e.g., anticoagulant). Blood will be separated into duplicate polypropylene culture tubes and frozen in an upright position at -80°C until sent on dry ice to iC42 Clinical Research and Development (University of Colorado) for LC-MS/MS analysis. The assay and its performance specifications are described in more detail below.

The exact clock time of dosing, as well as actual sample collection date and time will be entered on the PK blood collection summary page of the CRFs. Sampling problems will be noted in the Comments section of the CRFs.

18.4. Payment for Participation

Participants will be paid $3000 if they complete the study - $500 for each of the six Pharmacokinetic visits. Those that do not complete the study will be paid for the portion of the visits that they complete.

Table 12. *Pharmacokinetics and Safety Laboratory Sample Log.*

		Time (hrs and min)	Time (hrs)	PK Blood Samples (whole blood and waste)			Safety Lab Genetic testing (serum & whole blood)
Day				PK Collection No.	Sample No.	Volume (mL)	Volume (mL)
Day -14							25
Day 1							20
Day 7	Pre-dose	C0 (before morning dose)	0hr	1	1	3	20
	Post-dose	20 min		1	2	3	
		40 min		1	3	3	
		60 min	1hr	1	4	3	
		80 min		1	5	3	
		100 min		1	6	3	
		120 min	2hr	1	7	3	
		140 min		1	8	3	
		160 min		1	9	3	
		180 min	3hr	1	10	3	
		4hr	4hr	1	11	3	
		5hr	5hr	1	12	3	
		6hr	6hr	1	13	3	
		8hr	8hr	1	14	3	
		12hr	12hr	1	15	3	
Day 14	Pre-dose	C0 (before morning dose)	0hr	1	16	3	20
	Post-dose	20 min		1	17	3	
		40 min		1	18	3	
		60 min	1hr	1	19	3	
		80 min		1	20	3	
		100 min		1	21	3	
		120 min	2hr	1	22	3	
		140 min		1	23	3	
		160 min		1	24	3	
		180 min	3hr	1	25	3	
		4hr	4hr	1	26	3	
		5hr	5hr	1	27	3	
		6hr	6hr	1	28	3	
		8hr	8hr	1	29	3	
		12hr	12hr	1	30	3	
Day 21	Pre-dose	C0 (before morning dose)	0hr	1	31	3	20

	Post-dose	20 min		1	32	3	
		40 min		1	33	3	
		60 min	1hr	1	34	3	
		80 min		1	35	3	
		100 min		1	36	3	
		120 min	2hr	1	37	3	
		140 min		1	38	3	
		160 min		1	39	3	
		180 min	3hr	1	40	3	
		4hr	4hr	1	41	3	
		5hr	5hr	1	42	3	
		6hr	6hr	1	43	3	
		8hr	8hr	1	44	3	
		12hr	12hr	1	45	3	

		Time (hrs and min)	Time (hrs)	PK Blood Samples (whole blood and waste)	Safety Lab Genetic testing (serum & whole blood)		
Day 28	Pre-dose	C0 (before morning dose)	0hr	1	46	3	20
	Post-dose	20 min		1	47	3	
		40 min		1	48	3	
		60 min	1hr	1	49	3	
		80 min		1	50	3	
		100 min		1	51	3	
		120 min	2hr	1	52	3	
		140 min		1	53	3	
		160 min		1	54	3	
		180 min	3hr	1	55	3	
		4hr	4hr	1	56	3	
		5hr	5hr	1	57	3	
		6hr	6hr	1	58	3	
		8hr	8hr	1	59	3	
		12hr	12hr	1	60	3	
Day 35	Pre-dose	C0 (before morning dose)	0hr	1	61	3	20
	Post-dose	20 min		1	62	3	
		40 min		1	63	3	
		60 min	1hr	1	64	3	
		80 min		1	65	3	
		100 min		1	66	3	
		120 min	2hr	1	67	3	
		140 min		1	68	3	
		160 min		1	69	3	
		180 min	3hr	1	70	3	
		4hr	4hr	1	71	3	
		5hr	5hr	1	72	3	
		6hr	6hr	1	73	3	
		8hr	8hr	1	74	3	
		12hr	12hr	1	75	3	
Day 42	Pre-dose	C0 (before morning dose)	0hr	1	76	3	20
	Post-dose	20 min		1	77	3	
		40 min		1	78	3	
		60 min	1hr	1	79	3	
		80 min		1	80	3	
		100 min		1	81	3	

		120 min	2hr	1	82	3	
		140 min		1	83	3	
		160 min		1	84	3	
		180 min	3hr	1	85	3	
		4hr	4hr	1	86	3	
		5hr	5hr	1	87	3	
		6hr	6hr	1	88	3	
		8hr	8hr	1	89	3	
		12hr	12hr	1	90	3	
Totals						270	165
**Total Blood Volume**							**435**

(Table 12 continued)

19. Protection of Human Subjects

This clinical study was designed and shall be implemented and reported in accordance with the ICH Harmonized Tripartite Guidelines for Good Clinical Practice, with applicable local regulations (including European Directive 2001/20/EC, US Code of Federal Regulations Title 21, and Japanese Ministry of Health, Labor, and Welfare),and with the ethical principles laid down in the Declaration of Helsinki. Eligible patients may only be included in the study after providing written (witnessed, where required by law or regulation), IRB/IEC-approved informed consent, or, if incapable of doing so, after such consent has been provided by a legally acceptable representative of the subject. In cases where the subject's representative gives consent, the subject should be informed about the study to the extent possible given his/her understanding. If the subject is capable of doing so, he/she should indicate assent by personally signing and dating the written informed consent document or a separate assent form. Informed consent must be obtained before conducting any study-specific procedures (i.e. all of the procedures described in the protocol). The process of obtaining informed consent should be documented in the subject source documents. The informed consent form will comply with the ICH GCP guidelines and regulatory requirements and is considered appropriate for this study. Women of child bearing potential should be informed that taking tacrolimus may involve unknown risks to the fetus if pregnancy were to occur during the study and agree that in order to participate in the study they must adhere to the contraception requirement for the duration of the study. If there is any question that the subject will not reliably comply, they should not be entered in the study.

The protocol and the proposed informed consent form must be reviewed and approved by a properly constituted Institutional Review Board/Independent Ethics Committee/Research Ethics Board (IRB/IEC) before study start. Prior to study start, the investigator is required to sign a protocol signature page confirming his/her agreement to conduct the study in accordance with these documents and all of the instructions and procedures found in this protocol and to give access to all relevant data and records to monitors, auditors, IRBs/IECs/REBs, and regulatory authorities as required.

No change of study procedures will be initiated before an amendment has been approved and, if applicable, appropriate written informed re-consent has been obtained.

Patients participating in another research study may be eligible for inclusion. In such a case, the total amounts of blood drawn during both studies need to be within tolerable limits. The total amount of blood drawn during this study including for pharmacokinetics samples, genetic testing and safety laboratory evaluation will be 435 mL over 56 days, 85 mL hereof will be drawn during the first 2 weeks. For details please see Table 12 PK in section 5.6.1 Blood samples for pharmacokinetic evaluation.

Confidentiality of the identity of all subjects will be maintained. A unique identification number will be assigned to each subject upon entry to the study and will be used to identify the subject for study's duration. Only initials and numbers will be used on case report forms and in all study correspondence.

Data will exclusively be managed using the REDCap software in full compliance with all applicable rules and policies. All data transfers, management and handling will be in compliance with HIPAA regulations.

A description of this clinical trial will be available on http://www.ClinicalTrials.gov as required by US Law. This website will not identify the subjects. At most the Web site will include a summary of the results.

19.1. Potential Health Risks

All patients will receive the usual standard of care. Tacrolimus will be dosed targeting the usual trough blood concentrations. No other changes except repeatedly switching the patient between bioequivalent, FDA-approved tacrolimus formulations will be made. An additional risk arises from the more frequent blood draws and the associated blood loss and complications such as bruising and infections. In regards to formulation switching, transplant recipients, whether study participants or not, will likely be switched to a generic formulation of tacrolimus at some time post-transplant considering that generic formulations have over 50% market share. The physical risk of acute rejection related to changes in tacrolimus formulation have not been reported in published retrospective studies. The risk of conversion to different formulations in this study is minimized by weekly tacrolimus level and organ function markers (i.e. SrCr or LFTs) as compared to conversions that occur outside of the study environment which may not accompany these additional tests.

These patients typically do not have a psychological aversion to blood draws due to their previous medical histories. However, venipuncture will be minimized by placement of a venous catheter in the arm.

Over the past 10 years, we have enrolled over 75 patients in similar trials with no serious adverse events occurring secondary to study participation. By nature of this study, these patients are stable and are at no greater risk of adverse event than at any other time. All study personnel are trained in basic life support and a nurse is available to evaluate any medical emergency. Our study facilities are <5 miles from the transplant hospital. If an emergency were to arise, 911 would be called immediately. We have transplant surgery fellows on call 24/7 in whom we can call to evaluate in person for any non-urgent medical issues.

There are no other FDA approved treatment alternatives to tacrolimus in stable renal or liver transplant recipients. Other potential regimens are employed when transplant recipients are experiencing adverse events, i.e. belatacept, sirolimus or everolimus for renal dysfunction post-transplant.

19.2. Adverse Event Monitoring

19.2.1. Adverse events

An adverse event is the appearance or worsening of any undesirable sign, symptom, or medical condition occurring after starting the study drug even if the event is not considered to be related to study drug. Study drug includes the investigational drug under evaluation and the comparator drug or placebo that is given during any phase of the study. Medical conditions/diseases present before starting study drug are only considered adverse events if they worsen after starting study drug. Abnormal laboratory values or test results constitute adverse events only if they induce clinical signs or symptoms, are considered clinically significant, or require therapy.

The occurrence of adverse events should be sought by non-directive questioning of the subject at each visit during the study. Adverse events also may be detected when they are volunteered by the subject during or between visits or through physical examination, laboratory test, or other assessments. All adverse events must be recorded on the Adverse Event/Infection CRF with the following information:

1. the severity grade [mild, moderate, severe]
2. its relationship to the study drug(s) (suspected/not suspected)
3. its duration (start and end dates or if continuing at final exam)
4. whether it constitutes a serious adverse event (SAE)

An SAE is defined as an event which:

- is fatal or life-threatening
- results in persistent or significant disability/incapacity
- constitutes a congenital anomaly/birth defects

- requires in subject hospitalization or prolongation of existing hospitalization, unless hospitalization is for:
- routine treatment or monitoring of the studied indication, not associated with any deterioration in condition
- elective or pre-planned treatment for a pre-existing condition that is unrelated to the indication under study and has not worsened since the start of study drug
- treatment on an emergency out subject basis for an event not fulfilling any of the definitions of a SAE given above and not resulting in hospital admission
- social reasons and respite care in the absence of any deterioration in the subject's general condition
- is medically significant, i.e. defined as an event that jeopardizes the subject or may require medical or surgical intervention to prevent one of the outcomes listed above

Unlike routine safety assessments, SAEs are monitored continuously and have special reporting requirements (*vide infra*).

All adverse events will be treated appropriately. Treatment may include one or more of the following: no action taken (i.e. further observation only); study drug dosage adjusted/temporarily interrupted; study drug permanently discontinued due to this adverse event; concomitant medication given; non-drug therapy given; subject hospitalized/subject's hospitalization prolonged. The action taken to treat the adverse event should be recorded on the Adverse Event/Infection CRF.

Once an adverse event is detected, it will be followed until its resolution or until it is judged to be permanent, and assessment should be made at each visit (or more frequently, if necessary) of any changes in severity, the suspected relationship to the study drug, the interventions required to treat it, and the outcome.
Information about common side effects already known about the investigational drug can be found in the tacrolimus package insert. This information will be included in the subject informed consent and will be discussed with the subject before and during the study as appropriate.

19.2.2. Serious adverse event reporting

To ensure subject safety, every SAE, regardless of suspected causality, occurring after the subject has provided informed consent and until 30 days after the subject has stopped study participation (defined as time of last dose of study drug taken or last visit whichever is later) must be reported to the principal investigator within 24 hours.

Recurrent episodes, complications, or progression of the initial SAE must be reported as follow-up to the original episode, regardless of when the event occurs. This report must be submitted within 24 hours of the investigator receiving the follow-up information. An SAE that is considered completely unrelated to a previously reported one should be reported separately as a new event.

Information about all SAEs is collected and recorded on the Serious Adverse Event Report Form. The investigator must assess the relationship of any SAE to study drug.

If the SAE is not previously documented in the package insert (new occurrence) and is thought to be related to the study drug, it will be reported to the FDA. Suspected Unexpected Serious Adverse Reactions (SUSARs) will be collected and reported to the competent authorities and relevant ethics committees.

19.2.3. Pregnancies

To ensure subject safety, each pregnancy in a subject on study drug must be reported to the principal investigator within 24 hours of learning of its occurrence. The pregnancy should be followed up to determine outcome, including spontaneous or voluntary termination, details of the birth, and the presence or absence of any birth defects, congenital abnormalities, or maternal and/or newborn complications.

19.2.4. Data and Safety Monitoring Committee

Since patients are switched between bioequivalent tacrolimus formulations, all of which are approved in the United States, a data and safety monitoring committee is deemed unnecessary.

19.3. Adequacy of Protection against Risks

Recruitment and Informed Consent

Before enrollment in the study, prospective patients and, if applicable, their guardians will have the study explained by a member of the research team or an appropriately qualified member of their staff. The purpose of the study will be reviewed, and the potential risks and discomforts of study participation explained. A certified, medical interpreter will be provided for subjects with limited knowledge of the English language. Subjects and/or their guardians are given a copy of the consent form to read. If they verbally agree to participate in the study, written informed consent/assent is obtained after the patient and/or their guardians have read the consent and understand the study. The consent process is documented on the clinical reporting forms. Patients and their parents/ guardians are asked to sign a HIPAA Authorization B Form, which informs them what protected health information is collected on them and who will have access to such information. Subjects receive copies of all signed forms. Please also see the compliance statements in section 5.

Protection against Risk and Safety Monitoring Plan

Patients will continue to be monitored by their physicians. Adequacy and protection against risks of the proposed studies will have been reviewed by the University of Cincinnati IRB.

19.4. Potential Benefits of the Proposed Research to Subjects and Others

There is no known direct benefit for participation in this study, except the stipend provided to these patients. However, many of these patients come from great distances and each PK participation period may take up 2-3 days when travel and lodging are included. Many are also accompanied by a spouse or friend. The benefit for others is the generation of knowledge that will result in a better understanding generic immunosuppressant drugs, their pharmacokinetic bioequivalence, their safety and their switchability.

19.5. Importance of Knowledge to be Gained

Our study is designed to answer the following important questions:

- Are generic immunosuppressants for which bioequivalence was established in single-dose healthy volunteer studies also bioequivalent to the brand in stable transplant patients under steady-state conditions?
- Will generic tacrolimus be bioequivalent independent of the transplant type (kidney or liver)?
- Will generic tacrolimus be bioequivalent to each other even if the most disparate generic formulations currently approved in the United States as determined in *in vitro* dissolution studies are compared?
- Will the brand and tacrolimus generics as well as tacrolimus generics tested be bioequivalent with each other even in patients who are known expressors of cytochrome P4503A5 and/or high activity of the efflux transporter p-glycoprotein (based on MDR-1 haplotype analysis), which are known to be “poor absorbers” of tacrolimus?
- Will there be a difference in the metabolism of tacrolimus among the three formulations in the study population as well as in the subgroups?
- Will alternative bioequivalence metrics such as using narrower acceptance intervals, scaled average bioequivalence and a population pharmacokinetic approach also confirm bioequivalence between the brand and the two generics among each other in the complete study population as well as in the above-mentioned subgroups?

19.6. Data Safety and Monitoring Plan

This study will be carried out in full compliance with the rules of Good Clinical Practice (GCP), as described in the following documents:

- ICH Harmonized Tripartite Guidelines for Good Clinical Practice, 1996
- UC Code of Federal Regulations dealing with clinical studies (21 CFR including parts 50 and 56)
- The principles stated in the Declaration of Helsinki (V11 Oct, 2000) concerning medical research in humans (adopted by the 18th World Medical Assembly, Helsinki 1964, amended Tokyo 1975, Venice

1983, Hong Kong 1989 and Somerset West, Republic of South America 1996, Edinburgh 2000; Note of Clarification on Paragraph 29 added 2002)

19.7. Inclusion of Women and Minorities

There will be no recruitment restrictions regarding gender or ethnic background. There are no recruitment restrictions beyond the inclusion/exclusion criteria (see Approach section for inclusion/exclusion criteria). There are no restrictions regarding inclusion of minorities. Children and pregnant women will be excluded due to potential hormonal differences that may impact tacrolimus metabolic pathways. Prisoners will be excluded due to the amount of time required to participate in study activities. The enrollment tables below reflect the population demographics in the State of Ohio. Due to the nature and the goals of the study, only patients ≥ 18 years of age will be enrolled.

Total Planned Enrollment: At least 38 STABLE RENAL TRANSPLANT PATIENTS

**TARGETED/PLANNED ENROLLMENT: Number of Subjects**			
**Ethnic Category**	**Sex/Gender**		
	**Females**	**Males**	**Total**
Hispanic or Latino	0	0	0
Not Hispanic or Latino	14	24	38
Ethnic Category Total of All Subjects*	14	24	38
**Racial Categories**			
American Indian/Alaska Native	0	0	0
Asian	0	0	0
Native Hawaiian or Other Pacific Islander	0	0	0
Black or African American	3	5	8
White	10	20	30
Racial Categories: Total of All Subjects *	13	25	38

Total Planned Enrollment: At least 38 STABLE LIVER TRANSPLANT PATIENTS

TARGETED/PLANNED ENROLLMENT: Number of Subjects			
**Ethnic Category**	**Sex/Gender**		
	**Females**	**Males**	**Total**
Hispanic or Latino	0	0	0
Not Hispanic or Latino	14	24	38
Ethnic Category Total of All Subjects*	14	24	38
**Racial Categories**			
American Indian/Alaska Native	0	0	0
Asian	0	0	0
Native Hawaiian or Other Pacific Islander	0	0	0
Black or African American	1	3	4
White	13	21	34
Racial Categories: Total of All Subjects *	14	24	38

20. Tacrolimus pharmacogenetics

As already mentioned above, seven functional single nucleotide polymorphisms (SNPs) are selected in the genes of drug metabolizing enzymes CYP3A4/5, P450 oxidoreductase and drug transporter ABCB1/MDR1. The selected SNPs will be genotyped by a real-time TaqMan PCR with an appropriate variation step by direct sequencing. Eleven SNPs in the genes of nuclear factors, PXR, CAR and HNF4a, are also selected for screening purpose as they are potentially influencing factors on CYP3A activity since they regulate CYP3A expressions (priority level 2). These SNPs will also be genotyped by real-time TaqMan PCR. Additionally, eight minor SNPs (priority level 3) are selected for exploring influencing SNPs. These SNPs will be assessed by direct sequencing for patients who show extreme discordant tacrolimus concentrations.

Table 13. *Initially targeted SNPs*

Protein	Priority	Allele name	SNP location	SNP	dbSNP NCBI	Note (Phenotype)	Ref
**CYP3A4** #1	1	**CYP3A4*1B**	**Promoter**	**(-392)A>G**	**rs2740574**	Showed trend for tacrolimus	[86]
	3	**CYP3A4*4**	**Exon 4**	**13871A>G**	**NA**	Ile118Val	
	3	**CYP3A4*16**	**Exon 7**	**15603C>G**	**rs12721627**	Thr185Ser (found in Japanese)	[87,88]
	3	**CYP3A4*18**	**Exon 10**	**20070T>C**	**rs28371759**	Leu293Pro	[87]
	1	**CYP3A4*22**	**Intron 6**	**15389C>T**	**rs35599367**	Affects the expression and activity of CYP3A4	[89,90]
**CYP3A5** #2	3	**CYP3A5*2**	**Exon 11**	**27286C>A**	**rs28365083**	Thr398Asn	
	1	**CYP3A5*3**	**Intron 3**	**6986A>G**	**rs776746**	CYP3A5 non-expresser /significant influence on tacrolimus PK	[86, 91,92]

	**3**	**CYP3A5*4**	**Exon 7**	**14665A>G**	**NA**	Gln200Arg	
	**3**	**CYP3A5*5**	**Intron 5**	**12952T>C**	**NA**	CYP3A5 non-expresser	
	**3**	**CYP3A5*6**	**Exon 7**	**14690G>A**	**NA**	CYP3A5 non-expresser	
	**3**	**CYP3A5*7**	**Exon 11**	**27131 insT**	**rs41303343**	CYP3A5 non-expresser	
	**2**	**CYP3AP1*3**	**5'-UTR**	**(-44)G>A**	**NA**	CYP3A5 non-expresser	
**MDR1**	**3**		**Promoter**	**(-129)T>C**	**rs3213619**	-	
	**1**		**Exon 12**	**1236C>T**	**rs1128503**	-	[93]
	**1**		**Exon 21**	**2677G>T, A**	**rs2032582**	Ala893Ser, Thr	[93]
	**1**		**Exon 26**	**3435C>T**	**rs1045642**	-	[93]
	**3**			**3587T>G**	**NA**	I1196S、Loss of function	[94]
**P450 oxidoreductase** #3	**1**	**POR*28**		**1590C>T**	**rs1057868**	Ala503Val / effect on Tacrolimus PK was reported	[95]
**NR1|2 (PXR)**	**2**			**(-25385)C>T**	**rs3814055**		[96, 97]
	**2**			**(-25931)T>C**	**rs1523130**		[97]
	**2**			**(-6994)C>T**	**rs2472677**		[97, 98]
	**2**			**(-12202)T>C**	**rs13085558**		[97]
	**2**			**(-1650)T>A**	**rs2472679**		[97]
	**2**	***1B**			**rs2276707**		[99]
	**2**	***1B**			**rs3814058**		[99]
**NR1|3 (CAR)**	**2**			**5719C>T**	**rs2307424**		[100]
	**2**			**7738A>C**	**rs2307418**		[100]
	**2**			**7837T>G**	**rs4073054**		[100]
**HNF4a**	**2**				**rs2071197**		[101-103]

Study Sample Analysis Using a Validated Tacrolimus LC-MS/MS Assay for the Quantification of Tacrolimus.

Tacrolimus samples will be shipped to iC42 Clinical Research & Development (University of Colorado). iC42 Clinical Research and Development is a state-of-the-art mass spectrometry facility with 14 LC-MS/MS systems that was specifically established by the University of Colorado in 2001 as a laboratory that carries out pre-clinical drug development and clinical bioanalytics and development. United States and international bioequivalence guidances stipulate that samples from bioequivalence studies have to be analyzed in a regulatory compliant laboratory environment [30, 107] using an appropriately validated assays [108].

iC42 Clinical Research Regulatory Compliance, Certifications and Licenses.
To comply with regulatory standards and following the FDA “fit-for-purpose” principle all analytical procedures are and will be validated following generally accepted guidances and standards such as those set forth by FDA [108], CLSI [109] and EMEA [110]. All procedures are documented in the laboratory’s Quality Assurance Manual and standard operation procedures. For a detailed evaluation, please see the Resources section.

Bioanalytics for Clinical Trials. iC42 Clinical Research and Development labroatory is accredited by the College of American Pathologists (CAP, LAP number 2182803, AU-ID 118674) and is CLIA certified (06D0985306). The last CAP inspection was in October 2010 and the current CAP certificate is vaild until December 3, 2012.

cGLP Compliance. iC42 Clinical Research and Development has successfully conducted analyses for a multitude of FDA (21 CFR part 58) and OECD cGLP compliant studies and accordingly is audited by sponsors and their consultants on a regular basis. iC42 Clinical Research and Development has never received a

warning letter as consequence of a regulatory inspection. A quality and compliance self-evaluation and a list of all current standard operation procedures can be found in the Appendix.

Quality Assurance. The iC42 Clinical Research and Development Quality Assurance Unit conducts regular audits consisting of in-process, systems, data, documents, standard operations procedure and facility audits, for the duration of the task/study. The quality assurance unit is managed by Jelena Klawitter, PhD, MRQA (Assistant Professor, Quality Assurance Manager). Dr. Klawitter is a chemist and has extensive experience in drug metabolism, bioanalysis including assay development and validation. Overall, she has more than 7 years of experience in relevant work procedures and technologies. Dr. Klawitter was trained in research quality assurance by the British Association of Research Quality Assurance (BARQA) holds the title of Master of Research Quality Assurance (MRQA). She ensures integrity, correctness, completeness and/or regulatory compliance of the data, facilities, documentation, archivation, personnel qualification, study planning, assay development, quality control during study sample analysis, sample tracking, preventive maintenance of equipment, qualification of analytical equipment and data transfer, training of staff, and reporting.

Tacrolimus LC-MS/MS Assay.
iC42 Clinical Research and Development's laboratory director, Dr. Christians, has more than 20 years of experience with the quantification of tacrolimus and its metabolites in the blood of transplant patients. In fact, Dr. Christians and his group were the first to publish a tacrolimus LC-MS assay [68,111]. The assay is based on the principles published in reference [112]. In brief:
One hundred µL EDTA whole blood and 400 µL of a methanol/ 0.4 M ZnSO4, 4/1 v/v protein precipitation solution that also contains the internal standard tacrolimus -$^{13}C,D_2$ (Toronto Research Chemicals, North York, ON) resulting in a final concentration of 5 ng/mL. After vortex mixing (2.5 min) and centrifugation (5 minutes at 13,000*g* at 4º C), 100 µL of the supernatant is injected into the HPLC-MS/MS system for online extraction and analysis. For details in terms of the online extraction and the connections of online extraction and analytical HPLC column, please see reference [112]. Dried blood spot samples (DBS) will be punched out of the filter cards and pretreated as described before [113,114]. Thereafter, these samples will be extracted and analyzed using the same extraction and LC-MS/MS procedures as described for whole blood EDTA samples
The LC-MS/MS systems used and validated for tacrolimus analysis consists of Agilent 1100, 1200, or 1260 components (Agilent, Santa Clara, CA) in combination with AB Sciex API4000 or API5000 (ABSciex, Foster City, CA) mass spectrometers: HPLC I: binary pump, degasser and LEAP auto sampler equipped with cooling stack; HPLC II: binary pump, degasser, column thermostat and electrospray MS/MS system. The two HPLC systems are connected *via* a 7240 Rheodyne 6-port switching valve mounted on a step motor (Rheodyne, Cotati, CA). The system is controlled and data are processed using the Applied Biosystems Analyst software.

One hundred µL of the samples are injected onto a 10x2 mm extraction column (Zorbax XDB C8, Agilent Technologies, Palo Alto, CA) filled with Zorbax XDB C8 material. Samples are washed with a mobile phase of 20% methanol and 80% 0.1% formic acid. The flow is 5 ml/min and the temperature for the extraction column is set to 65 °C. After 1 min, the switching valve is activated and the analytes are eluted in the back flush mode from the extraction column onto a 150x4.6 mm C_8, 5µm analytical column (Zorbax XDB C8, Agilent Technologies, Palo Alto, CA). The mobile phase consists of methanol and 0.1% formic acid. The following gradient is run: time 0-2 min: from 87% methanol to 100% methanol, 2-3.5 min: 100 % methanol, 3.6-5 min: 87% methanol. The flow rate is 1 mL/ min. The analytical column is also kept at 65°C. Tacrolimus and its internal standard are detected in the positive multi-reaction mode (MRM).

The assay has the following key performance parameters (ABSciex API4000):
- lower limit of quantitation; 0.25 ng/mL,
- range of reliable response: 0.25- 100 ng/mL
- intra-day imprecision: 0.25 ng/mL: 12.3%, 1 ng/mL: 3.7%, 25 ng/mL: 1.5%, 100 ng/mL: 15.8%
- inter-day imprecision: 0.75 ng/mL: 7.1%, 5 ng/mL: 4.4%, 20 ng/mL: 1.7%, 70 ng/mL: 1.6%.
- inter-day accuracy: 0.75 ng/mL: +3.7%, 5 ng/mL: -3.4%, 20 ng/mL: +8.6%, 70 ng/mL: +3.6%.
- matrix interferences: none
- matrix effects: no significant effects detected (post-column infusion and the "Matuszewski" method [115])
- carry-over: none

- autosampler stability: at least 48 hours at +4°C
- freeze-thaw cycles: at least 3 cycles
- long-term stability at -80°C: at least 1 year.

If the project is awarded, the assay will be re-validated prior to sample study analysis. A validation report will be generated and no study samples will be analyzed without approval by Dr. Alloway, and if requested, FDA program staff involved in this project.

As the iC42 Clinical Research and Development’s tacrolimus assay is also used for clinical therapeutic drug monitoring, the assay has regularly participated in two proficiency testing programs over the last decade; the College of American Pathologists and the UK NEQAS/UKAS proficiency program initiated by Prof. D.W. Holt and now managed by LGC (Lancashire, UK). Two most recent, representative results of the latter proficiency testing program that is distributed on a monthly basis for iC42’s tacrolimus assay is shown in Figure 7.

Figure 7. *Most recent NEQAS/UKAS proficiency testing results of iC42’s tacrolimus assay.*

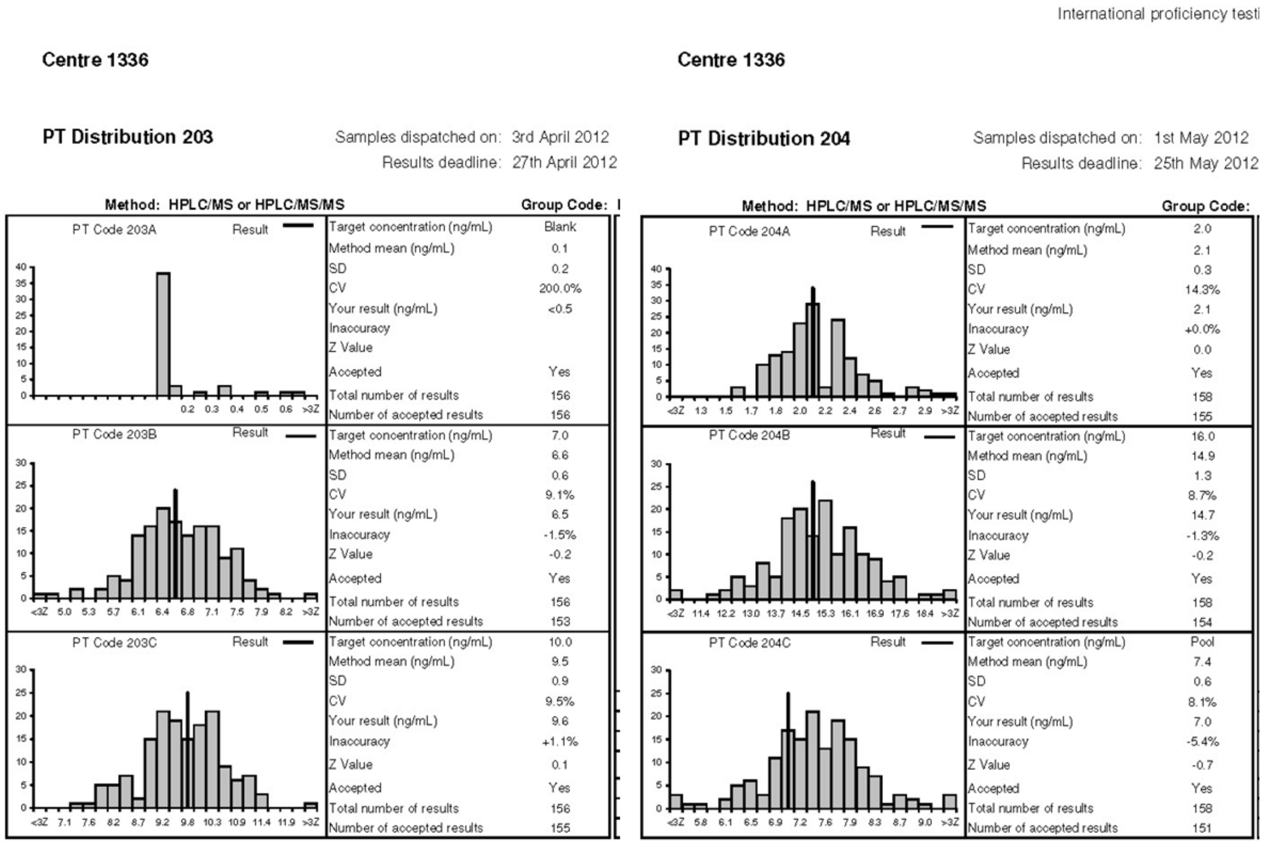

Study sample analysis

Sample analysis will follow the following sequence:

- system suitability testing
- calibration curve: blank and zero, 0.25 0.5, 1, 2.5, 5, 10, 25, and 50 ng/mL
- - QCs: 0.75; 7.5, 12.5 and 37.5 ng/mL
- - Blanks: 1 every 15 samples
- - Blanks for carry-over control: at the beginning of each batch and following the highest concentrations of the standard curve (50 ng/mL).
-

Not more than 100 study samples will be measured between calibration curves. Quality control and long-term monitoring of the assay as well as, if necessary, corrective action will follow all applicable iC42 Clinical Research and Development standard operation procedures.

Manual re-integration. Manual integration will be allowed to minimize errors made by the integration software, which especially may occur in the case of blanks and low-concentration samples, when integration software

tends to include obvious baseline noise into the integrated peak. However, it will be critical to avoid investigator/ analyst bias. Therefore, manual integration and its documentation will strictly follow the rules, procedures, checks and balances as set forth in iC42 Clinical Research and Development standard operation procedure CR-WP-303 “Manual Integration of Chromatograms” and will be closely reviewed by iC42 Clinical Research and Development Quality Assurance. Any manual integration of ion chromatograms will be justified in writing (with signature and date) and listed together with values from the automatic integration [30].

Incurred Sample Analysis. Ten % of the study samples will be re-analyzed. Incurred sample analysis will follow the predefined rules, procedures and criteria as set forth in iC42 Clinical Research and Development standard operation procedure CR-GE-013 “Incurred Sample Re-Analysis” in its most recent version.

Tacrolimus Metabolites

As aforementioned, we will also quantify the major tacrolimus metabolites. Dr. Christians’ group was the first to describe the tacrolimus metabolites and their structures [115-118] and to describe the tacrolimus metabolite patterns in transplant patients [68-70,76]. iC42 Clinical Research and Development has established procedures to generate all major tacrolimus metabolites using human liver microsomes, over-expressed human cytochrome P4503A4 enzymes (Puracyp, San Diego, CA) and actinomyces strains, subsequent isolation of the metabolites using preparative HPLC, confirming the structures using ion trap MS^n in combination of the analysis of fragmentation patterns, establishing purity using HPLC-UV-ion trap mass spectrometry and quantification using HPLC-UV-ion trap mass spectrometry. These purified metabolites have been made available to manufacturers of tacrolimus therapeutic drug monitoring assays for assay development and validation purposes and, most importantly, to establish cross reactivity of new immunoassay antibody batches with purified metabolites. To the best of our knowledge, iC42 Clinical Research and Development is currently the only source for the complete set of tacrolimus metabolites. The following authentic isolated metabolites are available (all >99% % free of other tacrolimus derivatives):

- 13-O-desmethyl tacrolimus
- 15-O-desmethyl tacrolimus
- 31-O-desmethy tacrolimus
- 12-hydroxy tacrolimus
- 13,31-di-O-desmethyl tacrolimus
- 13,15-di-O-desmethyl tacrolimus

For each of the metabolite preparations, iC42 Clinical Research and Development generates and archives a report that contains all relevant information in terms of methods, purity, quantification and structural identification. Hardcopies of the original results (MS/MS spectra for structural identification, NMR spectra, chromatograms) are included in these reports as an appendix.

After tacrolimus has been quantified using the validated assay above, tacrolimus metabolites will be quantified using a modified version of the assay described by us in reference [76]. The major difference will be that the assay will be run using a tandem mass spectrometer and that MRMs of the metabolites will be detected and used for quantification. The rationale for running the samples twice is that the quantitative tacrolimus assay is better controlled and validated than the multi-analyte assay measuring the metabolites. As tacrolimus concentrations are the primary outcome parameter, the quality of these measurements should not be compromised by simultaneously measuring the metabolites. The challenges with the validation, quality control and run acceptance criteria of quantitative multi-analyte LC-MS/MS assays has recently been reviewed by us [120].

For those metabolites that we can reliably quantify (likely 13-O-desmethyl tacrolimus only) we will perform similar modeling and bioequivalence metrics as described for tacrolimus.

21. Data review and database management

21.1. Site monitoring

At the site initiation visit, the principal investigator will review the protocol and CRFs in REDCap with the investigators and their staff. During the study, the field monitor will visit the site regularly to check the completeness of subject records, the accuracy of entries on the CRFs, the adherence to the protocol and to Good Clinical Practice, the progress of enrollment, and to ensure that study drug is being stored, dispensed, and accounted for according to specifications. Key study personnel must be available to assist the field monitor during these visits.

The investigator must maintain source documents for each subject in the study, consisting of case and visit notes (hospital or clinic medical records) containing demographic and medical information, laboratory data and the results of any other tests or assessments. All information on CRFs must be traceable to these source documents in the subject's file. Data not requiring a separate written record will be defined before study start and will be recorded directly on the CRFs. The investigator must also keep the original informed consent form signed by the subject (a signed copy is given to the subject).

The investigator must give the monitor access to all relevant source documents to confirm their consistency with the CRF entries.

21.2. Data collection

Designated investigator staff must enter the information required by the protocol onto the CRFs in REDCap. Field monitors will review the CRFs for completeness and accuracy and instruct site personnel to make any required corrections or additions. The CRFs are forwarded to the collaborating investigators, with one copy being retained at the investigational site.

21.3. Database management, quality control and data base lock

Study data will be collected and managed using REDCap electronic data capture tools hosted at the University of Cincinnati. REDCap (Research Electronic Data Capture) is a secure, web-based application designed to support data capture for research studies, providing: (1) an intuitive interface for validated data entry; (2) audit trails for tracking data manipulation and export procedures; (3) automated export procedures for seamless data downloads to common statistical packages; and (4) procedures for importing data from external sources [121]. Subsequently, the entered data are systematically checked by Data Management staff of CCTST, using error messages printed from validation programs and database listings. Obvious errors are corrected by Data Management personnel. Other errors or omissions are entered on Data Query Forms, which are returned to the investigational site for resolution. The signed original and resolved Data Query Forms are kept with the CRFs at the investigator site. Quality control audits of all key safety and efficacy data in the database are made prior to locking the database.

Concomitant medications entered into the database will be coded using the WHO Drug Reference List, which employs the Anatomical Therapeutic Chemical classification system.

Medical history/current medical conditions and adverse events will be coded using the Medical dictionary for regulatory activities (MedDRA) terminology.

Laboratory samples will be processed and results entered into CRFs.

At the conclusion of the study, unused drug supplies will be returned. The occurrence of any protocol deviations will be determined. After these actions have been completed and the database has been declared to be complete and accurate, it will be locked. Only after the database is locked as documented in writing the study will be unblinded to treatment sequence.

22. Data analysis

We have assembled a highly qualified team that will manage and analyze the data including a REDCap database manager who is part of CCTST, Dr. Tran (senior biostatistician), who will be supported by a SAS programmer (Mr. J. Consoer), Dr. Vinks (pharmacogenetics, pharmacokinetics, population pharmacokinetics and pharmacometrics) and Dr. Endrenyi (pharmacokinetics and bioequivalence metrics).

22.1. Analysis sets

The following analysis sets will be used in the analyses:

Safety Set: The Safety Set includes all subjects who received at least one dose of study medication. Subjects will be analyzed according to treatment received.

Dried Blood Spot tacrolimus concentrations: These samples have been collected to identify patients who may not have been tacrolimus steady state blood concentrations and/or have concentrations outside of the target trough blood concentration range.

Full Analysis Set: The Full Analysis Set comprises all subjects to whom study medication has been assigned.

The PK analyses will be based on the subset of subjects from the Full Analysis Set with evaluable PK data.

22.2. Subject demographics and other baseline characteristics

Summary statistics for transplant background and demographic variables including, but not limited to age, race, gender and ethnicity and baseline characteristics will be provided by pooling over all subjects. Continuous variables will be summarized by sample size, mean, median, standard deviation, minimum, and maximum. Discrete variables will be summarized by frequencies and percentages. These analyses will be based on the Full Analysis Set.

22.3. Treatments (study drug, rescue medication, other concomitant therapies, compliance)

Exposure to study medication will be summarized by treatment for all treated subjects. All concomitant therapies will be listed by treatment and by subject.

22.4. Analysis of the primary variable(s)

22.4.1. Variable

The primary pharmacokinetic variables will be

Kidney

- Ratio of C_0, C_{12}, $AUC_{0\text{-}12h}$ and C_{max} and apply CI testing at steady state of Prograf^R to generic Hi in stable kidney transplant subjects.
- Ratio of C_0, C_{12}, $AUC_{0\text{-}12h}$ and C_{max} and apply CI testing at steady state of Prograf^R to Generic Lo in stable kidney transplant subjects.
- Ratio of C_0, C_{12}, $AUC_{0\text{-}12h}$ and C_{max} and apply CI testing at steady state of Generic Hi to Generic Lo in stable kidney transplant subjects.
-

The primary pharmacokinetic variables will be

Liver

- Ratio of C_0, C_{12}, $AUC_{0\text{-}12h}$ and C_{max} and apply CI testing at steady state of Prograf^R to generic Hi in stable liver transplant subjects.
- Ratio of C_0, C_{12}, $AUC_{0\text{-}12h}$ and C_{max} and apply CI testing at steady state of Prograf^R to Generic Lo in stable liver transplant subjects.

Ratio of C_0, C_{12}, $AUC_{0\text{-}12h}$ and C_{max} and apply CI testing at steady state of Generic Hi to Generic Lo in stable liver transplant subjects.

Primary Efficacy and Safety Endpoints

This is a pharmacokinetic comparison study, but efficacy will be measured by reported kidney or liver function or biopsy-proven acute rejection or graft loss as these are the standard efficacy parameters in transplant studies. Safety data will be collected *via* adverse and serious adverse event reporting. Please see also Protection of Human Subjects for further detail. Summary tables (descriptive statistics and/or frequency tables) will be provided for all baseline variables, efficacy variables, and safety variables, as appropriate. Continuous variables will be summarized with descriptive statistics (n, mean, standard deviation, range, and median). Ninety-five (95) percent confidence intervals may also be presented, as appropriate. Frequency counts and percentage of subjects within each category will be provided for categorical data.

22.4.2. Pharmacokinetics and Bioequivalence Metrics

Pharmacokinetic analysis of tacrolimus whole blood concentration data as well as of the concentrations of its metabolites that we can reliably quantitate will be conducted using standard non-compartmental methods (Phoenix WinNonlin, version 6.1 or higher Pharsight/ Certara, St. Louis, MO). The concentration–time profiles of tacrolimus will be explored graphically. C_{max} and T_{max} will be determined by visual inspection of the plasma tacrolimus concentration–time profiles. The apparent terminal elimination rate constant will be estimated for each subject by nonlinear regression analysis. The area under the concentration-time curve (AUC_{0-t} and AUC_{0-inf}) will be determined using the linear trapezoidal method, using each patient's elimination rate constant estimate, which will based on at least six observations. Oral clearance (CL/F), volume of distribution (Vd/F), and terminal t1/2 will be calculated using standard equations. The Mean Residence Time (MRT) will be estimated by AUMC:AUC; where AUMC is the area under the first moment curve.

Concentrations below the Lower Limit of Quantification will be treated as zero in summary statistics for concentration data only. They will not be considered for calculation of PK variables (with the exception of the pre-dose samples).

The primary pharmacokinetic variables will be analyzed for statistical significance. Since subjects who enter this study may be on different twice-daily doses of Prograf^R or generic tacrolimus, as deemed appropriate by their physicians, the primary pharmacokinetic variables will be dose-normalized for comparison and reporting. 95% confidence intervals for the ratio of the pharmacokinetic variables between periods will be calculated.

The mean, standard deviation (S.D.) and coefficient of variation (C.V.) will be calculated for blood concentration at each individual time point, as well as for the pharmacokinetic variables. Additionally, intrasubject variability will be assessed for pharmacokinetic variables for each formulation.

Intra-subject variability will be assessed by calculation of the coefficient of variation and will be expressed as mean±SEM (95% CI) unless otherwise stated. Testing the equality between intra-subject variabilities will be done using Spearman's test which is based upon Spearman's rank correlation coefficient.

A parametric ANOVA model will be used to analyze the pharmacokinetic variables. The ANOVA model will have fixed factors for treatment, period and sequence and a random factor for subject effect (nested within sequences). Statistical significance was to be assessed at the 5% level. In the event that significant carryover effect is detected, the data from the first treatment period will be analyzed using t-tests.

In addition, the relative geometric mean and the 90% confidence interval of the relative geometric mean of pharmacokinetic variables of the formulation comparisons will be calculated.

The PK analyses will be based on the subset of subjects from the Full Analysis Set with evaluable PK data.
These analyses will be carried out using the statistical tools implemented in the Phoenix WinNonlin software or SAS/SPSS as appropriate.

The DBS tacrolimus concentration data set will be used to identify outliers. Such subjects may be excluded from bioequivalence analysis, especially if there is also evidence that lack of adherence was involved. In addition, the DBS tacrolimus concentration data set may be used for pharmacokinetic modeling and may be used to compare the different tacrolimus formulations.

Patients with a missed dose within 48 hours of PK analysis will not proceed to the subsequent PK assessments and will undergo and end of study visits. The rational for exclusion of patients with a missed dose within 48 hours of PK is based upon the following evaluations. To determine the effect of missed dose prior to the PK study on pharmacokinetic (PK) parameter estimates (Cmax, Cmin and AUC_{0-12}) a simulation study was performed. Population PK models were constructed using one- and two-compartmental clearance and volume PK model with first order absorption based on studies in stable liver transplant patients by Antignac at al. [122] and for kidney transplant patients during steady state by Press et al. [123] Models were

defined with between patient variability of 43% on CL and 40% on volume of distribution in liver transplant patients, and 19% on CL and 28% on volume of distribution in kidney transplant patients, respectively . Simulations were performed by randomly sampling 500 times 36 adult subjects with replacement (18,000 total) using the CDC database. All subjects were dosed 0.025 mg per Kg twice a day. Simulations included seven scenarios with all randomly selected subjects and their PK parameters being equal across the different scenarios: one scenario assuming fully adherence and six subsequent scenarios with one missing dose 12, 24, 36, 48, 60 or 72h prior to the study day dose. PK exposure was summarized as predicted maximum and minimum concentrations (Cmax, Cmin) and area under the concentration-time curve from 0 to 12h (AUC0-12h). Data are presented as ratios in relation to the estimates when full adherence is assumed: e.g. AUC ratio at steady state (AUCss, w. missed dose / AUCss, full adherence) for each simulated individual was used to quantify the effect of a missed dose on the AUC.

Figure 8 presents the AUC ratios of the six missed dose scenarios based upon a model developed in liver transplants. If the dose 12h immediately before the study dose is missed the median AUC is approx. 83% of that of the predicted AUC under full adherence. If the dose 36h (3rd) prior to the study dose is missed the predicted median AUC is 90% of the AUC with full adherence. Similar scenarios present the data for Cmax ratios (Figure 9) and Cmin ratios (Figure 10) utilizing the same model.

Figure 11 presents the AUC ratios of the six missed dose scenarios based upon a model developed in renal transplants. If the dose 12h immediately before the study dose is missed the median AUC is approx. 83% of that of the predicted AUC under full adherence. If the dose 36h (3rd) prior to the study dose is missed the predicted median AUC is 90% of the AUC with full adherence. Similar scenarios present the data for Cmax ratios (Figure 12) and Cmin ratios (Figure 13) utilizing the same model.

Based on the results of this simulation study the exclusion criterion was set at 48h, or exclude all patients that have missed a dose 48h prior to the PK study.

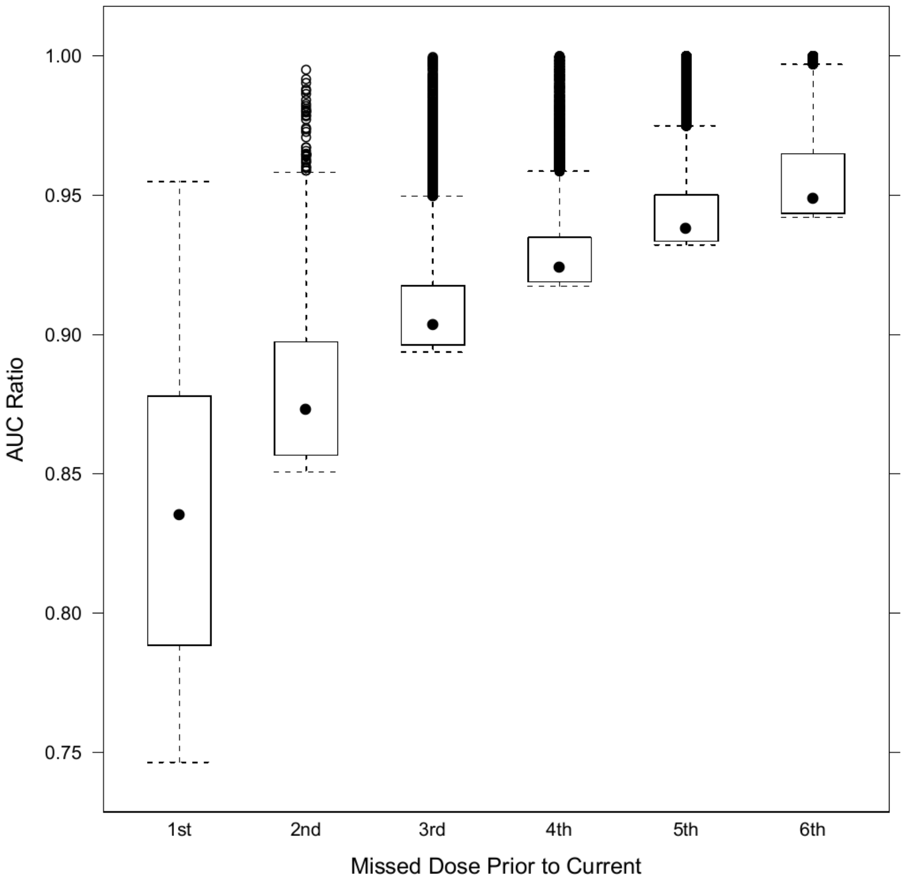

Figure 8. Box-Whisker plot of AUC ratios (AUCss, w. missed dose / AUCss, full adherence) versus one of missed dose scenarios. Results are based on the model developed by Antignac et al. in liver transplant patients. [120]

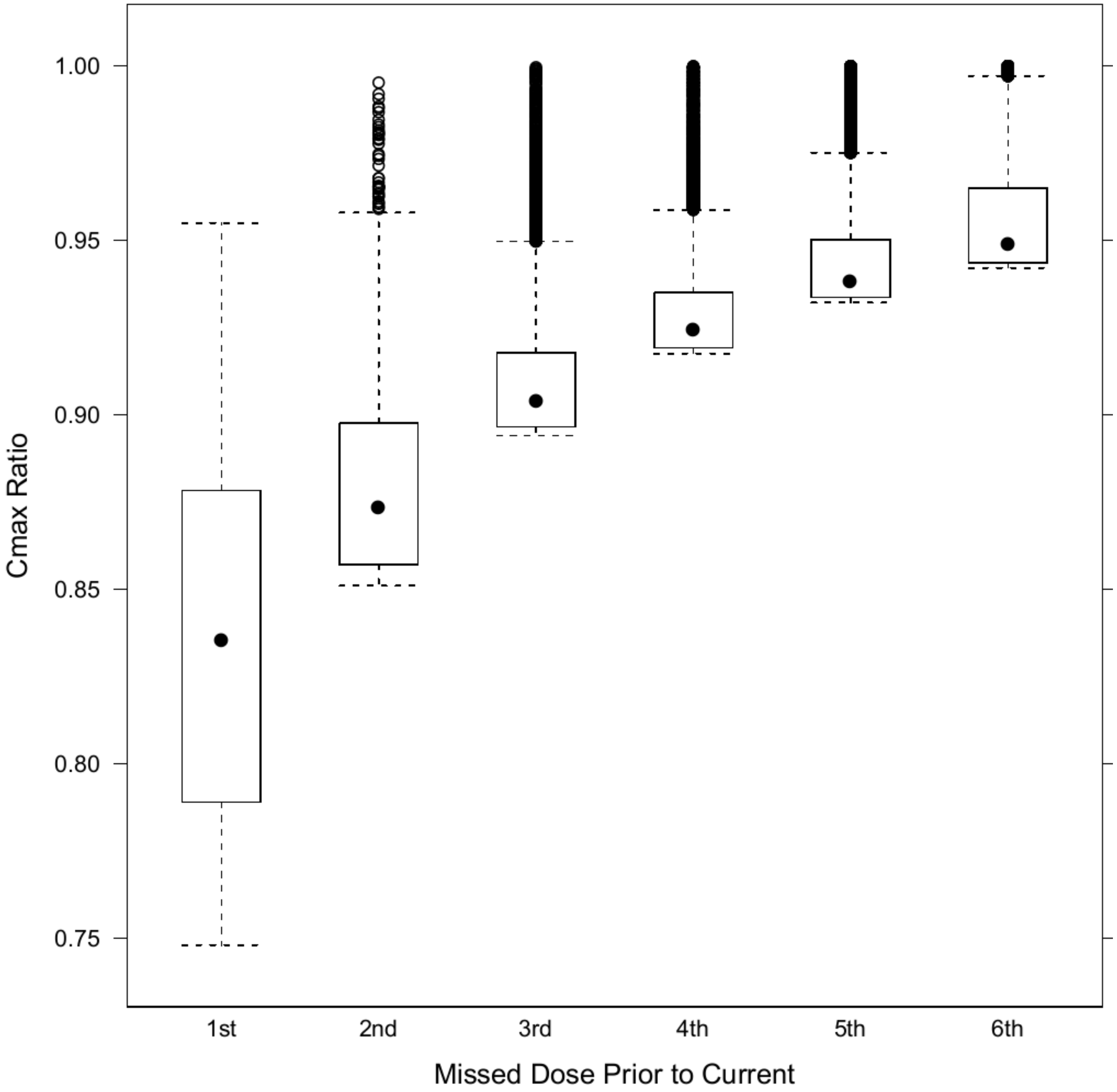

Figure 9. Box-Whisker plot of Cmax ratios (Cmaxss, w. missed dose / Cmaxss, full adherence) versus one of missed dose scenarios. Results are based on the model developed by Antignac et al. in liver transplant patients. [120]

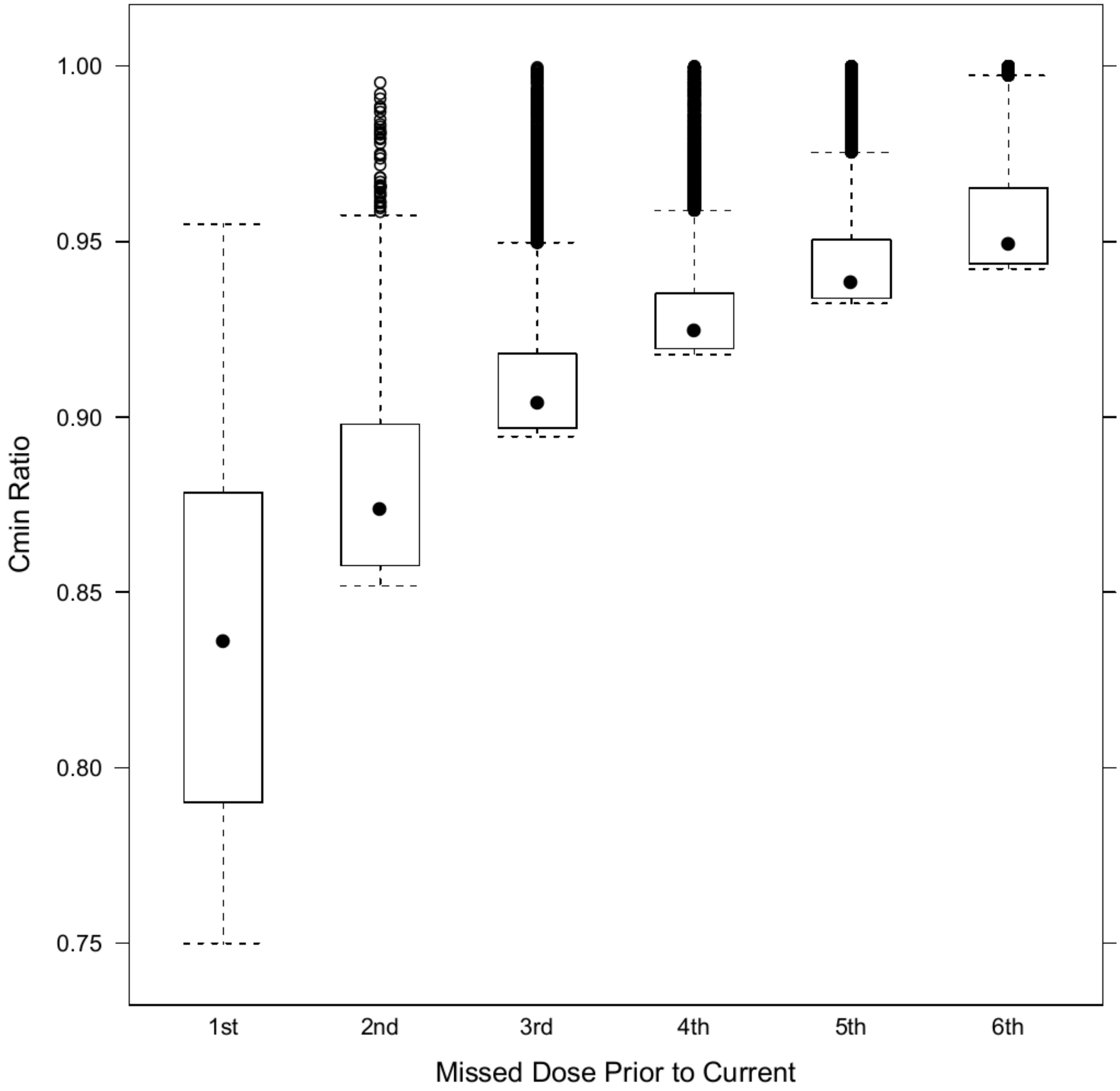

Figure 10. Box-Whisker plot of Cmin ratios (Cminss, w. missed dose / Cminss, full adherence) versus one of missed dose scenarios. Results are based on the model developed by Antignac et al. in liver transplant patients. [120]

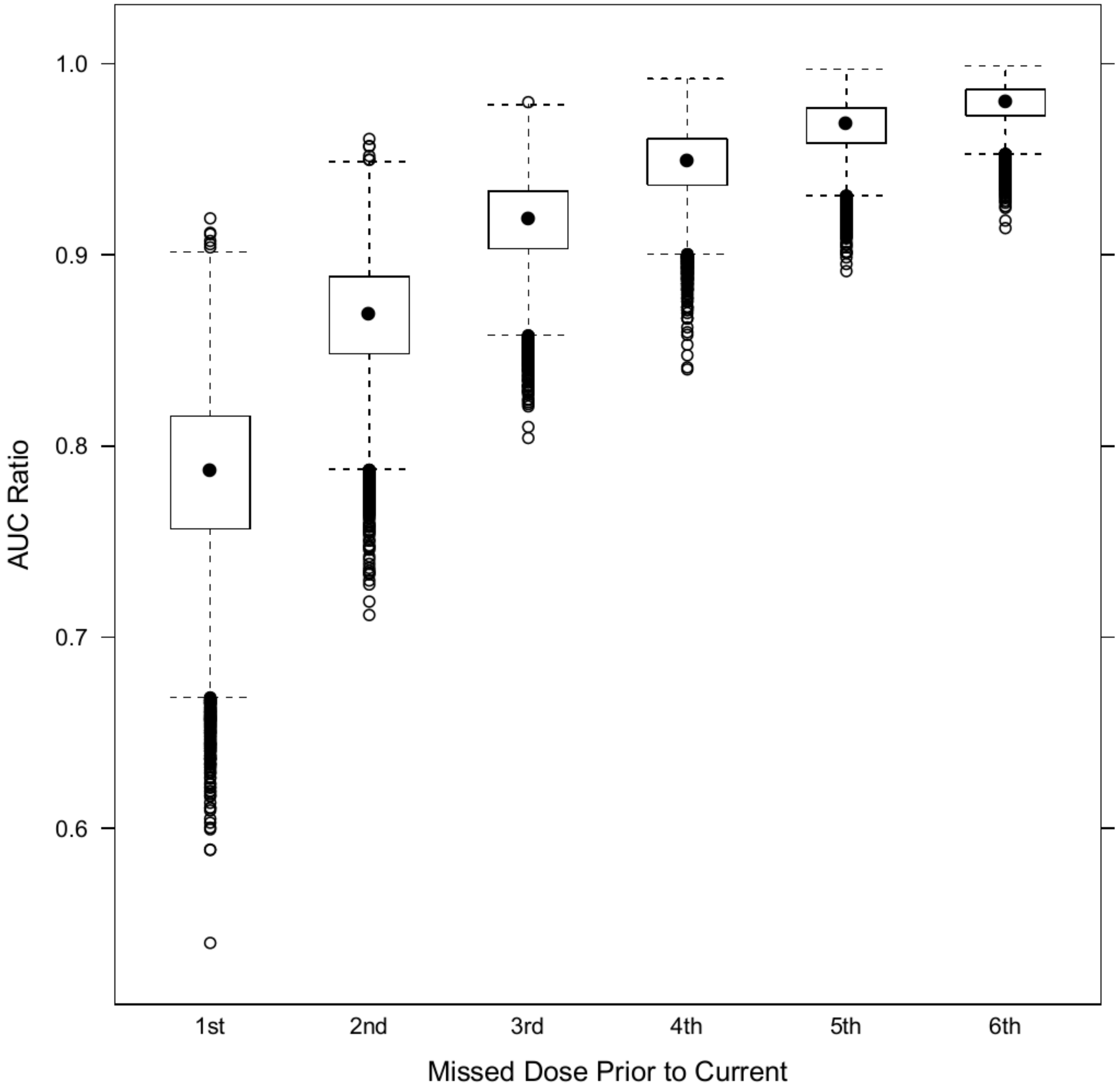

Figure 11. Box-Whisker plot of AUC ratios (AUCss, w. missed dose / AUCss, full adherence) versus one of missed dose scenarios. Results are based on the model developed by Press model in kidney transplant patients. [121]

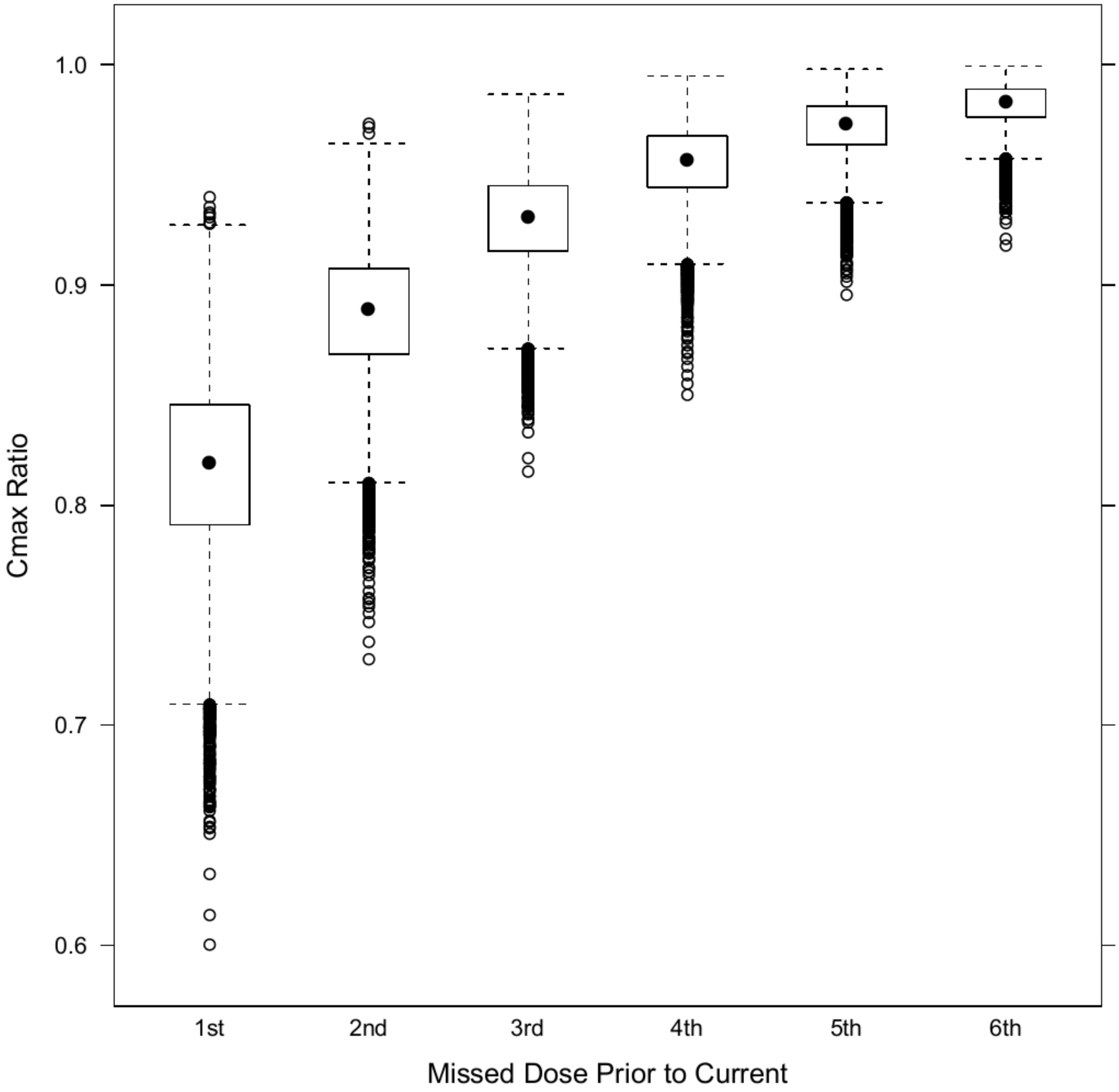

Figure 12. Box-Whisker plot of Cmax ratios (Cmaxss, w. missed dose / Cmaxss, full adherence) versus one of missed dose scenarios. Results are based on the model developed by Press model in kidney transplant patients. [121]

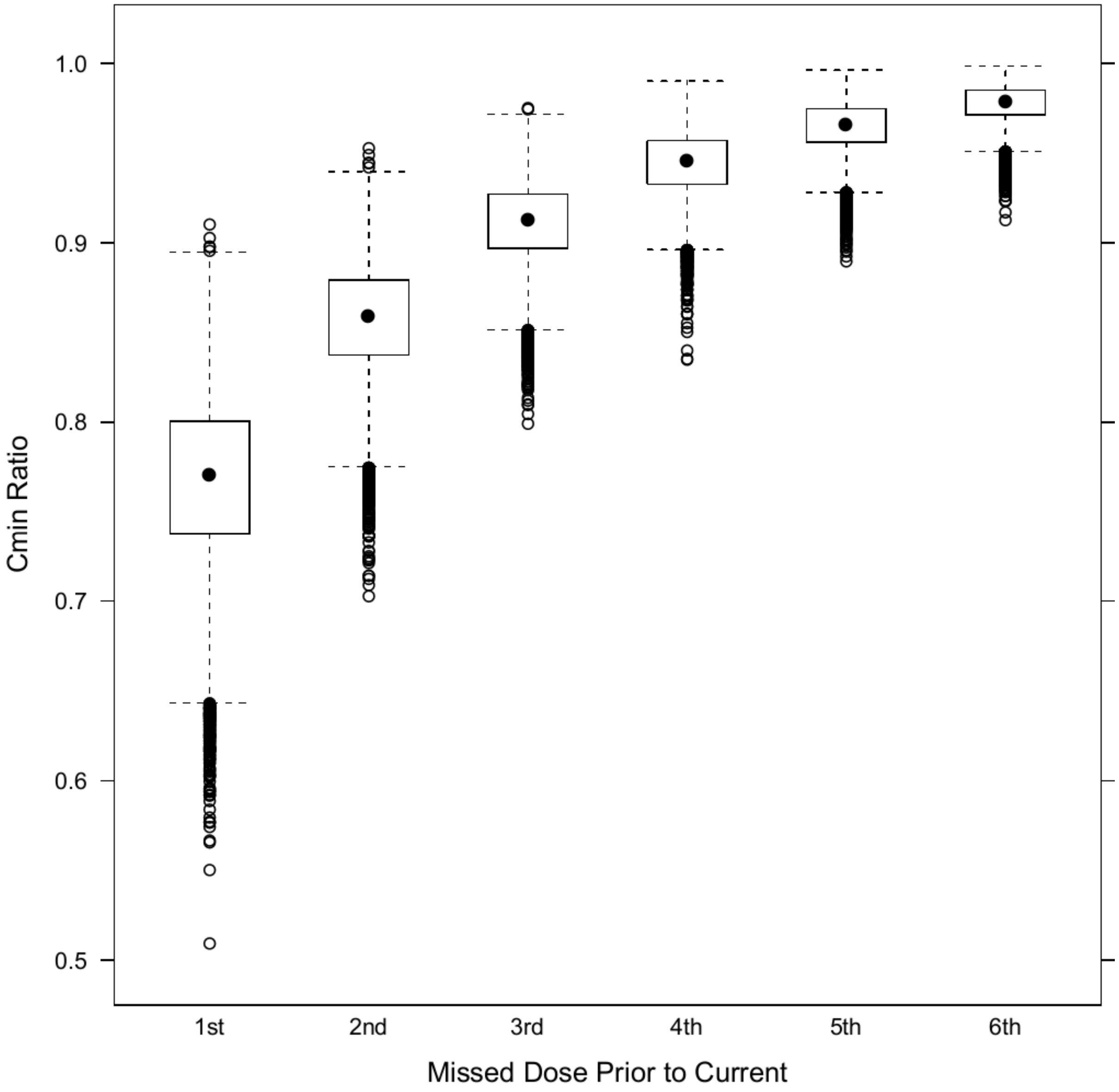

Figure 13. Box-Whisker plot of Cmin ratios (Cminss, w. missed dose / Cminss, full adherence) versus one of missed dose scenarios. Results are based on the model developed by Press model in kidney transplant patients. [121]

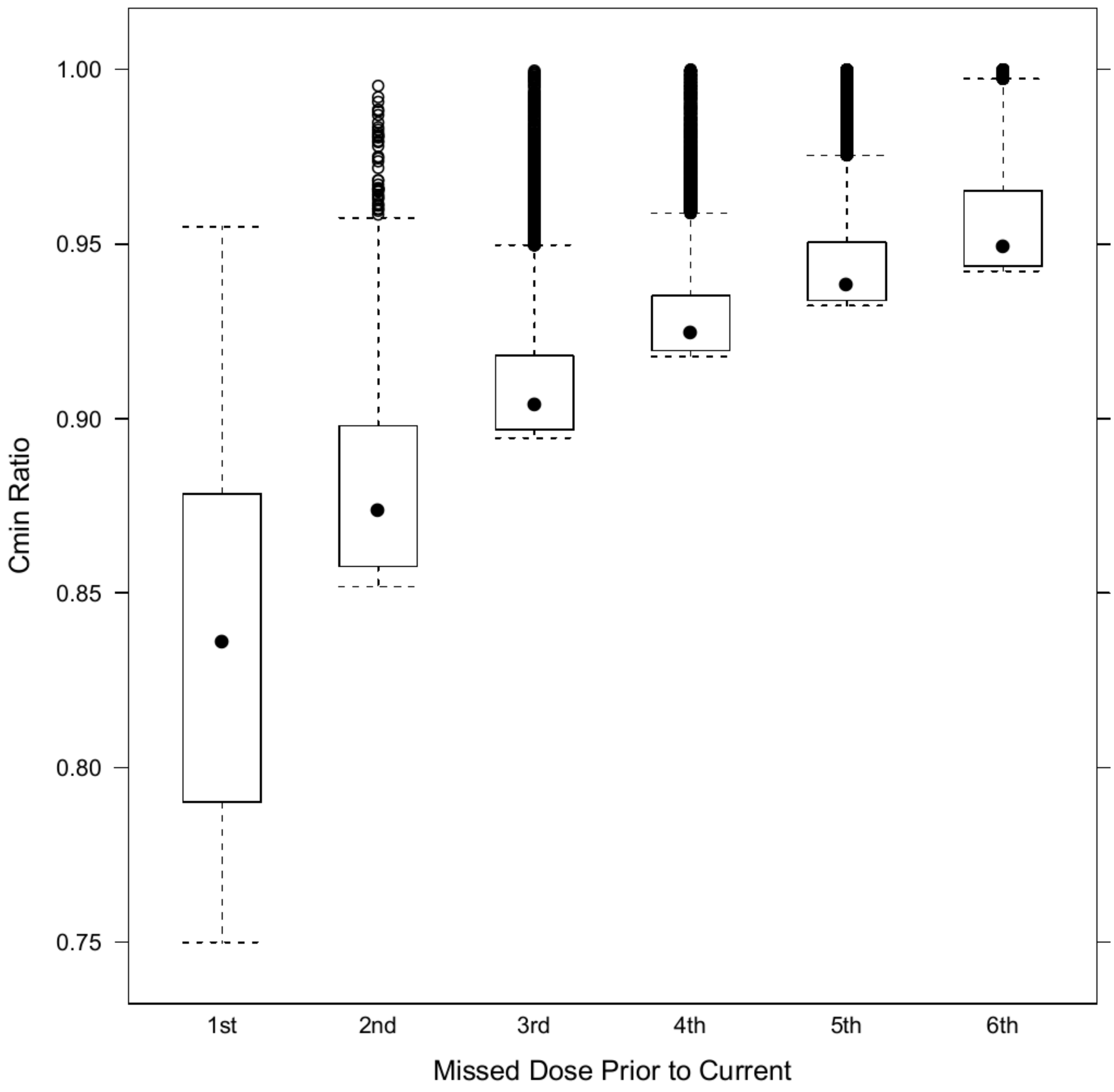

22.4.3. Presentation of data
All individual subject data will be provided. These presentations will include available data from subjects who eventually drop-out from the study. Drop-out and withdrawal of subjects will be fully documented. All individual concentration data and pharmacokinetic parameters will be listed by formulation together with summary statistics such as geometric mean, median, arithmetic mean, standard deviation, coefficient of variation, minimum and maximum. Individual blood concentration/time curves should be presented in linear/linear and log/linear scale. The graphic analysis and presentation will follow the recommendations described in reference [121]. For the pharmacokinetic parameters that were subject to statistical analysis, the point estimate and 90% confidence interval for the ratio of the test and reference products will be presented.

22.4.4. Handling of missing values/censoring/discontinuations
If a pharmacokinetic parameter could not be determined for a period, the corresponding subject will be excluded for that particular statistical comparison.

22.5. Analysis of secondary variables

22.5.1. Efficacy variables

Rates of organ function markers, biopsy-proven rejection and graft loss will be calculated by treatment group.

22.5.2. Safety variables

The assessment of safety will be based mainly on the frequency of adverse events. Other safety data (e.g. vital signs, special tests) will be considered as appropriate.
Adverse events will be summarized for each treatment by presenting the number and percentage of subjects having any adverse event, having an adverse event in each body system and having each individual adverse event. In addition, adverse events which result in discontinuation of the study medication will be summarized and listed separately. Any other information collected (e.g., severity or relatedness to study medication) will be listed, as appropriate.
Laboratory data will be listed by treatment and by subject with all abnormalities flagged.
Data from other tests (e.g. vital signs) will be listed, notable values will be flagged, and any other information collected will be listed as appropriate.
Safety analyses will use subjects in the Safety Set.

23. Sample size calculation and statistical power

In a bioequivalence study, the objective is to demonstrate that two formulations of a drug have similar bioavailability. The assumption is that if the two formulations have equivalent bioavailability then one can infer that they have equivalent effect for both efficacy and safety. Equivalent bioavailability is demonstrated if the drug concentration by time profiles for the “test” and “reference” formulations are super-imposable.

By determining that the two profiles are super-imposable, one can conclude that the two formulations are clinically the same. This is usually done by determining if the rate and extent of absorption are the same, where the pharmacokinetic parameter AUC (area under the concentration curve) is used to assess the extent of absorption, and C_{max} (maximum concentration) is used to assess the rate of absorption.

The objective of this bioequivalence study is to test the null hypothesis to see if the alternative is true. The ‘standard’ bioequivalence criterion (i.e., 0.80–1.25) is to demonstrate that average drug exposure on the test is within 20% of the reference on the log scale [30,106]. Thus, the null and alternative hypotheses can be written as:

$H_0 : \mu_{\text{generic tacrolimus}}/\mu_{\text{reference tacrolimus}} \leq 0.80$ or $\mu_{\text{generic tacrolimus}}/\mu_{\text{reference tacrolimus}} \geq 1{:}25$

$H_1 : 0.80 < \mu_{\text{generic tacrolimus}}/\mu_{\text{reference tacrolimus}} < 1.25$

The generic tacrolimus (GT) and the reference tacrolimus (RT) can be declared bioequivalent if it is demonstrated that the mean ratio is contained within 0.80–1.25. To test the null hypothesis, two one-sided tests at the 5% level are constructed to determine whether $\mu_{GT}/\mu_{RT} \leq 0.80$ or $\mu_{GT}/\mu_{RT} \geq 1{:}25$. If neither of these tests holds, then the alternative hypothesis can be accepted: $0.80 < \mu_{GT}/\mu_{RT} < 1.25$. Because we are performing two simultaneous tests on the null hypothesis, both of which must be rejected to accept the alternative hypothesis, the type I error is maintained at 5%. The two one-sided tests are represented as a 90% confidence interval (CI) around the mean ratio of μ_{GT}/μ_{RT} which summarizes the results of two one-tailed tests.

Thus, a test formulation of generic tacrolimus (Hi or Lo) is bioequivalent to the reference tacrolimus (Brand) if the 90% CI for the ratio test:reference is contained within the range 0.80 to 1.25, for dose-normalized $AUC_{0\text{-}12h}$, ng.h/mL, dose-normalized C_{max}, ng/mL, C_0, ng/mL, and T_{max}, hours.

In addition, a test formulation of Generic tacrolimus Hi is bioequivalent to Generic tacrolimus Lo if the 90% CI for the ratio Generic Hi:Generic Lo is contained within the range 0.80 to 1.25, for dose-normalized $AUC_{0\text{-}12h}$, ng.h/mL, dose-normalized C_{max}, ng/mL, C_0, ng/mL, and T_{max}, hours.

The standard bioequivalence criteria (i.e., 0.80–1.25) will be used to demonstrate that the average generic tacrolimus exposure on the test is bioequivalent to the reference tacrolimus or that this is true for the

comparison of two generics (Generic Hi *versus* Generic Lo). The within-subject coefficient of variation (CV) is expected to be <20% (range is 11.0% to 17.9%, see reference 66, copy is provided in the Appendix), and the mean ratio is expected to be unity (μ_{GT}/μ_{RT} = 1.0). To be conservative, we will assume CV=20%, and μ_{GT}/μ_{RT} =1.05. Although this study is proposed as a six-period, replicate design, we will conservatively estimate sample size based on an AB/BA two period cross-over design.

<u>Sample size</u> Thus, for 90% power, and a type I error rate of 5%, we will require 24 subjects [125]. To adjust for multiple comparisons, we increase the sample size by 30%; and to account for a possible 15% dropout, our final sample size estimate is 38 per kidney study group and 38 per liver study group.

24. Reporting

The final report of this bioequivalence study will be written in accordance with the ICH E3 guideline [126]. Results of the bioequivalence study will be presented as recommended by Sauter et al. [121] (see also 10.4.3.). All data and analyses will be presented in SAS tables.

The analytical report will include a detailed description of the bioanalytical method used, a detailed pre-study validation report and a detailed description of the in study validation results including the results for all standard and quality control samples. A representative number, of chromatograms or other raw data (e.g. for the first 5 subjects) will be included covering the whole concentration range for all standard and quality control samples as well as the specimens analyzed [30].

All reports will contain signed regulatory compliance and quality assurance statements. All reports will be subject to review and approval by Quality Assurance.

25. Expected Outcomes, Problems and Alternative Strategies

It is expected that the study will be conducted with the same lots selected from the initial dissolution, purity and potency testing, and that dissolution and stability testing of the finished product will support final testing in the patient population.

<u>Potential problems and Alternative Strategies:</u>

There are several potential challenges with this study. This is a study that needs to be completed in the shortest possible time period. Reasons are the limited stability of the drugs and the fact that we will use drug supplies from the same lot from the first comparative *in vitro* testing (AIM1) to the end of the clinical trial. In addition, a shorter study time period reduces the chance of changes in the patients' health status that may result in a period effect. However, this will make recruitment more difficult since the 42 study days will be intense and require a very significant time commitment. The Principal Investigator has a successful history of recruiting similar numbers of transplant patients for complex bioequivalence studies with multiple dosing periods. A representative example is reference [66] (for a copy, please see the Appendix 1) We therefore do not anticipate any problems. However, in order to cover all possibilities, we have already spoken to Dr. A. Wiseman (Nephrology) and Dr. M. Zimmerman (Liver Transplantation) at the University of Colorado who would be glad to function as additional study centers. Another potential problem with a 6-period study will be subject retention. In equally complex studies based on transplant patients, the principal investigator has always had an excellent retention record. However, as discussed in the Sample Size Calculation section (section 11, *vide supra*), we have already accounted for 15% drop-outs. In addition, we have planned to replace drop-outs to maintain statistical power.

We are aware that the interval between product dosing is 7 days. It is reasonable to expect that there will be no significant carry over effect between study periods. This is based upon a previous study which compared the PK analysis between days 7 and 14 of the same product. (Reference 66, Appendix 1, table 3) Based upon this previous experience, we have determined that increasing the time for study completion would significantly impact our ability to retain patients through the entire study period and assure proper data for comparison. However, this issue will be discussed with the FDA staff associated with this project if this is of significant concern.

We realize that this is a unique, high-impact clinical trial that will systematically address concerns raised by the transplant community in terms of the validity of average bioequivalence testing and the safety and efficacy of generic immunosuppressants. To ensure maximum confidence in the data and conclusions, it will be critical to rigorously ensure quality of the protocols, analysis plan, study conduct, documentation, data collection and management, bioanalytical procedures, data analysis and the report. Quality assurance and regulatory compliance can be a challenge in many academic research environments. The principal investigator has a long track record of successfully conducting and managing regulatory bioequivalence trials. The bioanalytical laboratory iC42 Clinical Research and Development has conducted quantitative drug measurements for regulatory trials on a regular basis for more than a decade and has all necessary standard operation procedures and quality systems in place. We are therefore confident that the proposed study will be supported by a team of experts in study monitoring and quality assurance.

<u>AIM 4.</u> *Subgroup analysis, individual bioequivalence, population pharmacokinetics, and scaled average bioequivalence*

25.1. Rationale

In addition to the concerns that bioequivalence as established in healthy individuals cannot be transferred to highly-complex and diverse transplant patient populations and that generics even if bioequivalent to the brand may not necessarily be bioequivalent among each other, both of which will be addressed in Aim 3, there are additional concerns regarding the simplicity of the bioequivalence metrics and the suspicion that this is a "one-size-fits-all" approach that may not apply to narrow therapeutic index drugs such as tacrolimus in a highly variable patient population [1,6-9,60]. These concerns are:

(A) There are subgroups of transplant patients that are considered "poor absorbers" and have been shown to be high risk patients [78]. Specific ethnic groups such as African Americans often belong into this category. However, in the meantime it has also been shown that this is caused by genetic polymorphisms, especially the cytochrome P4503A5 genotype and ABCB1 haplotype (p-glycoprotein). It has been speculated that bioequivalence established in healthy individuals does not translate in this especially problematic subgroup of patients.

(B) Also diseases, e.g. *via* the SXR nuclear receptor, may modify the expression and activity of drug metabolizing enzymes and may thus affect absorption and variability of tacrolimus pharmacokinetics [36]. Diseases such as diabetes may also affect absorption *via* changes of stomach and gut motility.

(C) The current average bioequivalence metrics required for establishing bioequivalence of tacrolimus generics and brand do not take factors into consideration that are of critical importance for transplant patients such as intra-individual variability. Thus, alternative bioequivalence metrics such as individual bioequivalence, population pharmacokinetic algorithms and scaled bioequivalence approaches have been discussed.

Hence it will be the goal of AIM 4 to conduct analysis in patient subgroups, to evaluate the data in Aim 3 using alternative statistical bioequivalence testing approaches, and to compare the conclusions with those drawn based on the currently recommended statistical approach [25] as used in Aim 3. This will test the following hypotheses:

- The three formulations tested (Brand, Generic Hi and Generic Lo) will be bioequivalent with each other even in patients who are known expressors of cytochrome P4503A5 and/or have high activity of the efflux transporter p-glycoprotein (based on MDR-1/ABCB1 haplotype analysis; poor absorbers)
- Alternative bioequivalence metrics such as narrower acceptance intervals, individual, scaled average bioequivalence and population pharmacokinetic approach will confirm bioequivalence between the brand and the two generics in the complete study population as well as in the above-mentioned subgroups.

26. Population Pharmacokinetics/ Pharmacometrics

A population PK analysis of tacrolimus will be performed using nonlinear mixed effect modeling (NONMEM, version 7.2, ICON Dev. Soln., Ellicott City, MD) with PDx-Pop® (version 3.0, 2007 ICON Dev. Soln., Ellicott City, MD) interfaced with Xpose® (version 4.0, release 6, update 1). The analysis will include the development

of a base model defining the structural PK model (one- or two compartment model) and a final covariate model describing the impact of patient characteristics, with all statistical and graphical analysis generated from NONMEM output. An exponential variance model will be used to describe the variability of pharmacokinetic parameters across individuals in the form: Pi = θk exp(ηki) where Pi is the estimated parameter value for the individual subject i, θk is the typical population value of parameter k, ηki are the inter-individual random effects for individual i and parameter k. First order conditional estimation (FOCE) with interaction methods will be used throughout. Models will describe the disposition of tacrolimus following oral administration and during maintenance treatment.

During model development, compartment models will be parameterized in terms of values of oral clearance (CL/F), volume of distribution (Vd/F) and absorption rate constant (Ka) with AUC estimated. Models will be evaluated and selected based on goodness of fit and a variety of criteria including physiological plausibility and stability. Standard errors will be assessed for all parameters. Further assessment and comparison will be based on the likelihood ratio test and changes in the objective function value (OFV, -2 log likelihood) between models. Improvement in model fit will be determined using chi-squared distribution with one degree of freedom (ΔOFV <3.84 = p <0.05). Models will also be compared using the Akaike information criterion (AIC) and Schwarz information criterion (SIC) to discriminate between non-hierarchical models in the selection of a structural model. During model development the following diagnostic plots will be used to visually assess model fit: observed vs. population predicted (PRED) or individual predicted (IPRED) values. Plots of residuals and conditional weighted residuals (CWRES) vs. time or PRED were also examined. The final PK models will be further evaluated by generating visual predictive checks.

26.1. Covariate analysis

Patient characteristics to be investigated in an exploratory fashion will include, but will not be limited to age, sex, bodyweight, co-medications, disease status (diabetes), race/ethnicity and genotype. The covariates will be included as either continuous covariates (age and weight) or as dichotomous covariates (sex, race, and genotype). An exploratory analysis will be used to look for relationships between the PK parameters and covariates by visually inspecting plots of the empirical Bayesian (posthoc) estimates of individual parameters from the base model against covariate values. Following the initial analysis, covariates will be included into the model using a forward stepwise inclusion approach and added into the model until there was no further decrease in OFV. Covariates were subsequently removed from the model using a backward stepwise approach. Change of the OFV approximates the chi-squared distribution (χ^2), with one degree of freedom. When there is a change > 3.84 in OFV the significance is p < 0.05; when the change is > 6.63 the significance is p < 0.01. A change in OFV of > 10.83 is considered highly significant, p < 0.001.

Descriptive and exploratory analysis of the primary pharmacokinetic (PK) variables (section 10.4) and the covariates (i.e., age, gender, weight) will be conducted prior to the genetic association tests for the potential impact of candidate gene polymorphisms on inter-individual variability of PK of tacrolimus. The distributions of the primary PK variables will be examined, and the influence of covariates on the primary variables will be assessed using correlation measures. This step will also provide preliminary information to aid the subsequent statistical model selection for genetic association tests.

26.2. Stratification and Subpopulation Analysis

Based on the results of the co-variate analysis, patients may be stratified based on significant co-variates and bioequivalence of Brand, Generic Hi and Generic Lo for tacrolimus and its metabolites will be assessed in these subgroups using the established average bioequivalence metrics as detailed in AIM 3, paragraph 10.4.2. In any case, due to the specific concerns discussed in the aforementioned consensus documents [1,6-9,60], we will at least stratify based on cytochrome P4503A5 genotype and on diabetes/ non-diabetes. We will perform single-marker association tests for each candidate SNP. A regression-based approach will be generally used. Each of the major dosage or PK variables (i.e., dose, AUC, C_{max}, T_{max}, etc.) will be tested as dependent variables and the SNP genotypes coded as 0, 1, and 2 (additive genetic model) will be examined as independent variable. The covariates showing significant influence (p < 0.05) on the dependent variable will

also be included as covariates. The non-parametric test (Kruskal-Wallis test) will be used if the distribution of the dependent variable significantly deviates from the normal distribution.

Power estimation: Previous studies suggested that the PK of tacrolimus is substantial influenced by CYP3A5 variation and, to a lesser extent, by CYP3A4 and ABCB1 variants. In multiple studies, the expressors and the non-expressors of CYP3A5 showed larger than 1 s.d. difference in trough level of tacrolimus, which approximately equals to a variance explained larger than 20% (given the frequency of non-expressors is around 80% in European samples). Given this level of effect, we estimated the power of our pharmacogenetics study of tacrolimus in 72 samples:

Estimated power of PGx study in 72 samples			
Significance level	Proportion of variance explained		
	20%	15%	10%
α=0.05	98.9%	94.6%	80.7%
α=0.05 / 7 *	94.0%	80.9%	55.5%
α=0.05 / 18 *	89.5%	71.7%	43.5%
* 7 and 18 are the numbers of candidate SNPs we will examine in our priority level 1 and 2 studies.			

This power calculation indicated that we should have adequate power to detect genetic association with relatively large effect (~15% variance explained) even when a stringent correction for multiple testing is applied.

We are fully aware that tacrolimus pharmacogenetics in liver transplant patients has to take into account that liver transplant patients are genetic chimera in the sense that they may express cytochrome P4503A5 in the intestine, but not in the transplant liver and *vise versa*. Thus, kidney and liver transplant patients will be analyzed separately as appropriate. If deemed of interest, stratified analyses may also be conducted using the alternative bioequivalence strategies mentioned.

26.3. Evaluation of Tighter Bioequivalence Acceptance Limits

Since 2006, Health Canada has required stricter bioequivalence criteria for narrow therapeutic index drugs such as tacrolimus. Instead of accepting 90% confidence intervals between 80-125%for the AUC, now confidence intervals have to fall between 90-112% [37]. In 2010, the European Medicines Agency followed and is requiring the 90% confidence intervals to fall between 90-111% [30]. The FDA stands behind its current standards and still requires acceptance intervals of 80-125% [25]. Benet and Goyan [4] argue that approved high variability drugs are generally safe and often have relatively flat dose response curves. In the case of drugs with high within–subject variability and steep dose-response curves, patients will frequently experience episodes of a lack of therapeutic effect (drug exposure too low) or toxicity (drug exposure too high) and these drugs typically fail during clinical drug development. Therefore, approved drugs with a steep dose-response curve such as narrow index drugs have relatively low within-subject variability. While bioequivalence testing for highly variable drugs is a challenge and requires large numbers of subjects to achieve adequate statistical power, bioequivalence testing of narrow therapeutic index drugs is usually straight forward. While in the case of highly variable drugs, a subset of subjects may respond differently to the test and reference formulations due to significant subject-formulation interaction, this is hardly ever the case with two bioequivalent formulations of a narrow therapeutic index drug or, due to the narrow inter-subject variability, testing would

reveal that both formulations are not bioequivalent [4,5]. If meeting the 80-125% acceptance criteria, narrow therapeutic index drug typically have no problems to meet these more stringent criteria [4, 5, 22]. This is in contrast to the assumptions made in most consensus documents discussing immunosuppressant generics [1, 6-8, 60]. Therefore, we propose to test the tighter acceptance criteria as adopted by Health Canada and the European Medicines Agency [30, 37] and based on the discussion above that the 90% confidence intervals of the comparisons of Brand, Generic Hi and Generic Lo will also meet these tighter criteria.

26.4. Individual Bioequivalence Metrics

Interchangeability of two drug products can be considered in terms of prescribability and switchability. Switchability, when a patient stabilized on the brand product is switched to a generic tacrolimus formulation or between generic tacrolimus formulations [127], is of greater clinical impact for transplant patients [128,129] and concern [1,6-9,60]. Average bioequivalence testing, which is, as discussed above, the basis of approval of generic drugs in the United States and most other countries, measures prescribability rather than switchability [127,130]. Therefore, the concept of individual bioequivalence has been developed [130-133]. Individual bioequivalence takes a possible subject-by-formulation interaction into account in the computation of the metrics. The subject-by-formulation interaction is important when one formulation is more bioequivalent than the other in one or more subsets of the study population. A large subject-by-formulation interaction is an indicator for a lack of switchability between the test and the reference formulation in some individuals [132]. Individual bioequivalence studies require a replicate design where each subject receives the generic formulation twice and the innovator formulation twice. This study design allows also for estimation of inter- and intra-individual variances. Since 1997 [133,134], the FDA has published three guidance documents on the proposed criterion and statistical methodology for the individual bioequivalence approach.

Despite the theoretical advantages of the individual bioequivalence approach, questions remain on proposed criterion for evaluation of bioequivalence between formulations and, in fact, in several cases was more liberal in accepting bioequivalence than average bioequivalence criteria (for more detail, please see [1231,135]). The FDA maintained the average bioequivalence criterion while allowing other criteria under certain circumstances [135].

Since the proposed study in AIM 3 has a replicate design, this will allow for analysis using individual bioequivalence metrics. The analysis will follow the recommendations detailed in applicable FDA guidance [136]. Studies to assess individual bioequivalence of immunosuppressant drugs have been published by us before [137] and Dr. Endrenyi who is a member of our data analysis team has published key manuscripts on this topic (for example reference [131].

26.5. Scaled Average Bioequivalence

On July 26, 2011 several members of the American Society of Transplantation (AST) including the principal investigator attended a committee meeting of the FDA CDER Advisory Committee for Pharmaceutical Sciences and Clinical Pharmacology to address concerns regarding bioequivalence (BE) testing for narrow therapeutic index (NTI) drugs [138]. The committee voted in favor of revising BE testing procedures for NTI drugs only. The revised procedures call for a two-treatment, four-period, fully replicated crossover design with data analysis performed by the reference-scaled average bioequivalence approach.

In this design, the brand product and the generic product are each administered twice to the subject. All doses administered must be from the same lot. This approach allows for the estimation of the variance of the brand *versus* brand, generic *versus* generic, and brand *versus* generic. In brief, scaled average bioequivalence (sABE) is an approach in which average bioequivalence is scaled based on a variance component. The bioequivalence acceptance limits are scaled based on reference to reference variance from a replicate design study [136]. Alternately the kinetic parameters obtained within a bioequivalence study may be scaled using the same variance. As the acceptance limits are scaled based on the variance, sABE is an alternative, and potentially superior, strategy to fixed tighter acceptance limits for NTI.

The analysis of the data will follow [136] and again Dr. Endrenyi who is a member of our data analysis team has published key manuscripts on this topic [139-145]. Again, analysis in subpopulations (see paragraph 2 above) will be conducted as deemed of interest.

27. Expected Outcomes, Problems and Alternatives Aim 4

Overall, Aim 4 will allow us to directly compare several currently still exploratory bioequivalence testing and analysis strategies among each other and with the standard average bioequivalence approach using the same data set. This takes full advantage of the replicate design of the proposed clinical trial (AIM 3).

Because Aim 4 solely focuses on the analysis of the data set generated in Aim 3 using alternative statistical and pharmacokinetic bioequivalence testing strategies and because members of the data analysis team are leading experts in this field, we do not anticipate any problems.

It is reasonable to expect that this will not only generate valuable and important data in terms of testing generic tacrolimus or other immunosuppressant generic formulations, but will also make a valuable contribution to the current discussion of alternative bioequivalence testing strategies in general.

Proposed Time Schedule

	Year 1		Year 2		Year 3	
Characterization of approved tacrolimus generics and brand including potency, impurity, in-vitro dissolution, and other quality attributes						
Select most disparate generics						
IRB submission, create case report forms, manuals of study operation, build and test study database						
Initiate patient screening						
Conduct 6-period cross over study in stable kidney (n=38) and liver (n=38) transplant recipients						
Study monitoring						
Database cleaning and quality assurance monitoring						
Quantify tacrolimus and metabolites via LC-MS/MS assay and profile pharmacogenetics						
Biostatistical analysis and evaluation of bioequivalence						
Population PK, scaled average, individual bioequivalence and covariate analyses						
Prepare final manuscripts and study reports						

THE FUTURE

The probably most difficult transplant patient population is transplant children at ages below 3 years. Especially in very young children, the current dosage forms do not work as the tacrolimus capsules are either to large, or the capsule strength are too large for dosing small children correctly. Therefore, local pharmacies often dump

the contents of tacrolimus capsules and reformulate the drug locally. The impact of this practice on drug levels is not well controlled and poorly understood. The results of the present study should allow us to design such a study.

REFERENCES

1. Ensor CR, Trofe-Clark J, Gabardi S, McDevitt-Potter LM, Shullo MA. Generic maintenance immunosuppression in solid organ transplantation. Pharmacother 2011; 31: 1111-1129.

2. O'Neil JE. How increased competition from generic drugs has affected prices and returns in the pharmaceutical industry. Congressional Budget Office, July 1988. http://www.fda.gov/ohrms/DOCKETS/dailys/04/June04/061404/03p-0029-bkg0001-Ref-15-vol3.pdf (accessed 5-29-2012)

3. U.S. Department of Health and Human Services. The 2009 annual report of the U.S. organ procurement and transplantation network and the scientific registry of transplant recipients: transplant data 1999-2008. Rockville, MD: Health Resources and Services Administration, Healthcare Systems Bureau, Division of Transplantation.

4. Benet LZ, Goyan JE. Bioequivalence and narrow therapeutic index drugs. Pharmacotherapy 1995; 15: 433-440.

5. Benet LZ. Relevance of pharmacokinetics in narrow therapeutic index drugs. Transplant Proc 1999; 31: 1642-1644.

6. Harrison JJ, Schiff JR, Coursol CJ, Daley CJ, Dipchand AI, Heywood NM, Keough-Ryan TM, Keown PA, Levy GA, Lien DC, Wichart JR, Cantarovich M. Generic immunosuppression in solid organ transplantation: a Canadian perspective. Transplantation. 2012; 93: 657-665.

7. van Gelder T; ESOT Advisory Committee on Generic Substitution. European Society for Organ Transplantation Advisory Committee recommendations on generic substitution of immunosuppressive drugs. Transpl Int. 2011; 24:1135-1141.

8. Uber PA, Ross HJ, Zuckermann AO, Sweet SC, Corris PA, McNeil K, Mehra MR. Generic drug immunosuppression in thoracic transplantation: an ISHLT educational advisory. J Heart Lung Transplant. 2009; 28: 655-660.

9. Alloway RR, Isaacs R, Lake K, Hoyer P, First R, Helderman H, Bunnapradist S, Leichtman A, Bennett MW, Tejani A, Takemoto SK. Report of the American Society of Transplantation conference on immunosuppressive drugs and the use of generic immunosuppressants. Am J Transplant 2003; 3: 1211-1215.

10. Pollard S, Nashan B, Johnston A, Hoyer P, Belitsky P, Keown P, Helderman H. CONSENT: Consensus on Substitution in European Transplantation. A pharmacokinetic and clinical review of the potential clinical impact of using different formulations of cyclosporin A. Berlin, Germany, November 19, 2001. Clin Ther 2003; 25: 1654-1669.

11. Jusko WJ, Thomson AW, Fung JJ, McMaster P, Wong PH, Zylber-Katz E, Christians U, Fitzsimmons W, Lieberman R, McBride J, Kobayashi M, Soldin SJ. Consensus Document: Therapeutic monitoring of tacrolimus (FK506). Ther Drug Monit 1995; 17: 606-614.

12. Christians U, Pokaiyavananichkul T, Chan L. Tacrolimus. In: Pharmacokinetics and Pharmacodynamics. Principles of Therapeutic Drug Monitoring. Burton ME, Shaw LM, Schentag JJ, Evans WE (eds.). 4th Edition, Lipincott, Wiliams & Wilkins, Baltimore (2005), pp. 529-562.

13. Kahan BD, Welsh M, Urbauer DL, Mosheim MB, Beusterien KM, Wood MR, Schoenberg LP, Dicesare J, Katz SM, Van Buren CT. Low intraindividual variability of cyclosporin A exposure reduces chronic rejection incidence and health care costs. J Am Soc Nephrol. 2000;11: 1122-1131.

14. Kahan BD, Welsh M, Schoenberg L, Rutzky LP, Katz SM, Urbauer DL, Van Buren CT. Variable oral absorption of cyclosporine. A biopharmaceutical risk factor for chronic renal allograft rejection. Transplantation 1996; 62: 599-606.

15. Drug Price Competition and Patent Term Restoration Act of 1984. Public Law 98-417, 98 Stat. 1585-1605, September 1984.

16. Dighe SV. A review of the safety of generic drugs. Transplant Proc 1999; 31 (suppl. 3A): 23S-24S.

17. Nightingale SL. From the Food and Drug Administration: Generic substitution. J Am Med Assoc 1998; 279: 645.

18. Davit BM, Nwakama PE, Buehler GJ, Conner DP, Haidar SH, Patel DT, Yang Y, Yu LX, Woodcock J. Comparing generic and innovator drugs: a review of 12 years of bioequivalence data from the United States Food and Drug Administration. Ann Pharmacother. 2009; 43:1583-1597.

19. Helderman JH, Kang N, Legorreta AP, Chen JY. Healthcare costs in renal transplant recipients using branded versus generic ciclosporin. Appl Health Econ Health Policy 2010; 8: 61-68.

20. Klintmalm GB. Immunosuppression, generic drugs and the FDA [Editorial]. Am J Transplant 2011; 11: 1765-1766.

21. Christians U. Generic substitution: taking a narrow view? FirstWord Dossier, November 2010. http://www.firstwordplus.com/dfwdo07350.do;jsessionid=F405D2DE8F44EB0A9355E68FDC5F1B6D (accessed 05-29-2012)

22. Christians U, Klawitter J, Clavijo C. Bioequivalence testing of immunosuppressants: concepts and misconceptions. Kidney Int 2010; 77 (suppl 115): S1-S7.

23. Pondrom S. Can generics be trusted? Am J Transplant 2010; 10: 708-709.

24. Clark-Trofe J, Gabardi S, McDevitt-Potter L, Alloway RR. Immunosuppression, generic drugs and the FDA [Letter to the Editor]. Am J Transplant 2012; 12: 792-793.

25. Food and Drug Administration. Guidance on tacrolimus. Capsule/ oral. Version September 2009. http://www.fda.gov/downloads/Drugs/GuidanceComplianceRegulatoryInformation/Guidances/UCM181006.pdf. Accessed 5/27/2012.

26. http://www.accessdata.fda.gov/scripts/cder/dissolution/ Search term: tacrolimus. Accessed 5/27/2012

27. Kahan BD. Cyclosporine: A powerful addition to the immunosuppressive armamentarium. Am J Kidney Dis 1984; 3:444-455

28. Kahan BD, Shaw LM, Holt D, Grevel J, Johnston A. Consensus document: Hawk's Cay meeting on therapeutic drug monitoring of cyclosporine. Clin Chem 1990; 36:1510-1516.

29. FDA. Bioavailability and bioequivalence requirements. Fed Regist 1992; 57:17997-18001.

30. European Medicines Agency. Guideline on the investigation of bioequivalence. CPMP/EWP/QWP/1401/98 Rev.1/Corr, January 20, 2010. http://www.ema.europa.eu/docs/en_GB/document_library/Scientific_guideline/2010/01/WC500070039.pdf (accessed 05-30-2012)

31. Wallemacq P, Armstrong VW, Brunet M, Haufroid V, Holt DW, Johnston A, Kuypers D, Le Meur Y, Marquet P, Oellerich M, Thervet E, Toenshoff B, Undre N, Weber LT, Westley IS, Mourad M. Opportunities to optimize tacrolimus therapy in solid organ transplantation: report of the European consensus conference. Ther Drug Monit 2009; 31: 139-152.

32. Wong KM, Shek CC, Chau KF, Li CS. Abbreviated tacrolimus area-under-the-curve monitoring for renal transplant recipients. Am J Kidney Dis 2000; 35: 660-666.

33. Mathew BS, Fleming DH, Jeyaseelan V, Chandy SJ, Annapandian VM, Subbanna PK, et al. A limited sampling strategy for tacrolimus in renal transplant patients. Br J Clin Pharmacol 2008; 66: 467-472.

34. Ihara H, Shinkuma D, Ichikawa Y, Nojima M, Nagano S, Ikoma F. Intra- and interindividual variation in the pharmacokinetics of tacrolimus (FK506) in kidney transplant recipients--importance of trough level as a practical indicator. Int J Urol. 1995: 2: 151-155.

35. Staatz CE, Tett SE. Clinical pharmacokinetics and pharmacodynamics of tacrolimus in solid organ transplantation. Clin Pharmacokinet 2004; 43: 623-653.

36. Christians U, Benet LZ, Lampen A. Mechanisms of clinically significant drug interactions associated with tacrolimus. Clin Pharmacokinet 2002; 41: 813-851.

37. Health Canada Guidance for the Industry. Bioequivalence requirements: critical dose drugs. 2006. http://www.hc-sc.gc.ca/dhp-mps/prodpharma/applic-demande/guide-ld/bio/critical_dose_critique-eng.php (accessed 05-30-2012)

38. Haidar SH, Davit B, Chen ML, Conner D, Lee L, Li QH, Lionberger R, Makhlouf F,Patel D, Schuirmann DJ, Yu LX. Bioequivalence approaches for highly variable drugs and drug products. Pharm Res 2008; 25: 237-241.

39. Tacrolimus (Prograf®): Prescribing Information. Astellas Pharma US, Inc., Deerfield, IL, USA. Revised September 2011. http://www.astellas.us/docs/prograf.pdf (accessed 05-30-2012)

40. Anglicheau D, Flamant M, Schlageter MH, Martinez F, Cassinat B, Beaune P, Legendre C, Thervet E. Pharmacokinetic interaction between corticosteroids and tacrolimus after renal transplantation. Nephrol Dial Transplant 2003; 18: 2409-2414.

41. Alloway RR. Generic immunosuppressant use in solid organ transplantation. Transplant Proc 1999; 31: 2S-5S.

42. Gaston R, Alloway RR, Gaber AO, Schroeder TJ, Irish WD, Canafax DM, First MR. Pharmacokinetic and safety evaluation of SangCya vs Neoral or Sandimmune in stable retransplant recipients. Transplant Proc 1999; 31: 326-327.

43. Alloway R, Steinberg S, Khalil K, Gourishankar S, Miller J, Norman D, Hariharan S, Pirsch J, Matas A, Zaltzman J, Wisemandle K, Fitzsimmons W, First MR. Two years postconversion from a prograf-based regimen to a once-daily tacrolimus extended-release formulation in stable kidney transplant recipients. Transplantation 2007; 83:1648-1651.

44. Alloway R, Steinberg S, Khalil K, Gourishankar S, Miller J, Norman D, Hariharan S, Pirsch J, Matas A, Zaltzman J, Wisemandle K, Fitzsimmons W, First MR. Conversion of stable kidney transplant recipients from a twice daily Prograf-based regimen to a once daily modified release tacrolimus-based regimen. Transplant Proc 2005; 37: 867-870.

45. Florman S, Alloway R, Kalayoglu M, Punch J, Bak T, Melancon J, Klintmalm G, Busque S, Charlton M, Lake J, Dhadda S, Wisemandle K, Wirth M, Fitzsimmons W, Holman J, First MR. Once-daily tacrolimus extended release formulation: experience at 2 years postconversion from a Prograf-based regimen in stable liver transplant recipients. Transplantation. 2007; 83: 1639-1642

46. Alloway RR, Vanhaecke J, Yonan N, White M, Haddad H, Rabago G, Tymchak, Molina BD, Grimm M, Eiskjaer H, Karpf C, Undre N. Pharmacokinetics in stable heart recipients following conversion from twice daily to once daily tacrolimus formulations. J Heart Lung Transplant. 2011; 30: 1003-1010.

47. van Hooff JP, Alloway RR, Trunečka P, Mourad M. Four-year experience with tacrolimus once-daily prolonged release in patients from phase II conversion and de novo kidney, liver, and heart studies. Clin Transplant. 2011; 25: E1-12

48. Bekersky I, Dressler D, Colburn W, Mekki Q. Bioequivalence of 1 and 5 mg tacrolimus capsules using a replicate study design. J Clin Pharmacol 1999; 39: 1032-1037.

49. Bekersky I, Dressler D, Boswell GW, Fergen B, Tracewell W, Mekki Q. Bioequivalence of a new strength tacrolimus capsule under development. Transplant Proc 1998; 30: 1457-1459.

50. U.S. Department of Health and Human Services, Food and Drug Administration, Center for Drug Evaluation and Research and Center for Veterinary Medicine. (2001) Guidance for the Industry. Bioanalytical Method Validation. Version May 2001. http://www.fda.gov/downloads/Drugs/GuidanceComplianceRegulatoryInformation/Guidances/UCM070107.pdf (accessed 11-4-2011)

51. Clinical Laboratory and Standard Institute, www.clsi.org (accessed 11-4-2011)

52. Chang T, Benet LZ, Hebert MF. The effect of water-soluble vitamin E on cyclosporine pharmacokinetics in healthy volunteers. Clin Pharmacol Ther. 1996; 59: 297-303.

53. Christians U, Schmitz V, Haschke M. Functional interactions between p-glycoprotein and CYP3A in drug metabolism. Exp Opin Drug Metab Toxicol 2005; 4: 641-654.

54. Lampen A, Christians U, Guengerich FP, Watkins P, Kolars JC, Bader A, Dralle H, Hackbarth I, Sewing KF. Metabolism of the immunosuppressant tacrolimus in the small intestine: cytochrome P450, drug interactions and interindividual variability. Drug Metab Dispos 1995; 23: 1315-1324.

55. Soldner A, Benet LZ, Mutschler E, Christians U. Active transport of the angiotensin-II antagonist losartan and its main metabolite EXP 3174 across MDCK-MDR1 and caco-2 cell monolayers. Br J Pharmacol 2000; 129: 1235-1243.

56. U.S. Department of Health and Human Services, Food and Drug Administration. Guidance for Industry. Q1A(R2) Stability Testing of New Drug Substances and Products. November 2003. http://www.fda.gov/downloads/Drugs/GuidanceComplianceRegulatoryInformation/Guidances/ucm073369.pdf (accessed 06-04-2012)

57. European Medicines Agency. ICH Topic Q 6 A. Note for guidance specifications: Test Procedures and acceptance criteria for new drug substances and new drug products: chemical substances. (CPMP/ICH/367/96), May 2000. http://www.emea.europa.eu/docs/en_GB/document_library/Scientific_guideline/2009/09/WC500002823.pdf (accessed 06-04-2012)

58. U.S. Department of Health and Human Services, Food and Drug Administration. International Conference on Harmonisation (ICH) - Guidance for Industry: Q3A Impurities in New Drug Substances. Revision 2, May 2008. http://www.fda.gov/downloads/RegulatoryInformation/Guidances/ucm127984.pdf (accessed 06-04-2012)

59. European Medicines Agency. ICH Topic Q 3 B (R2) Note for guidance on impurities in new drug products. (CPMP/ICH/2738/99), June 2006 http://www.emea.europa.eu/docs/en_GB/document_library/Scientific_guideline/2009/09/WC500002676.pdf (accessed 06-04-2012)

60. Sabatini S, Ferguson RM, Helderman JH, Hull AR, Kirkpatrick BS, Barr WH. Drug substitution in transplantation: a National Kidney Foundation White Paper. Am J Kidney Dis 1999; 33: 389-397.

61. McDevitt-Potter LM, Sadaka B, Tichy EM, Rogers CC, Gabardi S. A multicenter experience with generic tacrolimus conversion. Transplantation 2011; 92:653-657.

62. Momper JD, Ridenour TA, Schonder KS, Shapiro R, Humar A, Venkataramanan R. The impact of conversion from Prograf to generic tacrolimus in liver and kidney transplant recipients with stable graft function. Am J Transplant 2011; 11(9):1861-1867.

63. Marfo K, Akalin E, Daniel G, de Boccardo G, Aitken S, Jones T, et al. Clinical outcomes of inadvertent conversion from brand to generic tacrolimus in renal transplant recipients. Am J Transplant 2011; 11 (Suppl 2): Abstract 1635

64. Dick TB, Raines AA, Van der Werf W, Alonso D, Fujita S, Stinson J. Comparison of dose requirements of Sandoz generic tacrolimus with brand Prograf® in kidney transplant recipients. Am J Transplant 2011; 11(Suppl 2): Abstract 1102.

65. Abdulnour HA, Araya CE, Dharnidharka VR. Comparison of generic tacrolimus and Prograf drug levels in a pediatric kidney transplant program: Brief communication. Pediatr Transplant 2010; 14: 1007-1011.

66. Alloway RR, Sadaka B, Trofe-Clark J, Wiland A, Bloom RD. A randomized pharmacokinetic study of generic tacrolimus versus reference tacrolimus in kidney transplant recipients. Am J Transplant (accepted May 2012). A copy is provided in the Appendix to this grant application.

67. Cattaneo D, Perico N, Remuzzi G. Generic cyclosporine formulations: more open questions than answers. Transplant Int 2005; 18: 371-378.

68. Christians U, Braun F, Schmidt M, Kosian N, Schiebel HM, Ernst L, Winkler M, Kruse C, Linck A, Sewing KF. Specific and sensitive measurement of FK506 and its metabolites in blood and urine of liver graft recipients. Clin Chem 1992; 38: 2025-2032.

69. Gonschior AK, Christians U, Braun F, Winkler M, Linck A, Baumann J, Sewing KF. Measurement of blood concentrations of FK506 (tacrolimus) and its metabolites in seven liver graft patients after the first dose by h.p.l.c.-MS and microparticle immunoassay (MEIA). Br J Clin Pharmacol 1994; 38: 567-571.

70. Gonschior AK, Christians U, Winkler M, Linck A, Baumann J, Sewing KF. Tacrolimus (FK506) metabolite patterns in blood from liver and kidney transplant patients. Clin Chem 1996; 42: 1426-1432.

71. Winkler M, Christians U, Stoll K, Baumann J, Pichlmayr R. Comparison of different assays for the quantitation of FK 506 levels in blood or plasma. Ther Drug Monit 1994; 16: 281-286.

72. Mountfield RJ, Senepin S, Schleimer M, Walter I, Bittner B. Potential inhibitory effects of formulation ingredients on intestinal cyctochrome P450. Int J Pharm 2000; 211: 89-92.

73. Christians U, Strom T, Zhang YL, Steudel W, Schmitz V, Trump S, Haschke M. Active drug transport of immunosuppressants: new insights for pharmacokinetics and pharmacodynamics. Ther Drug Monit 2006; 28:39-44.

74. Thorn M, Finnstrom N, Lundgren S, Rane A, Loof L. Cytochromes P450 and MDR1 mRNA expression along the human gastrointestinal tract. Br J Clin Pharmacol 2005; 60:54-60.

75. Floren LC, Bekersky I, Benet LZ, Mekki Q, Dressler D, Lee JW, Roberts JP, Hebert MF. Tacrolimus oral bioavailability doubles with coadministration of ketoconazole. Clin Pharmacol Ther. 1997; 62: 41-49.

76. Mancinelli LM, Frassetto L, Floren LC, Dressler D, Carrier S, Bekersky I, Benet LZ, Christians U. The pharmacokinetics and metabolic disposition of tacrolimus: a comparison across ethnic groups. Clin Pharmacol Ther. 2001; 69:24-31.

77. Health Canada. Bioequivalence requirements: critical dose drugs. www.hc-sc.gc.ca/dhp-mps/prod pharma/applic-demande/guide-ld/bio/critical_dose_critique eng.php. Accessed (06-05-2012).

78. Deierhoi MH, Haug M 3rd. Review of select transplant subpopulations at high risk of failure from standard immunosuppressive therapy. Clin Transplant. 2000; 14: 439-448.

79. Anglicheau D, Legendre C, Thervet E. Pharmacogenetics in solid organ transplantation: present knowledge and future perspectives. Transplantation 2004; 78: 311-315.

80. Macphee IA, Fredericks S, Tai T, et al. Tacrolimus pharmacogenetics: polymorphisms associated with expression of cytochrome P4503A5 and p-glycoprotein correlate with dose requirement. Transplantation. 2002; 74: 1486–1489.

81. Goto M, Masuda S, Kiuchi T, Ogura Y, Oike F, Okuda M, Tanaka K, Inui K. CYP3A5*1-carrying graft liver reduces the concentration/oral dose ratio of tacrolimus in recipients of living-donor liver transplantation. Pharmacogenetics. 2004;14: 471–478.

82. Hesselink DA, van Schaik RHN, van der Heiden IP, van der Werf M, Gregoor PJ, Lindemans J, Weimar W, van Gelder T.Genetic polymorphisms of the CYP3A4, CYP3A5, and MDR-1 genes and pharmacokinetics of the calcineurin inhibitors cyclosporine and tacrolimus. Clin Pharmacol Ther. 2003;74:245–254.

83. Goto M, Masuda S, Kiuchi T, Ogura Y, Oike F, Tanaka K, Uemoto S, Inui K. Relation between mRNA expression level of multidrug resistance 1/ABCB1 in blood cells and required level of tacrolimus in pediatric living-donor liver transplantation. J Pharmacol Exp Ther. 2008; 325: 610-616.

84. Masuda S, Goto M, Fukatsu S, Uesugi M, Ogura Y, Oike F, Kiuchi T, Takada Y, Tanaka K, Inui K. Intestinal MDR1/ABCB1 level at surgery as a risk factor of acute cellular rejection in living-donor liver transplant patients. Clin Pharmacol Ther. 2006; 79: 90-102.

85. Shuker N, Bouamar R, Weimar W, van Schaik RH, van Gelder T, Hesselink DA. ATP-binding cassette transporters as pharmacogenetic biomarkers for kidney transplantation. Clin Chim Acta. 2011 Oct 5. [Epub ahead of print]

86. Kuypers DR, de Jonge H, Naesens M, Lerut E, Verbeke K, Vanrenterghem Y. CYP3A5 and CYP3A4 but not MDR1 single-nucleotide polymorphisms determine long-term tacrolimus disposition and drug-related nephrotoxicity in renal recipients. Clin Pharmacol Ther 2007;82:711-25.

87. Maekawa K, Harakawa N, Yoshimura T, et al. CYP3A4*16 and CYP3A4*18 alleles found in East Asians exhibit differential catalytic activities for seven CYP3A4 substrate drugs. Drug Metab Dispos 2010;38:2100-4.

88. Maekawa K, Yoshimura T, Saito Y, et al. Functional characterization of CYP3A4.16: catalytic activities toward midazolam and carbamazepine. Xenobiotica 2009;39:140-7.

89. Elens L, Bouamar R, Hesselink DA, Haufroid V, van Gelder T, van Schaik RH. The new CYP3A4 intron 6 C>T polymorphism (CYP3A4*22) is associated with an increased risk of delayed graft function and worse renal function in cyclosporine-treated kidney transplant patients. Pharmacogenet Genomics 2012;22:373-80.

90. Elens L, Bouamar R, Hesselink DA, et al. A new functional CYP3A4 intron 6 polymorphism significantly affects tacrolimus pharmacokinetics in kidney transplant recipients. Clin Chem 2011;57:1574-83.

91. Wehland M, Bauer S, Brakemeier S, et al. Differential impact of the CYP3A5*1 and CYP3A5*3 alleles on pre-dose concentrations of two tacrolimus formulations. Pharmacogenet Genomics 2011;21:179-84.

92. Hesselink DA, van Schaik RH, van der Heiden IP, et al. Genetic polymorphisms of the CYP3A4, CYP3A5, and MDR-1 genes and pharmacokinetics of the calcineurin inhibitors cyclosporine and tacrolimus. Clin Pharmacol Ther 2003;74:245-54.

93. Wang J, Zeevi A, McCurry K, et al. Impact of ABCB1 (MDR1) haplotypes on tacrolimus dosing in adult lung transplant patients who are CYP3A5 *3/*3 non-expressors. Transpl Immunol 2006;15:235-40.

94. Mutoh K, Mitsuhashi J, Kimura Y, et al. A T3587G germ-line mutation of the MDR1 gene encodes a nonfunctional P-glycoprotein. Mol Cancer Ther 2006;5:877-84.

95. de Jonge H, Metalidis C, Naesens M, Lambrechts D, Kuypers DR. The P450 oxidoreductase *28 SNP is associated with low initial tacrolimus exposure and increased dose requirements in CYP3A5-expressing renal recipients. Pharmacogenomics 2011;12:1281-91.

96. Benkali K, Premaud A, Picard N, et al. Tacrolimus population pharmacokinetic-pharmacogenetic analysis and Bayesian estimation in renal transplant recipients. Clin Pharmacokinet 2009;48:805-16.

97. Lamba J, Lamba V, Strom S, Venkataramanan R, Schuetz E. Novel single nucleotide polymorphisms in the promoter and intron 1 of human pregnane X receptor/NR1I2 and their association with CYP3A4 expression. Drug Metab Dispos 2008;36:169-81.

98. Schipani A, Siccardi M, D'Avolio A, et al. Population pharmacokinetic modeling of the association between 63396C->T pregnane X receptor polymorphism and unboosted atazanavir clearance. Antimicrob is Chemother 2010;54:5242-50.

99. Sandanaraj E, Lal S, Selvarajan V, et al. PXR pharmacogenetics: association of haplotypes with hepatic CYP3A4 and ABCB1 messenger RNA expression and doxorubicin clearance in Asian breast cancer patients. Clin Cancer Res 2008;14:7116-26.

100. van Erp NP, Eechoute K, van der Veldt AA, et al. Pharmacogenetic pathway analysis for determination of sunitinib-induced toxicity. J Clin Oncol 2009;27:4406-12.

101. Muller YL, Infante AM, Hanson RL, et al. Variants in hepatocyte nuclear factor 4alpha are modestly associated with type 2 diabetes in Pima Indians. Diabetes 2005;54:3035-9.

102. Lee SS, Cha EY, Jung HJ, et al. Genetic polymorphism of hepatocyte nuclear factor-4alpha influences human cytochrome P450 2D6 activity. Hepatology 2008;48:635-45.

103. Lamba V, Panetta JC, Strom S, Schuetz EG. Genetic predictors of interindividual variability in hepatic CYP3A4 expression. J Pharmacol Exp Ther 2010;332:1088-99.

104. Rapoff, M.A. Adherence to Pediatric Medical Regimens. 2nd ed. 2010: Springer.

105. Rohan J, Drotar D, McNally K, Schlucter M, Riekert K, Vavrek P, Schmidt A, Redline S, Kercsmar C. Adherence to pediatric asthma treatment in economically disadvantaged African American children and adolescents: an application of growth curve analysis. J Pediatr Psychol 2010; 35: 394-404.

106. Christians U, Benet LZ, Lampen A. Mechanisms of clinically significant drug interactions associated with tacrolimus. Clinical Pharmacokinetics 41 (2002) 813-851.

107. Center for Drug Evaluation and Research, U.S. Department of Health and Human Services. Guidance for industry. Bioavailability and bioequivalence. Study for orally administered drug products. General considerations. March 2003. http://www.fda.gov/downloads/Drugs/GuidanceComplianceRegulatoryInformation/Guidances/ucm070124.pdf, (last accessed 06/06/2012)

108. Center for Drug Evaluation and Research, U.S. Department of Health and Human Services. Guidance for industry. Bioanalytical method validation. May 2001. http://www.fda.gov/downloads/Drugs/GuidanceComplianceRegulatoryInformation/Guidances/UCM070107.pdf, (last accessed 06/06/2012)

109. Clinical Laboratory and Standards Institute, www.clsi.org (last accessed 11/04/2011).

110. European Medicines Agency. Guideline on bioanalytical method validation. EMEA/CHMP/EWP/192217/2009. Version 07/21/2011.

http://www.ema.europa.eu/docs/en_GB/document_library/Scientific_guideline/2011/08/WC500109686.pdf (last accessed 06/06/2012)

111. Christians U, Braun F, Kosian N, Schmidt M, Schiebel HM, Ernst L, Kruse C, Winkler M, Holze I, Linck A, Sewing KF. High performance liquid chromatography/ mass spectrometry of FK506 and its metabolites in blood, bile and urine of liver grafted patients. Transplant Proc 1991; 23: 2741-2744.

112. Christians U, Jacobsen W, Serkova N, Benet LZ, Vidal C, Sewing KF, Manns MP, Kirchner GI. Automated, fast and sensitive quantification of drugs in blood by liquid chromatography-mass spectrometry with on-line extraction: immunosuppressants. J Chromatogr B 2000; 748: 41-53.

113. Clavijo CF, Thomas JJ, Cromie M, Schniedewind B, Hoffman KL, Christians U,Galinkin JL. A low blood volume LC-MS/MS assay for the quantification of fentanyland its major metabolites norfentanyl and despropionyl fentanyl in children. JSep Sci. 2011 Dec;34(24):3568-77.

114. Clavijo CF, Hoffman KL, Thomas JJ, Carvalho B, Chu LF, Drover DR, Hammer GB, Christians U, Galinkin JL. A sensitive assay for the quantification of morphine and its active metabolites in human plasma and dried blood spots using high-performance liquid chromatography-tandem mass spectrometry. Anal Bioanal Chem. 2011 May;400(3):715-28.

115. Matuszewski BK, Constanzer ML, Chavez-Eng CM. Strategies for the assessment of matrix effect in quantitative bioanalytical methods based on HPLC–MS/MS. Anal Chem 2003; 75:3019–3030.

116. Schüler W, Christians U, Schmieder P, Schiebel HM, Holze I, Sewing KF, Kessler H. Structural identification of 13-demethyl-FK506 and its isomers generated by in-vitro metabolism of FK506 using human liver microsomes. Helv Chim Acta, 1993; 76: 2288-2302

117. Sattler M, Guengerich FP, Yun CH, Christians U, Sewing KF, Cytochrome P4503A enzymes are responsible for biotransformation of FK506 and rapamycin in man and rat. Drug Metab Dispos, 1992; 20: 753-761

118. Christians U, Radeke HH, Kownatzki R, Schottmann R, Sewing KF. Isolation of an immunosuppressive metabolite of FK506. Clin Biochem, 1991; 24: 271-275

119. Christians U, Kruse R, Kownatzki R, Schiebel HM, Schwinzer R, Sattler M, Schottmann R, Linck A, Almeida VMF, Braun F, Sewing KF. Measurement of FK506 by HPLC and isolation and characterization of its metabolites. Transplant Proc, 1991; 23: 940-941.

120. Christians U, Klepacki J, Shokati T, Klawitter J, Klawitter J. Mass spectrometry-based multiplexing for the analysis of biomarkers in drug development and clinical diagnostics- how much is too much? Microchem J (in print)

121. Harris PA, Taylor R, Thielke R, Payne J, Gonzalez N, Conde JG. Research electronic data capture (REDCap) - A meta data-driven methodology and workflow process for providing translational research informatics support, J Biomed Inform. 2009; 42: 377-381.

122. Antignac M, Hulot JS, Boleslawski E, Hannoun L, Touitou Y, Farinotti R, et al. Population pharmacokinetics of tacrolimus in full liver transplant patients: modelling of the post-operative clearance. European journal of clinical pharmacology. 2005 Jul;61(5-6):409-16. PubMed PMID: 15991041.

123. Press RR, Ploeger BA, den Hartigh J, van der Straaten T, van Pelt J, Danhof M, et al. Explaining variability in tacrolimus pharmacokinetics to optimize early exposure in adult kidney transplant recipients. Therapeutic drug monitoring. 2009 Apr;31(2):187-97. PubMed PMID: 19258929.

124. Sauter R, Steinijans VW, Diletti E, Böhm A, Schulz HU. Presentation of results from bioequivalence studies. Int J Clin Pharmacol Ther Toxicol 1992; 30: 233-256.

125. Julious SA. Sample sizes for clinical trials with normal data. Statist Med 2004; 23:1921-1986.

126. Center for Drug Evaluation and Research, U.S. Department of Health and Human Services. Guidance for industry. Structure and Content of Clinical Study Reports. Version July 1996. http://www.fda.gov/downloads/regulatoryinformation/guidances/ucm129456.pdf (last accessed 06/07/2012)

127. Hauck WW, Anderson S. Measuring switchability and prescribability: when is average bioequivalence sufficient? J Pharmacokinet Biopharm 1994; 22: 551-565.

128. Kahan BD. Recommendations concerning the introduction of generic formulations of cyclosporine. Transplant Proc 1999; 31: 1634.

129. Kahan BD. Discussion of recommendations concerning the introduction of generic formulations of cyclosporine. Transplant Proc 1999; 31: 1675-1684 .

130. Anderson S, Hauck WW. Consideration of individual bioequivalence. J Pharmacokinet Biopharm 1990; 18: 259-273.

131. Endrenyi L, Amidon GL, Midha KK, Skelly JP. Individual bioequivalence: attractive in principle, difficult in practice. Pharm Res 1998; 15: 1321-1325.

132. Midha KK, Rawson MJ, Hubbard JW. Individual and average bioequivalence of highly variable drugs and drug products. J Pharm Sci 1997; 86: 1193-1197.

133. Patnaik RN, Lesko LJ, Chen ML, Williams RL, FDA Individual bioequivalence Working Group. Individual Bioequivalence: New concepts in the statistical assessment of bioequivalence metrics. Clin Pharmacokinet 1997; 33: 1-6.

134. U.S. Department of Health and Human Services, Food and Drug Administration, Center for Drug Evaluation and Research (CDER). Draft Guidance for Industry: In vivo bioequivalence studies based on population and individual bioequivalence approaches. Rockville, MD, October 1997.

135. Chen ML, Lesko LJ. Individual bioequivalence revisited. Clin Pharmacokinet 2001; 40: 701-706.

136. Center for Drug Evaluation and Research, U.S. Department of Health and Human Services. Guidance for industry. Statistical Approaches to Establishing Bioequivalence. Version January 2001. http://www.fda.gov/downloads/Drugs/GuidanceComplianceRegulatoryInformation/Guidances/ucm070244.pdf (last accessed 06/07/2012)

137. Canafax DM, Irish WD, Berger-Moran H, Squiers E, Levy R, Pouletty P, First RM, Christians U. An individual bioequivalence approach to compare the intra-subject variability of two ciclosporin formulations SangCya™ and Neoral®. Pharmacology 1999; 59: 78-88.

138. Yu LX. Approaches to Demonstrate Bioequivalence of Narrow Therapeutic Index Drugs. Advisory Committee for Pharmaceutical Science and Clinical Pharmacology. July 26, 2011. http://www.fda.gov/downloads/AdvisoryCommittees/CommitteesMeetingMaterials/Drugs/AdvisoryCommitteeforPharmaceuticalScienceandClinicalPharmacology/UCM266777 (last accessed 06/07/2012)

139. Claxton R, Cook J, Endrenyi L, Lucas A, Martinez MN, Sutton SC. Estimating product bioequivalence for highly variable veterinary drugs. J Vet Pharmacol Ther. 2012 ; 35 Suppl 1:11-16.

140. Tothfalusi L, Endrenyi L. Sample sizes for designing bioequivalence studies for highly variable drugs. J Pharm Pharm Sci. 2012;15:73-84.

141. Tothfalusi L, Endrenyi L, Arieta AG. Evaluation of bioequivalence for highly variable drugs with scaled average bioequivalence. Clin Pharmacokinet. 2009; 48:725-743

142. Endrenyi L, Tothfalusi L. Regulatory and study conditions for the determination of bioequivalence of highly variable drugs. J Pharm Pharm Sci.2009;12:138-149.

143. Tothfalusi L, Endrenyi L, Midha KK. Scaling or wider bioequivalence limits for highly variable drugs and for the special case of C(max). Int J Clin Pharmacol Ther. 2003;41: 217-225

144. Tothfalusi L, Endrenyi L. Limits for the scaled average bioequivalence of highly variable drugs and drug products. Pharm Res. 2003; 20: 382-389.

145. Tothfalusi L, Endrenyi L, Midha KK, Rawson MJ, Hubbard JW. Evaluation of the bioequivalence of highly-variable drugs and drug products. Pharm Res. 2001; 18: 728-733.
