# Supplementary material for: Bioequivalence between innovator and generic tacrolimus in liver and kidney transplant recipients: A randomized, crossover clinical trial
Source: PLoS Med. 2017 Nov 14;14(11):e1002428. doi: 10.1371/journal.pmed.1002428 (PMC5685573; doi:10.1371/journal.pmed.1002428)
Supplement: S2 Text — (DOCX) [file pmed.1002428.s003.docx]

**Statistical Analysis Plan**

CCHMC Division of Biostatistics and Epidemiology

**Pharmacokinetic Studies of Tacrolimus in Transplant Patients**

Principal Investigator: Rita Alloway, Pharm.D., FCCP

Statisticians: Eileen C King, PhD; Yuanshu Zou, PhD

Project Sponsor: United States National Institutes of Helth and the Food and Drug Administration: Office of Generic Drugs

Grant Number (if applicable): U01 FD004573

________________________________________________________________________

**Study Objectives**

1. To assess scaled average bioequivalence of Generic Hi Tacrolimus and Generic Lo Tacrolimus to Brand Tacrolimus and to each other in a prospective, appropriately powered, fully replicated, blinded, 3-way cross-over study using 6 periods including kidney and liver transplant patients.
2. To compare the replicate relative bioavailability in important subgroups of interest among each of the three formulations of tacrolimus.
3. To assess the safety of each of the three formulations of tacrolimus.

**Statistical Analyses Details**

A six period, three treatment, three sequence crossover design was used for this study. Each treatment was replicated two times. The statistical analysis approach for assessing scaled average bioequivalence (SCABE) of each generic tacrolimus product (i.e. Generic Hi, Generic Lo) versus Brand tacrolimus was based on the FDA “Draft Guidance on Warfarin” with recommendation date of December 2012 (<http://www.fda.gov/downloads/drugs/guidancecomplianceregulatoryinformation/guidances/ucm201283.pdf>).

**Pharmacokinetic analysis**

Pharmacokinetic analysis of Tacrolimus whole blood concentration data was conducted using standard non-compartmental methods (Phoenix WinNonlin, version 6.2, Certara Company, St. Louis, MO). The concentration–time profiles of tacrolimus were explored graphically. The maximum observed plasma concentration (Cmax) and the time of the maximum observed plasma concentration (Tmax) were determined by visual inspection of the plasma tacrolimus concentration–time profiles. The area under the concentration-time curve (AUC from time 0 to the time of the last measured concentration at 12h) was determined using the linear trapezoidal method.

SCABE for tacrolimus was assessed in liver and kidney transplant patients separately for the following pharmacokinetic (PK) parameters: AUC_0-t_, Cmax, and Cmin at steady state. SCABE was also assessed for _AUCo-t_  for the metabolite13-O-desmethyl tacrolimus. Each of these parameters was log_e_ transformed prior to analyses. Resulting point estimates and confidence limits were back transformed by exponentiating each value to put the estimates back on the original scales.

The following steps were performed to calculate the SCABE limits.

**Step 1. Determine S_WR_, the estimate of within-subject standard deviation (SD) of the reference product, for the PK parameters.**

For the 6 period crossover design, a term named “REP” was created to indicate whether an observation was obtained on the first or second replication of the treatment. A mixed effects analysis of variance model was run with fixed effects terms of sequence, rep, and sequence by rep interaction. The Satterwaite method for estimating degrees of freedom was used. A random intercept model with random subject nested within sequence was used to account for the repeated measures within subject. This model was run for each of the three products separately to obtain estimates of the within-subject standard deviation for each of the products to be used when each product was used as the reference product. This analysis also provided the degrees of freedom for the corresponding standard deviation.

**Step 2: Use the referenced-scaled procedure to determine criterion bound for individual PK parameter(s).**

A mixed effect analysis of variance model was run to obtain the least square mean estimates for each of the three treatments. Fixed effect terms in the model included sequence, period, and treatment. The model used a random slope term and random subject nested within sequence to account for the repeated measures within a subject. The covariance structure was allowed to vary across the treatment groups. Degrees of freedom were estimated using the Satterwaite method and an unstructured covariance matrix was specified. Appropriate linear contrasts were used to obtain estimates of the difference in the least square means of the log transformed data. Corresponding two-sided 90% confidence limits were obtained.

The criterion bounds were calculated for each of the following BE comparisons: Generic Hi vs. Brand, Generic Lo vs. Brand, Generic Hi vs. Generic Lo, Generic Lo vs. Generic High.

The criterion bound was calculated as follows:

$\theta=\left( \frac{ln(\Delta)}{\sigma_{w0}} \right)^{2}$ where $\sigma_{w0}=0.10$ and $\Delta=1/0.9$.

The 95% upper confidence bound was calculated for the following:

$\left( \bar{Y}_{T}-\bar{Y}_{R} \right)^{2}- \theta s_{WR}^{2}$ (which we defined as X + Y)

The following provides the details for estimating the upper confidence bound:

X=ESTIMATE****2** - STDERR****2**;

BOUND=(max((abs(LOWER)), (abs(UPPER))));

BOUNDX=BOUND****2**;

where the estimate of the difference in means (ESTIMATE) and the standard error of the difference (STDERR) was obtained from the difference in the least square means of the log transformed data from the mixed effects model described in Steps 1 and 2. This model also provided the values for the 90% confidence limits (LOWER, UPPER).

Y= -θ*S^2^_WR_

where S_WR_ is the within subject standard deviation for the reference product

BOUNDY=Y*df_R_/cinv(**0.95**,dfd)

where df_R_ is degrees of freedom for S_WR_ and cinv(0.95, df_R_) is the 95^th^ quantile from the Chi-Square distribution with df_R_.

The final 95% upper confidence bound (criterion bound) is calculated as follows:

CRITBOUND=(X+Y)+ sqrt(((BOUNDX-X)**2)+((BOUNDY-Y)**2)

The upper confidence bound was calculated in accordance with the example in the FDA Draft Guidance on Warfarin (referenced above) and was based on Howe’s Approximation I described in the following reference:

*W. G . Howe (1974) Approximate Confidence Limits on the mean of X+Y Where X and Y are Two Table Independent Random Variables, Journal of the American Statistical Association, 69 (347): 789-794.*

**Step 3: Calculate the SCABE limits based on the within subject variability, regulatory constant, and upper BE limit for narrow therapeutic drugs.**

Define the regulatory constant: σ_w0_=0.10.

Define upper BE limit: Δ=1/0.90.

Calculate lower limit (LL): -[ln_e_(Δ)( σ_wR_/ σ_w0_)]

Calculate upper limit (UL): [ln_e_(Δ)( σ_wR_/ σ_w0_)]

SCABE limits: [ exp(LL), exp(UL)]

*Davit, Barbara, et. al. (2012) Implementation of a Reference-Scaled Average Bioequivalence Approach for Highly Variable Generic Drug Products by the US Food and Drug Administration. The AAPS Journal, Vol. 14, No. 4: 915-924.*

**Step 4: Compare the 90% confidence interval for the ratio of the geometric means of the two products being tested for bioequivalence to the average bioequivalence (ABE) limits. For each comparison, determine if the entire confidence interval falls within the 80%-120% equivalence boundaries. To conclude bioequivalence, the average bioequivalence criteria must also be met.**

**Step 5: Calculate the two-sided 90% confidence interval of the ratio of the within subject standard deviation of the test product to the reference product** $\left( \frac{\boldsymbol{\sigma}_{\boldsymbol{WT}}}{\boldsymbol{\sigma}_{\boldsymbol{WR}}} \right)$**.**

The two-sided 90% confidence interval was calculated using the within subject standard deviations and the corresponding degrees of freedom *(v_T_,v_R_)* obtained in Step 1. The formula used to calculate the interval was:

$$\left( \frac{\frac{S_{WT}}{S_{WR}}}{\sqrt{F_{0.05,v_{T,},v_{R}}}} , \frac{\frac{S_{WT}}{S_{WR}}}{\sqrt{F_{0.95,v_{T,},v_{R}}}} \right)$$

The upper limit of this confidence interval was compared to 2.5.

**Step 6: SCABE was concluded if both of the following criterion were met:**

1) the two-sided 90% confidence interval calculated for the average bioequivalence assessment must fall entirely within the SCABE limits

2) the upper 90% confidence limit for the ratio of the within subject variabilities for the two treatments being assessed must be less than 2.5.

**Subgroup analyses**

ABE and SCABE were assessed for the following subgroups of interest:

- Sex: ( male, female)
- Age Group: (≤ 60 years, > 60 years)
- Race: (African-American, Not African-American) (Kidney transplant patients only)
- Diabetes Status: (Yes, No)
- Recipient POR 28: (C/C, C/T or T/T combined)
- Recipient CYP3A5: (*3/*3, *1/*1 or *1/*3 combined)
- Recipient CYP3A4*1B (T/T, C/T or C/C combined) (Kidney transplant patients only)
- Recipient ABCB1 3435C>T: (C/C, C/T or T/T combined) (Kidney transplant patients only)
- Donor CYP3A5 (*3/*3, *1/*1 or *1/*3 combined) (Liver transplant patients only)

Within each of the levels of the subgroup, point estimates and two-sided 90% confidence limits for the ratio of the geometric means for AUC_0-t_ were calculated using the mixed effects analysis of variance model for the 6 period, 3 treatment crossover design as described above for the entire population. These analyses were performed for kidney and liver transplant patients separately. Graphical displays of the ratios and the confidence intervals are presented along with the symbol “x” to indicate the SCABE limits. The graphs also display the sample size within each subgroup and the upper confidence limit for the ratio of the within subject standard deviations for the two treatments being assessed for bioequivalence.

**Safety Assessment**

*Adverse Events*

Adverse events were summarized for each of the treatment groups. Adverse events were assigned to each treatment group based on the start date of the event. Summaries included the number of patients that had at least one occurrence of the adverse event that had a first reporting while on the treatment. In addition, the total number of adverse events that had a first reporting of the event while on treatment is displayed. The analysis was for descriptive purposed only and no inferential testing was performed for adverse events.

*Kidney and Liver Function Assessment*

To assess kidney function, serum creatinine (mg/dL) was evaluated in kidney and liver transplant patients. Alkaline phosphatase (Units/Liter), SGPT (Units/Liter), and SGOT (Units/Liter) were evaluated to assess liver function in liver transplant patients.

The mean (± standard error) of the laboratory parameters were displayed using two different graphical approaches: 1) summary statistics were calculated and displayed chronologically for eight time periods including baseline, randomization, and the 6 periods (including all treatments in the calculation); and 2) summary statistics were calculated and displayed for eight groupings including baseline, randomization, the two replications of each treatment separately. One display assesses changes over the course of the study in the laboratory parameter across all treatments combined. A second display assesses the changes between the replications of the same treatment and also assesses the changes among the treatments. The analysis was for descriptive purposes only and no inferential testing was performed.

**Software Used**

SAS version 9.3 was used to perform calculations and SAS Proc Mixed was used to perform the mixed effects analyses.
