# Supplementary material for: Bioequivalence between innovator and generic tacrolimus in liver and kidney transplant recipients: A randomized, crossover clinical trial
Source: PLoS Med. 2017 Nov 14;14(11):e1002428. doi: 10.1371/journal.pmed.1002428 (PMC5685573; doi:10.1371/journal.pmed.1002428)
Supplement: S1 Appendix — (DOCX) [file pmed.1002428.s004.docx]

**S1 Appendix**

**Bioequivalence Between Innovator and Generic Tacrolimus Formulations in Liver and Kidney Transplant Recipients: a Randomized Crossover Clinical Trial**

**Supporting Information - Methods**

Table of Contents

[A. Quantification of Tacrolimus in EDTA Whole Blood 3](#_Toc496176202)

[Figure A - Connections of the Switching Valve 6](#_Toc496176203)

[Table A – Key HPLC Settings 6](#_Toc496176204)

[Table B – Key Electrospray Interface and Mass Spectrometer Settings 7](#_Toc496176205)

[Table C – Summary of Key Assay Performance Parameters and Validation Results 8](#_Toc496176206)

[B. Quantification of Tacrolimus Metabolites in EDTA Whole Blood 9](#_Toc496176207)

[Table D – Key HPLC Settings 12](#_Toc496176208)

[Table E – Key Electrospray Interface and Mass Spectrometer Settings 13](#_Toc496176209)

[Table F – Summary of Key Assay Performance Parameters During Study Sample Analysis 14](#_Toc496176210)

[C. CyP3A4/5, P450 Oxidoreductase and ABCB1/MDR1 Genotyping 15](#_Toc496176211)

[Table G – Allele Information for SNP genotyping 15](#_Toc496176212)

[D. Tacrolimus Selection Criteria Review of ANDA Data 15](#_Toc496176213)

[Table H – Tacrolimus AUC_inf_ ANDA Data - Fasting 16](#_Toc496176214)

[Table I – Tacrolimus C_max_ ANDA Data - Fasting 16](#_Toc496176215)

[Table J – Generic Product Comparison from ANDA Data 17](#_Toc496176216)

[E. Product Assay, Impurity and Dissolution Testing 19](#_Toc496176217)

[Table K – USP Assay Results for Amount of Tacrolimus in Study Product (1 mg Capsule) 19](#_Toc496176218)

[Table L – USP Organic Impurities, Procedure 1 for Tacrolimus Study Product 20](#_Toc496176219)

[Table M – USP Dissolution Results for Tacrolimus Study Product – Test Method 1 21](#_Toc496176220)

[Table N – USP Dissolution Results for Tacrolimus Study Product – Test Method 2 22](#_Toc496176221)

[Table O – USP Dissolution Results for Tacrolimus Study Product – Test Method 3 23](#_Toc496176222)

[F. Inclusion and Exclusion Criteria 24](#_Toc496176223)

[Inclusion criteria 24](#_Toc496176224)

[Exclusion criteria 24](#_Toc496176225)

[G. Randomization Sequence and Study Design Diagrams and Blinding 27](#_Toc496176226)

[Figure B – Randomization Sequence and Study Design Diagrams 27](#_Toc496176227)

[H. Study Assessment Schedule 28](#_Toc496176228)

[Table P – Study Assessment Schedule 28](#_Toc496176229)

[I. Adherence Monitoring 31](#_Toc496176230)

[J. Pharmacokinetic Sample Handling and Storage 32](#_Toc496176231)

[K. Study Investigators and other personnel 33](#_Toc496176232)

[Corresponding Author 33](#_Toc496176233)

[Authors 33](#_Toc496176234)

[Consultants 36](#_Toc496176235)

[Study Coordinators 41](#_Toc496176236)

[Investigational Drug Services 43](#_Toc496176237)

[IRB/IEC Oversight 43](#_Toc496176238)

[Cincinnati Children’s Hospital and Medical Center Data Management 44](#_Toc496176239)

[Food and Drug Administration 45](#_Toc496176240)

[L. References 46](#_Toc496176241)

# Quantification of Tacrolimus in EDTA Whole Blood

The assay was based on the principles published in [1].

| *Reference compounds* | Tacrolimus (U.S. Pharmacopeial Convention, Rockville, MD, catalogue number 164 2802)  Tacrolimus-D_2_,^13^C (Toronto Research Chemicals, Toronto, ON, Canada, catalogue number F370002) |
| --- | --- |
| *Formula / weight* | C_44_H_69_NO_12_ / 804.018 g/mol |
| *Structure of tacrolimus* | 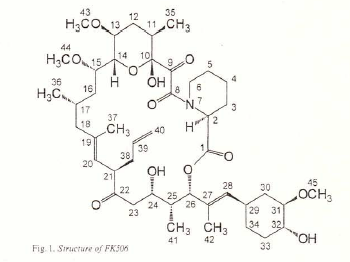 |

Instruments

| *HPLC System* | Two binary pumps, a thermostated column compartment with integrated 6 port switching valve, all Agilent 1100 Series (Agilent Technologies, Palo Alto, CA) |
| --- | --- |
| *Autosampler* | 1100 G13167A Autosampler (thermostated) |
| *Additional switching valve* | Valco valve 6-port switching valve (VICI, Valco Instruments, Waterbury, TX) |
| *MS/MS System* | API-4000 (AB Sciex, Concord, ON) |

Calibrators and Quality Control Samples

| *Calibrators* | 0.5, 1, 2.5, 5, 10, 25, 50 ng/mL tacrolimus + blanks + zero samples, prepared in human EDTA whole blood. |
| --- | --- |
| *Quality Controls* | 2, 4, 20, 40 ng/mL, prepared in human EDTA whole blood. |

Sample Extraction

Two hundred µL of the EDTA whole blood samples were extracted with 800 µL methanol / 0.2 M ZnSO_4_ (7:3, volume/ volume) containing the internal standard tacrolimus-D_2_,^13^C (2.5 ng/mL). Samples were shaken for 10 min, centrifuged at 16,000·g and 4°C for 10 min. Hereafter, 600 µL of supernatant was transferred into glass HPLC vials with pre-slit Teflon caps.

HPLC-MS/MS Analysis

One hundred µL of the extracted sample was loaded onto a cartridge extraction column filled with Zorbax XDB C8 (5 µm particle size, 4.6 · 12.5 mm, Agilent Technologies, Palo Alto CA) and was washed with 5 mL/min 80% 0.1 % formic acid in water and 20% acetonitrile with 0.1% formic acid for 1 min (Table A). Hereafter, the switching valve was activated and the analytes were back-flushed from the pre-column onto the analytical column (filled with Zorbax XDB, 4.6 · 150 mm, 5 µm particle size, Agilent Technologies, Palo Alto CA). The connections of the switching valve are shown in Figure A below. The column thermostat was set to 65°C and the analytes were separated on the analytical column using the gradient shown in Table A below. The analytical column was connected to the tandem mass spectrometer *via* a turbo flow electrospray ionization source. Ions ([M+Na]^+^) were detected in the multiple reaction mode (MRM). The following ion transitions were used for quantification: tacrolimus: m/z= 826.6 → 616.2 and tacrolimus-D_2_,^13^C: m/z= 829.6 → 619.2. Key electrospray and mass spectrometer settings are summarized in Table B.

Tacrolimus was quantified by plotting nominal concentrations *versus* response factor of analyte (Peak Area [Analyte]/Peak Area [IS]). Using a quadratic fit in combination with 1/X weighting gave the best results. All calculations were carried out using AB Sciex Analyst Software (version 1.4.2., AB Sciex, Concord, ON).

The assay was validated following the guidelines set forth in references [2,3] as considered fit for purpose. Key assay performance parameters are summarized in Table C.

The assay regularly and successfully participated in the International Proficiency Testing scheme organized by Prof. Dr. D.W. Holt in the UK ([www.bioanalytics.co.uk](http://www.bioanalytics.co.uk)) as well as in the proficiency testing program provided by the College of American Pathologists ([www.cap.org](http://www.cap.org)). In addition, analysis was successfully traced to a certified tacrolimus standard in blood that was verified using an exact matching isotope dilution reference mass spectrometry method (ERM-DA110, LGC Standards, Teddington, UK).

Figure A - Connections of the Switching Valve

Table A – Key HPLC Settings

| **Loading Pump** | | | | **Analytical (Elution) Pump** | | | |
| --- | --- | --- | --- | --- | --- | --- | --- |
| Time [min] | Water + 0.1% formic acid | Acetonitrile + 0.1% formic acid | Flow rate [µL/min] | Time [min] | Water + 0.1% formic acid | Acetonitrile + 0.1% formic acid | Flow rate [µL/min] |
| 0 | 80 | 20 | 5000 | 0 | 13 | 87 | 1000 |
| 1 | 80 | 20 | 5000 | 2 | 0 | 100 | 1000 |
| 1.1 | 2 | 98 | 200 | 3.5 | 0 | 100 | 1000 |
| 3.0 | 2 | 98 | 200 | 3.6 | 13 | 87 | 1000 |
| 3.1 | 2 | 98 | 5000 |  |  |  |  |
| 3.5 | 2 | 98 | 5000 |  |  |  |  |
| 3.6 | 80 | 20 | 5000 |  |  |  |  |

Table B – Key Electrospray Interface and Mass Spectrometer Settings

| **Parameter** | **Setting** |
| --- | --- |
| Collision gas (CAD) | 12 |
| Curtain gas (CUR) [psi] | 35 |
| Ion Source gas 1 (GS1) [psi] | 50 |
| Ion source gas 2 (GS2) [psi] | 50 |
| Temperature (TEM) [°C] | 500 |
| IonSpray Voltage (IS) [V] | 5500 |
| Interface heater | On |
| Declustering potential (DP) [V] | 111 |
| Entrance potential (EP) [V] | 10 |
| Collision energy (CE) [V] | 61 |
| Collision cell exit potential (CXP) [V] | 24 |

Table C – Summary of Key Assay Performance Parameters and Validation Results

| **Parameter/ Description** | **Nominal Tacrolimus Concentration [ng/mL]** | | | |
| --- | --- | --- | --- | --- |
|  | **2** | **4** | **20** | **40** |
| Intra-day imprecision (n=6, CV%) | 2.0 | 3.4 | 4.0 | 9.5 |
| Intra-day accuracy (n=6, % of nominal) | 89.3 | 89.6 | 95.4 | 92.8 |
| Inter-day imprecision (20 days, n=4/day, CV%) | 7.3 | 9.4 | 7.2 | 6.0 |
| Inter-day accuracy (20 days, n=4/ day, % of nominal) | 93.4 | 95.1 | 95.7 | 95.9 |
| Extraction recovery (%, n=8/ concentration) | 95.8 | 98.9 | 96.8 | 98.1 |
| Matrix effects (according to reference 4, % of signal of neat solution, n=8 samples from 8 different individuals) | 86.6 | 86.2 | 87.1 | 92.2 |
|  |  | | | |
| Lower limit – upper limit of quantification (LLOQ-ULQ) | 0.5- 50 ng/mL | | | |
| Linearity (n=40, 20 days, 2/day) | Average r^2^= 0.9990, 0.9971-0.9998 | | | |
| Matrix interferences (n=8 samples from 8 individuals) | <20% of signal at LLOQ | | | |
| Carry-over (n=40) | <20% of signal at LLOQ | | | |
| Autosampler stability (+4°C) | At least 72 hours | | | |
| Whole blood sample stability:  At room temperature  At +4°C  At -20°C  Freeze-thaw cycle stability (-80°C) | At least 3 days  At least 7 days  At least 7 days  At least 3 cycles | | | |

# Quantification of Tacrolimus Metabolites in EDTA Whole Blood

After tacrolimus was quantified using the validated assay above, tacrolimus metabolites were quantified using a modification of the assay described in references [5,6]. The rationale for running the samples twice was that the quantitative tacrolimus assay was better controlled and validated than the multi-analyte assay measuring the metabolites. Authentic reference materials for the tacrolimus metabolites were not available so that the assay could not be validated to the extent as required by regulatory guidelines [2,3]. As tacrolimus concentrations were the primary outcome parameter of the present study, the strategy of quantifying the tacrolimus metabolites independently ensured that the quality of the tacrolimus measurements was not compromised by simultaneously measurement of the metabolites.

As tacrolimus metabolites were not available from external sources, the metabolites were generated by incubation of tacrolimus with pooled human liver microsomes (Xenotech, Kansas City, KS), isolated using preparative HPLC, the structures were confirmed using ion trap MS^n^ in combination with analysis of fragmentation patterns, purity was established using HPLC-UV-ion trap mass spectrometry and the metabolites were quantified using HPLC-UV-ion trap mass spectrometry based on tacrolimus calibration curves [7-9]. The following isolated metabolites were available (all >99% free of other tacrolimus derivatives):

- 13-O-desmethyl tacrolimus
- 15-O-desmethyl tacrolimus
- 31-O-desmethy tacrolimus
- 12-hydroxy tacrolimus
- 13,31-di-O-desmethyl tacrolimus
- 13,15-di-O-desmethyl tacrolimus

These metabolites were used for identification of their peaks in the ion chromatograms. Since as aforementioned their quantities was estimated using tacrolimus calibrators, tacrolimus calibrators were also used for the quantification of the metabolites in the study samples. This quantification strategy in the absence of pure tacrolimus metabolite reference materials has also been used in previously published studies [5,6], although the assumption that the metabolites have the same signal intensities under the same LC-MS/MS conditions as tacrolimus may not be correct for 15-O-desmethyl tacrolimus, the concentrations of which may be underestimated [10].

Instruments

| *HPLC System* | Two binary pumps, a thermostated column compartment with integrated 6 port switching valve, all Agilent 1100 Series (Agilent Technologies, Palo Alto, CA) |
| --- | --- |
| *Autosampler* | CTC PAL LC autosampler, model PAL HTC (LEAP Technologies, Carrboro, NC) |
| *MS/MS System* | API-4000 (AB Sciex, Concord, ON) |

Calibrators and Quality Control Samples

| *Calibrators* | 0.5, 1, 2.5, 5, 10, 25, 50 ng/mL tacrolimus + blanks + zero sample, prepared in human EDTA whole blood. |
| --- | --- |
| *Quality Controls* | 2, 4, 20, 40 ng/mL, prepared in human EDTA whole blood. |

Sample Extraction

The extracts of the tacrolimus analysis were used for this analysis (*vide supra*).

HPLC-MS/MS Analysis

One hundred µL of the extracted sample was loaded onto a C8 cartridge extraction column (Zorbax-XDB C8 5 µm particle size, Agilent Technologies, Palo Alto, CA) and was washed with 5 mL/min 80% 0.1 % formic acid in water and 20% methanol with 0.1% formic acid for 0.75 min (Table D). Hereafter, the switching valve was activated and the analytes were back-flushed from the pre-column onto the analytical column (3.0 x 100mm, HALO C8 of 2.7 μm particle size, Advanced Materials Technology, Wilmington, DE). The connections of the switching valve are shown in Figure A above. The column thermostat was set to 65°C and the analytes were separated on the analytical column using the gradient shown in Table D. The analytical column was connected to the tandem mass spectrometer *via* a turbo flow electrospray ionization source. Ions ([M+Na]^+^) were detected in the selective ion mode (SIM). The following ions were scanned for quantification: m/z= 826.6 (tacrolimus [M+Na]^+^); m/z= 812.6 (desmethyl-tacrolimus metabolites [M+Na]^+^); m/z= 798.6 (di-desmethyl-tacrolimus metabolites; m/z= 842.6 (hydroxy-tacrolimus [M+Na]^+^); and m/z= 829.6 (tacrolimus-D_2_,^13^C, internal standard, [M+Na^+^]^+^). Key electrospray and mass spectrometer settings are summarized in Table E.

Tacrolimus metabolites were quantified by plotting nominal concentrations *versus* response factor of tacrolimus (Peak Area [Analyte]/Peak Area [IS]). Using a quadratic fit in combination with 1/X weighting gave the best results. Key assay performance parameters are shown in Table F.

Table D – Key HPLC Settings

| **Loading Pump** | | | | **Analytical (Elution) Pump** | | | |
| --- | --- | --- | --- | --- | --- | --- | --- |
| Time [min] | Water + 0.1% formic acid | Methanol + 0.1% formic acid | Flow rate [µL/min] | Time [min] | Water + 0.1% formic acid | Methanol + 0.1% formic acid | Flow rate [µL/min] |
| 0 | 80 | 20 | 5000 | 0 | 35 | 65 | 600 |
| 0.70 | 80 | 20 | 5000 | 0.75 | 35 | 65 | 600 |
| 0.75 | 2 | 98 | 200 | 4.90 | 26 | 74 | 600 |
| 4.40 | 2 | 98 | 200 | 6.00 | 0 | 100 | 600 |
| 6.00 | 2 | 98 | 5000 | 7.00 | 0 | 100 | 600 |
| 7.50 | 2 | 98 | 5000 | 7.10 | 35 | 65 | 600 |
| 8.00 | 80 | 20 | 5000 | 8.00 | 35 | 65 | 600 |

Table E – Key Electrospray Interface and Mass Spectrometer Settings

| **Parameter** | **Setting** |
| --- | --- |
| Collision gas (CAD) | 12 |
| Curtain gas (CUR) [psi] | 40 |
| Ion Source gas 1 (GS1) [psi] | 50 |
| Ion source gas 2 (GS2) [psi] | 35 |
| Temperature (TEM) [°C] | 500 |
| IonSpray Voltage (IS) [V] | 5500 |
| Interface heater | On |
| Declustering potential (DP) [V] | 130 |
| Entrance potential (EP) [V] | 10 |

Table F – Summary of Key Assay Performance Parameters During Study Sample Analysis

| **Parameter/ Description** | **Nominal Tacrolimus Concentration [ng/mL]** | | | |
| --- | --- | --- | --- | --- |
|  | **2** | **4** | **20** | **40** |
| Inter-day imprecision (65 days, n=1/day, CV%) | 10.2 | 7.4 | 5.3 | 8.2 |
| Inter-day accuracy (65 days, n=1/ day, % of nominal) | 91.0 | 93.2 | 102.2 | 97.7 |
|  |  | | | |
| Lower limit – upper limit of quantification (LLOQ-ULQ) | 0.5- 50 ng/mL | | | |
| Linearity (n=65) | Average r^2^= 0.9994, 0.9964-1.0000 | | | |
| Matrix interferences (n=65 blank samples) | <20% of signal at LLOQ | | | |
| Carry-over (n=65) | <20% of signal at LLOQ | | | |
| Autosampler stability (+4°C) | At least 72 hours | | | |
| Whole blood sample stability:  At room temperature  At +4°C  At -20°C  Freeze-thaw cycle stability (-80°C) | At least 3 days  At least 7 days  At least 7 days  At least 3 cycles | | | |

# CyP3A4/5, P450 Oxidoreductase and ABCB1/MDR1 Genotyping

Genomic DNA was isolated from patient’s blood samples using the QIAamp DNA Blood Midi Kit (Qiagen, Valencia, CA, USA). The participants were genotyped for seven single nucleotide polymorphisms (SNPs) in CYP3A5, CYP3A4, P450 oxidereductase (POR), and ABCB1 genes as shown in supplemental Table G based on existing evidence. The genotyping was performed using commercially available TaqMan SNP Genotyping Assays (Applied Biosystems, Foster City, CA, USA). Genotype determination was performed on a StepOnePlus Real Time PCR System (Applied Biosystems) according to manufacturer instructions. [12, 13, 14].

Table G – Allele Information for SNP genotyping

| **Protein** | **Allele name** | **Allele** | **db SNP ID** | **Reference** |
| --- | --- | --- | --- | --- |
| CYP3A5 | CYP3A5*3 | 6986A>G | rs776746 | [12] |
| CYP3A4 | CYP3A4*1B | -290A>G | rs2740574 | [12] |
|  | CYP3A4*22 | 15389C>T | rs35599367 | [13] |
| P450 oxidoreductase | POR*28 | 1508C>T | rs1057868 | [14] |
| P-glycoprotein (ABCB1/MDR1) |  | 1236C>T | rs1128503 | [12] |
|  |  | 2677G>T/A | rs2032582 | [12] |
|  |  | 3435C>T | rs1045642 | [12] |

# Tacrolimus Selection Criteria Review of ANDA Data

All FDA approved tacrolimus ANDA data in healthy volunteers were ranked by AUC_inf_ and C_max_ under fasting and fed conditions results prioritization as follows: 1) Fasting results were prioritized over fed ABE results, 2) Greatest geometric mean ratio distance from 1.00, 3) Widest confidence interval range. The tables below include the AUC_inf_ (fasting) and C_max_ (fasting) ANDA data for all FDA-approved tacrolimus generic formulations as of September 2012.

Table H – Tacrolimus AUC_inf_ ANDA Data - Fasting

| **Product**  **Manufacturer** | **Point**  **Estimate** | **90% Confidence Interval** | |
| --- | --- | --- | --- |
|  |  | **Lower**  **Bound** | **Upper**  **Bound** |
| Sandoz | 1.10 | 1.00 | 1.21 |
| Panacea | 0.92 | 0.85 | 0.996 |
| Dr. Reddy | 0.96 | 0.90 | 1.02 |
| Mylan | 1.02 | 0.95 | 1.09 |
| Accord | 0.98 | 0.93 | 1.03 |

Table I – Tacrolimus C_max_ ANDA Data - Fasting

| **Product**  **Manufacturer** | **Point**  **Estimate** | **90% Confidence Interval** | |
| --- | --- | --- | --- |
|  |  | **Lower**  **Bound** | **Upper**  **Bound** |
| Sandoz | 1.10 | 1.02 | 1.19 |
| Panacea | 1.00 | 0.937 | 1.07 |
| Dr. Reddy | 0.91 | 0.84 | 1.00 |
| Mylan | 1.04 | 0.99 | 1.09 |
| Accord | 1.18 | 1.12 | 1.25 |

Based upon these criteria, the Sandoz manufacturer was selected as the Generic Hi. There were two potential candidates for Generic Lo, Panacea and Dr. Reddy formulations. Upon attempts to purchase both products for testing, it was identified that Panacea was not commercially available as of September 2012. Therefore, it was decided to proceed with Dr. Reddy as the Generic Lo. Under fasting conditions, the point estimate of AUC and C_max_ for Generic Hi (Sandoz) and Lo (Dr. Reddy) differed by 15% and 18%, respectively.

Based on all factors considered above, the following ANDAs are recommended as the most disparate ANDAs for the BE studies.

Tacrolimus predicted low: ANDA 90509

Tacrolimus predicted high: ANDA 065461

Table J – Generic Product Comparison from ANDA Data

|  | **Dr. Reddy Tacrolimus 1mg**  **ANDA 090509** | **Sandoz Tacrolimus 1mg**  **ANDA 065461** |
| --- | --- | --- |
| Bioequivalence data | Under fasting, the point estimate of AUC and Cmax for Dr Reddy and Sandoz differed by 15% and 18%, respectively. | |
| Bioequivalence strength | 5 mg | 5 mg |
| Formulation composition | Similar | |
| Fill weight | 1mg capsule, 140 mg in size “4” capsule  5 mg capsule, 140 mg in size “4” capsule | 1 mg capsule, 50 mg in size “4” capsule  5 mg capsules, 250 mg in size “3” capsule |
| Manufacturing process | Similar, processing solvent slightly different | |
| Dissolution limits | Test conditions differ | |
| Impurity limits | Slightly different | |

All commercially available lots of Prograf 1 mg (Astellas), tacrolimus 1 mg (Sandoz Pharmaceuticals) and tacrolimus 1 mg (Dr. Reddy) were purchased from pharmacy wholesale purchasers. Three Prograf 1 mg (Astellas), four tacrolimus 1 mg (Sandoz Pharmaceuticals) and three tacrolimus 1 mg (Dr. Reddy) lots were purchased for product testing. All lots passed product assay, impurity, and dissolution testing (please see below).

In an effort to complete the entire study with a single lot of each product prior to the expiry date, product acquisition became difficult. Typical expiration dates from the generic manufacturers were 12-18 months. It was determined to purchase all study product and simultaneously proceed with product and individual testing.

# Product Assay, Impurity and Dissolution Testing

Drug products selected for individual testing included Prograf Tacrolimus 1mg (Astellas Pharma, Deerfield IL) Lot # 046661, Generic Hi Tacrolimus 1mg (Sandoz Inc, Princeton, NJ) Lot # DT4399, and Generic Lo Tacrolimus 1mg (Dr. Reddy’s Laboratories Ltd. Bachupally, India) Lot # C306775. Tacrolimus RS (USP Lot # H0M009, purity 97.7%) was used as tacrolimus standards. Products were tested for amount of Tacrolimus in the capsules and impurities by the USP Assay and Organic Impurity Test Procedures (Tacrolimus USP Monograph, USP37, version of December 1, 2014).

Table K – USP Assay Results for Amount of Tacrolimus in Study Product (1 mg Capsule)

Table L – USP Organic Impurities, Procedure 1 for Tacrolimus Study Product

Table M – USP Dissolution Results for Tacrolimus Study Product – Test Method 1

**

Table N – USP Dissolution Results for Tacrolimus Study Product – Test Method 2

**

Table O – USP Dissolution Results for Tacrolimus Study Product – Test Method 3

# Inclusion and Exclusion Criteria

## Inclusion criteria

Individuals were screened and enrolled into the trial if they met all the inclusion criteria on the day of study entry (Visit 1) and on the day of randomization (Visit 2).

Individuals eligible for inclusion in this study have to fulfill **all** of the following criteria:

1. ≥18 years old, male or female
2. Able to participate and willing to give written informed consent and to comply with the study visits and restrictions.
3. Individual who had received a primary or secondary kidney or liver transplant.
4. Individual who was at least 6 months post-transplant and on a stable doses of tacrolimus as defined by physician, one tacrolimus trough level within the physician defined target range within past 6 months and one additional trough level during the screening period within 30% of the physician defined target range.
5. BMI greater than or equal to 19 but less than or equal to 40.
6. Ability to perform daily finger sticks to provide blood sample.

## Exclusion criteria

Individuals were screened and enrolled into the trial if they met none of the exclusion criteria on day of study entry (Visit 1) and on the day of randomization (Visit 2).

Individuals fulfilling **any** of the following criteria are not eligible for inclusion in this study:

1. Evidence of any acute rejection
2. Individuals who required dialysis within 6 months prior to study entry
3. Recipients of multiple organ transplants
4. Individuals who had tested positive for HBsAG or HIV, or who were recipients of organ from donors who were known to be HBsAG or HIV positive. Virology screening at the time of transplant.
5. HepC positive individuals with liver biopsy proven recurrent disease considered relevant by physician oversight.
6. Individuals with any severe medical condition requiring acute or chronic treatment that in the investigator’s opinion would interfere with study participation
7. History of malignancy, treated or untreated, with the past 2 years with the exception of carcinoma in situ or excised basal cell carcinoma, or hepatocellular carcinoma prior to transplant.
8. GFR ≤ 35 ml/min measured as estimated using the MDRD4 formula
9. Individuals with AST, ALT, total bilirubin ≥ 3 · ULN or other evidence of severe liver disease
10. Individuals with white blood cell (WBC) count ≤2,000/ mm3 or with thrombocytopenia (platelet count ≤ 75,000/ mm3), with an absolute neutrophil count of ≤ 1,500/ mm3 or hemoglobin <8g/dL)
11. Individuals with clinically significant infections, requiring therapy, which, in the investigator’s opinion, would interfere with the objectives of the study
12. Other mental or physical conditions, which, in the investigator’s opinion, were considered clinically significant
13. Presence of intractable immunosuppressant complications or side effects resulting in dose adjustment of tacrolimus
14. Individuals who had been exposed to an investigational therapy within 30 days prior to enrollment or 5 half-lives of the investigational product, whichever was greater.
15. An anticipated change in the immunosuppressive regimen during individual participation other than that required by the protocol.
16. Individual with severe GI disturbance or diarrhea which could interfere with tacrolimus absorption
17. Severe diabetic gastroparesis
18. Initiation of any medications that could interfere with tacrolimus blood levels, including OTC medications, herbal supplements, grapefruit or grapefruit juice.
19. Pregnant or nursing (lactating) women, where pregnancy was defined as the state of a female after conception and until the termination of gestation, confirmed by a positive BhCG laboratory test (> 5 mIU/mL)
20. Women of child-bearing potential, defined as all women physiologically capable of becoming pregnant, unless they were
    1. women whose career, lifestyle, or sexual orientation precludes intercourse with a male partner;

women whose partners have been sterilized by vasectomy or

- 1. using a highly effective method of birth control (i.e. one that resulted in a less than 1% per year failure rate when used consistently and correctly, such as implants, injectables, combined oral contraceptives, and some intrauterine devices (IUDs); periodic abstinence (e.g. calendar, ovulation, symptothermal, post-ovulation methods) was not acceptable.

# Randomization Sequence and Study Design Diagrams and Blinding

Figure B – Randomization Sequence and Study Design Diagrams


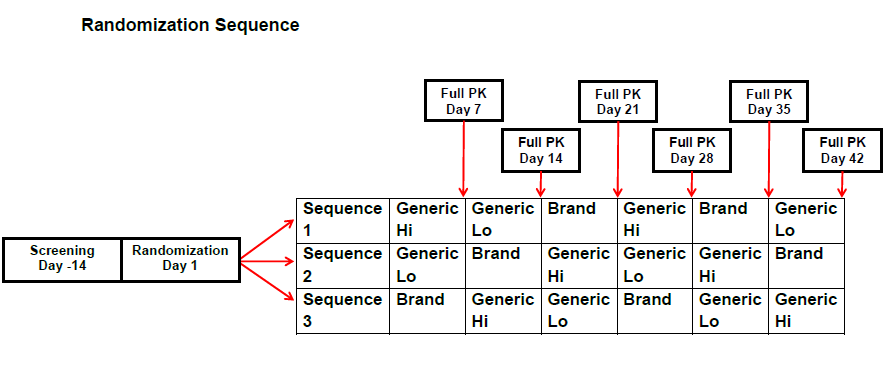


Over-encapsulation was considered for blinding purposes, however, was abandoned due to its potential impact on PK parameters.  The investigational pharmacy was the only group to have access to the randomization sequence until the statistician for final data analysis.  The clinical PK study team in Ohio and the patients were able to identify the tacrolimus formulation by visual inspection, but did not know the sequence of formulation administration.  Tacrolimus samples collected by the PK study team only included the patient identification number, study period, date and time of sample collection.  No information regarding formulation was collected during the PK visits.  Tacrolimus samples were batched shipped to Colorado for LC-MS/MS analysis.  The bioanalytical team in Colorado was completely blinded until database lock.  Sample results were reported to the PK analysis team with this necessary information for calculation of the PK parameters.  The PK analysis team calculated the PK parameters, and provided these results to the study statistician.  The statistician completed all statistical programming and data cleaning.  Upon receipt of the PK parameter results, the study statistician received the treatment sequence from the investigational pharmacy to analyze the data by formulation.  The study teams were blinded to study product administered although the product was not blinded to the PK clinical team or the individual.

# Study Assessment Schedule

Table P – Study Assessment Schedule

| Visit | 1 | 2 | 3 | 4 | 6 | 6 | 7 | 8 |
| --- | --- | --- | --- | --- | --- | --- | --- | --- |
| Study Day | -14 | 1 | 7+2 | 14+2 | 21+2 | 28+2 | 35+2 | 42+2 |
| Activity | Screen | Random-mization | PK 1 | PK 2 | PK 3 | PK 4 | PK 5 | PK 6/  EOS |
| Transplant background information | X |  |  |  |  |  |  |  |
| Vital Signs^1^ | X | X | X | X | X | X | X | X |
| Physical Exam^2^ | X |  |  |  |  |  |  | X |
| Randomization^3^ |  | X |  |  |  |  |  |  |
| Hematology/  Chemistry/  Local tacrolimus level^4^ | X | X | X | X | X | X | X | X |
| Genotype and MDR haplotype^5^ | X |  |  |  |  |  |  |  |
| Pregnancy Test^6^ | X |  |  |  |  |  |  |  |
| Tacrolimus 12hr PK^7^ |  |  | X | X | X | X | X | X |
| Meal Records^8^ |  |  | X | X | X | X | X | X |
| AEs/SAEs^9^ |  | X | X | X | X | X | X | X |
| Infections |  | X | X | X | X | X | X | X |
| Concomitant Medications^10^ | X | X | X | X | X | X | X | X |
| Immunosuppres-sive Medications | X | X | X | X | X | X | X | X |
| Kidney allograft rejection/biopsy^11^ |  |  |  |  |  |  |  |  |
| Graft loss |  |  |  |  |  |  |  |  |
| Dose Administration Records^12^ |  |  | X | X | X | X | X | X |
| Drug Accountability and collection of protein saver cards^13^ |  |  | X | X | X | X | X | X |
| MEMS cap assessement^14^ |  |  | X | X | X | X | X | X |
| Study Completion/  Termination |  |  |  |  |  |  |  | X |

^1^Vital signs include sitting blood pressure, weight, height (only at the screening visit) and temperature.

^2^Physical Exam: rectal, genital, and breast exam may be deferred unless clinically indicated.

^3^Randomization into one of six sequences will be consecutively assigned

^4^Hematology labs will include Hemoglobin, hematocrit, red blood cell count, white blood cell count with differential, and

platelet count. Chemistry labs will include blood urea, creatinine, glucose, carbon dioxide, total bilirubin, AST, ALT, alkaline phosphatase, sodium, potassium, chloride, calcium, total protein, and albumin. Tacrolimus level will be performed locally only for safety purposes.

^5^ Samples will be collected for drug metabolizing enzymes. Available donor samples will also be collected..

^6^Urine pregnancy test if positive test confirmed by serum BhCG.

^7^PK samples to be collected at the following time points: C0 (before morning dose) and then 20, 40, 60(1hr), 80, 100, 120(2hr), 140, 160, 180(3hr) minutes, 4, 5, 6, 8 and 12 hour after dosing. Actual samples collection times will be captured. +3 minute window is allowed for samples prior to 4hr and +5 minute window for samples after 4 hrs.

^8^ Meals will be provided in a controlled fashion with a light breakfast after the 3hr blood sample, lunch after the 6hr blood sample, and dinner after the 12hr blood sample. Patients were instructed to fast after midnight prior to the PK visit.

^9^ All abnormal findings will be collected as adverse events. Any abnormal finding meeting the criteria for SAE will be reported via proper channels.

^10^ All concomitant medications will be collected during the study.

^11^ Any kidney dysfunction episodes will result in a kidney biopsy at the discretion of the PI and Physician. Any liver dysfunction episodes will result in a liver biopsy at the discretion of the PI and Physician. Any biopsy data will be captured. No protocol biopsies will be performed.

^12^ Study drug dose and administration time will be captured at each visit.

^13^ Drug accountability will be performed by pill counts and patient diary review. Collection of daily trough blood samples via protein saver cards and documentation of time of collection will be reviewed in the patient diary. Eligibility of subsequent PK period will be assessed.

^14^ MEMS cap data will be downloaded. Eligibility of subsequent PK period will be assessed.

# Adherence Monitoring

Adherence monitoring was performed weekly by 3 different mechanisms during the study: 1) pill count, 2) diary review, and 3) Medication Event Monitoring System (MEMS^R^, AARDEX, Palo Alto, CA). The physician provided a individual specific dose for a 9-day supply with 5 refills. Each study week, the investigational drug pharmacy would dispense a 9-day supply to each participant. Upon arrival for each PK visit, the pill count was assessed to determine adherence. Reference was made to the individual diary for any necessary pill count reconciliations. Prior to the study, each individual received a MEMS cap programmed with a 1-hour window around the target morning and evening dosing time. MEMS is a bottle cap for electronic monitoring of study medication access. MEMS is similar to a prescription bottle, but contains a micro-electronic chip in the cap that registers dates/times when the bottle is opened and closed. Time-stamped medication events were stored in the MEMS and transferred to the PowerView software and a database (in combination with SQL2012, both Microsoft) that records the daily history of medication taking. This information was exported to SAS for statistical analysis. Patients were instructed to take their medication only from the MEMS bottle for the duration of the study, not to open the bottle unless they were taking a dose of medication at that time, and to close the bottle immediately after removing the prescribed dose. A standardized form was used during each download to capture information regarding extra openings, refills, and periods of nonuse. Adherence was defined as the number of times that doses of oral medication were taken as prescribed. Individuals non-adherent within 48 hours likely failed to present steady state tacrolimus concentration with the current formulation and were therefore considered unanalyzable.

# Pharmacokinetic Sample Handling and Storage

Pharmacokinetic samples were to be collected at C_0_ (before morning dose) and then 20, 40, 60 (1hr), 80, 100, 120 (2hr), 140, 160, 180 (3hr) minutes, 4, 5, 6, 8 and 12 hours after dosing. As whole blood is the matrix of choice, specimens were collected into tubes containing ethylene diamine tetraacetic acid (EDTA) anticoagulant. Heparin anticoagulation is not recommended because of the tendency to form clots on storage. Actual samples collection times were captured. Windows of ±3 minutes were allowed for samples prior to 4hr and ±5-minute window for samples after 4 hours. A minimum of 2mL of whole blood was collected and mixed well by repeated inversion of the collection tube. The tube was divided into two aliquots and labeled. Appropriate cold and moisture-resistant labels written with indelible ink was affixed to the polypropylene tubes. Each label included individual ID, date, PK visit number, and target sample collection time and actual collection time. Samples were frozen upright immediately on dry ice and transferred to a monitored -80°C temperature controlled freezer until shipment. One aliquot of all samples for each patient were batched and shipped together for blinded analysis at iC42 Clinical Research and Development (University of Colorado) on dry ice using an overnight courier. One aliquot on each patient was kept on site frozen until all samples for each patient had been completely analyzed at the bioanalytical laboratory. Samples stored at -80°C in an access-controlled and freezer at iC42 Clinical Research and Development and University of Cincinnati Transplant Clinical Research Laboratory until the final study report/ the last manuscript had been accepted. All freezers were constantly monitored, alert personnel automatically if the temperature increased above an alert threshold and samples will be transferred into a back-up freezer within one hour or less. In case electricity in the building was down, liquid nitrogen backup for cooling the freezers was available at iC42 Clinical Research and Development. All samples were handled, stored and archived following applicable iC42 Clinical Research and Development standard operation procedures.

# Study Investigators and other personnel

## Corresponding Author

Rita R. Alloway, PharmD, FCCP

Division Nephrology and Hypertension

Section of Transplantation

University of Cincinnati Medical Center

231 Albert Sabin Way, ML 585

MSB, Room 6023

Cincinnati OH 45267

Phone: 513-558-1658 / Fax: 513-558-4944 / Email Address: [rita.alloway@uc.edu](https://ucmail.uc.edu/owa/redir.aspx?C=TJa9941RGkae4ig0TUl71liZRAiDdM8Ii1HthaE3S3bqeczSVMYQ9tBtqxAvW7RNNc0N3DPbgEw.&URL=mailto%3arita.alloway%40uc.edu)

## Authors

Alexander Vinks, PharmD, PhD, FCP

Cincinnati Children’s Hospital Medical Center

Division of Clinical Pharmacology

3333 Burnet Avenue MLC 6018

Cincinnati, OH 45229-3039

Phone: 513-636-5833 / Fax: 513-636-4402 / Email Address: [Sander.Vinks@cchmc.org](mailto:Sander.Vinks@cchmc.org)

Tsuyoshi Fukuda, PhD

Cincinnati Children’s Hospital Medical Center

Division of Clinical Pharmacology

3333 Burnet Avenue MLC 6018

Cincinnati, OH 45229-3039

Phone: 513-636-5833 / Fax: 513-636-4402 / Email Address: [Tsuyoshi.Fukuda@cchmc.org](mailto:Tsuyoshi.Fukuda@cchmc.org)

Tomoyuki Mizuno, PhD

Cincinnati Children’s Hospital Medical Center

Division of Clinical Pharmacology

3333 Burnet Avenue MLC 6018

Cincinnati, OH 45229-3039

Phone: 513-636-5833 / Fax: 513-636-4402 / Email Address:Tomoyuki.Mizuno@cchmc.org

Eileen King, PhD

Cincinnati Children’s Hospital Medical Center

3333 Burnet Avenue

Cincinnati, OH  45229

513-803-1819

[Eileen.King@cchmc.org](mailto:Eileen.King@cchmc.org)

Yuanshu Zou, PhD

Cincinnati Children’s Hospital Medical Center

3333 Burnet Avenue

Cincinnati, OH  45229

513-803-1819

[yuanshu.zou@cchmc.org](mailto:yuanshu.zou@cchmc.org)

Wenlei Jiang, PhD

Office of Research and Standards, Office of Generic Drugs, Center for Drug Evaluation and Research, U.S. Food & Drug Administration, Silver Spring, Maryland, USA

10903 New Hampshire Ave., Bldg. 75

Silver Spring, MD 20993

Wenlei.jiang@fda.hhs.gov

E. Steve Woodle, M.D., F.A.C.S

University of Cincinnati Medical Center

Department of Surgery/Division of Transplantation

231 Albert Sabin Way, ML 558

Cincinnati, OH 45267

Phone: 513-585-6001 / Fax: 513-558-8689/ Email Address: [woodlees@uc.edu](mailto:woodlees@uc.edu)

Simon Tremblay, BPharm, MSc, PharmD, BCPS

Division Nephrology and Hypertension

Section of Transplantation

University of Cincinnati Medical Center

231 Albert Sabin Way, ML 585

Cincinnati OH 45267

Phone: 513-558-9967 / Fax: 513-558-8689 / Email Address: [simon.tremblay@uc.edu](mailto:simon.tremblay@uc.edu)

Jelena Klawitter, PhD

iC42 Clinical Research and Development

Department of Anesthesiology

University of Colorado Anschutz Medical Campus

Bioscience 2, Suite 200

12705 East Montview Boulevard

Aurora CO 80045

Phone: 303-724-5666 / Fax: 303-724-5662/ Email Address: [Jelena.klawitter@ucdenver.edu](mailto:Jelena.klawitter@ucdenver.edu)

Jost Klawitter, PhD

iC42 Clinical Research and Development

Department of Anesthesiology

University of Colorado Anschutz Medical Campus

Bioscience 2, Suite 200

12705 East Montview Boulevard

Aurora CO 80045

Phone: 303-724-5672 / Fax: 303-724-5662/ Email Address: [jost.klawitter@ucdenver.edu](mailto:jost.klawitter@ucdenver.edu)

Uwe Christians, MD, PhD

iC42 Clinical Research and Development

Department of Anesthesiology

University of Colorado Anschutz Medical Campus

Bioscience 2, Suite 200

12705 East Montview Boulevard

Aurora CO 80045

Phone: 303-724-5665 / Fax: 303-724-5662/ Email Address: [Uwe.Christians@ucdenver.edu](mailto:Uwe.Christians@ucdenver.edu)

## Consultants

Gautham Mogilishetty, MD

Division Nephrology and Hypertension

Section of Transplantation

University of Cincinnati Medical Center

231 Albert Sabin Way, ML 585

Cincinnati OH 45267

Phone: 513-558-6742 / Fax: 513-558-4944 / Email Address: [gautham.mogilishetty@uc.edu](mailto:gautham.mogilishetty@uc.edu)

Amit Govil, MD

Division Nephrology and Hypertension

Section of Transplantation

University of Cincinnati Medical Center

231 Albert Sabin Way, ML 585

Cincinnati OH 45267

Phone: 513-558-6742 / Fax: 513-558-4944 / Email Address: [amit.govil@uc.edu](mailto:amit.govil@uc.edu)

Bassam Abu Jawdeh, MD

Division Nephrology and Hypertension

Section of Transplantation

University of Cincinnati Medical Center

231 Albert Sabin Way, ML 585

Cincinnati OH 45267

Phone: 513-558-6742 / Fax: 513-558-4944 / Email Address: [bassam.abujawdeh@uc.edu](mailto:bassam.abujawdeh@uc.edu)

Michael Cardi, M.D.

The Kidney and Hypertension Center

2123 Auburn Avenue, Suite 404

Cincinnati, OH 45219

Phone: 513-241-5630 / Fax: 513-241-7146/ Email Address: [mike.cardi@gmail.com](mailto:mike.cardi@gmail.com)

Kenneth Sherman, M.D.

University of Cincinnati Medical Center

Division of Digestive Diseases

231 Albert Sabin Way, ML 0595

Phone: 513-558-3918 / Fax: 513-558-1744/ Email Address: [Kenneth.Sherman@uc.edu](mailto:Kenneth.Sherman@uc.edu)

Tiffany Kaiser, Pharm.D, BCPS

University of Cincinnati Medical Center

Department of Internal Medicine, Division of Digestive Disease

231 Albert Sabin Way, ML 0595

Cincinnati, OH 45267

Phone: 513-558-0821/ Email Address: [kaisertn@uc.edu](mailto:kaisertn@uc.edu)

Adele Rike-Shields, Pharm.D.

University of Cincinnati Medical Center

Department of Surgery/Division of Transplantation

231 Albert Sabin Way, ML 585

Cincinnati, OH 45219

Phone: 513-585-2145 / Fax: 513-558-3580/ Email Address: [rikea@uc.edu](mailto:rikea@uc.edu)

Touraj Shokati, PhD

iC42 Clinical Research and Development

Department of Anesthesiology

University of Colorado Anschutz Medical Campus

Bioscience 2, Suite 200

12705 East Montview Boulevard

Aurora CO 80045

Phone: 303-724-5670 / Fax: 303-724-5662

Randy Yeates

University of Iowa Pharmaceuticals

115 South Grand Avenue

Iowa City, IA 52242

Phone: 319-335-8674 / Fax: 319-335-9418/ Email Address: [randhall-yeates@uiowa.edu](mailto:randhall-yeates@uiowa.edu)

Samatha Franck-Rezac

University of Iowa Pharmaceuticals

115 South Grand Avenue

Iowa City, IA 52242

Phone: 319-335-8674 / Fax: 319-335-9418/ Email Address: [samantha-frack-rezac@uiowa.edu](mailto:samantha-frack-rezac@uiowa.edu)

Jennifer Rohan, MA

Children's Hospital Boston

Dept of Psychiatry, Fegan 8

300 Longwood Ave

Boston MA 02115

Phone: 617-355-6680/ Fax: 617-730-0319/ Email: [jennifer.rohan@childrens.harvard.edu](mailto:jennifer.rohan@childrens.harvard.edu)

Laszlo Endrenyi, PhD

University of Toronto

Department of Pharmacology and Toxicology

Medical Sciences Building, Room 4207
1 King's College Circle
Toronto, ON M5S 1A8

Canada

Phone: 416-978-8620/ Fax: 416-978-6395/ Email Address: [l.endrenyi@utoronto.ca](mailto:l.endrenyi@utoronto.ca)

Dennis Drotar, Ph.D.

Cincinnati Children’s Hospital Medical Center

Division of Behavioral Medicine and Psychology

3333 Burnet Avenue ML 7039

Cincinnati, OH 45229-3039

Phone: 513-636-3936/ Fax: 513-803-0415/ Email Address: [Dennis.Drotar@cchmc.org](mailto:Dennis.Drotar@cchmc.org)

## Study Coordinators

Elizabeth Cole, CCRP

University of Cincinnati

Department of Internal Medicine/Division of Nephrology

231 Albert Sabin Way, ML 585

Cincinnati, OH 45249

Stefanie Young, CCRP

University of Cincinnati

Department of Internal Medicine/Division of Nephrology

231 Albert Sabin Way, ML 585

Cincinnati, OH 45249

Lacey Thieken, BA, CCRP

University of Cincinnati

Department of Surgery/Division of Transplantation

231 Albert Sabin Way, ML 558

Cincinnati, OH 45249

Angela Horn

University of Cincinnati

Department of Internal Medicine/Division of Nephrology

231 Albert Sabin Way, ML 585

Cincinnati, OH 45249

Rachel Craddock-Jones, RN

University of Cincinnati

Department of Internal Medicine/Division of Nephrology

231 Albert Sabin Way, ML 585

Cincinnati, OH 45249

Lindsey Craddock, RN

University of Cincinnati

Department of Internal Medicine/Division of Nephrology

231 Albert Sabin Way, ML 585

Cincinnati, OH 45249

Lauren Noble

University of Cincinnati

Department of Internal Medicine/Division of Nephrology

231 Albert Sabin Way, ML 585

Cincinnati, OH 45249

## Investigational Drug Services

Judy Houston, RPh

Kanyetta Wansley, PharmD

Investigational Drug Services

234 Goodman Avenue, ML 0740

Dept of Pharmacy Services

Cincinnati, Ohio 45219

E-mail: [IDS-Pharmacy@UCHealth.com](mailto:IDS-Pharmacy@UCHealth.com)

## IRB/IEC Oversight

University of Cincinnati Institutional Review Board

University of Cincinnati Medical Center

University Hall, Suite 300

51 Goodman Drive

PO Box 210567

Cincinnati, OH 45221-0567

Phone: 513-558-5259

IRB # 202-4891

The Christ Hospital Institutional Review Board

The Christ Hospital

2139 Auburn Avenue, Room 3131, 3-North

Cincinnati, OH 45219

Phone: 513-585-2107

IRB # 13-26

FDA Research Involving Human Subjects Committee (RIHSC)

White Oak Bldg 32

Room 4286

10903 New Hampshire Ave

Silver Springs MD 20993

RIHSC # 13-018D

## Cincinnati Children’s Hospital and Medical Center Data Management

Judd Jacobs, BS, CCDM

Specialist - Clinical Data Management

Division of Biostatistics & Epidemiology Cincinnati Children's Hospital Medical Center

MLC 5041

3333 Burnet Ave.

Cincinnati, Ohio 45229

Tel: 513-803-4433/ E-mail: [judd.jacobs@cchmc.org](mailto:judd.jacobs@cchmc.org)

Ting Sa, PhD

Cincinnati Children’s Hospital Medical Center

3333 Burnet Avenue

Cincinnati, OH  45229

Tel: 513-803-1819/ E-mail: [ting.sa@cchmc.org](mailto:ting.sa@cchmc.org)

Pierce Kuhnell

Specialist - Clinical Data Management

Division of Biostatistics & Epidemiology Cincinnati Children's Hospital Medical Center

MLC 5041

3333 Burnet Ave.

Cincinnati, Ohio 45229

Tel: 513-803-4433/ Fax: 513-636-7509/ E-mail: [pierce.kuhnell@cchmc.org](mailto:pierce.kuhnell@cchmc.org)

## Food and Drug Administration

Xinyuan Zhang, PhD

Minori Kinjo, PharmD

Hyewon Kim, Ph.D.

Division of Quantitative Methods and Modeling

Office of Research and Standards/OGD/CDER/FDA

10903 New Hampshire Ave., Bldg. 75

Silver Spring, MD 20993

Phone: 240-402-9176

# References

1. Christians U, Jacobsen W, Serkova N, Benet LZ, Vidal C, Sewing KF, Manns MP, Kirchner GI. Automated, fast and sensitive quantification of drugs in blood by liquid chromatography-mass spectrometry with on-line extraction: immunosuppressants. J Chromatogr B 2000; 748: 41-53.
2. U.S. Department of Health and Human Services, Food and Drug Administration, Center for Drug Evaluation and Research and Center for Veterinary Medicine. (2001) Guidance for the Industry. Bioanalytical Method Validation. Version May 2001.

<http://www.fda.gov/downloads/Drugs/GuidanceComplianceRegulatoryInformation/Guidances/UCM070107.pdf>

1. U.S. Department of Health and Human Services, Food and Drug Administration. Bioanalytical Method Validation. Draft Guidance, 2013.

<http://www.fda.gov/downloads/drugs/guidancecomplianceregulatoryinformation/guidances/ucm368107.pdf>

1. Matuszewski BK, Constanzer ML, Chavez-Eng CM. Strategies for the assessment of matrix effect in quantitative bioanalytical methods based on HPLC–MS/MS. Anal Chem 2003; 75:3019–3030.
2. Mancinelli LM, Frassetto L, Floren LC, Dressler D, Carrier S, Bekersky I, Benet LZ, Christians U. The pharmacokinetics and metabolic disposition of tacrolimus: a comparison across ethnic groups. Clin Pharmacol Ther. 2001; 69:24-31.
3. Chitnis SD, Ogasawara K, Schniedewind B, Gohh RY, Christians U, Akhlaghi F. Concentration of tacrolimus and major metabolites in kidney transplant recipients as a function of diabetes mellitus and cytochrome P450 3A gene polymorphism. Xenobiotica. 2013; 43:641-9
4. Schüler W, Christians U, Schmieder P, Schiebel HM, Holze I, Sewing KF, Kessler H. Structural identification of 13-demethyl-FK506 and its isomers generated by in-vitro metabolism of FK506 using human liver microsomes. Helv Chim Acta, 1993; 76: 2288-2302
5. Sattler M, Guengerich FP, Yun CH, Christians U, Sewing KF, Cytochrome P4503A enzymes are responsible for biotransformation of FK506 and rapamycin in man and rat. Drug Metab Dispos, 1992; 20: 753-761
6. Christians U, Radeke HH, Kownatzki R, Schottmann R, Sewing KF. Isolation of an immunosuppressive metabolite of FK506. Clin Biochem, 1991; 24: 271-275
7. Dubbelboer IR, Pohanka A, Said R, Rosenborg S, Beck O. Quantification of tacrolimus and three demethylated metabolites in human whole blood using LC-ESI-MS/MS. Ther Drug Monit. 2012; 34: 134-142.
8. Shokati T, Bodenberger N, Gadpaille H, Schniedewind B, Vinks AA, Wenlei J, Alloway RR, Christians U. Quantification of the immunosuppressant tacrolimus on dried blood spots using LC-MS/MS. J Vis Exp (in print)
9. Kuypers DR, de Jonge H, Naesens M, Lerut E, Verbeke K, Vanrenterghem Y. CYP3A5 and CYP3A4 but not MDR1 single-nucleotide polymorphisms determine long-term tacrolimus disposition and drug-related nephrotoxicity in renal recipients. *Clin Pharmacol Ther.* 2007; 82(6):711-25.
10. Pallet N, Jannot AS, El Bahri M, Etienne I, Buchler M, de Ligny BH, Choukroun G, Colosio C, Thierry A, Vigneau C, Moulin B, Le Meur Y, Heng AE, Subra JF, Legendre C, Beaune P, Alberti C, Loriot MA, Thervet E. Kidney transplant recipients carrying the CYP3A4*22 allelic variant have reduced tacrolimus clearance and often reach supratherapeutic tacrolimus concentrations. *Am J Transplant.* 2015; 15(3):800-5.
11. Elens L, Hesselink DA, Bouamar R, Budde K, de Fijter JW, De Meyer M, Mourad M, Kuypers DR, Haufroid V, van Gelder T, van Schaik RH. Impact of POR*28 on the pharmacokinetics of tacrolimus and cyclosporine A in renal transplant patients. *Ther Drug Monit.* 2014; 36(1):71-9.
12. Common Toxicity Criteria for Adverse Events (CTCAEv4.0). <http://ctep.cancer.gov/protocolDevelopment/electronic_applications/docs/ctcae_4_with_lay_terms.pdf>. Accessed Nov 2015.
