# Supplementary material for: Bioequivalence between innovator and generic tacrolimus in liver and kidney transplant recipients: A randomized, crossover clinical trial
Source: PLoS Med. 2017 Nov 14;14(11):e1002428. doi: 10.1371/journal.pmed.1002428 (PMC5685573; doi:10.1371/journal.pmed.1002428)
Supplement: S2 Appendix — (DOCX) [file pmed.1002428.s005.docx]

**S2. Appendix**

**Bioequivalence Between Innovator and Generic Tacrolimus Formulations in Liver and Kidney Transplant Recipients: a Randomized Crossover Clinical Trial**

**Supporting Information – Results**

**Table of Contents**

[A. Population Demographics 3](#_Toc497297617)

[Table Q – Population Demographics – Intent to Treat Population 3](#_Toc497297618)

[B. Adherence Rates 4](#_Toc497297619)

[Table R – Adherence Rates – Intent to Treat Population 4](#_Toc497297620)

[Table S – Adherence Rates – Analyzed Population 4](#_Toc497297621)

[C. Individual Tacrolimus Concentration Time Plots by Tacrolimus Product 5](#_Toc497297622)

[Fig D – Individual Tacrolimus Concentration Time Plots by Tacrolimus Product – Kidney Individuals 5](#_Toc497297623)

[Fig E – Individual Tacrolimus Concentration Time Plots by Tacrolimus Product – Liver Individuals 13](#_Toc497297624)

[D. Tacrolimus AUC Results by Product for CYP3A5 Genotype for Kidney and Liver Individuals....... 24](#_Toc497297625)

[Fig F – Kidney Individuals Expressing CYP3A5 (*1/*3, *1/*1), Tacrolimus AUC Results (n=12) 24](#_Toc497297626)

[Fig G – Kidney Individuals Not Expressing CYP3A5 (*3/*3) Tacrolimus AUC Results (n=23) 25](#_Toc497297627)

[Fig H – Liver Individuals Expressing CYP3A5 (*1/*3; there were no individuals with the *1/*1 genotype), Tacrolimus AUC results (n=6) 26](#_Toc497297628)

[Fig I – Liver Individuals Not Expressing CYP3A5 (*3/*3), Tacrolimus AUC Results (n=30) 27](#_Toc497297629)

[Fig J – Kidney Donors Expressing CYP3A5 (*1/*3, *1/*1), Tacrolimus AUC Results (n=5) 27](#_Toc497297630)

[Fig K – Kidney Donors Not Expressing CYP3A5 (*3/*3), Tacrolimus AUC results (n=12) 28](#_Toc497297631)

[Fig L – Liver Donors Expressing CYP3A5 (*1/*3, *1/*1), Tacrolimus AUC Results (n=10) 28](#_Toc497297632)

[Fig M – Liver Donors Expressing Not CYP3A5 (*3/*3) Tacrolimus AUC Results (n= 14) 29](#_Toc497297633)

[E. Tacrolimus AUC results by product for *ABCB1* transporter genotype for Kidney and Liver Individuals 30](#_Toc497297634)

[Fig N – Kidney Individuals Tacrolimus Product AUC Results by *ABCB1* 3435 genotype C/C, C/T, and T/T 30](#_Toc497297635)

[Fig O – Liver Individuals Tacrolimus Product AUC Results by ABCB1 3435 Genotype C/C, C/T, and T/T 31](#_Toc497297636)

[F. AUC Bioequivalence by Subgroup Testing 32](#_Toc497297637)

[Fig P – Kidney Individuals AUC Bioequivalence Testing by Subgroups 32](#_Toc497297638)

[Fig PA – Generic Hi *versus* Innovator 32](#_Toc497297639)

[Fig PB – Generic Lo *versus* Innovator 33](#_Toc497297640)

[Fig PC – Generic Hi *versus* Generic Lo 34](#_Toc497297641)

[Fig Q – Liver Individuals AUC Bioequivalence Testing by Subgroups 35](#_Toc497297642)

[Fig QA – Generic Hi *versus* Innovator 35](#_Toc497297643)

[Fig QB – Generic Lo *versus* Innovator 36](#_Toc497297644)

[Fig QC– Generic Hi vs Generic Lo 37](#_Toc497297645)

[G. 13-O Desmethyl Tacrolimus AUC Scaled Average Bioequivalence results for Kidney and Liver Individuals 38](#_Toc497297646)

[Table T – Kidney Individuals 13-O-Desmethyl Tacrolimus AUC Scaled Average Bioequivalence Results 39](#_Toc497297647)

[Table U – Liver Individuals 13-O Desmethyl Tacrolimus AUC Scaled Average Bioequivalence Results 40](#_Toc497297648)

[S. Safety Information 41](#_Toc497297649)

[Table V – Adverse Events in Individuals with a Kidney and Liver Transplant Coded by CTCAE v4.0 Disorder Classification 42](#_Toc497297650)

[Fig R – Kidney Function by PK Period in Kidney Individuals 43](#_Toc497297651)

[Fig S – Kidney Function by Tacrolimus Product in Kidney Individuals 44](#_Toc497297652)

[Fig T – Kidney Function by PK Period in Liver Individuals 45](#_Toc497297653)

[Fig U – Kidney Function by Tacrolimus Product in Liver Individuals 46](#_Toc497297654)

[Fig V – Liver Function by PK Period in Liver Individuals 47](#_Toc497297655)

[Fig W – Liver Function by Tacrolimus Product in Liver Individuals 48](#_Toc497297656)

# Population Demographics

Table Q – Population Demographics – Intent to Treat Population

| **Variable** | **Kidney Individuals (n=42)** | **Liver Individuals (n= 40)** |
| --- | --- | --- |
| **Age (yrs) median (IQR)** | 51 (38.0, 59.0) | 58 (51.0, 62.0) |
| **Gender (male)** | 66.7% | 50% |
| **Race (African American)** | 16.7% | 5% |
| **Transplant Donor Type** |  |  |
| **Deceased** | 26.2% | 100% |
| **Living Related** | 33.3% | 0% |
| **Living Unrelated** | 40.5% | 0% |
| **Time post transplant (yrs) median (IQR)** | 4.5 (3.8, 7.9) | 3.2 (1.9, 7.0) |
| **Presence of Diabetes (%)** | 38.1 | 27.5 |
| **Maintenance Immunosuppression** |  |  |
| **Steroids (%)** | 14.3 | 7.5 |
| **Mycophenolic acid (%)** | 100 | 87.5 |
| **Median Tacrolimus Dose (mg/day)** | 4 (4.0, 7.0) | 4 (3.0, 6.0) |

# Adherence Rates

Table R – Adherence Rates – Intent to Treat Population

|  | **Entire Sample** | **Kidney Transplant** | **Liver Transplant** |
| --- | --- | --- | --- |
| **Adherence %, mean ± SD** | 99.65 ± 0.86 | 99.56 ± 1.07 | 99.75 ± 0.56 |
| **Minimum Adherence %** | 94.19 | 94.19 | 97.62 |
| **Maximum Adherence %** | 100.00 | 100.00 | 100.00 |

Table S – Adherence Rates – Analyzed Population

|  | **Entire Sample** | **Kidney Transplant** | **Liver Transplant** |
| --- | --- | --- | --- |
| **Adherence %, M ± SD** | 99.75 ± 0.51 | 99.70 ± 0.59 | 99.81 ± 0.44 |
| **Minimum Adherence %** | 97.67 | 97.67 | 98.81 |
| **Maximum Adherence %** | 100.00 | 100.00 | 100.00 |

# Individual Tacrolimus Concentration Time Plots by Tacrolimus Product

Fig D – Individual Tacrolimus Concentration Time Plots by Tacrolimus Product – Kidney Individuals

Fig E – Individual Tacrolimus Concentration Time Plots by Tacrolimus Product – Liver Individuals

# Tacrolimus AUC Results by Product for CYP3A5 Genotype for Kidney and Liver Individuals.......

Fig F – Kidney Individuals Expressing CYP3A5 (*1/*3, *1/*1), Tacrolimus AUC Results (n=12)

**
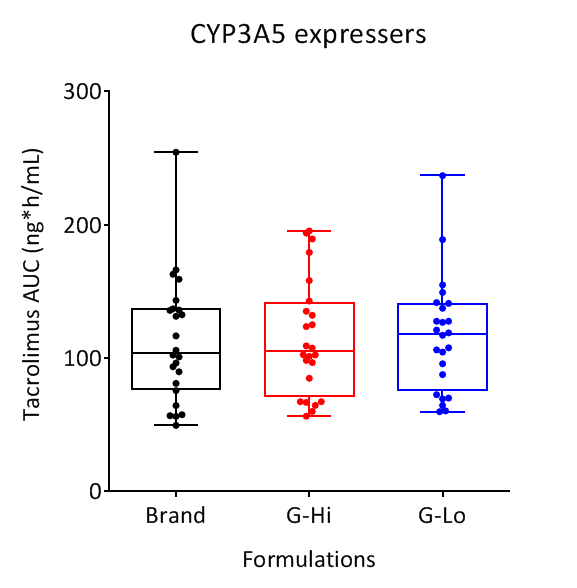
**

p > 0.05

Fig G – Kidney Individuals Not Expressing CYP3A5 (*3/*3) Tacrolimus AUC Results (n=23)

**
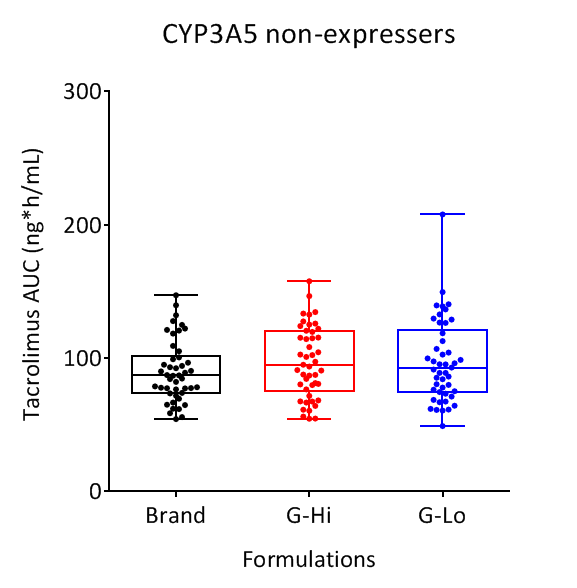
**

p > 0.05

Fig H – Liver Individuals Expressing CYP3A5 (*1/*3; there were no individuals with the *1/*1 genotype), Tacrolimus AUC results (n=6)

*
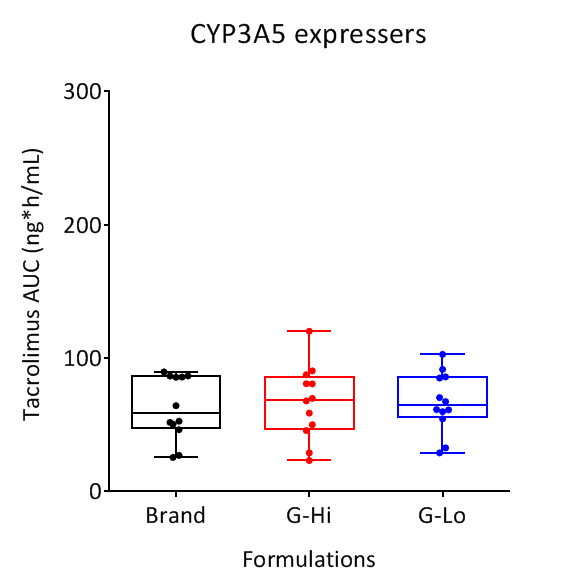
*

p > 0.05

Fig I – Liver Individuals Not Expressing CYP3A5 (*3/*3), Tacrolimus AUC Results (n=30)

*
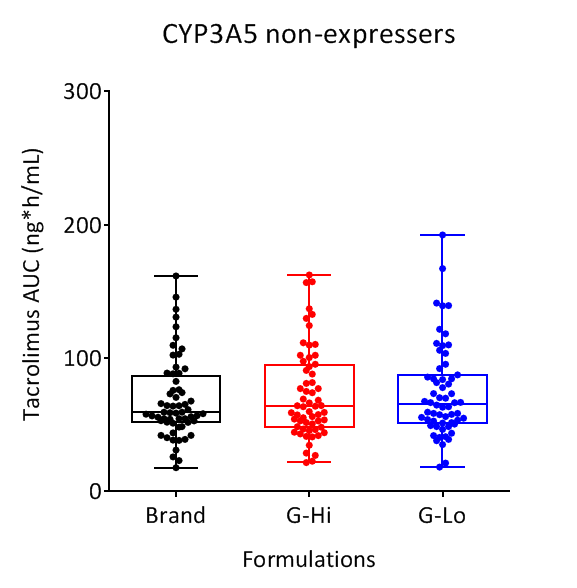
*

p > 0.05

Fig J – Kidney Donors Expressing CYP3A5 (*1/*3, *1/*1), Tacrolimus AUC Results (n=5)

**
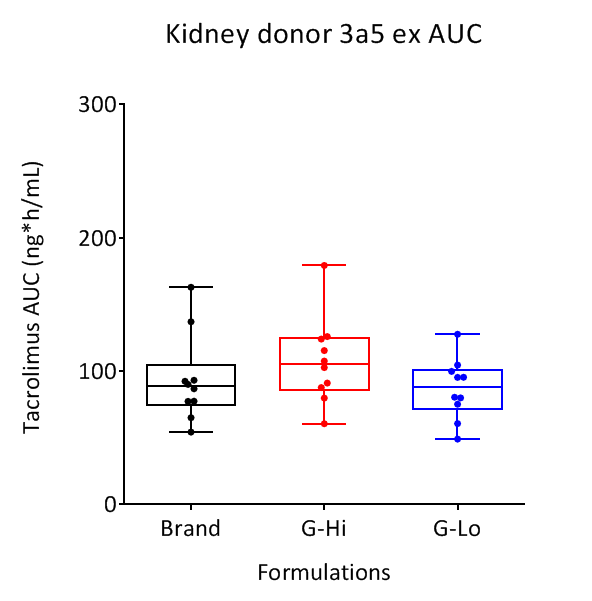
**

p > 0.05

Fig K – Kidney Donors Not Expressing CYP3A5 (*3/*3), Tacrolimus AUC results (n=12)

*
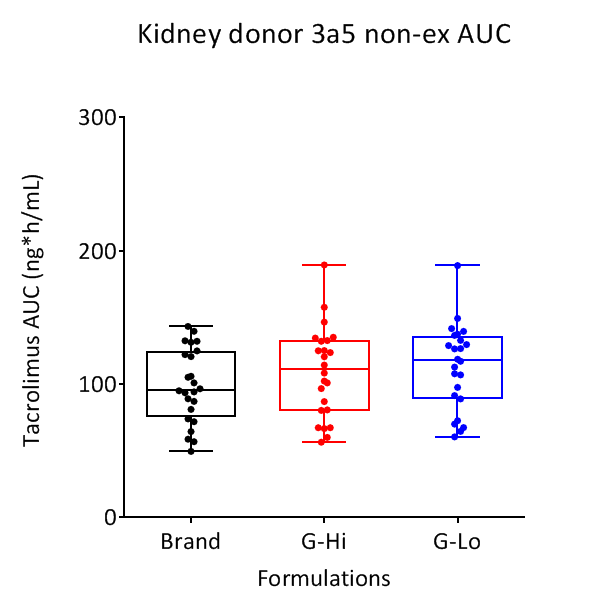
*

p > 0.05

Fig L – Liver Donors Expressing CYP3A5 (*1/*3, *1/*1), Tacrolimus AUC Results (n=10)

*
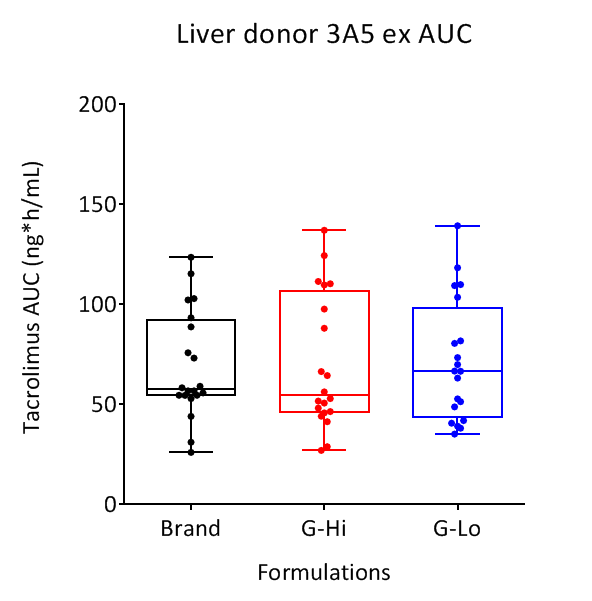
*

p > 0.05

Fig M – Liver Donors Expressing Not CYP3A5 (*3/*3) Tacrolimus AUC Results (n= 14)

p > 0.05

*
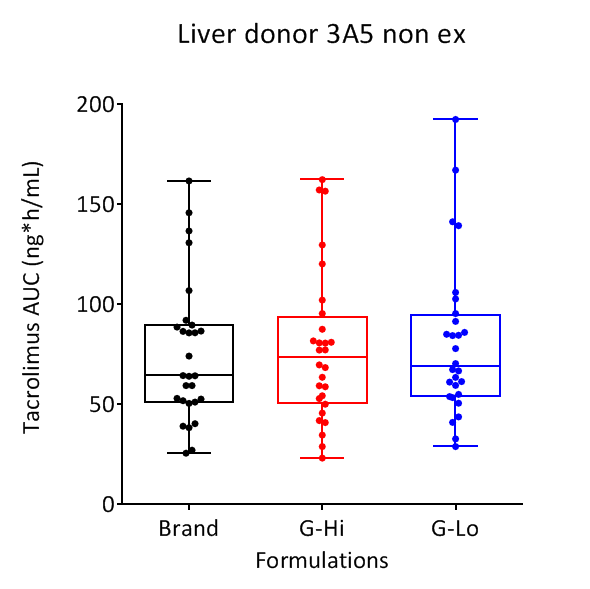
*

# Tacrolimus AUC results by product for *ABCB1* transporter genotype for Kidney and Liver Individuals

Fig N – Kidney Individuals Tacrolimus Product AUC Results by *ABCB1* 3435 genotype C/C, C/T, and T/T

n=10

n=16

n=9

p > 0.05

p > 0.05

p > 0.05

Fig O – Liver Individuals Tacrolimus Product AUC Results by ABCB1 3435 Genotype C/C, C/T, and T/T

**

p > 0.05

n=8

n=23

n=5

p > 0.05

p > 0.05

# AUC Bioequivalence by Subgroup Testing

Fig P – Kidney Individuals AUC Bioequivalence Testing by Subgroups

### Fig PA – Generic Hi *versus* Innovator

Kidney Individuals AUC Bioequivalence Testing by Subgroups Generic Hi *versus* Innovator. Calculated product confidence interval with SCABE limits denoted by “x” symbols. UCL σ_WT_/σ_WR_ ^=^ Upper 90% confidence interval for ratio of the within subject standard deviation of the test product to the reference product.

### Fig PB – Generic Lo *versus* Innovator

Kidney Individuals AUC Bioequivalence Testing by Subgroups Generic Lo v*ersus* Innovator Calculated product confidence interval with SCABE limits denoted by “x” symbols. UCL σWT/σWR = Upper 90% confidence interval for ratio of the within subject standard deviation of the test product to the reference product.

### Fig PC – Generic Hi *versus* Generic Lo

Kidney Individuals AUC Bioequivalence Testing by Subgroups Generic Hi vs Generic Lo. Calculated product confidence interval with SCABE limits denoted by “x” symbols. UCL σWT/σWR = Upper 90% confidence interval for ratio of the within subject standard deviation of the test product to the reference product.

Fig Q – Liver Individuals AUC Bioequivalence Testing by Subgroups

### Fig QA – Generic Hi *versus* Innovator

Liver Individuals AUC Bioequivalence Testing by Subgroups Generic Hi vs Innovator. Calculated product confidence interval with SCABE limits denoted by “x” symbols. UCL σ_WT_/σ_WR_ ^=^ Upper 90% confidence interval for ratio of the within subject standard deviation of the test product to the reference product.

### Fig QB – Generic Lo *versus* Innovator

Liver Individuals AUC Bioequivalence Testing by Subgroups Generic Lo *versus* Innovator Calculated product confidence interval with SCABE limits denoted by “x” symbols. UCL σWT/σWR = Upper 90% confidence interval for ratio of the within subject standard deviation of the test product to the reference product.

### Fig QC– Generic Hi vs Generic Lo

Liver Individuals AUC Bioequivalence Testing by Subgroups Generic Hi *versus* Generic Lo. Calculated product confidence interval with SCABE limits denoted by “x” symbols. UCL σWT/σWR = Upper 90% confidence interval for ratio of the within subject standard deviation of the test product to the reference product.

# 13-O Desmethyl Tacrolimus AUC Scaled Average Bioequivalence results for Kidney and Liver Individuals

Evaluation of metabolite concentrations were conducted only on AUC parameters to quantitate extent of metabolite exposure. As C_max_ is considered a surrogate marker of the rate of absorption in bioequivalence, the analysis of the C_max_ of 13-O-desmethyl tacrolimus would be irrelevant, as 13-O-desmethyl tacrolimus is not absorbed. Instead it is generated *in vivo* by cytochrome P4503A-mediated biotransformation of tacrolimus.

Table T – Kidney Individuals 13-O-Desmethyl Tacrolimus AUC Scaled Average Bioequivalence Results

| **Reference** | **Test** | **AUC**  **Geometric mean ratio (%)** | **Reference**  **Within Subject variability**  **(s_WR,_ %)** | **Observed**  **90%**  **Confidence Interval** | **Confidence Bound** | **SCABE**  **limit** | **Variability Comparison (^1^statistics of σ_WT_/σ_WR_)** |
| --- | --- | --- | --- | --- | --- | --- | --- |
| innovator | Generic Hi | 110.15 | 24.40 | 104.38-116.23 | -0.033 | 80.00-125.00 | 1.70 |
| Innovator | Generic Lo | 96.81 | 24.40 | 90.29-103.80 | -0.044 | 80.00-125.00 | 1.02 |
| Generic  hi | Generic lo | 87.89 | 30.85 | 82.49-93.64 | -0.051 | 80.00-125.00 | 0.81 |
| Generic  lo | Generic hi | 113.78 | 18.51 | 106.79-121.23 | -0.0020 | 82.28-121.53 | 2.24 |

^1^The upper limit of the 90% confidence interval of the ratio of within-subject standard deviation of test product to reference product, σ_WT_/σ_WR._

Table U – Liver Individuals 13-O Desmethyl Tacrolimus AUC Scaled Average Bioequivalence Results

| **Reference** | **Test** | **AUC**  **Geometric mean ratio (%)** | **Reference**  **Within Subject variability**  **(s_WR,_ %)** | **Observed**  **90%**  **Confidence Interval** | **Confidence Bound** | **SCABE**  **limit** | **Variability Comparison (^1^statistics of σ_WT_/σ_WR_)** |
| --- | --- | --- | --- | --- | --- | --- | --- |
| innovator | Generic Hi | 98.41 | 24.25 | 92.82-104.33 | -0.045 | 80.00-125.00 | 1.38 |
| Innovator | Generic Lo | 104.68 | 24.25 | 99.27-110.39 | -0.043 | 80.00-125.00 | 1.20 |
| Generic  hi | Generic lo | 106.38 | 24.97 | 99.27-113.99 | -0.041 | 80.00-125.00 | 1.17 |
| Generic  lo | Generic hI | 94.00 | 21.79 | 87.73-100.73 | -0.029 | 80.00-125.00 | 1.53 |

^1^The upper limit of the 90% confidence interval of the ratio of within-subject standard deviation of test product to reference product, σ_WT_/σ_WR._

# S. Safety Information

Adverse event information was collected at each study visits. Events were collected in lay terms and then classified by Common Toxicity Criteria for Adverse Events (CTCAEv4.0). [15] The Table X describes the adverse events by organ transplant type and tacrolimus product.

Table V – Adverse Events in Individuals with a Kidney and Liver Transplant Coded by CTCAE v4.0 Disorder Classification

| **Disorder Classification** | **Innovator** | | **Generic Hi** | | **Generic Lo** | |
| --- | --- | --- | --- | --- | --- | --- |
|  | **Kidney** | **Liver** | **Kidney** | **Liver** | **Kidney** | **Liver** |
| Nervous system | 1 (1) | 6 (6) | 2 (2) | 5 (6) | 1 (1) | 3 (4) |
| Blood and lymphatic system |  |  |  |  |  | 1 (1) |
| Metabolism and nutrition | 4 (4) |  | 3 (3) | 2 (2) | 1 (1) | 1 (1) |
| Gastrointestinal | 1 (1) | 6 (8) | 1 (1) | 2 (2) | 7 (9) | 4 (4) |
| Respiratory, thoracic and mediastinal | 1 (2) |  | 2 (2) | 1 (1) | 1 (2) |  |
| Musculoskeletal and connective tissue |  |  |  |  |  |  |
| Reproductive system and breast disorders |  |  |  | 1 (1) |  |  |
| Infections and infestations |  |  | 1 (1) |  |  |  |
| Skin and subcutaneous tissue | 1 (1) |  |  |  |  |  |
| Psychiatric |  |  |  |  | 1 (1) |  |
| Vascular | 1 (1) | 1 (1) | 1 (1) | 1 (1) |  |  |
| Investigations | 4 (5) |  | 1 (1) |  | 1 (1) |  |
| Eye disorder |  |  |  | 1 (1) |  |  |
| General disorders and administrative site conditions | 5 (6) | 1 (1) | 4 (4) | 1 (1) | 6 (7) | 8 (8) |

n = number of individuals with at least one occurrence of each adverse event

nAE= total number of times the AE occurs across all individuals

Fig R – Kidney Function by PK Period in Kidney Individuals

**
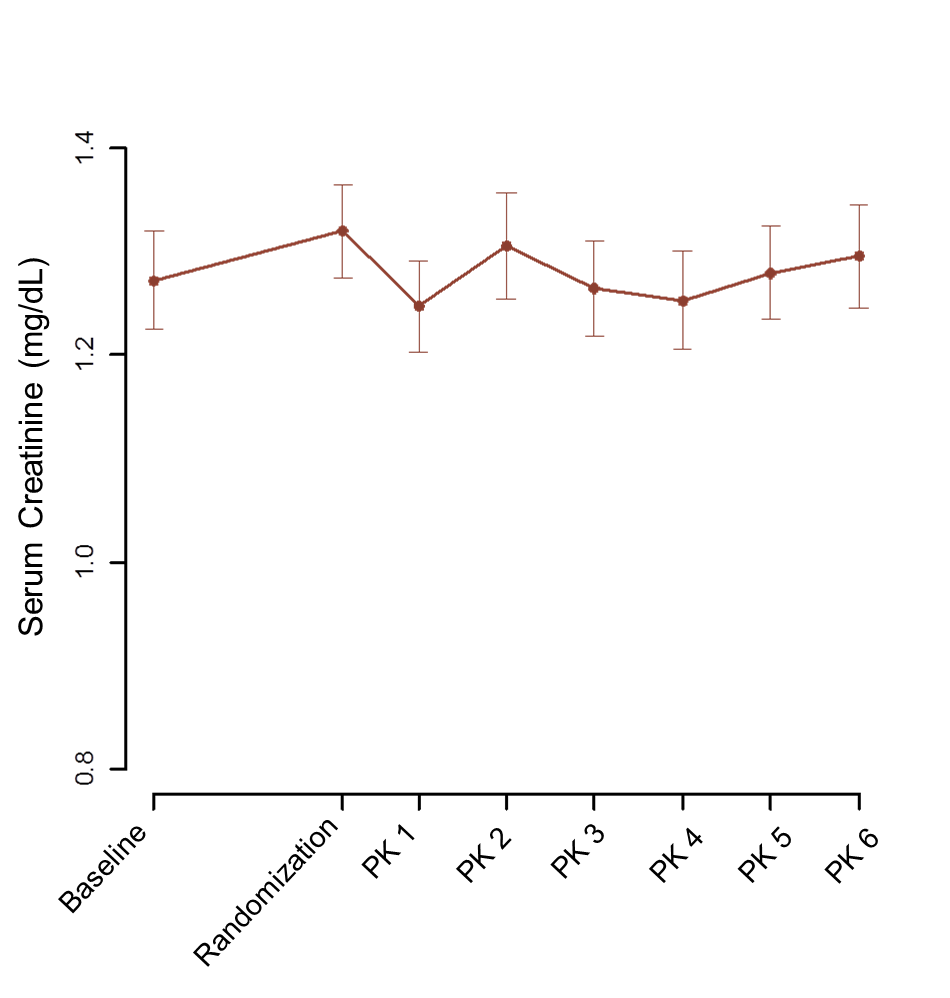
**

(mean ± SEM)

Fig S – Kidney Function by Tacrolimus Product in Kidney Individuals


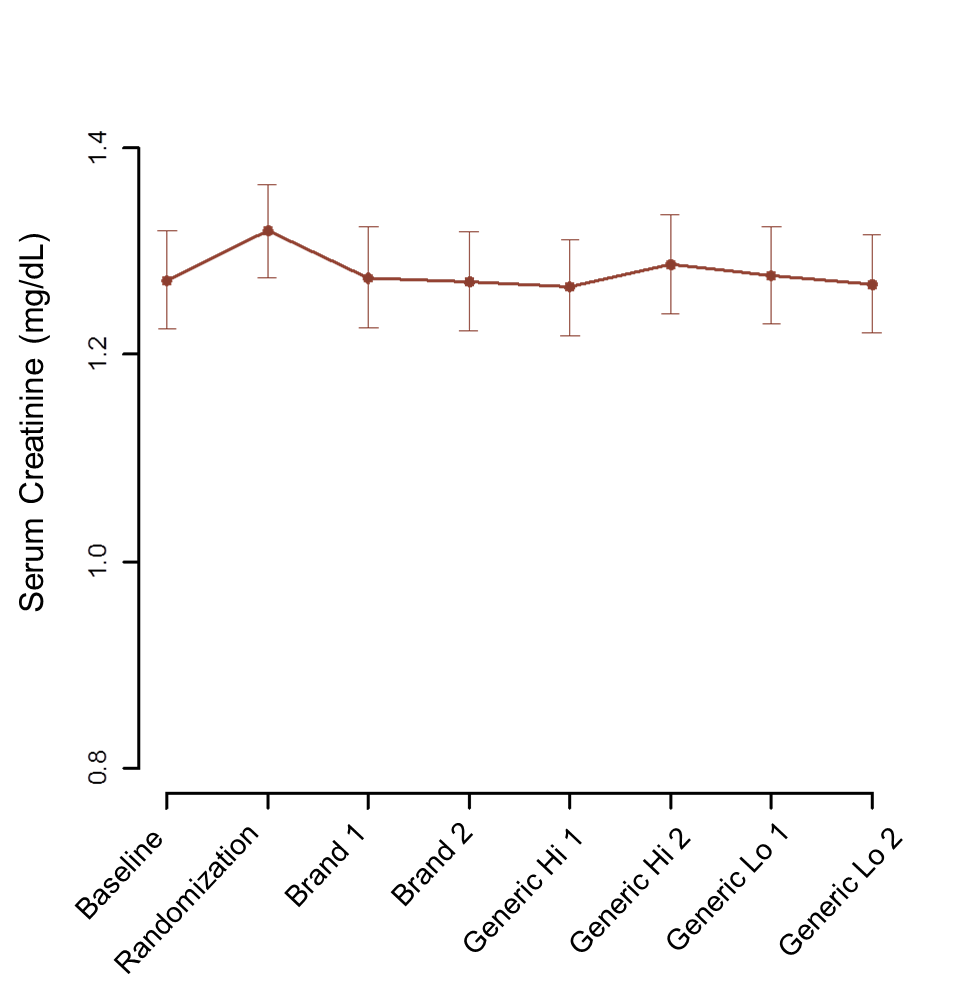


(mean ± SEM)

Fig T – Kidney Function by PK Period in Liver Individuals

*
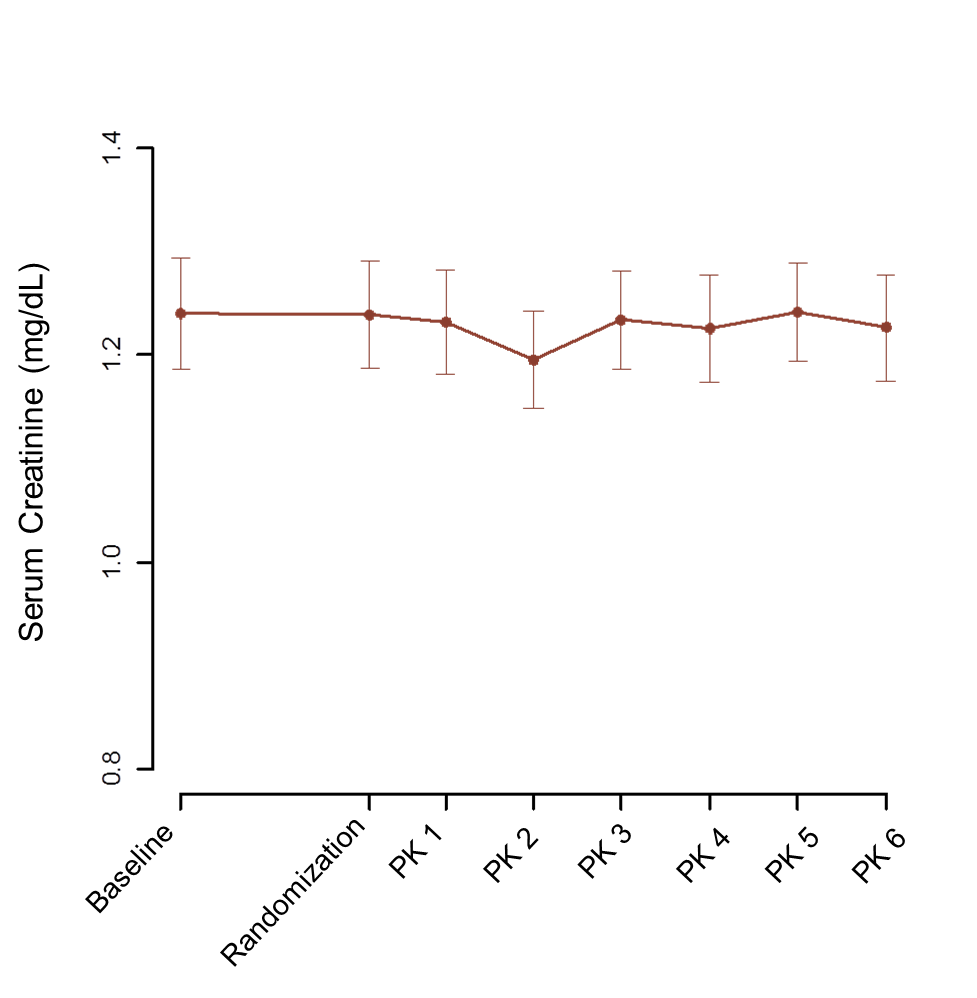
*

(mean ± SEM)

Fig U – Kidney Function by Tacrolimus Product in Liver Individuals


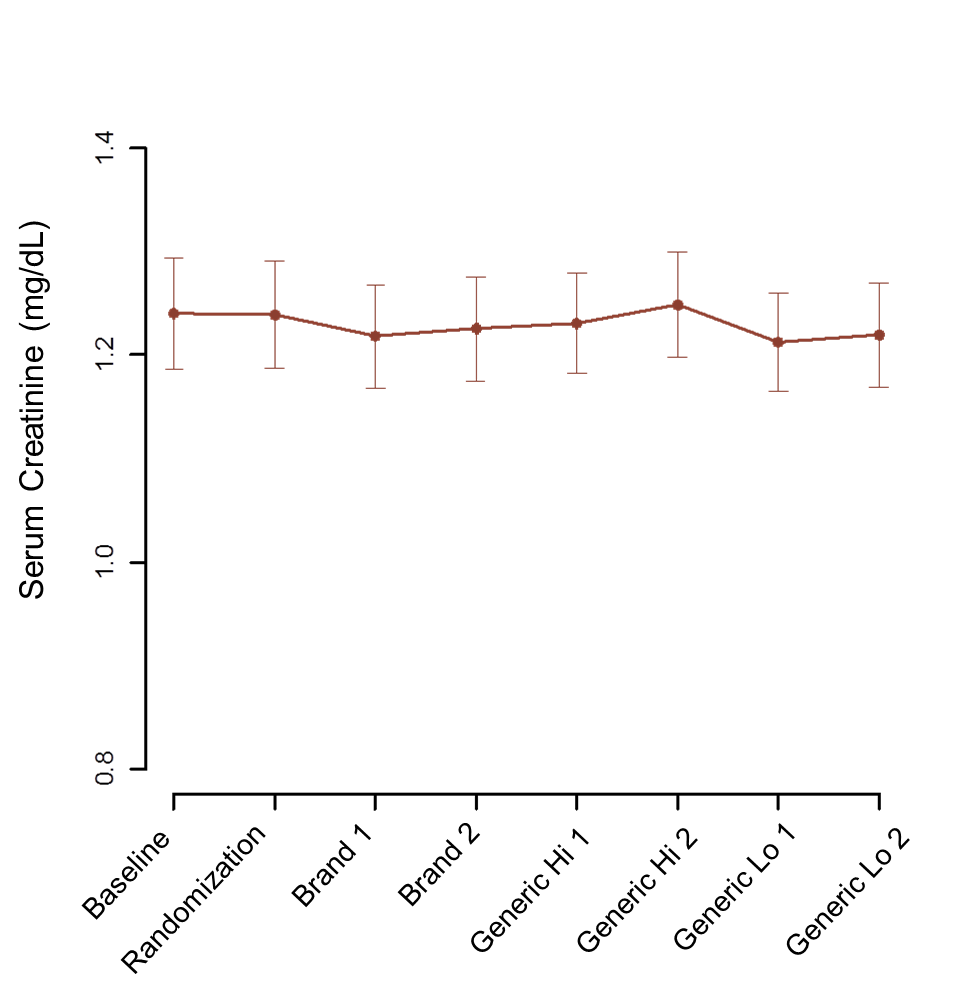


(mean ± SEM)

Fig V – Liver Function by PK Period in Liver Individuals

*
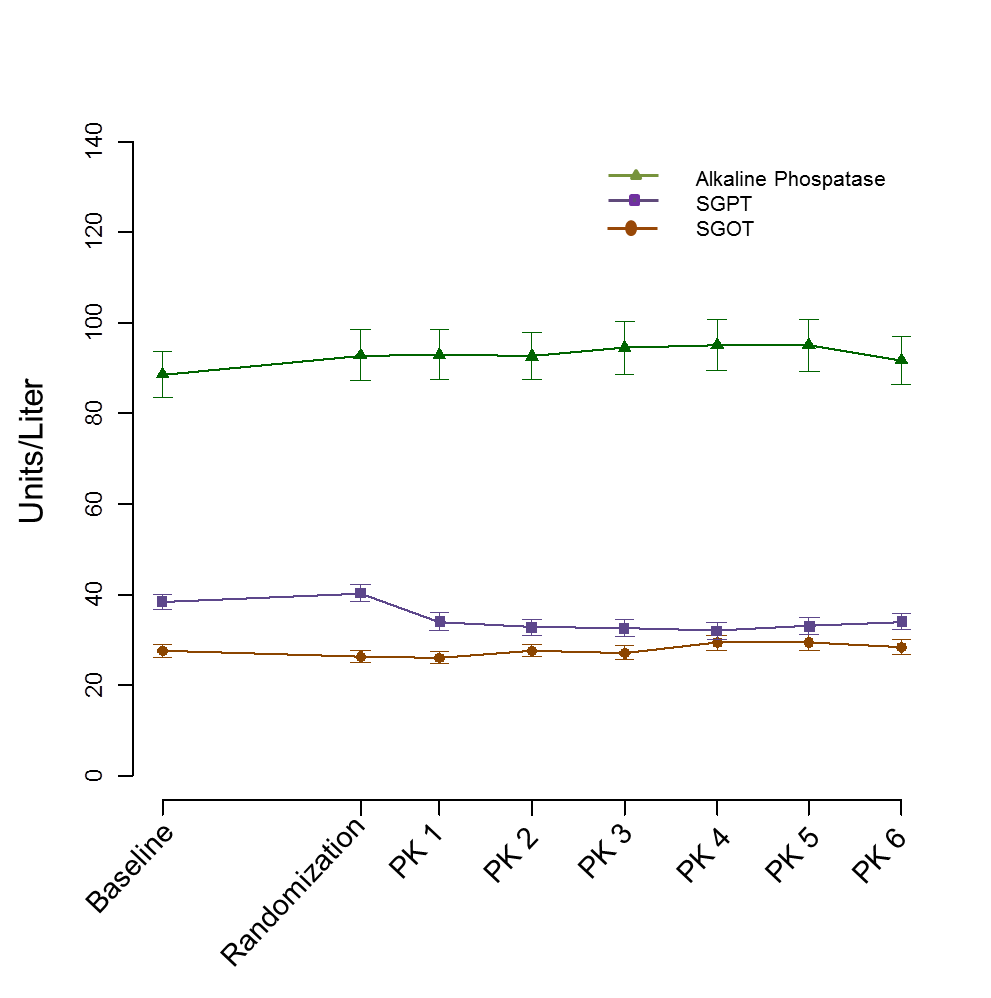
*

(mean ± SEM)

Fig W – Liver Function by Tacrolimus Product in Liver Individuals


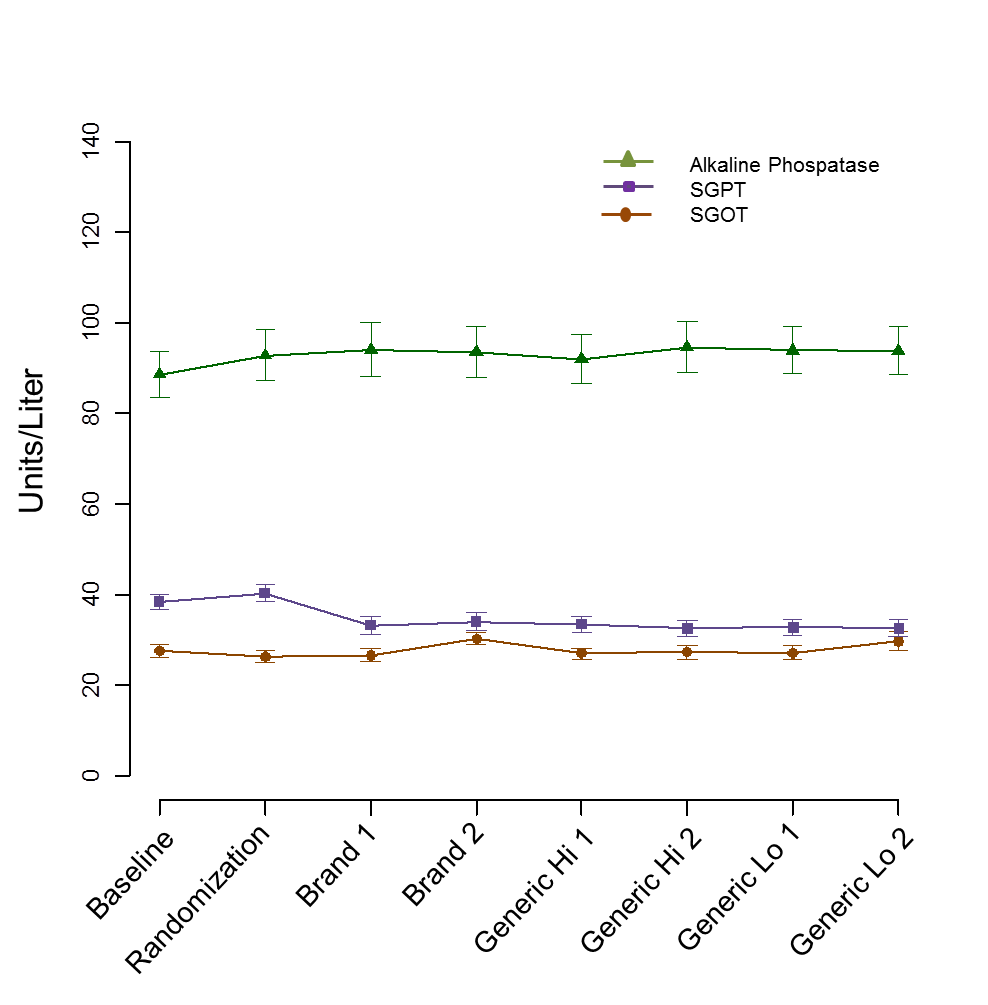


(mean ± SEM)
